# Retinal pathological features and proteome signatures of Alzheimer’s disease

**DOI:** 10.1007/s00401-023-02548-2

**Published:** 2023-02-11

**Authors:** Yosef Koronyo, Altan Rentsendorj, Nazanin Mirzaei, Giovanna C. Regis, Julia Sheyn, Haoshen Shi, Ernesto Barron, Galen Cook-Wiens, Anthony R. Rodriguez, Rodrigo Medeiros, Joao A. Paulo, Veer B. Gupta, Andrei A. Kramerov, Alexander V. Ljubimov, Jennifer E. Van Eyk, Stuart L. Graham, Vivek K. Gupta, John M. Ringman, David R. Hinton, Carol A. Miller, Keith L. Black, Antonino Cattaneo, Giovanni Meli, Mehdi Mirzaei, Dieu-Trang Fuchs, Maya Koronyo-Hamaoui

**Affiliations:** 1 Department of Neurosurgery, Maxine Dunitz Neurosurgical Research Institute, Cedars-Sinai Medical Center, 127 S. San Vicente Blvd., Los Angeles, CA 90048 USA; 2 Doheny Eye Institute, University of California Los Angeles, Los Angeles, CA USA; 3 Biostatistics and Bioinformatics Research Center, Cedars-Sinai Medical Center, Los Angeles, CA 90048 USA; 4 Norris Comprehensive Cancer Center, Keck School of Medicine, University of Southern California, Los Angeles, CA USA; 5 Clem Jones Centre for Ageing Dementia Research, Queensland Brain Institute, The University of Queensland, Brisbane, QLD Australia; 6 Institute for Memory Impairments and Neurological Disorders, University of California, Irvine, CA USA; 7 Department of Cell Biology, Harvard Medical School, Boston, USA; 8 School of Medicine, Deakin University, Victoria, Australia; 9 Department of Biomedical Sciences and Eye Program, Board of Governors Regenerative Medicine Institute, Cedars-Sinai Medical Center, Los Angeles, CA USA; 10 Departments of Neurology and Biomedical Sciences, Division of Applied Cell Biology and Physiology, Cedars-Sinai Medical Center, 127 S. San Vicente Blvd., Los Angeles, CA USA; 11 Barbra Streisand Women’s Heart Center, Cedars-Sinai Medical Center, Los Angeles, CA USA; 12 Department of Medicine, Cedars-Sinai Medical Center, Los Angeles, CA USA; 13 Save Sight Institute, University of Sydney, Sydney, NSW Australia; 14 Macquarie Medical School, Faculty of Medicine, Health and Human Sciences, Macquarie University, Sydney, NSW Australia; 15 Department of Neurology, Keck School of Medicine of USC, Los Angeles, CA USA; 16 Departments of Pathology and Ophthalmology, Keck School of Medicine, USC Roski Eye Institute, University of Southern California, Los Angeles, CA USA; 17 Department of Pathology Program in Neuroscience, Keck School of Medicine, University of Southern California, Los Angeles, CA USA; 18 European Brain Research Institute (EBRI), Viale Regina Elena, Rome, Italy

**Keywords:** Ocular abnormalities, Eye, Neurodegenerative disorders, S100β, GFAP, IBA1, scFvA13-intraneuronal oligomers, Immune responses

## Abstract

Alzheimer’s disease (AD) pathologies were discovered in the accessible neurosensory retina. However, their exact nature and topographical distribution, particularly in the early stages of functional impairment, and how they relate to disease progression in the brain remain largely unknown. To better understand the pathological features of AD in the retina, we conducted an extensive histopathological and biochemical investigation of postmortem retina and brain tissues from 86 human donors. Quantitative examination of superior and inferior temporal retinas from mild cognitive impairment (MCI) and AD patients compared to those with normal cognition (NC) revealed significant increases in amyloid β-protein (Aβ_42_) forms and novel intraneuronal Aβ oligomers (AβOi), which were closely associated with exacerbated retinal macrogliosis, microgliosis, and tissue atrophy. These pathologies were unevenly distributed across retinal layers and geometrical areas, with the inner layers and peripheral subregions exhibiting most pronounced accumulations in the MCI and AD versus NC retinas. While microgliosis was increased in the retina of these patients, the proportion of microglial cells engaging in Aβ uptake was reduced. Female AD patients exhibited higher levels of retinal microgliosis than males. Notably, retinal Aβ_42_, S100 calcium-binding protein B^+^ macrogliosis, and atrophy correlated with severity of brain Aβ pathology, tauopathy, and atrophy, and most retinal pathologies reflected Braak staging. All retinal biomarkers correlated with the cognitive scores, with retinal Aβ_42_, far-peripheral AβOi and microgliosis displaying the strongest correlations. Proteomic analysis of AD retinas revealed activation of specific inflammatory and neurodegenerative processes and inhibition of oxidative phosphorylation/mitochondrial, and photoreceptor-related pathways. This study identifies and maps retinopathy in MCI and AD patients, demonstrating the quantitative relationship with brain pathology and cognition, and may lead to reliable retinal biomarkers for noninvasive retinal screening and monitoring of AD.

**Supplementary Information:**

The online version contains supplementary material available at 10.1007/s00401-023-02548-2.

## Introduction

The pathological processes of Alzheimer’s disease (AD), a devastating neurodegenerative disorder and a major cause of morbidity and mortality worldwide [1], are not only confined to the brain but also manifest in the neurosensory retina [40, 77, 100, 105]. The hallmark signs of AD, cerebral amyloid β-protein (Aβ) plaques and neurofibrillary tangles (NFTs) comprised of hyperphosphorylated (p)Tau, are prerequisite for a definitive AD diagnosis and have been shown to precede clinical dementia onset by decades [48, 107]. Disease detection during the earlier stages of AD, when neuronal damage is limited, should allow early intervention and increased therapeutic efficacy. With current limitations on early diagnosis and clinical monitoring [52], the retina, a developmental extension of the brain unshielded by bone [20, 30, 85, 87], offers unparalleled accessibility for direct, affordable, and noninvasive visualization and temporal monitoring of central nervous system (CNS) targets at vascular, cellular, and molecular resolutions [9, 63, 118]. Exploring the manifestations of AD in the retina and its relationship to brain pathology is thus a priority.

Early studies described retinal ganglion cell (RGC) degeneration and identified AD-specific pathology, including Aβ deposits, pTau, and NFTs, in the postmortem retinas of AD patients [11, 12, 57, 59, 95]. Subsequent studies have demonstrated that, similar to AD brains, the retinas of AD patients exhibit accumulation of Aβ peptides and plaques, vascular Aβ_40_ and Aβ_42_ deposits, pTau inclusions, gliosis, and pericyte and neuronal degeneration [4, 5, 12, 18, 22, 24, 34, 39, 57, 59, 62, 65, 88, 95, 96, 101, 119]. Notably, these disease signs are often detected in the superior temporal (ST) and inferior temporal (IT) peripheral retinal regions. In our earlier study, we found a 4.7-fold increase in Aβ_42_ plaques in ST flat mount retinas from eight AD patients compared to seven individuals with normal cognition (NC), with a positive correlation between the burden of retinal and brain plaques in eight human subjects [57]. Beyond plaques, soluble Aβ oligomers (AβOs) isolated from AD brains exert high synaptic and neuronal toxicity and can drive cognitive impairment [41, 68, 98, 99]. While AβOs were demonstrated in retinas of rodent models of AD [36, 37], their manifestation in human AD retinas and the potential relevance to disease status have never been explored. Further, in the brains of AD patients, Aβ plaques are typically associated with surrounding inflammatory cells, including activated microglia and reactive astrocytes [69, 73]. In murine AD model brains, microglia were also found to exhibit reduced capacity for Aβ clearance [15, 43, 58, 60]. This glial cell dysfunction may contribute to a vicious cycle of neurotoxicity, cellular senescence, and synaptic and neuronal loss, leading to cognitive decline [2, 10, 42]. Although a few studies have provided evidence of enhanced glial responses in retinal tissues from AD patients [12, 34, 119], our knowledge of retinal gliosis in AD is very limited and it remains unclear whether similar interrelations among brain gliosis and neurodegeneration and cognition also exist in the AD retina.

Notably, noninvasive retinal optical imaging has been used to detect amyloid plaques, atrophy, and vascular damage in living AD patients [16, 19, 23, 53, 57, 61, 77, 100]. Pilot studies have found that the retinal amyloid load is significantly greater in patients with preclinical AD, mild cognitive impairment (MCI), or AD dementia than in individuals with normal cognition [27, 28, 38, 57, 66, 80, 83, 108]. Additionally, the degree of retinal amyloid burden has been found to correlate with cerebral amyloid load, as measured by positron emission tomography (PET), as well as hippocampal and whole gray matter atrophy and verbal memory deficits [27, 28, 83, 108].

While advances in retinal imaging and identification of the neuroretina as a site of AD pathology have been made, there are still gaps in our understanding of the pathophysiological processes in the retina and the potential link between retinal and brain pathology that could be used to predict disease status. We hypothesized that the principal AD processes, such as Aβ accumulation and associated gliosis and neurodegeneration, occur in the retina at the onset of functional impairment (MCI), particularly in peripheral subregions, and worsen as the disease progresses to AD dementia. We anticipate that the degree of retinal pathology is indicative of the severity of brain pathology and cognitive decline. To gain deeper insight into AD processes in the retina, and given the pivotal role of brain Aβ and its link to inflammation and neurodegeneration in disease pathogenesis [2, 10, 42, 117], we conducted an exploratory histological and biochemical investigation of these pathological changes in retinal tissues from patient donors with definitive AD dementia and those with MCI (due to AD) compared to NC individuals (*n* = 54). We identified intraneuronal AβO (AβOi) species and determined the burden and spatiotemporal distribution of Aβ_42_ and AβOi species, glial fibrillary acidic protein (GFAP)^+^ and S100 calcium-binding protein B (S100β)^+^ macrogliosis, ionized calcium-binding adaptor molecule 1 (IBA1)^+^ microgliosis, and tissue atrophy in predefined subregions of retinal cross-sections. Moreover, to explore the connection between the retina and brain, we evaluated correlations between these retinal pathologies, the severity of paired brain pathology (e.g., Aβ plaques, NFTs, neuropil threads [NTs], atrophy, and Braak stage), and cognitive impairment. Importantly, we investigated whether these retinal abnormalities are present in the earliest stages of functional impairment, namely in the retinas of MCI patients. To further elucidate our findings, we explored global proteomic profiles in retinal and brain tissues from AD patients compared to NC controls (*n* = 32). Our data revealed early and substantial pathological changes specific to AD in the retina, which were particularly evident in certain geometric regions and closely associated with brain pathology and cognitive status.

## Materials and methods

### Postmortem eyes and brains from human donors

Human eye and brain tissues collected from deceased donor patients with premortem clinical diagnoses of MCI and AD dementia (and confirmed postmortem AD neuropathology), and age- and sex-matched deceased NC controls (total *n* = 86 subjects) were primarily obtained from the Alzheimer’s Disease Research Center (ADRC) Neuropathology Core in the Department of Pathology (IRB protocol HS-042071) of Keck School of Medicine at the University of Southern California (USC, Los Angeles, CA). Additional eyes were obtained from the National Disease Research Interchange (NDRI, Philadelphia, PA) under approved Cedars-Sinai Medical Center IRB protocol Pro00019393. Subjects with macular degeneration, diabetic retinopathy, and glaucoma were excluded. For a subset of patients and controls, we also obtained brain specimens from the ADRC Neuropathology Core at the University of California, Irvine (UCI [IRB protocol HS#2014–1526]). USC-ADRC, NDRI, and UCI ADRC maintain human tissue collection protocols that are approved by their managerial committees and subject to oversight by the National Institutes of Health.

**Fig. 1:**
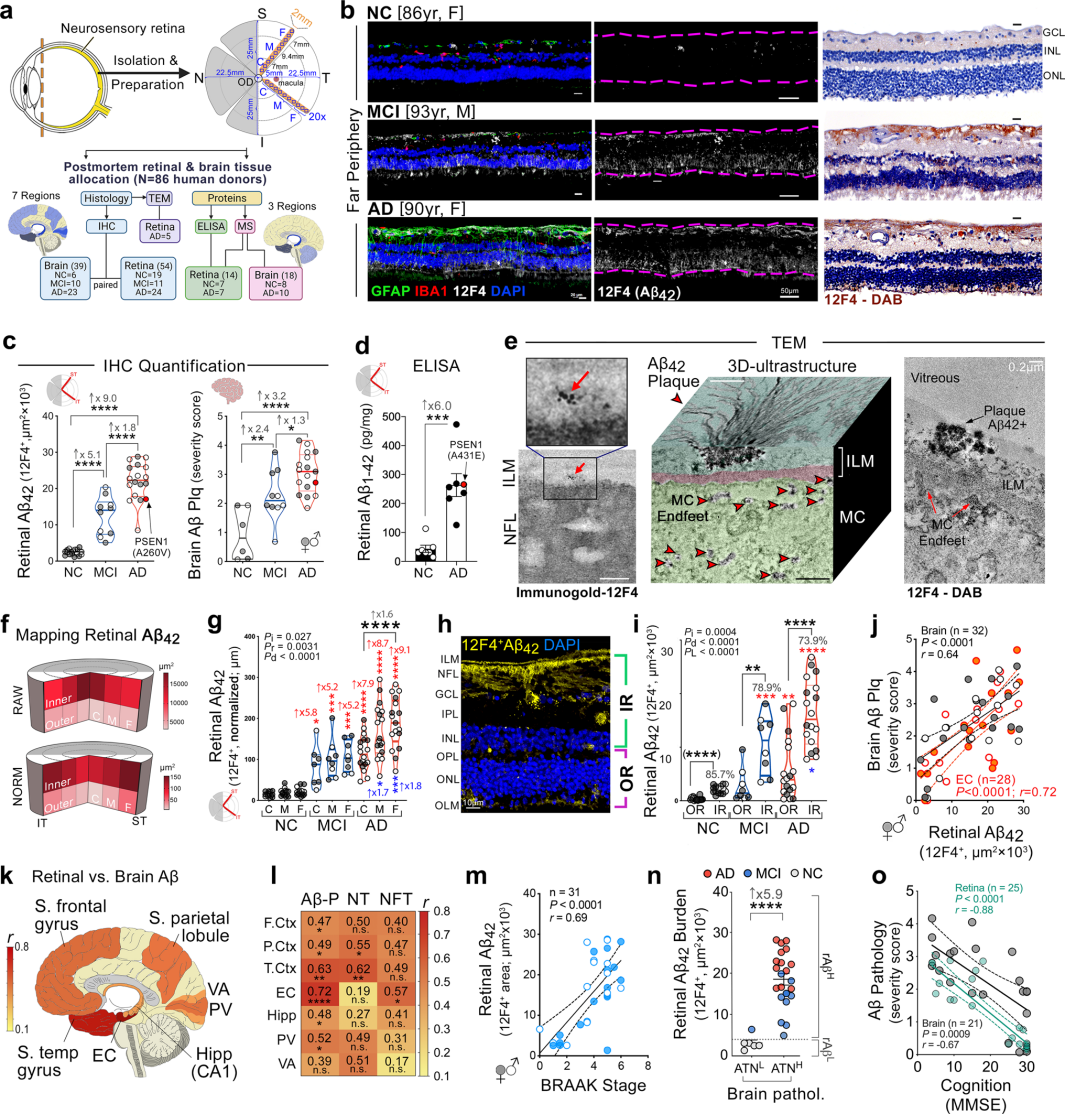
Spatiotemporal distribution of Aβ_42_ burden in retinas of MCI and AD patients and relations to brain pathology and cognition. **a** Illustration depicts analyzed retinal cross-sections in predefined geometrical regions including superior- and inferior temporal (ST/IT) strips (orange) extending from the optic disc (OD) to the ora serrata and separated into subregions: central (C), mid-periphery (M) and far periphery (F). Schematic flow-diagram describes human donor eyes and brains allocated for histological and protein analyses (N = subjects). **b** Fluorescence micrographs of retinal cross-sections from MCI and AD patients compared to normal cognition (NC) controls. Tissues were immunolabeled for GFAP^+^-macroglia (green), IBA1^+^-microglia (red), 12F4^+^-Aβ_42_ (white), and DAPI^+^-nuclei (blue; dashed lines indicate margins of analyzed layers between the inner and outer limiting membranes–ILM/OLM). Scale bar: 50 µm. Right micrographs are from the same individuals immunolabeled with 12F4^+^-Aβ_42_ using peroxidase-based 3,3′diaminobenzidine (DAB) and hematoxylin counterstaining. Scale bar: 20 µm. **c** Violin plots display quantitative-IHC analysis of retinal (r)Aβ_42_-immunoreactive area in age- and sex-matched patients with premortem clinical diagnoses of NC (*n* = 17), MCI (*n* = 10), or AD (*n* = 18), and paired-brain (b)Aβ-plaque severity scores in NC (*n* = 6), MCI (*n* = 10), and AD (*n* = 17) patients. Red circle represents an ADAD patient with an A260V mutation in presenilin-1 (*PSEN1*). **d** Retinal Aβ_1–42_ levels determined by ELISA are shown in an additional cohort of NC and AD patients (*n* = 14; ADAD patient with *PSEN1-*A431E mutation, red circle). **e** TEM-micrographs from AD patients’ retina: Left, 12F4^+^-immunogold Aβ_42_-positive black puncta signals at high-magnification (red arrow) in the ILM/innermost layers. Scale bar: 200 nm. Middle: 3D-reconstruction of vertical/en face TEM images show rAβ_42_ plaque ultrastructure with fibril arms emanating from its dense core and Aβ-containing deposits (red arrowheads). Scale bar: 1 μm. Right, Aβ_42_ plaque (black arrow) and deposits within Müller cell (MC) endfeet (red arrows). Scale bar: 0.2 µm. **f** Pie charts display Aβ_42_ distribution across the inner retina (IR), outer retina (OR), and C, M, and F subregions: raw data and normalized per retinal thickness (density); higher burden in darker red. **g** Violin plot displays rAβ_42_ density for C, M, and F subregions. **h** Definition of inner retina (IR) and outer retina (OR) in a cross-section. Scale bar: 10 μm. **i** Aβ_42_ burden in IR vs. OR; percentages indicate rAβ_42_ area in IR of total area. Statistics: red or blue asterisks mark significance relative to NC or MCI, respectively. *P*_d_–diagnostic groups; *P*_*r*_–C, M, vs. F subregions; *P*_*L*_–IR vs. OR layers; *P*_i_–interactions. **j** Scatterplot presents correlations between rAβ_42_ area and Aβ plaques in total brain (gray) or EC (orange). **k–l** Mid-sagittal brain illustration and heatmap show color-grading magnitude of Pearson’s correlation coefficient (*r*) values with multivariable Holm-Bonferroni adjusted *P-* values (asterisks) between rAβ_42_ burden and brain pathology: Aβ-(P)laques, neuropil threads (NT), and neurofibrillary tangles (NFT) in the hippocampus (Hipp), superior (S.) frontal (F. Ctx) and temporal (temp, T. Ctx) gyrus, S. parietal lobule (P. Ctx), entorhinal (EC), primary visual (PV), and visual association (VA) cortices. **m** Pearson’s correlation between rAβ_42_ burden and BRAAK stage. **n** Subjects were stratified based on high(H) or low(L) brain ATN-histopathology severity and plotted based on rAβ_42_ burden; extrapolated dotted-gray line marks rAβ_42_ level separating ATN^H^ from ATN^L^ individuals. **o** Pearson’s correlations between rAβ_42_ area or bAβ burden and the Mini-Mental State Examination (MMSE)-cognitive scores. Data points are presented with group means ± SEMs. Filled and empty circles represent women and men, respectively. Median and lower and upper quartiles are indicated on each violin plot. *P < 0.05, **P < 0.01, ***P < 0.001, ****P < 0.0001, by one-way or two-way ANOVA and Tukey’s post hoc multiple comparison test, or by two-tailed paired (parenthesis) or unpaired Student’s *t* test

Histological studies at Cedars-Sinai Medical Center were performed under IRB protocols Pro00053412 and Pro00019393. For histological examinations, 54 retinas were collected from deceased donors with confirmed AD (*n* = 24) or MCI due to AD (*n* = 11), and from age- and sex-matched deceased donors with NC (*n* = 19). In a subset of patients, paired brain tissues were also analyzed (*n* = 39). For the biochemical assays [enzyme-linked immunosorbent assay (ELISA), mass spectrometry (MS)] of retinal proteins, eyes were collected from another deceased donor cohort (*n* = 14) comprised of clinically and neuropathologically confirmed AD patients (*n* = 7) and matched NC controls (*n* = 7). Demographic, clinical, and neuropathological information on human donors is detailed in Table 1; more data on individual human donors is found in Suppl. Table S1, online resource. For mass spectrometry of brain proteins, fresh-frozen human brain tissue was obtained from an additional donor cohort (*n* = 18) of clinically and neuropathologically confirmed AD patients (*n* = 10) and matched NC controls (*n* = 8). Demographic, clinical, and neuropathological information on human donors is detailed in Table 2 and Suppl. Table S2, online resource. Tissue allocation to histological and biochemical analyses is depicted in Fig. 1a. Patients’ identities were protected by de-identifying all tissue samples in a manner not allowing tracing back to tissue donors.

**Table 1:**
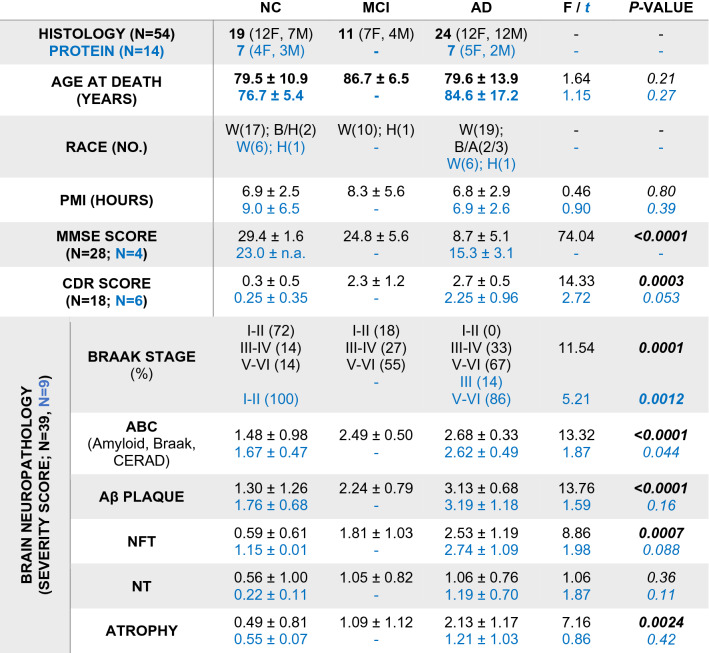
Demographic data on human donors whose postmortem retinas and brains were used for histological or protein analyses

Human donors (total *n* = 68 subjects) were included in histological analyses (*n* = 54) and protein analyses (*n* = 14)

Paired brains with full neuropathological assessments were available for 39 human donors in the histological cohort (*n* = 6 NC, *n* = 10 MCI, *n* = 23 AD) and 8 in the protein cohort (*n* = 2 NC, *n* = 6 AD)

Values are presented as mean ± SD. *F*, *t*, and *P* values were determined by one-way ANOVA with Tukey’s multiple comparisons test (histology cohort) or unpaired Student’s *t* test (protein cohort)

*NC* normal cognition, *MCI* mild cognitive impairment, *AD* Alzheimer’s disease, *F* female, *M* male, *SD* standard deviation, *W* White, *B* Black, *H* Hispanic, *A* Asian, *PMI* postmortem interval, *MMSE* Mini-Mental State Examination, *CDR* Clinical dementia rating, *n.a.* not available, *Aβ* Amyloid-β protein, *NFT* neurofibrillary tangles, *NT* neuropil threads

Mean ABC scores determined as: *A* Aβ plaque score modified from Thal, *B* NFT stage modified from Braak, *C* Neuritic plaque score modified from CERAD

*P* values presented in bold type demonstrate significance < 0.05

**Table 2:** Demographic data on human donors whose postmortem brains were used for mass spectrometry analysis

Human donors	NC	AD	*t*	*P*
*n* = 18	**8** (5F, 3 M)	**10** (7F, 3 M)	–	–
Age at death [years]^§^	**91.3** ± 3.6	**90.0** ± 4.8	0.61	0.55
Race [no]	W (7)	W (9)	–	–
	H/A (1)	H (1)		
MMSE score^§^	27.0 ± 4.1	14.6 ± 2.9	6.74	** < 0.0001**
PMI [hours]^§^	3.7 ± 0.8	5.3 ± 3.2	1.43	0.17
Brain neuropathology [*n* = 18]				
AΒ-plaque stage (no; %)	None (6; 75%)	Stage C (10; 100%)	–	–
	Stage A (1; 12.5%)			
	Stage B (1; 12.5%)			
BRAAK stage (no; %)	I–II (6; 75%)	V–VI (10; 100%)	–	–
	III–IV (2; 25%)			
	V–VI (0)			

*NC* normal cognition, *AD* Alzheimer’s disease, *F* female, *M* male, *SD* standard deviation, *A* American Indian, *W* White, *H* Hispanic, *MMSE* Mini-Mental State Examination, *Aβ* Amyloid-β protein, *NFT* neurofibrillary tangles, *PMI* postmortem interval, *Un* unknown

Braak (NFT) stage scores; Aβ-Plaque stages score: None, no Aβ plaque or amyloid plaque; A, mild, A1 Thal phases 1 or 2; B, moderate, A2, Thal phase 3; C, severe plaque pathology, A3 Thal phases 4 or 5; Values are presented as mean ± SD

The *t* and *P* values were determined by Student’s *t* test. *P* values presented in bold type demonstrate statistical significance < 0.05

^§^Mean ± SD

### Clinical and neuropathological assessments

ADRC provided clinical and neuropathological reports on the patients’ neurological examinations, neuropsychological and cognitive tests, family history, and medication lists as collected in the ADRC system using the Unified Data Set (UDS) [9]. The NDRI provided reports on additional patients, including sex, race, age at death, cause of death, medical history indicating AD, presence or absence of dementia, and co-morbidities. Most cognitive evaluations had been performed annually and, in most cases, less than 1 year prior to death. Cognitive testing scores from evaluations made closest to the patient’s death were used for this analysis. Two global indicators of cognitive status were used for clinical assessment: the Clinical Dementia Rating (CDR scores: 0 = normal; 0.5 = very mild impairment; 1 = mild dementia; 2 = moderate dementia; or 3 = severe dementia) [81] and the Mini-Mental State Examination (MMSE scores: normal cognition = 24–30; MCI = 20–23; moderate dementia = 10–19; or severe dementia ≤ 9) [31].

In this study, the composition of the clinical diagnostic group (AD, MCI, or NC) was determined by source clinicians based on a comprehensive battery of tests including neurological examinations, neuropsychological evaluations, and the aforementioned cognitive tests. To obtain a final diagnosis based on the neuropathological reports, we used the modified Consortium to Establish a Registry for Alzheimer's Disease (CERAD) [76, 92], as outlined in the National Institute on Aging (NIA)/Regan protocols with revision by the NIA and Alzheimer’s Association [47]. The Aβ plaque burden, including diffuse and neuritic plaques (immature and mature), amyloid angiopathy, NFTs, NTs, granulovacuolar degeneration, Lewy bodies, Hirano bodies, Pick bodies, balloon cells, neuronal loss, microvascular changes, and gliosis pathology were assessed in pertinent brain areas, specifically in the hippocampus (particularly the Cornu ammonis CA1, at the level of the thalamic lateral geniculate body), entorhinal cortex (EC), superior frontal gyrus of the frontal lobe, superior temporal gyrus of the temporal lobe, superior parietal lobule of the parietal lobe, primary visual cortex (PV, Brodmann Area-17), and visual association (VA, Area-18) of the occipital lobe. In all cases, uniform brain sampling was done by a neuropathologist.

Cerebral amyloid plaques, NFTs, and NTs were evaluated using anti-β-amyloid mAb clone 4G8 immunostaining, Thioflavin-S (ThioS) histochemical stain, and Gallyas silver stain in formalin-fixed, paraffin-embedded tissue sections. Neuropathologists (Chief, C.A.M. and Dr. Debra Hawes) provided severity scores based on semi-quantitative observations. The scale for Aβ/neuritic plaques was determined by 4G8- and/or Thioflavin-S-positive and/or Gallyas silver-positive plaques measured per 1 mm^2^ brain area (0 = none; 1 = sparse [≤ 5 plaques]; 3 = moderate [6–20 plaques]; 5 = abundant/frequent [21–30 plaques or greater]; or N/A = not applicable), as previously described [79]; NACC NP Guidebook, Version 10, January 2014: https://naccdata.org/data-collection/forms-documentation/np-10]. Brain NFT or NT severity scoring system was derived from observed burden of these AD neuropathologic changes detected by Gallyas silver and/or Thioflavin-S staining [78, 79, 111] and measured per 1 mm^2^ brain area. The assigned NFT or NT scores are as following: 0 = none; 1 = sparse (mild burden); 3 = moderate (intermediate burden); or 5 = frequent (severe burden); this scoring system is illustrated in representative microscopic images in Suppl. Fig. 1a, b, online resource. In both histochemical and immunohistochemical staining, each anatomic area of interest is assessed for the relevant pathology using the 20 × objective (200 × high power magnification) and representative fields are graded using a semiquantitative scale as detailed above. Validation of AD neuropathic change (ADNP), especially NTs, is performed using the 40 × objectives (400 × high power magnification); an average of 2 readings was assigned to each individual patient.

A final diagnosis included AD neuropathological change using an “ABC” score derived from three separate four-point scales. We used the modified Aβ plaque Thal score (A0 = no Aβ or amyloid plaques; A1 = Thal phase 1 or 2; A2 = Thal phase 3; or A3 = Thal phase 4 or 5) [109]. For NFT staging, we used modified Braak staging for silver-based histochemistry or p-tau immunohistochemistry (IHC) (B0 = no NFTs; B1 = Braak stage I or II; B2 = Braak stage III or IV; or B3 = Braak stage V or VI) [13]. For neuritic plaques, we used the modified CERAD score (C0 = no neuritic plaques; C1 = CERAD score sparse; C2 = CERAD score moderate; or C3 = CERAD score frequent) [76].

Neuronal loss, gliosis, granulovacuolar degeneration, Hirano bodies, Lewy bodies, Pick bodies, and balloon cells were all evaluated (0 = absent or 1 = present) in multiple brain areas by staining tissues with hematoxylin and eosin (H&E). Brain atrophy was evaluated (0 = none; 1 = mild; 3 = moderate; 5 = severe; or 9 = not applicable). We classified brain amyloid, tauopathy, and neurodegeneration (ATN) histopathological scores based on high (H) and low (L) severity of brain Aβ deposition (A; cutoff score = 2.0), tauopathy (T; cutoff score = 1.1), and neurodegeneration (N; cutoff score = 0.4), which were modified from the latest NIA-AA’s in vivo A/T/N biomarker classification scheme for AD [49]. Our ATN^High^ and ATN^low^ designations were determined according to a combined cutoff score of 3.5; the terms were used to refer to humans with high or low histopathology levels of brain amyloid, tauopathy, and neuronal loss, respectively.

### Processing of eye and brain tissues

Donor eyes were collected within an average of 7.5 h after time of death and were (1) preserved in Optisol-GS media (Bausch & Lomb, 50,006-OPT) and stored at 4 °C for less than 24 h; (2) fresh-frozen (snap frozen; stored at − 80 °C); or (3) punctured once and fixed in 10% neutral buffered formalin (NBF) or 4% paraformaldehyde (PFA) and stored at 4 °C. In addition, fresh brain tissues [hippocampus, occipital lobe–PV, and frontal cortex (area 9)] from the same donors were snap frozen and stored at − 80 °C. Portions of fresh-frozen brain tissues were fixed in 4% PFA for 16 h following dehydration in 30% sucrose in phosphate-buffered saline (PBS). Brain tissues were sectioned (30 μm thick) on a cryostat and placed in PBS with 0.01% sodium azide (Sigma-Aldrich) at 4 °C. Regardless of the source of the human donor eye (USC-ADRC or NDRI), the same tissue collection and processing methods were applied.

### Preparation of retinal strips

Eyes were fixed and processed as previously described [101]. Briefly, after careful dissection and thorough cleaning of the vitreous humor, flat mount strips (~ 2 mm wide) extending diagonally from the optic disc (OD) to the ora serrata (~ 20–25 mm long) were prepared to create four strips (ST, IT, inferior-nasal, and superior-nasal; Fig. 1a). Retinal strips from the fresh-frozen retinas were either fixed in 4% PFA for cross-sectioning or stored at − 80 °C for protein analysis. The fixed flat mount strips were further processed and embedded in paraffin and cryosectioned to 7-µm retinal cross-sections, which were mounted on 3-aminopropyltriethoxysilane (APES, Sigma A3648)-coated slides, as previously described [101]. Before the IHC procedure, slides with paraffin-embedded cross-sections were deparaffinized with 100% xylene twice (10 min each), rehydrated with decreasing concentrations of ethanol (100% to 70%), and washed with distilled water followed by PBS. This sample preparation technique allowed for extensive and consistent access to retinal quadrants, layers, and pathological subregions.

### Immunohistochemistry

Brain sections and deparaffinized retinal cross-sections were treated with target retrieval solution (pH 6.1; S1699, DAKO) at 99 °C for 1 h, washed with PBS, and then treated in formic acid 70% (ACROS) for 10 min at room temperature (RT) before staining for Aβ burden. Peroxidase-based and fluorescence-based immunostaining were performed. A list of antibodies and their working dilutions are shown in Suppl. Table S3, online resource. Before peroxidase-based immunostaining, tissues were treated with 3% H_2_O_2_ for 12 min, and two peroxidase-based staining protocols were followed. We first used a Vectastain Elite ABC HRP kit (Vector, PK-6102, Peroxidase Mouse IgG) according to the manufacturer’s instructions and second a Dako reagents protocol. Following treatment with formic acid, the tissues were washed with wash buffer (Dako S3006), adding 0.2% Triton X-100 (Sigma, T8787) for 1 h, and then treated with H_2_O_2_ and rinsed with wash buffer. Primary antibody (Ab) was diluted with background-reducing components (Dako S3022) and incubated with the tissues for 1 h at 37 °C for JRF/cAβ42/26 #8151 (Aβ42) or JRF/Aβtot/17 Pur 117–120 (N-terminal region of Aβ) Abs, or overnight at 4 °C for 12F4 (Aβ42) mAb. Tissues were rinsed twice with wash buffer on a shaker, incubated for 30 min at 37 °C with secondary Ab (goat anti-mouse Ab, HRP conjugated, DAKO Envision K4000), and rinsed again with wash buffer. For both protocols, 3,3′-diaminobenzidine (DAB) substrate was used (DAKO K3468). Hematoxylin counterstaining was performed followed by mounting with Paramount aqueous mounting medium (Dako, S3025). To ensure that the component detected in the tissue was not derived from exogenous normal serum, the blocking step was omitted for a subset of retinal tissues. Routine controls were processed using identical protocols while omitting the primary Ab to assess nonspecific labeling.

For fluorescence-based immunostaining, sections were treated with blocking solution (DAKO X0909) supplemented with 0.2% Triton X-100 (Sigma, T8787) prior to overnight incubation with primary Abs (Suppl. Table S3, online resource) at 4 °C. Secondary Abs were added the following day and incubated for 1.5 h at RT. Specifically, for AβOi staining, scFvA13 was used as the primary Ab [74, 75, 97], which required intermediate anti-V5 tag Ab followed by a secondary Ab. The scFvA13 is a conformation-sensitive and sequence-specific Ab in the format of a single-chain Fv fragment (scFv), which selectively recognizes AD-relevant AβOs. To eliminate background autofluorescence, we treated the sections with 0.3% (w/v) Sudan Black B (199,664, Sigma-Aldrich) in 70% ethanol (v/v) for 10 min at RT. Next, we mounted the samples using ProLong Gold Antifade Mountant with DAPI (Thermo Fisher; #P36935). Routine controls were processed using identical protocols, omitting the primary Ab to assess nonspecific labeling.

### ***Biochemical determination of Aβ***_***1–42***_*** levels by sandwich ELISA***

Fresh-frozen human retinal strips from the temporal hemisphere (ST, IT) were weighed and placed in a tube (1 mg tissue/10 µl buffer) with cold homogenizing buffer [100 mM TEA Bromide (Sigma, 241,059), 1% sodium deoxycholate (SDC; Sigma, D6750), and 1 × Protease Inhibitor cocktail set I (Calbiochem, 539,131)]. Retinal homogenates were sonicated (Qsonica sonicator with an M-Tip probe, amplitude 4, 6 W, for 90 s, with the sonication pulse stopped every 15 s to allow the cell suspension to cool down for 10 s), while the ultrasonic probe was positioned inside the sample tube that was placed in ice water. After determination of protein concentrations (Thermo Fisher Scientific), the amount of retinal Aβ_1–42_ was determined using an anti-human Aβ_1–42_ end-specific sandwich ELISA kit (Thermo Fisher, KHB3441).

### Brain Aβ burden

ADRC neuropathological reports provided comprehensive data on cerebral Aβ plaques in different brain areas. Brain Aβ plaque severity scores were calculated from neuropathological reports based on the Aβ burden assigned to each patient, as described earlier. Brain Aβ plaque severity for each patient was calculated based on grades that were obtained after staining with Gallyas silver stain and anti-β-amyloid mAb 4G8 or ThioS. Averages of burden scores were calculated for each brain area or region separately, for total brain regions, and for neuritic plaques, NFTs, and NTs.

### Mapping retinal pathology

Retinal Aβ_42_ burden, AβOi, macrogliosis, microgliosis, and retinal atrophy were determined by examining four radial cross-sections (for each experiment) from the temporal hemisphere (strips from the ST and IT regions). Following specific staining, ten images were obtained covering 4500 µm (4.5 mm) of linear retinal tissue from each strip and all retinal layers. Four sequential cross-sections were analyzed, totaling 4 × 4500 = 18,000 linear microns or 18 mm in ST and IT retinal length. The single snap images were evenly spaced across the strip, captured at 20 × objective, representing the neuroretina from the optic disc to the ora serrata, including central (three images), mid- (four images), and far- (three images) peripheral retinal subregions (C, M, F as defined above). The average ± SD measured length of each processed paraffin-embedded strip was 23.45 ± 0.2 mm. Retinal subregions were defined based on their radial distance from the center of the OD: C [~ 7 mm length (from OD)], M [~ 9.4 mm length (from ~ 7 mm to 16.5 mm)], and F [~ 7 mm length (from ~ 16.5 mm to the strip end, ~ 23.45 mm)].

To convert the histological C, M, and F anatomical subregions to visual field locations in live retinal imaging, we assessed the degree angle (α) based on the following: an average 4.76 mm distance between the OD center and the fovea in fundus images [50], which was roughly 5 mm in our flat mount retinas, and an estimated 15 degrees α distance between the OD center and the fovea in live retinal imaging [91]. Hence, we determined that live imaging centered on the fovea at 30 degrees α will cover the central (C) subregions, including the macula, imaging at 60 degrees α will cover the M subregions [27, 28], and imaging at > 60 degrees α will cover parts of the F peripheral subregions.

To assess retinal layer distribution, retinal pathology burden was analyzed in the inner and outer retina separately. The inner retina was anatomically defined as extending from the inner limiting membrane (ILM) to and including the inner nuclear layer (INL). The outer retina was analyzed from the outer plexiform layer (OPL) to the outer limiting membrane (OLM). Given that retinal thickness differs greatly throughout the C, M, and F subregions—thickest near the optic nerve head and thinnest toward the ora serrata in the F subregion (~ 260–300 µm and ~ 80–120 µm in elderly healthy persons, respectively)—we also normalized immunoreactive (IR) area to retinal thickness; the width was measured at three defined tissue areas per each image. Images were captured at 20 × or 40 × objective at a respective resolution of 0.5 or 0.25 µm. Images were exported to ImageJ (version 1.52o; NIH) to calculate the total area of Aβ_42_ burden, AβOi, gliosis, and microgliosis.

### Tissue-atrophy morphometric analysis

For morphometric analysis, ten images were obtained (20 × ; three images from the far periphery, four from the mid-periphery, and three from the central area of the retina). Thickness measurements (µm) were manually performed using Axiovision Rel. 4.8 software. Retinal thickness assessments were taken from the ILM through the OLM (illustrated in Fig. 4a), across the following retinal layers: nerve fiber layer (NFL), ganglion cell layer (GCL), inner plexiform layer (IPL), inner nuclear layer (INL), outer plexiform layer (OPL), and outer nuclear layer (ONL).

### Severity score analysis of retinal atrophy

To assess the magnitude of retinal tissue loss in disease, we devised a scoring system for retinal atrophy that is similar to severity scores assigned to brain atrophy in postmortem neuropathological reports. For this purpose, cross-sectional thicknesses of the C, M, and F subregions were used to calculate the mean retinal thickness for each patient. Retinal atrophy scores were subsequently assigned according to a severity range of 0–5, separated by 0.5 intervals, with 0 assigned to the thickest and most intact retina (155 μm) and 5 to the thinnest and most atrophied retina (110 μm).

### Microscopy

Fluorescence and bright field images were acquired using a Carl Zeiss Axio Imager Z1 fluorescence microscope with ZEN 2.6 blue edition software (Carl Zeiss MicroImaging, Inc.) equipped with ApoTome, AxioCam MRm, and AxioCam HRc cameras. Multi-channel image acquisition was used to create images with multiple channels. Tiling mode and post-acquisition stitching were used to capture and analyze large areas. Images were repeatedly captured at the same focal planes with the same exposure time. Images were captured at 20 × , 40 × , 63 × , and 100 × objectives for different purposes.

### Quantitative immunohistochemistry

Images were captured using the same exposure time and settings. We randomly acquired three images from the F, four from the M, and three from the C subregions for analytical purposes (as shown in Fig. 1a). Images were exported to ImageJ (version 1.52o; NIH) to analyze parameters of interest. Throughout the analysis process, the researchers were blinded to each patient’s diagnosis. The fluorescence of specific signals was captured, using the same setting and exposure time for each image and human donor, with an Axio Imager Z1 microscope (with motorized Z-drive) and an AxioCam MRm monochrome camera ver. 3.0 (at a resolution of 1388 × 1040 pixels, 6.45 µm × 6.45 µm pixel size, and dynamic range of > 1:2200, which delivers low-noise images due to a Peltier-cooled sensor). Images were captured at either 20 × or 40 × objective at a respective resolution of 0.5 or 0.25 µm. Acquired images were converted to grayscale and standardized to baseline by using a histogram-based threshold in ImageJ. The images were then submitted to ImageJ particle analysis for each biomarker to determine total IR area. For each biomarker, the IR area was determined using the same threshold percentage from the baseline in ImageJ with the same percentage threshold setting for all diagnostic groups.

To quantify co-localized 12F4^+^Aβ_42_-immunoreactive puncta in IBA1^+^ microglia, images were compiled and analyzed using Adobe Photoshop (Adobe Systems Inc., Mountain View, CA, USA). The puncta count of 12F4^+^ foci was obtained by tracing an outline around each IBA^+^ cell. Co-localization of 12F4^+^ (green) with IBA^+^ (red) was quantified by measuring the fluorescence of coincident (yellow) particles. The frequency of coincidence of 12F4^+^Aβ_42_ area co-localizing within IBA^+^ microglia in retinal cross-sections was plotted as the percentage of total IBA1^+^ area.

### Transmission electron microscopy (TEM) analysis

Retinal flat mounts were fixed and peroxidase-based immunostained with anti-human Aβ_42_ mAb (12F4), as described above. Retinal tissues were then prepared and microdissected for electron microscopic analysis. The samples were dehydrated in a graded series of ethanol and then infiltrated in Eponate 12 resin (Ted Pella, Inc. Redding, CA, USA) prior to embedding between 2 acetate sheets. Ultrathin sections of retina were cut (vertical and en face) at a thickness of 70 nm, collected on copper grids, and lightly contrasted using Reynolds’ lead stain. The sections on grids were analyzed using a JEOL JEM-2100 LaB6 TEM at 80 kV (JEOL USA). Images were captured using the Orius SC1000B CCD camera (Gatan) and processed and colorized using Adobe Photoshop CS4 (Adobe Inc.).

### Immunogold labeling transmission electron microscopy

Retinal paraffin blocks were sectioned at 7 µm thickness on a microtome and floated in a 40 °C distilled water bath. The sections were transferred onto a Superfrost Plus slide, allowed to dry overnight, and stored at RT until use. Prior to staining, the slides were baked at 55 °C and deparaffinized with xylene and alcohol. The slides were placed into a Coplin jar containing citrate antigen retrieval buffer and heated in a microwave until boiling. Then, they were allowed to cool down to RT, rinsed in PBS, and incubated in blocking solution [2% bovine serum albumin (BSA) in PBS] for 30 min. Next, the sections were incubated in a moist chamber overnight at 4 °C with primary mouse anti-human Aβ_42_ mAb (12F4) that was diluted in blocking solution (2% BSA in PBS). After rinsing with PBS, the sections were incubated with goat anti-mouse 10-nm gold conjugate secondary Ab (Ted Pella, Inc.) for 1 h at 37 °C before being rinsed in PBS again. Samples were fixed in ½ Karnovsky’s fix for 15 min, rinsed in 0.1 M cacodylate buffer pH 7.2, postfixed with 2% osmium for 20 min, rinsed again in cacodylate buffer pH 7.2, and finally rinsed in sodium acetate. The slides were then en bloc stained with 1% uranyl acetate for 1 h and rinsed in sodium acetate. Samples were passed through a dehydration process involving 50% EtOH; 70%, 85%, 95%, and 100% EtOH; 1:1 EtOH/propylene oxide (PO) mix; 1:2 EtOH/PO mix; and pure PO; followed by PO/Eponate resin and 100% Epon. Following this, the samples were embedded with Eponate using a beam capsule filled with Eponate and inverted onto the section. The beam capsules were removed from the slides using liquid nitrogen, sectioned ultra-thin at 70 nm, and placed onto grids. The sections were analyzed in a JEOL JEM-2100 LaB6 TEM at 80 kV (JEOL USA). Images were captured using an Orius SC1000B CCD camera (Gatan).

### Proteome analysis by mass spectrometry (MS)

#### Preparation of retinal and brain samples from NC and AD donors

Frozen brains (from the neuropathology core of the ADRC at the University of California, Irvine) and retinas (from the ADRC Neuropathology Core in the Department of Pathology at the University of Southern California, Los Angeles, CA) were processed for MS by the University of Queensland in accordance with approval granted by the institution’s Human Research Ethics Committee (#2017000490). Frozen brain aliquots from the hippocampus, medial temporal gyrus, and cerebellum were used for the brain analysis study. Frozen tissues were transferred into a Precellys homogenization tube (Bertin Technologies), homogenized in liquid nitrogen, and lysed in ice-cold T-PER extraction buffer (Thermo Scientific) containing protease and phosphatase inhibitors. Next, tissue lysates were cleared of any debris by ultracentrifugation at 100,000 g for 60 min at 4 °C. Retinal temporal hemisphere (ST, IT) tissues were homogenized (100 mM TEA Bromide [Sigma, 241059], 1% SDC [Sigma, D6750], and 1 × protease inhibitor cocktail set I [Calbiochem 539131]) by sonication [Qsonica sonicator with M-Tip probe, amplitude 4, 6 W, for 90 s, with the sonication pulse stopped every 15 s to allow the cell suspension to cool down for 10 s). Insoluble materials were removed by centrifugation at 15,000* g* for 10 min at 4 °C. Protein concentrations of brain and retinal lysates were determined via the Bradford assay (Bio-Rad Laboratories). Extracted brain and retinal proteins were reduced using 5-mM DTT alkylation with 10-mM iodoacetamide. Protein concentration was determined using a BCA assay kit (Pierce). Dual digestion was carried out on 150 µg protein, initially using Lys-C (Wako, Japan) at a 1:100 enzyme:protein ratio overnight at RT followed by trypsin (Promega) at a 1:100 enzyme:protein ratio overnight at 37 °C.

#### Tandem mass tag (TMT) labeling

To accommodate 14 retinal samples (7 AD and 7 NC) and 54 brain samples (10 AD and 8 NC, for each of the 3 corresponding tissue: hippocampus, cerebellum, and frontal cortex), 8 separate TMT10plex experiments were performed. As described previously [102], 50 μg peptides from each sample were labeled with 0.8 mg TMT reagent. Labeling for 1 h occurred at RT with continuous vortexing. To quench any remaining TMT reagent and reverse the tyrosine labelling, 8 μl 5% hydroxylamine was added to each tube, followed by vortexing and incubation for 15 min at RT. Combined samples from each TMT experiment were subjected to high-pH fractionation using an Agilent 1260 HPLC system equipped with a quaternary pump, a degasser, and a multi-wavelength detector (set at 210-, 214-, and 280-nm wavelengths). Peptides were separated on a 55-min linear gradient from 3 to 30% acetonitrile in 5 mM ammonia solution (pH 10.5) at a flow rate of 0.3 ml/minute on an Agilent 300 Extend C18 column (3.5-μm particles, 2.1-mm inner diameter, 150-mm length). The 96 fractions were finally consolidated into 17 fractions. Each peptide fraction was dried by vacuum centrifugation, resuspended in 1% formic acid, and desalted again using SDB-RPS (3M Empore) stage tips.

#### Nanoflow liquid chromatography electrospray ionization tandem mass spectrometry (nano LC–ESI–MS/MS)

Cleaned peptides from each fraction were analyzed using a Q Exactive Orbitrap mass spectrometer (MS; Thermo Scientific) coupled to an EASY-nLC1000 nanoflow HPLC system (Thermo Scientific). Reversed-phase chromatographic separation was performed on an in-house packed reverse-phase column (75 μm × 10 cm Halo 2.7-μm 160 Å ES-C18, Advanced Materials Technology). Labeled peptides were separated for 2 h using a gradient of 1%–30% solvent B (99.9% acetonitrile/0.1% formic acid) and Solvent A (97.9% water/2% acetonitrile/0.1% formic acid). The Q Exactive MS was operated in the data-dependent acquisition mode to automatically switch between full MS and MS/MS acquisition. Following the full MS scan from m/z 350–1850, MS/MS spectra were acquired at a resolution of 70,000 at m/z 400 and an automatic gain control target value of 10^6^ ions. The top ten most abundant ions were selected with a precursor isolation width of 0.7 m/z for higher energy collisional dissociation (HCD) fragmentation. HCD-normalized collision energy was set to 35%, and fragmentation ions were detected in the Orbitrap at a resolution of 70,000. Target ions that had been selected for MS/MS were dynamically excluded for 90 s. After quality control of protein homogenates for MS analysis, six AD and six NC retinas and ten AD and eight NC brains were included for further analyses.

#### Database searching, peptide quantification, and statistical analysis

Raw data files were processed using Proteome Discoverer V2.1 software (Thermo Scientific) and Mascot (Matrix Science, UK). Data were matched against the reviewed SwissProt *Homo sapiens* protein database. The MS1 tolerance was set to ± 10 ppm and the MS/MS tolerance to 0.02 Da. Carbamidomethyl (C) was set as a static modification, while TMT10-plex (N-term, K), oxidation (M), deamidation (N, Q), Glu- > pyro-Glu (N-term E), Gln- > pyro-Glu (N-term Q), and acetylation (Protein N-Terminus) were set as dynamic modifications. The percolator algorithm was used to discriminate correct from incorrect peptide-spectrum matches and to calculate statistics including *q* value (FDR) and posterior error probabilities. Search results were further filtered to retain protein with an FDR of < 1%, and only master proteins assigned via the protein grouping algorithm were retained. Proteins were further analyzed using the TMTPrepPro analysis pipeline. TMTPrepPro scripts are implemented in the R programming language and are available as an R package, which was accessed through a graphic user interface provided by a local Gene Pattern server. In pairwise comparison tests, the relative quantitation of protein abundance was derived from the ratio of the TMT label S/N detected in each condition (AD vs*.* NC), and differentially expressed proteins (DEPs) were identified based on Student’s t tests between AD and NC group ratios (log-transformed). The overall fold changes were calculated as geometric means of the respective ratios. Differential expression required the proteins to meet both a ratio fold change (> 1.2 for upregulated or < 0.80 for downregulated expression) and a *P* value cutoff (*t* test *P* < 0.05). Information for down- and upregulation of DEPs in the human retina and brain (temporal cortex, hippocampus, and cerebellum) is listed in Suppl. Tables S4–11, online resource.

#### Functional network and computational analysis

Differentially expressed proteins were classified according to KEGG pathways and biological processes using the Cytoscape stringApp plugin (http://apps.cytoscape.org/apps/stringapp). Significantly changed proteins were loaded into Cystoscape, and the *Homo sapiens* protein database in the StringDB was selected to reveal protein interactions in the context of enriched pathways. Detectable protein hierarchies, displayed as heatmaps from R. Venn diagram, were created using Venny 2.1 (https://bioinfogp.cnb.csic.es/tools/venny/). Volcano plots were created using Prism9 (GraphPad). DAVID pathway analysis was performed using the David Bioinformatic Database (https://david.ncifcrf.gov/). The pie chart showing the PANTHER-functional classification analysis was created using the online PANTHER tool: http://pantherdb.org/geneListAnalysis.do. Biological functions analysis was performed using Ingenuity Pathway Analysis (IPA) by Qiagen. For the comparison of retinal data against brain cortex proteome data from the literature [7, 44, 94, 114], proteins were regarded as DEPs if the *t* test *P* value was less than 0.05 and determined to be up- or downregulated based on the log fold change of AD/control (> 0 upregulated, < 0 downregulated; Suppl. Tables S12 and 13, online resource). R Shiny tool (https://insightstats.shinyapps.io/meta-checker/) was developed and used to visualize the DEPs overlap.

### Statistical analysis

Analyses were performed using GraphPad Prism version 9.3.1. One- or two-way analysis of variance (ANOVA) was applied for comparisons between 3 or more groups followed by Tukey’s post hoc multiplicity adjustment. In 2-way ANOVA analyses, the *P*d (diagnosis), *P*l (IR/OR layers), *P*r (C/M/F regions), *P*_S_ (sex), and/or *P*i (interactions) were presented. Unpaired or paired Student’s t tests were applied for two-group comparisons. The statistical association between two or more Gaussian-distributed variables was determined by Pearson’s correlation coefficient (*r*) test with Holm-Bonferroni correction for multiple analyses as required. Scatterplot graphs present the null hypothesis of pair-wise Pearson’s *r* with the unadjusted *P* values that indicate direction and strength of the linear relationship between two variables. For multiple comparisons in groups of 6 retinal markers, 7 brain regions, or 36 retinal markers and brain-cognitive parameters, Pearson’s correlations determined associations between variables with Holm-Bonferroni adjusted *P* values using Statistical Analysis System (SAS) version 9.4 (SAS Institute). Multiple linear regression models were made with the MMSE score as the outcome and two explanatory variables, one a marker of retinal gliosis and the other retinal amyloidosis controlled for markers of brain pathology (atrophy, Aβ plaques, or NFTs). Model fit was assessed using R-squared as all models had two explanatory variables. All tests were two sided with < 0.05 significance level. Results are expressed as the mean ± standard deviation (SD) or standard error of the mean (SEM). Degrees of significance are presented as: *P < 0.05, **P < 0.01, ***P < 0.001, and ****P < 0.0001. Data analysis was conducted with coded identifiers, and analysts remained blinded to the diagnostic group until after completion of all analyses.

## Results

### ***Retinal Aβ***_***42***_*** accumulation within spatiotemporal regions in MCI and AD patients reflect brain pathology and cognitive deficit***

To explore the effects of AD on the retina and determine possible relationships with brain pathology, we conducted histological and biochemical investigations of postmortem retinas and brain tissues from 86 human donors; tissue allocation for various analyses is depicted in Fig. 1a. Initially, 54 retinal tissues and 39 paired brains from deceased human donors with premortem clinical diagnoses of MCI [due to AD; *n* = 11; mean age (years ± SD) 86.7 ± 6.5], AD dementia (*n* = 24; 79.6 ± 13.9), and normal cognition (NC, *n* = 19; 79.5 ± 10.9) were analyzed histopathologically for the key AD hallmark (amyloidosis) and related gliosis and degeneration. Patients’ demographics and clinical and neuropathological information are detailed in Table 1. We first quantitatively determined the geometric and layer distributions of total Aβ_42_ burden (fibrillar and non-fibrillar forms) pathognomonic of AD [41, 68, 99] in superior temporal (ST) and inferior temporal (IT) retinal cross-sections from MCI and AD patients and compared with data from age- and sex-matched NC controls (Fig. 1b, c). Representative microscopic images demonstrate the accumulation of 12F4-positive Aβ_42_ burden along with GFAP^+^-macrogliosis (marking reactive astrocytes and Müller glia cells) and IBA1^+^-microgliosis, as observed in retinas from MCI and AD patients versus NC controls [Fig. 1b, fluorescence and peroxidase-based staining are shown; extended data presenting tile images from central (C) and mid-peripheral (M) and far-peripheral (F) subregions are presented in Suppl. Figs. 1c & 2a, online resource]. Retinal (r)Aβ plaques are occasionally found in the inner nuclear layer (INL; Suppl. Fig. 2b, online resource).

Quantitative IHC analysis of the rAβ_42_-IR area in the ST and IT (ST/IT) retinas showed five- and ninefold increases in MCI and AD patients compared to NC controls, respectively (Fig. 1c,  *P* < 0.0001; extended data in Suppl. Fig. 2c, online resource, shows similar changes between the diagnostic groups when the IR area is normalized to retinal thickness). In these patients, the respective brain (b)Aβ-plaque burden was increased by two–threefold (Fig. 1c, * P* < 0.01–0.0001). A patient with early-onset autosomal dominant AD (ADAD) carrying a *Presenilin 1 (PSEN1)* A260V mutation [90] displayed comparable increases in rAβ_42_ and bAβ-plaque burdens. Further, levels of rAβ_1–42_ determined by a sandwich ELISA assay were on average sixfold increased in an additional cohort of AD patients compared to NC controls (Fig. 1d, *P*<0.001; demographics, clinical, and neuropathological information on human donors whose tissues were used for protein analyses are detailed in Table 1). A comparable increase was detected in an ADAD patient carrying a *PSEN1* A431E mutation [82]. No confounding age, sex, or postmortem interval (PMI) factors significantly affected the rAβ_42_ load (Suppl. Fig. 2d–h, online resource).

Next, we studied the ultrastructure of rAβ_42_ deposits using transmission electron microscopy (TEM; Fig. 1e panel; extended TEM images are shown in Suppl. Fig. 3, online resource). Immunogold-12F4 analysis validated the existence of typical punctate Aβ_42_ clusters (red arrows) in the ILM/innermost layers of the AD retina (Fig. 1e, left). Peroxidase-based TEM imaging shows a 3D morphology of 12F4^+^-Aβ_42_ dense-core plaque with emanating fibril arms, detected in retinal inner layers including the ILM of AD patients (Fig. 1e middle, 3D-display of vertical cross-sectional and en face images). Deposits of Aβ_42_ were also observed inside Müller cell (MC) endfeet (Fig. 1e, red arrows, middle-right images).

Mapping of the spatial and layer-specific distribution of rAβ_42_ in our cohort (Fig. 1f–i) revealed uneven burdens across 12 predefined subregions. These subregions are the C, M, and F from the ST and IT quadrants as described in Fig. 1a, defined within the inner and outer retina (IR and OR, respectively, between the inner (ILM)- and outer (OLM)-limiting membranes; Fig. 1h). Figure 1f upper pie chart illustrates the distribution of raw rAβ_42_-IR area with non-significant trends of higher burden in the C versus M and F subregions and significantly higher burdens in the IR versus OR subregions [Fig. 1i; IR (74–86%) vs. OR (14–26%), *P* < 0.0001]. The normalized data to retinal thickness (density) were also analyzed since retinal tissue greatly differs throughout the C, M, and F subregions, being thickest near the optic nerve head and thinnest toward the ora serrata. Unlike the total burden, rAβ_42_ density as measured in the normalized index was highest in the F subregion (Fig. 1f, lower pie chart), reaching statistical significance in the comparison of F versus C subregions in the AD group (Fig. 1g,  *P*< 0.0001). No difference between ST and IT quadrants was detected (Suppl. Fig. S4b, online resource). Regardless of whether normalized or raw data, all subregions showed significantly higher rAβ_42_ signals in MCI and AD patients compared with NC controls (Fig. 1g, i; extended data in Suppl. Fig. 4a–d, online resource), except for the OR in the MCI versus NC groups. Notably, the most significant increases in rAβ_42_ burden in the AD versus MCI or NC groups were found in the M and F subregions.

We next asked whether retinal Aβ_42_ pathology reflected the severity of brain pathologies and cognitive deficit. To this end, we performed a series of Pearson’s correlation coefficient (*r*) analyses between retinal Aβ_42_ burden and disease parameters. A positive association was detected between rAβ_42_ burden and bAβ-plaque scores (Fig. 1j; *r* = 0.64, *P* < 0.0001). A summary of the relationship between rAβ_42_ burden and bAβ plaques in different regions is depicted in a mid-sagittal brain illustration (Fig. 1k). The severity of plaques in all brain regions significantly correlated with rAβ_42_ burden (Fig. 1l, corrected values for multivariable analyses), except for the VA. The strongest correlations between rAβ_42_ burden and plaque severity were found for the entorhinal and temporal cortices (EC, *r* = 0.72, *P* < 0.0001; T. Ctx, *r* = 0.63, *P* = 0.0003, respectively; Fig. 1j–l; extended data for various retinal and brain subregions in Suppl. Fig. 4e–k, online resource). In relation to brain-regional tauopathy, multivariable Pearson’s correlation analyses revealed that rAβ_42_ burden significantly correlated with NFT in the EC (*r* = 0.57, *P* = 0.033) and with NT burden in the T. Ctx (*r* = 0.62, *P* = 0.0098) and parietal (P.) Ctx (*r* = 0.55, *P* = 0.030; Fig. 1l). Further, rAβ_42_ burden was significantly associated with Braak stage [47] (*r* = 0.69, *P* < 0.0001; extended data on ABC (Amyloid/Braak/CERAD) scores shown in Suppl. Fig. 4l, online resource).

To assess if rAβ_42_ burden can differentiate subjects with high (H) versus low (L) brain parameters that define AD diagnosis, we stratified our cohort based on brain Aβ deposition (A), tauopathy (T), and neurodegeneration (N) histopathological scores (modified from NIA-AA’s live imaging of ATN biomarkers) [49], regardless of diagnostic group. In accordance with the clinical diagnosis, rAβ_42_ burden clearly differentiated between H and L subjects, with 5.9-fold higher rAβ_42_ burden in ATN^H^ versus ATN^L^ subjects (*P* < 0.0001; Fig. 1n; extended data on separate A, T, and N stratification in Suppl. Fig. 4m, online resource). Notably, a strong inverse correlation was found between rAβ_42_ burden and Mini–Mental State Examination (MMSE) cognitive scores (Fig. 1o; *r* =  − 0.88, *P* < 0.0001; extended data per retinal subregion in Suppl. Fig. 4n, online resource), surpassing the correlation observed for brain plaques and cognition (*r* =  − 0.67, *P* = 0.0009).

### Identification of intraneuronal Aβ oligomers with increases in MCI and AD retinas

**Fig. 2:**
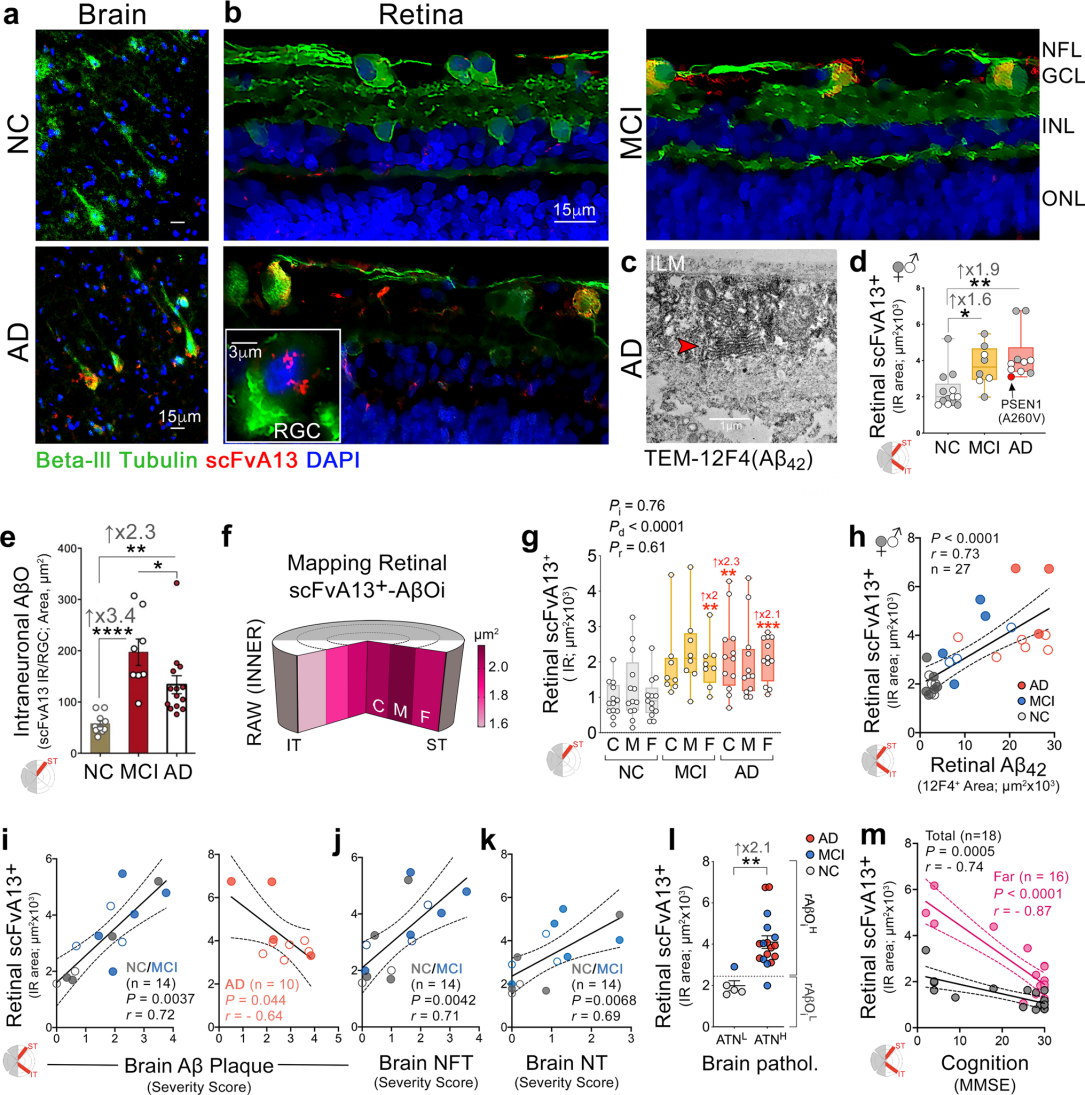
Identification and mapping of scFvA13^+^-AβOi in the retina of MCI and AD patients. **a** Representative microscopic images of postmortem brain cross-sections show the existence of intracellular Aβ oligomers (rAβOi) detected by scFvA13 (red), a conformation-sensitive and sequence-specific antibody in the format of a single-chain Fv fragment (scFv), selectively recognizing AD-relevant AβOs inside cortical pyramidal neurons in AD patients with a minimal signal in NC controls. **b** Microscopic images of retinal cross-sections showing rAβOi inside βIII-tubulin^+^-ganglion cells and nerve fibers (green) in MCI and AD patients, while less so in NC controls. Scale bar: 15 µm. Insert image: AβOi-positive retinal ganglion cell (RGC). Scale bar: 3 µm. **c** TEM micrograph shows subcellular localization of 12F4^+^-Aβ_42_ (DAB, black) in RGC’s endoplasmic reticulum (red arrowhead). Scale bar: 1 µm. **d** A quantitative analysis of scFvA13^+^-AβOi-IR area in ST/IT retina in MCI and AD patients vs. NC controls (*n* = 31; red circle, an ADAD patient with *PSEN1*-A260V mutation). Data presented as median and lower and upper quartiles. **e** Bar graph with individual data points displays the scFvA13^+^-AβOi-area colocalized within βIII-tubulin^+^-RGCs. **f** Pie chart of inner retinal AβOi immunoreactive area distributed in C, M, and F subregions; higher burden shown by darker pink. **g** Quantitative scFvA13^+^-AβOi-IR area for C, M, and F subregions in the ST retina in MCI and AD patients vs. NC controls. Statistics: red asterisks mark significance relative to the NC control group. *P*_i_–interactions; *P*_d_–diagnostic groups; *P*_*r*_–C, M, vs. F subregions. **h–k** Pearson’s correlation coefficient (*r*) analysis between scFvA13^+^-rAβOi load and (**h**) rAβ_42_ area, (**i**) brain (b)Aβ-plaque, (**j**) bNFT, and (**k**) bNT severity scores. **l**. Retinal AβOi-burden in human donors stratified based on high(H) versus low(L) brain ATN histopathological scores; extrapolated dotted-gray line marks the rAβOi level separating ATN^H^ from ATN^L^ individuals. **m** Pearson’s correlations of ST/IT rAβOi vs. MMSE cognitive scores. Data points are presented with group means ± SEMs. *P < 0.05, **P < 0.01, ***P < 0.001, ****P < 0.0001, by 1-way or 2-way ANOVA with Tukey’s post hoc multiple comparison test

To determine the existence of synaptotoxic AβO [68, 98] in the human retina, cross-sections were labeled for the neuronal-specific βIII-tubulin marker with a conformation-sensitive and sequence-specific antibody, in the format of a single-chain Fv fragment (scFv), selectively recognizing AD-relevant AβOi species [74, 75, 97]. ScFvA13 immunoreactivity was detected predominantly within RGCs and their axonal projections and in cortical pyramidal neurons in MCI and AD patients, compared with low signals in NC controls (Fig. 2a, b). A TEM analysis of retinas from AD patients detected 12F4^+^-Aβ_42_ accumulation in the endoplasmic reticulum compartment (Fig. 2c), comparable with the subcellular localization of scFvA13^+^-AβOi in RGCs (Fig. 2b). Increased ST/IT retinal ScFvA13 immunoreactivity was measured for MCI (1.6-fold, *P* < 0.05) and AD (1.9-fold, *P* < 0.01) versus NC (Fig. 2d; extended data showing no significant effect of age, PMI, or sex on levels of retinal AβOi in Suppl. Fig. S5a–c, online resource). While total scFvA13^+^-AβOi burden plateaued in MCI patients and did not further increase in AD (Fig. 2d), the mean area of intraneuronal scFvA13^+^ per ganglion cell increased in these patients versus NC controls (2.3–3.4-fold, *P* < 0.01–0.0001) but decreased in AD compared to MCI patients (*P* < 0.05, Fig. 2e). Mapping of retinal scFvA13^+^-AβOi demonstrated a nonuniform distribution, with mean loads higher in the ST and, moreover, in the ST-M subregion (Fig. 2f). The increase in rAβOi burden in MCI and AD patients versus NC controls was most statistically significant in the ST-F subregion (Fig. 2g; extended data in Suppl. Fig. 5d–f, online resource). Interestingly, rAβOi load directly correlated with rAβ_42_ burden (Fig. 2h; *r* = 0.73, *P* < 0.0001), suggesting the interconnection between these two Aβ forms accumulation in the retina.

To assess the relationship between retinal scFvA13^+^-AβOi burden and disease status, we first conducted Pearson’s correlations with the respective brain AD pathology. Since no significant correlations were found between rAβOi and brain amyloid plaques, NFT, NT, or atrophy for combined diagnostic groups (Suppl. Fig. 5g–k, online resource) and based on early accumulation in the MCI retina (Fig. 2d, e), we performed separate correlation analyses for the NC/MCI and the AD groups. We detected significant linear correlations between the rAβOi load and severity scores of bAβ plaques, NFTs, and NTs among NC controls and MCI patients (Fig. 2i–k; *r* = 0.72–0.69, *P* = 0.0037–0.0068), whereas an inverse correlation was found for bAβ plaques in AD patients (Fig. 2i; *r* =  − 0.64, *P* = 0.044). Further, retinal AβOi burden differentiated individuals with combined ATN^H^ versus ATN^L^ brain histopathology (Fig. 2l; *P* < 0.01), but not between each A, T, and N neuropathological scores separately (Suppl Fig. 5l, online resource). Importantly, we revealed a strong negative association between rAβOi burden and cognitive MMSE scores (Fig. 2m; *r* =  − 0.74, *P* = 0.0005), with AβOi in the retinal far periphery appearing as the strongest predictor of cognitive status (*r* =  − 0.87, *P* < 0.0001; extended data on retinal C, M, and F subregions in Suppl. Fig. 5m, online resource).

### Increased retinal macrogliosis in MCI and AD patients is linked to Aβ pathology and may predict cognitive decline

**Fig. 3:**
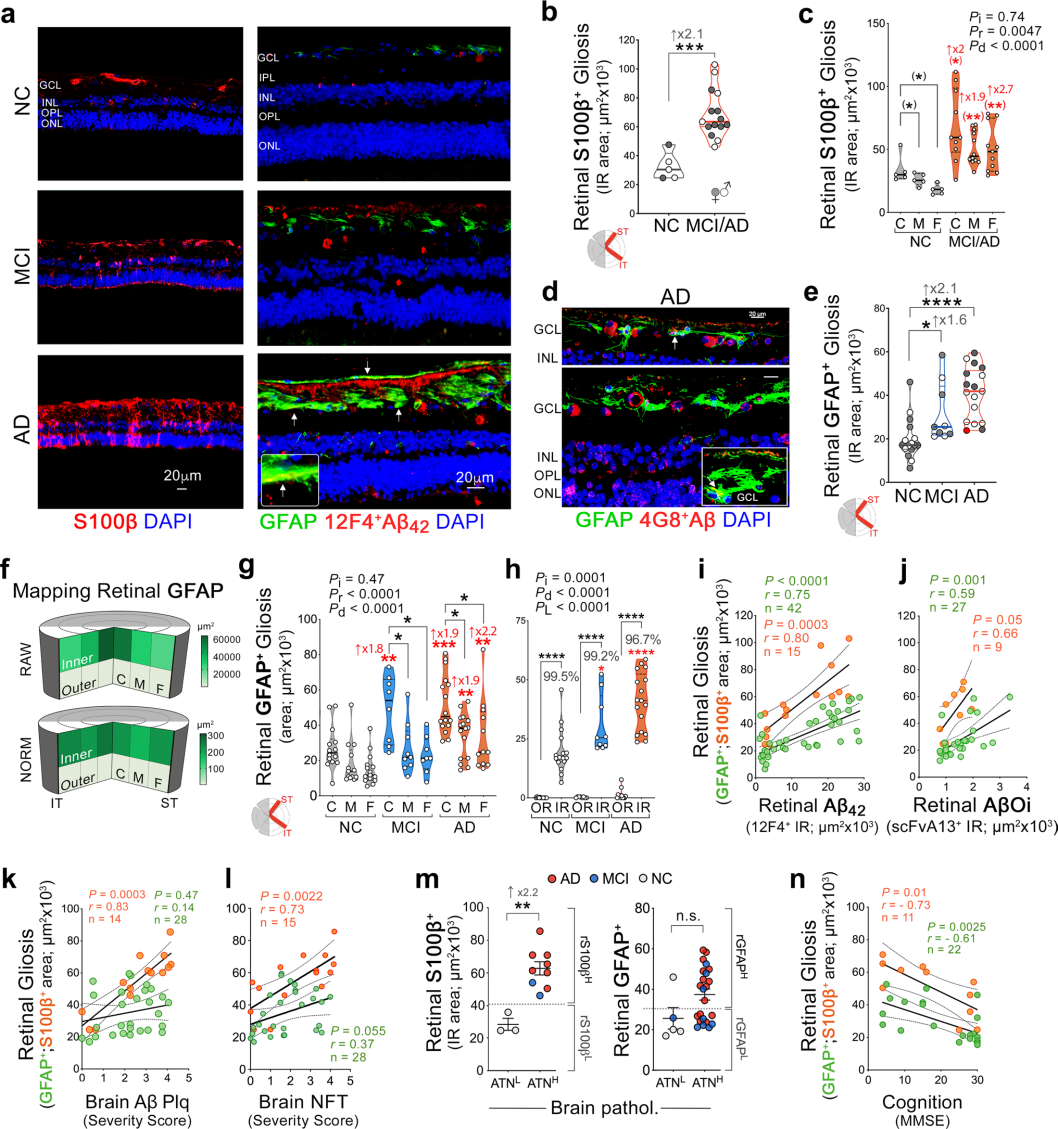
Distribution of macrogliosis in retinas of MCI and AD patients and relations to disease status. **a** Representative fluorescence micrographs of retinal cross-sections immunolabeled for S100β^+^ (red)- or GFAP^+^ (green), markers of reactive astrocytes and Müller glia. Retinal (r)GFAP^+^ macrogliosis is detected surrounding sites of 12F4^+^-Aβ_42_ deposits (red), especially in the ganglion cell layer (GCL) in patients with MCI or AD versus NC. White arrows indicate Aβ colocalized within GFAP^+^ macroglia. **b**, **c** Violin plots display quantitative IHC analyses of (**b**) rS100β-immunoreactive areas in ST/IT retina; total *n* = 20 patients, and (**c**) rS100β-positive area per C, M, and F subregion. **d** Representative images from AD patients stained for 4G8^+^-Aβ (red), GFAP^+^ reactive astrocytes (green), and DAPI nuclei (blue). **e.** A quantitative IHC analysis of GFAP-positive areas in the ST/IT retina in patients with NC (*n* = 16), MCI (*n* = 8) and AD (*n* = 17; red circle, an ADAD patient with *PSEN1*-A260V mutation). **f** Pie charts show GFAP^+^ macrogliosis distribution (raw and normalized to tissue thickness) in the inner retina (IR) and outer retina (OR) and in C, M, and F subregions; higher burden shown by darker green. **g, h** Quantitative GFAP-positive area analyses in patients with MCI (*n* = 8–9), AD (*n* = 12–17), and NC (*n* = 15–16) in the ST/IT retina, separated for C, M, and F subregions (**g**), and inner versus outer retinal (IR vs. OR) layers (**h**). GFAP^+^ macrogliosis is almost exclusively detected in the IR layers (% of total area). **i–l** Pearson’s correlations of rS100β^+^ or rGFAP^+^ macrogliosis against (**i**) rAβ_42_-immunoreactive area, (**j**) rAβOi-immunoreactive area, (**k**) brain Aβ plaque score, and (**l**) NFT score. **m** rS100β^+^ and rGFAP^+^ macrogliosis burden in subjects stratified based on high(H) or low(L) brain ATN histopathological scores; extrapolated dotted-gray lines mark rS100β^+^ level (but not rGFAP^+^-gliosis) separating ATN^H^ from ATN^L^ individuals. **n.** Pearson’s correlations of rS100β^+^ or rGFAP^+^ macrogliosis against MMSE cognitive scores. Data points are presented with group means ± SEMs. Filled and empty circles represent women and men, respectively. Median and lower and upper quartiles are indicated on each violin plot; red asterisks mark significance relative to the NC control group: *P*_i_–interactions, *P*_*r*_–C, M, vs. F subregions, *P*_L_–retinal IR vs. OR layers,* P*_d_–diagnostic groups. Statistics: *P < 0.05, **P < 0.01, ***P < 0.001, by one-way or two-way ANOVA and Tukey’s post hoc multiple comparison test

Neuroinflammatory responses have a central role in AD pathogenesis [67, 72]. In the brains of AD patients and animal models, astrocytes become reactive and exhibit aberrant activation states surrounding Aβ deposits; these cells have been shown to express high levels of GFAP and S100β markers [21, 51]. In the retina, both markers are upregulated in astrocytes and Müller glial cells under inflammatory or neurodegenerative conditions [14, 112]. Here, we observed increases in S100β^+^ and GFAP^+^ macrogliosis, labeling reactive astrocytes and Müller glia, in the retinas of MCI and AD patients relative to NC controls (Fig. 3a). Whereas S100β^+^-macrogliosis appeared in all retinal layers in the patients, GFAP^+^-macrogliosis was almost entirely observed in the innermost retinal layers and often surrounded Aβ deposits. Occasionally, retinal Aβ were found to colocalize within GFAP^+^ macroglia (white arrows). Quantitative analyses of rS100β^+^-gliosis burden and distribution showed 1.9–2.7-fold increases in MCI and AD patients versus NC controls in the ST/IT retina and per C, M, or F subregions (Fig. 3b, c; *P* < 0.001–0.05), with trends of higher rS100β^+^ gliosis in the C versus M and F subregions (Fig. 3c).

In the AD retina, GFAP^+^ astrocytes were found to encircle blood vessels positive for 4G8^+^Aβ deposits and were concentrated near Aβ-containing ganglion cells (Fig. 3d). Like rS100β, a quantitative analysis of rGFAP^+^ macrogliosis in a larger cohort (*n* = 42) revealed 1.6- and 2.1-fold increases in MCI and AD patients versus the NC group (Fig. 3e; *P* < 0.05 and *P* < 0.0001, respectively). No significant differences were observed with age, PMI, or sex for both macrogliosis markers (Suppl. Fig. S6a–f, online resource). Mapping of the rGFAP^+^-IR area (raw and normalized data) showed higher macrogliosis in C- versus M and F subregions (Fig. 3f, g; *P*_region_ < 0.0001), in which 97–99% occurred in the IR compared to the OR (Fig. 3h; *P*_Layer_ < 0.0001; extended data Suppl. Fig. 6g–i, online resource). Notably, the largest early increases in rGFAP^+^ macrogliosis between MCI and NC retinas were found in IR layers and C subregions (Fig. 3g, h).

We next found, as predicted, a tight correlation between expression levels of S100β and GFAP macrogliosis markers in the retina (*r* = 0.75, *P* = 0.0008; Suppl. Fig. 6j, online resource). Moreover, there were strong associations between both macrogliosis markers and retinal amyloidosis: rAβ_42_ (rGFAP: *r* = 0.75, *P* < 0.0001; rS100β: *r* = 0.80, *P* = 0.0003) and rAβOi (rGFAP: *r* = 0.59, *P* = 0.001; rS100β: *r* = 0.66, *P* = 0.05; Fig. 3i, j). Regarding the relationship with respective AD brain pathology, retinal S100β, not rGFAP, strongly correlated with the severity of brain Aβ plaque, NFT, and NT (Fig. 3k, l; bAβ: *r* = 0.83, *P* = 0.0003; NFT: *r* = 0.73, *P* = 0.0022; NT: *r* = 0.67, *P* = 0.0062; extended data in Suppl. Fig. 6k, l, online resource). Interestingly, rS100β but not rGFAP differentiated individuals with combined ATN^H^ versus ATN^L^ and separated A^H/L^ from T^H/L^ brain histopathology (Fig. 3m,  *P*< 0.01; extended data in Suppl. Fig. 6m, n, online resource). These findings suggest that retinal S100β macroglia may be a more sensitive indicator of brain AD pathology than rGFAP. Nevertheless, both glial cell markers inversely correlated with patients’ MMSE cognitive scores (Fig. 3n, rS100β: *r* =  − 0.73, *P* = 0.01; rGFAP: *r* =  − 0.61, *P* = 0.0025; extended data for C, M, and F subregions in Suppl. Fig. 6o, p, online resource).

### Increased retinal microgliosis in MCI and AD with impaired Aβ uptake, and association to cognitive deficit but not brain pathology

**Fig. 4:**
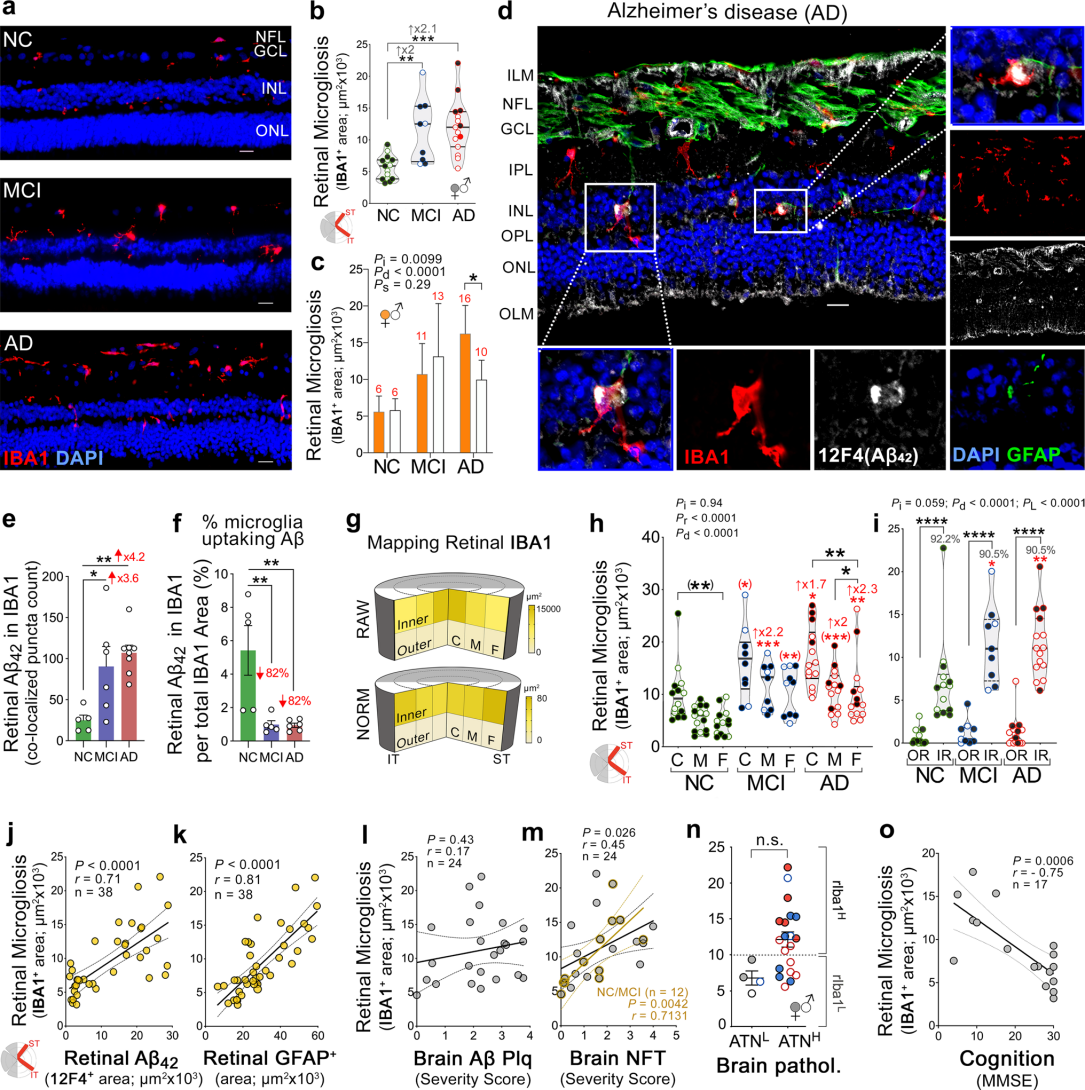
Distribution of retinal microgliosis, Aβ_42_ phagocytosis, and relationships to disease status.** a** Representative fluorescence micrographs showing IBA1^+^ microgliosis (red) in retinal cross-sections from NC, MCI, and AD patients. Scale bar: 20 µm. **b** Violin plot displays quantitative IHC analysis of rIBA1^+^-immunoreactive area in subjects with NC (*n* = 15), MCI (*n* = 9), and AD (*n* = 15; red circle, an ADAD patient with *PSEN1*-A260V mutation). **c** Bar graph displays rIBA1^+^ microgliosis by sex in NC (*n* = 9F/6 M), MCI (*n* = 6F/3 M,) and AD groups (*n* = 5F/10 M). **d** Fluorescence micrograph shows rIBA1^+^ microgliosis (red) colocalized at sites of 12F4^+^-Aβ_42_ deposits (white) with GFAP^+^ macrogliosis (green) and DAPI nuclei (blue). Scale bar: 20 µm. Retinal IBA1^+^ microglia often internalize rAβ_42_ (enlarged images). **e** Quantitative analysis of co-localized 12F4^+^-Aβ_42_ puncta count with IBA1^+^ microglial cells. **f** Percent 12F4^+^-Aβ_42_ puncta count co-localized with IBA1^+^ microglia of total retinal IBA1^+^ microglia. **g** Pie charts show rIBA1^+^ microgliosis distribution (raw and normalized to tissue thickness) in IR, and OR, and in C, M, and F subregions, with higher burden marked by darker yellow. **h, i** Quantitative IBA1-positive area in the ST/IT retinas of patients with NC (*n* = 14–15), MCI (*n* = 9–10) and AD (*n* = 14–15), separated for (**h**) C, M, and F subregions and for (**i**) inner versus outer retinal (IR vs. OR) layers. **j–m** Scatterplot displays Pearson’s correlations between rIBA1^+^ microgliosis and (**j**) rAβ_42_, (**k**) rGFAP, (**l**) brain Aβ plaque, and (**m**) NFT scores. **n** rIBA1^+^ microgliosis in subjects stratified based on high(H) or low(L) brain ATN-histopathology; extrapolated dotted-gray line marks potential rIBA1^+^ microgliosis level for separating ATN^H^ from ATN^L^ individuals. **o** Scatterplot displays Pearson’s correlation between rIBA1.^+^ microgliosis and MMSE cognitive scores. Data points are presented with group means ± SEMs. Filled and empty circles represent women and men, respectively. Median and lower and upper quartiles are indicated on each violin plot; red asterisks mark significance relative to the NC control group: *P*_d_–diagnostic groups; *P*_s_–sex groups; *P*_i_–interactions. Statistics: *P < 0.05, **P < 0.01, ***P < 0.001, by one-way or two-way ANOVA and Tukey’s post hoc multiple comparison test, or Student *t* test (in parenthesis)

To study another key aspect of neuroinflammation that is triggered by Aβ accumulation in the AD brain, we next mapped and quantified IBA1^+^ microgliosis in ST/IT retinal cross-sections from a subset of our cohort (*n* = 39; Fig. 4 and Suppl. Fig. 7, online resource). Examination of retinal microgliosis revealed early and marked elevation of the IBA1^+^-IR area in MCI and AD patients compared to NC controls (2.0- and 2.1-fold increases, *P* < 0.01 and *P* < 0.001, respectively), with no difference between AD and MCI patients (Fig. 4a, b). Notably, rIBA1^+^ microgliosis was 1.6-fold higher in female versus male AD patients but was not affected by age or PMI (Fig. 4c,  *P*< 0.05; extended data in Suppl. Fig. 7b, c, online resource). High-resolution images identified the presence of intracellular Aβ_42_ deposition in IBA1^+^ microglia, especially in MCI and AD retinas, suggesting direct microglial involvement in retinal Aβ phagocytosis (Fig. 4d; extended images in Suppl. Fig. 7a, online resource). Quantification of retinal Aβ_42_ colocalized in IBA1^+^ microglia showed 3.6- and 4.2-fold higher Aβ_42_ puncta counts internalized within microglia in MCI and AD versus NC controls (Fig. 4e,  *P*< 0.05 and *P* < 0.01, respectively). This result is expected since significantly more Aβ_42_ burden and IBA1 cells are detected in the retinas of these patients. In contrast, an analysis of Aβ_42_ puncta within the microglial cell portion in all IBA1^+^ microglia revealed 82% lower Aβ_42_-positive microglia in MCI and AD retinas than in NC retinas (Fig. 4f,  *P*< 0.01). Our data indicate relatively fewer microglial cells engaged in Aβ_42_ uptake, suggesting impaired Aβ phagocytosis by retinal microglia in MCI and AD patients.

Mapping of rIBA1 distribution indicated that microgliosis was significantly greater in the C subregion (close to the optic disc) than in M and F subregions, did not differ between ST and IT regions, and very significantly (91%–92%) aggregated in the IR rather than the OR (Fig. 4g–i and Supp. Fig. 7d). Normalized data to retinal thickness suggest a trend of denser microgliosis in the ST-F. While microgliosis in all retinal subregions separated between diagnostic groups, M subregions reached a higher significance for differentiating between the MCI and NC groups (Fig. 4h), suggesting accumulation of microgliosis in the retinal M subregion in the earliest stages of cognitive impairment.

The relationship between retinal microgliosis and other AD-related pathologies in the retina and brain was next determined by Pearson’s correlation analyses (Fig. 4j–m; extended data in Suppl. Fig. 7e–h, online resource). Retinal microgliosis strongly correlated with rAβ_42_ burden and rGFAP^+^ macrogliosis (Fig. 4j, k; *r* = 0.71–0.81, *P* < 0.0001) and moderately correlated with rAβOi and rS100β^+^ macrogliosis (Suppl. Fig. 7e, f, online resource; *r* = 0.61–0.62, *P* = 0.0006 and *P* = 0.047, respectively). Further, retinal microgliosis correlated with brain NFT scores (*r* = 0.45; *P* = 0.026), especially among NC and MCI patients (*r* = 0.71, *P* = 0.0042) but was not reflective of cerebral Aβ plaques, NT, nor atrophy severity scores (Fig. 4l, m and Suppl. Fig. S7g, h, online resource).

Similarly, retinal IBA1^+^ microgliosis differentiated between individuals with high and low cerebral tauopathy (1.7-fold higher in T^H^ vs. T^L^ cases; *P* < 0.05) but did not distinguish between combined brain ATN^H^ and ATN^L^ cases or cases with high versus low cerebral amyloid or atrophy, separately (Fig. 4n and Suppl. Fig. S7i, online resource). These data suggest a possible connection between retinal microgliosis and brain tauopathy. Moreover, an inverse and strong correlation was detected with the MMSE cognitive status (Fig. 4o,  *r*=  − 0.75; *P* = 0.0006; extended data for retinal subregions in Suppl. Fig. 7j, online resource).

### Retinal atrophy associated with retinal and brain pathology and cognitive status

**Fig. 5:**
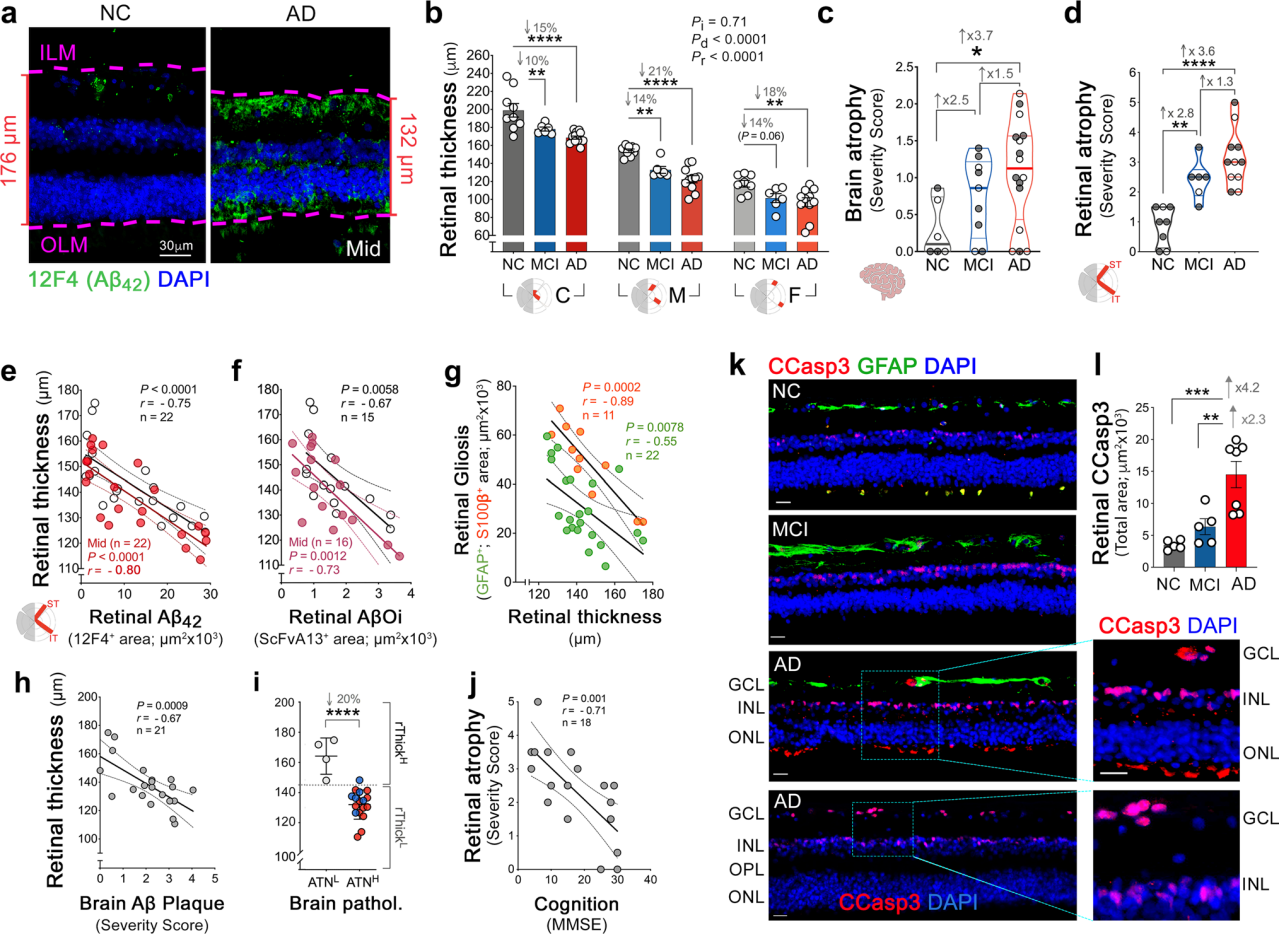
Retinal atrophy in MCI and AD patients in relation to retinal and brain pathologies and cognition. **a** A reduction in tissue thickness is shown in representative retinal cross-sections from an AD patient (132 µm) vs. NC control (176 µm). Thickness was measured from ILM to OLM (purple dashed lines). Scale bar: 30 µm. **b** Histomorphometric analysis of retinal thickness in the ST/IT retina per C, M, and F subregions in patients with AD (*n* = 11), MCI (*n* = 6), and NC (*n* = 8–9). Two-way ANOVA: *P*_i_–interactions, *P*_d_–diagnostic groups, and *P*_r_–retinal subregions. **c**, **d** Quantitative analysis of (**c**) brain atrophy severity scores in a subset of human donors with AD (*n* = 16), MCI (*n* = 9), or NC (*n* = 6), and (**d**) ST/IT retinal atrophy scores for an overlapping subset of patients with AD (*n* = 11), MCI (*n* = 6), or NC (*n* = 8 or 9). **e–g** Scatterplots display Pearson’s correlations between retinal thickness and retinal (**e**) Aβ_42_ burden, (**f**) AβOi, and (**g**) S100β^+^ or GFAP^+^ macrogliosis. Color-filled dots represent these correlations in retinal mid-peripheral subregions. **h** Pearson’s correlation between retinal thickness and brain Aβ plaques. **i** Retinal thickness in subjects stratified based on high(H) or low(L) brain ATN-histopathology; extrapolated dotted-gray line marks the retinal thickness level for separating ATN^H^ from ATN^L^ individuals. **j** Pearson’s correlation between ST/IT retinal atrophy and MMSE cognitive score. **k** Representative retinal cross-sections labeled for cleaved caspase-3 (CCasp3; red) early apoptotic marker, GFAP (green), and DAPI nuclei (blue) in NC, MCI, or AD patients. Zoomed-in inserts are provided for representative images from the 2 AD patients to illustrate the presence of CCasp3^+^ cells within the INL and GCL. **l** Quantitative CCasp3-immunoreactive area in the ST/IT retina (*n* = 17). *P < 0.05, **P < 0.01, ***P < 0.001, ****P < 0.0001 by 1-way or 2-way ANOVA and Tukey’s post-hoc multiple comparison test

We next investigated whether retinal degeneration occurs during early stages of cognitive impairment (MCI) and if the intensified retinal amyloidosis and gliosis observed in MCI and AD patients are linked to retinal degeneration. To this end, we conducted a histomorphometric analysis of retinal thickness in the C, M, and F subregions and found significant tissue thinning in MCI (*P* < 0.01) and AD (*P* < 0.0001) patients versus NC controls, with the largest 14% and 21% retinal thinning, respectively, detected in M subregions (Fig. 5a, b; extended data for total retinal thickness in Suppl. Fig. 8a, online resource). After conversion of retinal thickness to atrophy severity scores, we found similar increases in retinal and brain atrophy in MCI and AD patients versus NC controls (Fig. 5c, d; retina: 2.8–3.6-fold, brain: 2.5–3.7-fold; extended data in Suppl. Fig. 8b, c, online resource), with more significant changes in retinal versus brain atrophy. Retinal thickness was not affected by age, PMI, or sex (Suppl. Fig. 8d–f, online resource). Notably, retinal thickness strongly and inversely correlated with retinal amyloidosis, with stronger correlations for mid-peripheral rAβ_42_ (*r* =  − 0.80, *P* < 0.0001) and rAβOi (*r* =  − 0.73, *P* = 0.0012), and with rS100β^+^ macrogliosis (*r* =  − 0.89, *P* = 0.0002). Retinal thickness was moderately associated with rGFAP^+^ and rIBA1^+^ gliosis (GFAP: *r* =  − 0.55, *P* = 0.0078; IBA1: *r* =  − 0.50, *P* = 0.029; Fig. 5e–g and Suppl. Fig 8g, online resource).

We further investigated whether retinal thinning might be indicative of AD brain pathology by comparing retinal thickness to the severity levels of respective brain amyloid plaque, tauopathy, and atrophy. We found that retinal thickness strongly and inversely correlated with brain Aβ-plaque severity (Fig. 5h; *r* =  − 0.67, *P* = 0.0009) and had moderate to no correlation with brain tauopathy (NFT: *r* =  − 0.49, *P* = 0.025 and NT: *r* =  − 0.33, *P* = 0.12; Suppl. Fig. 8i–l, online resource). A moderate correlation was detected between retinal and brain atrophy (*r* =  − 0.48, *P* = 0.029; Suppl. Fig. S8m, n, online resource). Notably, retinal thickness markedly differentiated cases with ATN^H^ from ATN^L^ brain histopathology [Fig. 5i; *P* < 0.0001; extended data on retinal thinning significantly differentiating (*P* < 0.01) between high and low brain A, T, and N separately in Suppl. Fig S8o, online resource]. Moreover, a strong correlation was noted between retinal atrophy and the MMSE cognitive score (Fig. 5j; *r* =  − 0.71, *P* = 0.001; extended data per retinal subregion in Suppl. 8p, online resource). To assess whether retinal thinning is due to apoptotic cell loss, we analyzed the early apoptotic marker cleaved caspase-3 (CCasp3) in the retinas of a subset of cases. CCasp3^+^ apoptotic cells were observed in the GCL, INL, and ONL/photoreceptor-nuclear layers of AD retinas (Fig. 5k). Significant 2.3-fold and 4.2-fold increases in retinal CCasp3^+^-IR area were detected in the retinas of MCI and AD patients compared to NC controls, respectively (Fig. 5l; *P* < 0.01–0.001). The observed increases in amyloidosis and gliosis in the GCL of MCI and AD patients prompted us to further assess the relative population of GCL cells that undergo early apoptosis. On average, 13.7% (SD ± 12.5) of GCL cells from NC individuals stained positively for CCasp3, whereas this percentage was significantly higher (42.6%, SD ± 7.6) among MCI and AD patients (*P* = 0.00013).

To evaluate the predictability of the studied retinal pathologies in reflecting the severity of brain AD pathology and cognitive status, we conducted multivariable correlation analyses, simultaneously comparing each retinal marker with six brain pathological and cognitive parameters (Table 3). Pearson’s I correlations with Holm-Bonferroni correction for multiple comparisons showed brain Aβ plaques and NFTs were most significantly associated with retinal Aβ_42_, S100β^+^ macrogliosis, and atrophy (*r* = 0.59–0.80, *P* = 0.0088–0.0004), with no association with retinal GFAP^+^ and IBA1^+^ gliosis. Brain NT scores were correlated with retinal Aβ_42_, AβOi, and S100β^+^ macrogliosis, but not with other retinal markers. Brain atrophy was only reflected by retinal atrophy and Aβ_42_. Braak stage strongly correlated with all retinal biomarkers except AβOi (*r* = 0.62–0.86, *P* < 0.0001–0.0055). Furthermore, all retinal markers had a strong correlation with cognitive status, with the strongest being Aβ_42_ (*r* = 0.61–0.88, *P* < 0.0001–0.039).

**Table 3:** Multivariable analysis of retinal pathologies against brain and cognitive parameters

Retina	Brain					
	Aβ Plaque	NFT	NT	Atrophy	Braak Stage	MMSE
Aβ_42_ *n* = 32	***r* = 0.64** ***P* = 0.0004**	***r* = 0.59** ***P* = 0.0009**	***r* = 0.54** ***P* = 0.003**	*r* = 0.40 *P* = 0.023	***r* = 0.69** ***P* < 0.0001**	***r* = − 0.88** ***P* < 0.0001** *n* = 24
AβOi *n* = 14 (NC/MCI)	*r* = 0.72 *P* = 0.022	*r* = 0.71 *P* = 0.022	*r* = 0.69 *P* = 0.027	*r* = 0.40 n.s	*r* = 0.49 n.s	***r* = − 0.74** ***P* = 0.0030** *n* = 18
S100β *n* = 15	***r* = 0.80** ***P* = 0.002**	***r* = 0.73** ***P* = 0.0088**	*r* = 0.67 *P* = 0.019	*r* = 0.46 *P* = 0.088	***r* = 0.86** ***P* = 0.0002**	*r* = ** − **0.73 *P* = 0.021 *n* = 11
GFAP *n* = 28	*r* = 0.24 n.s	*r* = 0.37 n.s	*r* = 0.31 n.s	*r* = 0.18 n.s	***r* = 0.63** ***P* = 0.0018**	*r* = ** − **0.61 *P* = 0.039 *n* = 22
IBA1 *n* = 24	*r* = 0.23 n.s	*r* = 0.50 *P* = 0.062	*r* = 0.38 n.s	*r* = 0.0026 n.s	***r* = *0.65*** ***P* = *0.0036***	***r* = − 0.75** ***P* = 0.0036** *n* = 17
Atrophy *n* = 24	***r* = 0.64** ***P* = 0.0042**	***r* = 0.62** ***P* = 0.0055**	*r* = 0.33 n.s	*r* = 0.56 *P* = 0.0092	***r* = 0.62** ***P* = 0.0055**	***r* = − 0.71** ***P* = 0.0069** *n* = 18

Multivariable analysis chart presents Pearson’s (*r*) correlations and Holm-Bonferroni corrected *P* values for each retinal pathology against 6 brain parameters and cognition (bolded values are also significant after Holm-Bonferroni corrected *P* adjustment for 36 combined retina/brain-parameters)

AβOi, scFvA13^+^ intra-neuronal oligomers

*NFT* neurofibrillary tangles, *NT* neuropil threads, *n.s.* not significant

### Exploring the proteome landscape of Alzheimer’s disease in the retina and brain

**Fig. 6:**
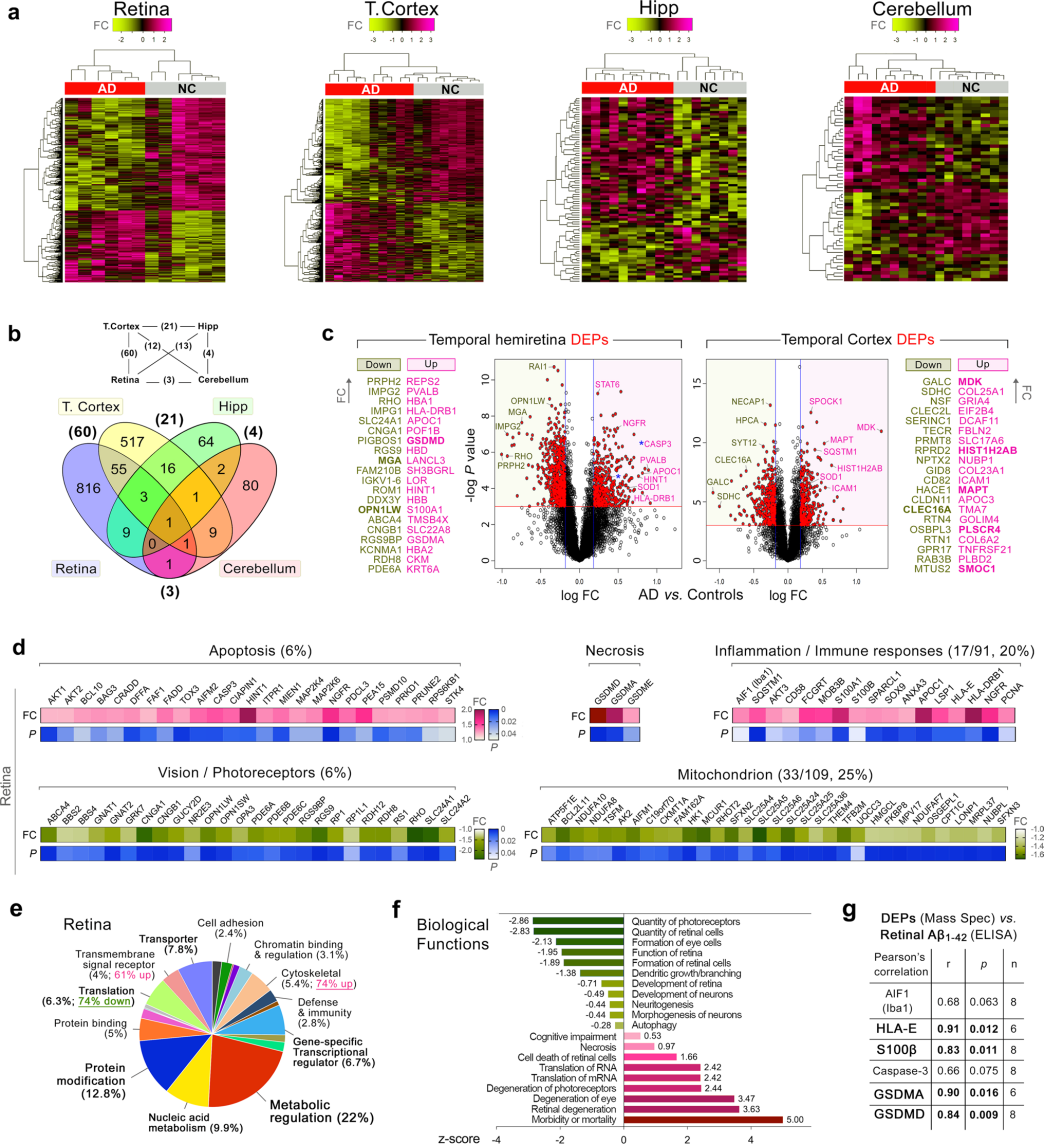
Proteomic landscape of the retina and brain in AD. **a** Proteomics profiling of retinal (*n* = 6 AD; *n* = 6 NC) and brain (*n* = 10 AD; *n* = 8 NC) tissues. Heatmaps display detectable protein hierarchies from the ST/IT retina (temporal hemiretina), medial temporal gyrus (T.Cortex), hippocampus (Hipp.), and cerebellum; upregulated proteins are shown in pink and downregulated proteins in green. **b** Venn diagram depicting the number of overlapping differentially expressed proteins (DEPs) according to statistical significance (*P* < 0.05) and 1.2-fold change (FC) threshold criteria in the 4 analyzed CNS tissues; the number of common DEPs between paired CNS tissues (bold). **c** Volcano plots and top 20 up- or downregulated DEPs organized by FC (lowest *P* values highlighted in bold) in retinas and temporal cortices of AD vs. NC (DEPs marked by red circles). **d** DAVID-biological classification analysis displays top upregulated DEPs (pink) and top downregulated DEPs (green) in AD vs. NC retinas; lower blue bars represent magnitude of *P* values. Percentages indicate the fraction of each category of total up- or downregulated DEPs. **e** Pie chart of PANTHER-functional cluster analysis showing fraction and percentage of significant DEPs grouped by protein class category in retinas of AD patients vs. NC controls. **f** Ingenuity pathway analysis (IPA) of top up- and downregulated biological functions in AD vs. NC retinas. **g** Pearson’s (*r*) correlations between inflammatory/apoptotic-related DEPs in AD retinas identified by mass spectrometry and retinal Aβ_1–42_ measured by ELISA in the same individuals

The histopathological evidence of AD in the human retina and its significant parallel with paired brain pathologies inspired us to investigate global protein expression profiles of AD retinas and brains (Fig. 6). Hence, to gain a deeper understanding of AD pathological processes in the retina and brain, including inflammation and degeneration, we conducted mass spectrometry (MS) analysis. Proteins were isolated from the temporal hemiretina of AD patients (*n* = 6) and age- and sex-matched NC controls (*n* = 6) as well as from three brain regions in another cohort of AD patients (*n* = 10) and matched NC controls (*n* = 8; demographic, clinical, and neuropathological data on human cohorts are detailed in Tables 1, 2). MS analysis identified 8,286 retinal and 7,312 brain targets, of which 886 retinal differentially expressed proteins (DEPs; > 1.2-fold change cutoff) and 602 temporal cortex, 96 hippocampus, and 95 cerebellum DEPs were detected in AD patients versus NC controls (Fig. 6a; extended data in Suppl. Fig. 9 and Suppl. Tables 4–11, online resource). Heatmaps display AD-specific proteome signatures for each CNS tissue, with similar patterns for the retina and the temporal cortex (Fig. 6a). Venn diagrams identified 60 AD-related overlapping DEPs between the retina and temporal cortex, but only 13 between the retina and hippocampus and 3 between the retina and cerebellum. Fewer AD-related overlapping DEPs were found between the temporal cortex and hippocampus (21), temporal cortex and cerebellum (12), and hippocampus and cerebellum (4), despite being from the same human brains (Fig. 6b; extended data in Suppl. Fig. 9a–c, online resource).

Volcano plots highlight the top AD-specific DEPs identified for the retina and temporal cortex (Fig. 6c; extended data including volcano plots for the hippocampus and cerebellum in Suppl. Fig. 9d–g, online resource). In the AD retina, among the top 20 upregulated proteins by fold change are immune and pyroptosis-related proteins including major histocompatibility complex, Class-II (HLA-DRB1), and gasdermin-D (GSDMD), a protein that is involved in pyroptosis—a programmed cell death. Among the top 20 downregulated DEPs in the retina, more than 50% are photoreceptor markers such as PRPH2 (Peripherin-2), RHO (OPN2; Rhodopsin; Opsin-2), and OPN1LW (a long wave sensitive opsin). In the AD temporal cortex, among the top 20 upregulated proteins are inflammatory-related Midkine (MDK) and microtubule-associated protein tau (MAPT; Fig. 6c; extended data for top 20 up- or down-regulated proteins sorted by *P* value in Suppl. Fig. 9d, e, online resource).

Further, among the top upregulated DEPs common in both, the AD retina and temporal cortex were cell death- and inflammatory-related proteins (Suppl. Fig. 9h, i, online resource). These included histidine triad nucleotide binding protein 1 (HINT1), which hydrolyzes purine nucleotides related to cell death; superoxide dismutase type 1 (SOD1), an enzyme crucial for ROS release and neuronal damage and degeneration; heat shock protein family B member 1 (HSPB1), which increases under stress signals such as inflammation; and heme-binding protein 2 (HEBP2), which promotes mitochondrial permeability transition and necrotic cell death. Among the top downregulated DEPs common in both the AD retina and temporal cortex were mitochondria- and signaling-related proteins. These included solute carrier family 25 members 4 and 5 (SLC25A4 and SLC25A5), which aid mitochondrial ADP/ATP transport and mitochondrial membrane potential; Reticulon 4 (RTN4, also Nogo), which inhibits neuritic outgrowth; G protein subunit beta 5 (GNB5), which integrates signal transduction between receptor and effector protein; and syntaxin-binding protein 1 (STXBP1), which releases neurotransmitters.

The DAVID (database for annotation, visualization and integrated discovery) functional classification of AD retinas revealed that apoptosis, necrosis, and inflammation were the most activated pathways, while vision and photoreceptors, oxidative phosphorylation and mitochondria, and transcription and translation were the most inhibited (Fig. 6d; extended data in Suppl. Fig. 9j–l, online resource). The PANTHER (protein annotation through evolutionary relationship) protein classification showed similar patterns for AD retinas and temporal cortices, in contrast to hippocampi and cerebella (Fig. 6e; extended data in Suppl. Fig. 9m–p, online resource). Ingenuity pathway analysis (IPA) indicated that the top activated biological functions in AD versus NC retinas were related to retinal/photoreceptor degeneration and morbidity or mortality (Z-scores: 3.63–5.00), while the top inhibited functions were related to the quantity of retinal cells and photoreceptors [Z-scores = –2.83 to –2.86); Fig. 6f].

In agreement with our histological data, the MS analysis indicated significant increases in retinal proteins such as S100β, IBA1 (AIF1), and caspase-3 in AD patients versus NC controls (Suppl. Fig. 9q–u, online resource). Notably, very strong correlations were identified between retinal Aβ_1–42_ levels (measured by ELISA) and the MS-detected retinal proteins: MHC class I antigen E (HLA-E; *r* = 0.91, *P* = 0.012), S100β (*r* = 0.83, *P* = 0.011), GSDMA (*r* = 0.90, *P* = 0.016), and GSDMD (*r* = 0.84, *P* = 0.009; Fig. 6g). Overall, these findings suggest that AD predominantly affects the retina and brain through inflammation and mitochondrial dysregulation and neurodegeneration.

## Discussion

How does Alzheimer’s disease affect the human retina? The results of this comprehensive study reveal the impact of AD pathology on the retinas of MCI and AD patients at the molecular, cellular, and structural levels. Proteome analysis identified AD-specific profiles for the retina and three brain regions, with the greatest similarities found between retinas and temporal cortices. AD retinal proteome signatures exhibited overlaps with previously reported DEPs across seven AD brain datasets (Suppl. Tables S12 & 13, online resource) [7], with marked activation of inflammatory and apoptosis–necrosis pathways and inhibition of mitochondrial and ribosomal machinery. It is noteworthy that the AD retina was specifically marked by degeneration of photoreceptor-enriched pathways.

In this study, extensive histological analyses identified the presence of novel intraneuronal Aβ oligomers in MCI and AD retinas, determined the spatiotemporal distribution of these AβOi species along with total Aβ_42_ forms, and found that these retinal Aβ forms were closely associated with enhanced retinal gliosis and atrophy. Alzheimer’s retinopathy, especially retinal Aβ_42_, S100β^+^ macrogliosis, and atrophy, correlated with the severity of neuropathology, and all retinal pathologies but AβOi reflected Braak staging. Interestingly, women exhibited higher levels of retinal IBA1^+^ microgliosis than men, whereas retinal Aβ_42_ and microgliosis displayed the strongest correlations with cognitive status regardless of sex differences.

Mapping of retinal biomarkers revealed that they are not uniformly distributed across retinal subregions and layers but are instead predominantly present in the inner retina and certain retinal subregions of the overall cohort (pie charts). Notably, the accumulation of retinopathies in the early stages of functional impairment (MCI) was more pronounced in the inner layers and peripheral subregions and further progressed in AD dementia. The higher Aβ_42_ density in the far periphery versus central retina may suggest that Aβ_42_ aggregates more readily to plaques in peripheral subregions. In contrast, retinal GFAP^+^ macrogliosis was more significantly elevated in the central subregions closer to the optic disc in MCI and AD patients. To gain a better understanding of this phenomenon, future studies should investigate why certain anatomical regions in the retina are more susceptible than others to disease processes, perhaps due to altered blood flow or cell-type content. This knowledge can be ultimately used to guide retinal imaging modalities, by indicating which areas should be captured to increase specificity for AD detection.

Consistent with our findings, previous histological studies found that the number of Aβ^+^ deposits (clone D54D2) was increased in the retinas of AD patients compared to NC controls, along with increases in retinal GFAP^+^ and IBA1^+^ gliosis and CCasp3^+^ apoptotic cells [34]. Similarly, another study reported significant increases in the percentage of 6F/3D^+^Aβ immunoreactive area and IBA1^+^ microgliosis in the retinal mid-periphery in AD patients versus NC controls [119]. Overall, these and our findings suggest that AD retinopathy is characterized by a spatiotemporal pattern, with the most pronounced changes occurring in the peripheral subregions and inner retina.

Aβ_42_, which is a key hallmark of brain AD pathology, was found here to be considerably increased in MCI retinas, compared with NC retinas, and was closely correlated with Aβ plaque severity in the entorhinal cortex, a brain region affected early and heavily in AD [54]. Note that the results of the present study confirmed our previous findings [57] and went further by identifying a larger, ninefold increase in total Aβ_42_ forms (including fibrillar and non-fibrillar species) in retinal cross-sections from AD patients versus NC controls (compared with the 4.7-fold increase in *Aβ*_*42*_* plaques* previously identified in retinal flat mounts [57]). For the first time, analysis of retinas from MCI patients revealed a substantial fivefold increase in Aβ_42_ burden as compared to retinas from NC controls. The levels of retinal Aβ_42_ forms were significantly higher in patients with AD dementia than in those with MCI, suggesting further accumulation of retinal Aβ_42_ with disease progression. Notably, recent pilot studies that imaged the retinas of living patients reported significant increases in retinal amyloid burden in preclinical AD or MCI patients compared to NC controls [27, 83, 108]. Hence, Aβ_42_ may be an early pathological marker in AD retinas, comparable to brain amyloid pathology, and may be used to facilitate early diagnosis. Interestingly, given that we identified similar retinal changes in persons with familial AD due to *PSEN1* mutations, our data support similarities between downstream retinal manifestations in autosomal dominant and sporadic AD, despite the fact that the initiating pathogenic events are distinct [3, 104].

The present study also provides novel insights into the relationship between retinal and brain AD pathology. Retinal Aβ_42_, S100β^+^ macrogliosis, and atrophy, but not retinal GFAP^+^ macrogliosis or IBA1^+^ microgliosis, reflected cerebral neuropathology and differentiated patients into those with high versus low brain ATN histopathology. Notably, cognitive status correlated very strongly with retinal Aβ_42_, far-peripheral AβOi, and IBA1^+^ microgliosis—the latter resembling the link between brain microgliosis and cognitive decline [17, 29]. Importantly, we also found similar inverse associations between retinal Aβ_42_ burden and cognitive scores using linear regression models after controlling for brain atrophy, NFT, or Aβ plaques [rAβ_42_ (*R*^2^ = 0.75–0.77, *P* < 0.0001–0.0003)]; this indicates the potential of Aβ_42_ burden as a reliable indicator of cognitive impairment. Indeed, a correlation between retinal and brain amyloidosis was found, demonstrating that ST/IT retinal Aβ_42_ burden can strongly predict cerebral Aβ-plaque severity, especially in the entorhinal and temporal cortices, as compared to other brain regions. Our findings suggest that a retinal imaging modality that captures all Aβ_42_ forms and covers central and peripheral ST and IT subregions across all focal planes could be used to detect early AD-specific changes in individuals with MCI and to monitor those patients whose disease progresses to AD dementia.

Our findings suggest that retinal intracellular Aβ oligomer accumulation may be an early biomarker of AD. We found that the scFvA13^+^-AβOi burden plateaued in the MCI stage and did not further increase in AD dementia, which is in line with previous rodent studies [37]. While significant linear correlations were found between retinal AβOi load and the severity scores of brain Aβ plaques, NFTs and NTs among NC and MCI patients, an inverse correlation was found between retinal AβOi and brain Aβ plaques among AD patients. These results suggest that retinal AβOi accumulation may be an early indicator of AD, associated with increased severity of brain pathology in NC and early phase of functional impairment, and decreased severity of brain Aβ plaques in AD dementia patients. Further research is needed to better understand the relationship between retinal AβOi accumulation and AD progression, and to determine if it can be used as a reliable biomarker for early diagnosis.

Astrogliosis and microgliosis in AD brains have been linked to excessive synaptic pruning and cognitive decline [32, 45, 46, 86]. In this study, retinal microglia were identified at the synaptic-rich plexiform layers (IPL and OPL), suggesting their potential involvement in synaptic pruning and functional impairment in AD. Further, studies in microglial cell culture and mouse models of AD have demonstrated that microglia become dysfunctional and fail to clear Aβ, suggesting an important role for these cells in the pathogenesis of AD [6, 35]. Here, we found that microglia in MCI and AD retinas often surround Aβ deposits and are directly involved in Aβ uptake, mirroring their role in the brain [55, 71, 102, 121]. Consistent with findings in a recent report [119], our data demonstrated substantially fewer retinal microglial cells internalizing Aβ_42_ in MCI and AD patients compared to matched NC controls. This suggests that, despite the increased presence of microglia in the retinas of patients with MCI or AD, there is a relative decrease in the number of microglia engaging in Aβ phagocytosis. Such a finding may indicate that the microglia are dysfunctional and unable to effectively clear Aβ, resulting in its accumulation in these patients. Future studies should examine various populations of microglia and their activation states as well as their relationships to Aβ aggregation and synaptic loss in the retinas of MCI and AD patients. Moreover, in agreement with the sexual dysmorphism of inflammatory responses reported in AD brains [25], women exhibited higher retinal microgliosis than men, which correlated with cognitive deficits. Future studies should address this disproportionate female vulnerability to retinal inflammatory process in AD.

Unlike GFAP^+^-gliosis found predominantly in the inner retina, S100β^+^-gliosis was also apparent in the outer retina, implicating the reactivation of several glial cell populations. In response to brain injury, the GFAP and S100β astrocytic markers display differential expression, with scar-forming GFAP^+^-astrocytes surrounding lesion sites and S100β typically marking migrating hypertrophic astrocytes at distal regions [120]. A recent study reported lower levels of the synaptic-protective glutamine synthetase (GS) marker and GFAP^+^ glial cells in the retinas of AD patients compared to controls [119]. In the current study, we found upregulation in GFAP and S100β markers of reactive astrocytes and Müller glial cells in the retinas of MCI and AD patients. Yet, our findings are consistent with other reports showing increases in the expression of these markers in the brains of AD patients [51, 86] and increases in GFAP^+^ gliosis in AD retinas [12, 34], suggesting that neuroinflammation may be a common feature of AD pathology in both the brain and retina. Future studies are warranted to explore the range of reactive astrocyte populations as well as Müller glia activation states and their relationships to AD pathophysiological processes in the retina.

The present study marks the first exploration of the proteome landscape in the AD retina in an independent cohort, which supported our histological demonstration of augmented inflammation and neurodegeneration and further identified novel molecular targets of AD retinopathy. One-fifth of upregulated DEPs in the AD retinas were related to immune responses, including HLA-DRB1, APOC1, S100A1, HLA-E, IBA1, and S100β, and involved lymphocyte and myelomonocyte activation in the presence of neuroinflammation and infection. Indeed, the connection between brain Aβ and inflammation [32, 86] was paralleled in this study by strong correlations between retinal Aβ_42_ and retinal HLA-E, S100β, GFAP, and IBA1.

The impact of AD on retinal degeneration was previously reported [5, 11, 12, 23], linking retinal thinning and cognitive deficits [4, 23, 33, 84, 105, 106]. Although retinal degeneration is not unique to AD [40], here, we found it mirroring atrophy in respective brains and strongly associated with retinal Aβ_42_ and AβOi burdens; these findings together with increases in the CCasp3-apoptotic marker suggest causality for AD-specific retinal atrophy. The activation of apoptosis and a necrosis-enriched pathway and the upregulated DEPs Hint1 and caspase-3, along with GSDMA and GSDMD involved in pyroptotic cell death [103], which strongly correlated with retinal Aβ_1-42_ levels, further highlight a possible connection between Aβ and neurodegeneration in AD retinas, as we earlier demonstrated for retinal vascular amyloidosis and pericyte loss [101]. Since cerebral Aβ oligomers were implicated in AD-related neuronal and synaptic toxicity [8, 41, 56, 64, 110], our findings of Aβ oligomers in RGCs may explain their susceptibility to neurodegeneration in AD [11, 62].

One-quarter of downregulated DEPs in AD retinas were related to mitochondrial dysfunction, as implicated in AD brains [115], and > 70% of the top 20 downregulated DEPs were markers of photoreceptors, retinal cells found to accumulate Aβ_42_ in MCI and AD patients. We postulate that, like highly energy-demanding brain regions such as the EC [54], mitochondria- and activity-rich photoreceptors are especially vulnerable to AD processes. Future studies should investigate retinal cell-specific vulnerability to AD pathology, including tauopathy, in predicting tissue loss and dysfunction and guiding next-generation retinal imaging. Further, the irregular electroretinogram patterns [77], disturbed circadian rhythms [62, 70, 116], and visual abnormalities [40, 77, 89, 93, 113] reported in AD patients may be attributed to the retinal damage found in these patients.

Although our comprehensive histological and biochemical quantification of AD pathologies in the retina across the AD spectrum provides much information and possesses many strengths, we recognize certain limitations. Our study is primarily correlational and, therefore, caution must be taken before making cause-and-effect inferences. Two studies [26, 95] have been unable to consistently identify AD-specific changes in the retina, as described here, but a growing number of independent groups have detected AD hallmark pathology in the human AD retina [18, 22, 24, 27, 28, 34, 38, 39, 57, 59, 62, 65, 66, 80, 83, 88, 96, 101, 108, 119]. Consensus groups or workshops harmonizing on the methodology used when assessing retinas for AD pathologies can be very helpful to clarify this issue.

Taken together, our findings provide novel and deeper understanding of the susceptibility of the retina to AD processes, including molecular, cellular, and structural abnormalities that can be detected in the earliest stages of functional impairment. Furthermore, our study has identified the pathological connections between the retina, brain, and cognition, proposing that the retina could serve as a reliable biomarker for non-invasive AD detection and monitoring.

## Supplementary Information

Below is the link to the electronic supplementary material.

Supplementary file1 (PDF 10451 KB)

## Data Availability

Most data generated or analyzed for this study are included in this published manuscript and supplementary online material. All the processed proteomics data generated in this study have been included in the manuscript and the online supplementary materials. The mass spectrometry raw files and search results have been deposited to the ProteomeXchange Consortium via the PRIDE partner repository. Additional data are available from the corresponding author upon reasonable request.

## Abbreviations

A: Amyloid
Ab: Antibody
ABC: Amyloid/Braak/CERAD score
AD: Alzheimer’s disease
ADRC: Alzheimer’s disease research center
ATN: Amyloid, tauopathy, and/or neurodegeneration
Aβ: Amyloid β-protein
Aβ_42_: 42 Amino acid-long alloform
AβOi: Intracellular oligomers
Aβ-P: Plaques
ANOVA: Analysis of variance
APP: Amyloid precursor protein
ATN: Aβ plaque, tauopathy, and neurodegeneration histopathological scores
(b): Brain
BSA: Bovine serum albumin
C: Central retina
CA: Cornu ammonis
CDR: Clinical Dementia Rating
CNS: Central nervous system
DEP: Differentially expressed proteins; EC—Entorhinal cortex
ELISA: Enzyme-linked immunosorbent assay
F: Far peripheral retina
F. Ctx: Frontal cortex
GFAP: Glial fibrillary acidic protein
GCL: Ganglion cell layer
GSDMA: Gasdermin-A
GSDMD: Gasdermin-D
H: High
Hipp: Hippocampus
IBA1: Ionized calcium-binding adaptor molecule 1
IHC: Immunohistochemistry
ILM: Inner limiting membrane
INL: Inner nuclear layer
IPA: Ingenuity pathway analysis
IPL: Inner plexiform layer
IR area: Immunoreactive area
IR: Inner retina
IT: Inferior-temporal
L: Low
M: Mid-peripheral retina
LOAD: Late-onset AD
mAb: Monoclonal antibody
MCI: Mild cognitive impairment
MMSE: Mini-mental state examination
MS: Mass spectrometry
N: Nasal
NDRI: National disease research interchange
NC: Normal cognition
NFL: Nerve fiber layer
NFT: Neurofibrillary tangle
NS: Nasal superior
NT: Neuropil thread
OD: Optic disc
OLM: Outer limiting membrane
ONL: Outer nuclear layer
OPL: Outer plexiform layer
OR: Outer retina
P. Ctx: Parietal cortex
PET: Positron emission tomography
PRPH2: Peripherin-2
PMI: Postmortem interval
PV: Primary visual cortex
pTau: Hyperphosphorylated tau
PO: Propylene oxide
(r): Retinal
RGC: Retinal ganglion cell
RHO: Rhodopsin
S100β: S100 calcium-binding protein B
SAS: Statistical analysis system
scFv: Single-chain Fv fragment
SD: Standard deviation
SEM: Standard error of the mean
ST: Superior-temporal
T^L/H^: Low/high tauopathy
T.Ctx or Temp: Temporal cortex
TEM: Transmission electron microscopy
TMT: Tandem mass tag
UDS: Unified data set
VA: Visual association cortex

## References

[1] Alzheimer's Association (2022). 2022 Alzheimer's disease facts and figures. Alzheimers Dement.

[2] Akiyama H, Barger S, Barnum S, Bradt B, Bauer J, Cole GM, Cooper NR, Eikelenboom P, Emmerling M, Fiebich BL (2000). Inflammation and Alzheimer's disease. Neurobiol Aging.

[3] Armstrong GW, Kim LA, Vingopoulos F, Park JY, Garg I, Kasetty M, Silverman RF, Zeng R, Douglas VP, Lopera F (2021). Retinal imaging findings in carriers with PSEN1-associated early-onset familial Alzheimer disease before onset of cognitive symptoms. JAMA Ophthalmol.

[4] Asanad S, Fantini M, Sultan W, Nassisi M, Felix CM, Wu J, Karanjia R, Ross-Cisneros FN, Sagare AP, Zlokovic BV (2020). Retinal nerve fiber layer thickness predicts CSF amyloid/tau before cognitive decline. PLoS One.

[5] Asanad S, Ross-Cisneros FN, Nassisi M, Barron E, Karanjia R, Sadun AA (2019). The retina in Alzheimer's disease: histomorphometric analysis of an ophthalmologic biomarker. Invest Ophthalmol Vis Sci.

[6] Ayyubova G (2022). Dysfunctional microglia and tau pathology in Alzheimer's disease. Rev Neurosci.

[7] Bai B, Wang X, Li Y, Chen PC, Yu K, Dey KK, Yarbro JM, Han X, Lutz BM, Rao S (2020). Deep multilayer brain proteomics identifies molecular networks in Alzheimer's disease progression. Neuron.

[8] Baker-Nigh A, Vahedi S, Davis EG, Weintraub S, Bigio EH, Klein WL, Geula C (2015). Neuronal amyloid-β accumulation within cholinergic basal forebrain in ageing and Alzheimer's disease. Brain.

[9] Besser L, Kukull W, Knopman DS, Chui H, Galasko D, Weintraub S, Jicha G, Carlsson C, Burns J, Quinn J (2018). Version 3 of the national Alzheimer's coordinating center's uniform data set. Alzheimer Dis Assoc Disord.

[10] Bettcher BM, Tansey MG, Dorothee G, Heneka MT (2021). Peripheral and central immune system crosstalk in Alzheimer disease-a research prospectus. Nat Rev Neurol.

[11] Blanks JC, Hinton DR, Sadun AA, Miller CA (1989). Retinal ganglion cell degeneration in Alzheimer's disease. Brain Res.

[12] Blanks JC, Schmidt SY, Torigoe Y, Porrello KV, Hinton DR, Blanks RH (1996). Retinal pathology in Alzheimer's disease. II. Regional neuron loss and glial changes in GCL. Neurobiol Aging.

[13] Braak H, Alafuzoff I, Arzberger T, Kretzschmar H, Del Tredici K (2006). Staging of Alzheimer disease-associated neurofibrillary pathology using paraffin sections and immunocytochemistry. Acta Neuropathol.

[14] Bringmann A, Reichenbach A (2001). Role of Muller cells in retinal degenerations. Front Biosci.

[15] Butovsky O, Koronyo-Hamaoui M, Kunis G, Ophir E, Landa G, Cohen H, Schwartz M (2006). Glatiramer acetate fights against Alzheimer's disease by inducing dendritic-like microglia expressing insulin-like growth factor 1. Proc Natl Acad Sci U S A.

[16] Byun MS, Park SW, Lee JH, Yi D, Jeon SY, Choi HJ, Joung H, Ghim UH, Park UC, Kim YK (2021). Association of retinal changes with Alzheimer disease neuroimaging biomarkers in cognitively normal individuals. JAMA Ophthalmol.

[17] Cagnin A, Brooks DJ, Kennedy AM, Gunn RN, Myers R, Turkheimer FE, Jones T, Banati RB (2001). In-vivo measurement of activated microglia in dementia. Lancet.

[18] Cao KJ, Kim JH, Kroeger H, Gaffney PM, Lin JH, Sigurdson CJ, Yang J (2021). ARCAM-1 facilitates fluorescence detection of amyloid-containing deposits in the retina. Transl Vis Sci Technol.

[19] Coppola G, Di Renzo A, Ziccardi L, Martelli F, Fadda A, Manni G, Barboni P, Pierelli F, Sadun AA, Parisi V (2015). Optical coherence tomography in alzheimer's disease: a meta-analysis. PLoS One.

[20] Crair MC, Mason CA (2016). Reconnecting eye to brain. J Neurosci.

[21] Cristóvão JS, Gomes CM (2019). S100 proteins in Alzheimer's disease. Front Neurosci.

[22] den Haan J, Morrema THJ, Verbraak FD, de Boer JF, Scheltens P, Rozemuller AJ, Bergen AAB, Bouwman FH, Hoozemans JJ (2018). Amyloid-beta and phosphorylated tau in post-mortem Alzheimer’s disease retinas. Acta Neuropathol Commun.

[23] Doustar J, Torbati T, Black KL, Koronyo Y, Koronyo-Hamaoui M (2017). Optical coherence tomography in Alzheimer's disease and other neurodegenerative diseases. Front Neurol.

[24] Du X, Koronyo Y, Mirzaei N, Yang C, Fuchs DT, Black KL, Koronyo-Hamaoui M, Gao L (2022). Label-free hyperspectral imaging and deep-learning prediction of retinal amyloid beta-protein and phosphorylated tau. PNAS Nexus.

[25] Duarte-Guterman P, Albert AY, Inkster AM, Barha CK, Galea LAM, Alzheimer's Disease Neuroimaging Initiative (2020). Inflammation in Alzheimer's disease: do sex and APOE matter?. J Alzheimers Dis.

[26] Dubois B, Chupin M, Hampel H, Lista S, Cavedo E, Croisile B, Louis Tisserand G, Touchon J, Bonafe A, Ousset PJ (2015). Donepezil decreases annual rate of hippocampal atrophy in suspected prodromal Alzheimer's disease. Alzheimers Dement.

[27] Dumitrascu OM, Lyden PD, Torbati T, Sheyn J, Sherzai A, Sherzai D, Sherman DS, Rosenberry R, Cheng S, Johnson KO (2020). Sectoral segmentation of retinal amyloid imaging in subjects with cognitive decline. Alzheimers Dement (Amst).

[28] Dumitrascu OM, Rosenberry R, Sherman DS, Khansari MM, Sheyn J, Torbati T, Sherzai A, Sherzai D, Johnson KO, Czeszynski AD (2021). Retinal venular tortuosity jointly with retinal amyloid burden correlates with verbal memory loss: a pilot study. Cells.

[29] Edison P, Archer HA, Gerhard A, Hinz R, Pavese N, Turkheimer FE, Hammers A, Tai YF, Fox N, Kennedy A (2008). Microglia, amyloid, and cognition in Alzheimer's disease: An [11C](R)PK11195-PET and [11C]PIB-PET study. Neurobiol Dis.

[30] Erskine L, Herrera E (2014). Connecting the retina to the brain. ASN Neuro.

[31] Folstein MF, Folstein SE, McHugh PR (1975). "Mini-mental state". A practical method for grading the cognitive state of patients for the clinician. J Psychiatr Res.

[32] Frost GR, Li YM (2017). The role of astrocytes in amyloid production and Alzheimer's disease. Open Biol.

[33] Golzan SM, Goozee K, Georgevsky D, Avolio A, Chatterjee P, Shen K, Gupta V, Chung R, Savage G, Orr CF (2017). Retinal vascular and structural changes are associated with amyloid burden in the elderly: ophthalmic biomarkers of preclinical Alzheimer's disease. Alzheimers Res Ther.

[34] Grimaldi A, Pediconi N, Oieni F, Pizzarelli R, Rosito M, Giubettini M, Santini T, Limatola C, Ruocco G, Ragozzino D (2019). Neuroinflammatory processes, A1 astrocyte activation and protein aggregation in the retina of Alzheimer's disease patients, possible biomarkers for early diagnosis. Front Neurosci.

[35] Grubman A, Choo XY, Chew G, Ouyang JF, Sun G, Croft NP, Rossello FJ, Simmons R, Buckberry S, Landin DV (2021). Transcriptional signature in microglia associated with Abeta plaque phagocytosis. Nat Commun.

[36] Habiba U, Descallar J, Kreilaus F, Adhikari UK, Kumar S, Morley JW, Bui BV, Hamaoui MK, Tayebi M (2021). Detection of retinal and blood Aβ oligomers with nanobodies. Alzheimers Dement (Amst).

[37] Habiba U, Merlin S, Lim JKH, Wong VHY, Nguyen CTO, Morley JW, Bui BV, Tayebi M (2020). Age-specific retinal and cerebral immunodetection of amyloid-beta plaques and oligomers in a rodent model of Alzheimer's disease. J Alzheimers Dis.

[38] Hadoux X, Hui F, Lim JKH, Masters CL, Pebay A, Chevalier S, Ha J, Loi S, Fowler CJ, Rowe C (2019). Non-invasive in vivo hyperspectral imaging of the retina for potential biomarker use in Alzheimer's disease. Nat Commun.

[39] Hart de Ruyter FJ, Morrema THJ, den Haan J, Twisk JWR, de Boer JF, Scheltens P, Boon BDC, Thal DR, Rozemuller AJ, Netherlands Brain Bank (2022). Phosphorylated tau in the retina correlates with tau pathology in the brain in Alzheimer's disease and primary tauopathies. Acta Neuropathol.

[40] Hart NJ, Koronyo Y, Black KL, Koronyo-Hamaoui M (2016). Ocular indicators of Alzheimer's: exploring disease in the retina. Acta Neuropathol.

[41] Hayden EY, Teplow DB (2013). Amyloid beta-protein oligomers and Alzheimer's disease. Alzheimers Res Ther.

[42] Heneka MT, Carson MJ, El Khoury J, Landreth GE, Brosseron F, Feinstein DL, Jacobs AH, Wyss-Coray T, Vitorica J, Ransohoff RM (2015). Neuroinflammation in Alzheimer's disease. Lancet Neurol.

[43] Hickman SE, Allison EK, El Khoury J (2008). Microglial dysfunction and defective beta-amyloid clearance pathways in aging Alzheimer's disease mice. J Neurosci.

[44] Higginbotham L, Ping L, Dammer EB, Duong DM, Zhou M, Gearing M, Hurst C, Glass JD, Factor SA, Johnson ECB (2020). Integrated proteomics reveals brain-based cerebrospinal fluid biomarkers in asymptomatic and symptomatic Alzheimer's disease. Sci Adv.

[45] Hong S, Beja-Glasser VF, Nfonoyim BM, Frouin A, Li S, Ramakrishnan S, Merry KM, Shi Q, Rosenthal A, Barres BA (2016). Complement and microglia mediate early synapse loss in Alzheimer mouse models. Science (New York, NY).

[46] Hong S, Dissing-Olesen L, Stevens B (2016). New insights on the role of microglia in synaptic pruning in health and disease. Curr Opin Neurobiol.

[47] Hyman BT, Phelps CH, Beach TG, Bigio EH, Cairns NJ, Carrillo MC, Dickson DW, Duyckaerts C, Frosch MP, Masliah E (2012). National institute on aging-Alzheimer's association guidelines for the neuropathologic assessment of Alzheimer's disease. Alzheimers Dement.

[48] Jack CR, Bennett DA, Blennow K, Carrillo MC, Dunn B, Haeberlein SB, Holtzman DM, Jagust W, Jessen F, Karlawish J (2018). NIA-AA research framework: toward a biological definition of Alzheimer's disease. Alzheimers Dement.

[49] Jack CR, Bennett DA, Blennow K, Carrillo MC, Feldman HH, Frisoni GB, Hampel H, Jagust WJ, Johnson KA, Knopman DS (2016). A/T/N: an unbiased descriptive classification scheme for Alzheimer disease biomarkers. Neurology.

[50] Jonas RA, Wang YX, Yang H, Li JJ, Xu L, Panda-Jonas S, Jonas JB (2015). Optic disc-fovea distance, axial length and parapapillary zones. The Beijing eye study 2011. PLoS One.

[51] Kamphuis W, Middeldorp J, Kooijman L, Sluijs JA, Kooi EJ, Moeton M, Freriks M, Mizee MR, Hol EM (2014). Glial fibrillary acidic protein isoform expression in plaque related astrogliosis in Alzheimer's disease. Neurobiol Aging.

[52] Khan TK, Alkon DL (2015). Alzheimer's disease cerebrospinal fluid and neuroimaging biomarkers: diagnostic accuracy and relationship to drug efficacy. J Alzheimers Dis.

[53] Kirbas S, Turkyilmaz K, Anlar O, Tufekci A, Durmus M (2013). Retinal nerve fiber layer thickness in patients with Alzheimer disease. J Neuroophthalmol.

[54] Kobro-Flatmoen A, Lagartos-Donate MJ, Aman Y, Edison P, Witter MP, Fang EF (2021). Re-emphasizing early Alzheimer's disease pathology starting in select entorhinal neurons, with a special focus on mitophagy. Ageing Res Rev.

[55] Koenigsknecht J, Landreth G (2004). Microglial phagocytosis of fibrillar beta-amyloid through a beta1 integrin-dependent mechanism. J Neurosci.

[56] Koffie RM, Hyman BT, Spires-Jones TL (2011). Alzheimer's disease: synapses gone cold. Mol Neurodegener.

[57] Koronyo Y, Biggs D, Barron E, Boyer DS, Pearlman JA, Au WJ, Kile SJ, Blanco A, Fuchs DT, Ashfaq A (2017). Retinal amyloid pathology and proof-of-concept imaging trial in Alzheimer's disease. JCI Insight.

[58] Koronyo Y, Salumbides BC, Sheyn J, Pelissier L, Li S, Ljubimov V, Moyseyev M, Daley D, Fuchs DT, Pham M (2015). Therapeutic effects of glatiramer acetate and grafted CD115^+^ monocytes in a mouse model of Alzheimer's disease. Brain.

[59] Koronyo-Hamaoui M, Koronyo Y, Ljubimov AV, Miller CA, Ko MK, Black KL, Schwartz M, Farkas DL (2011). Identification of amyloid plaques in retinas from Alzheimer's patients and noninvasive in vivo optical imaging of retinal plaques in a mouse model. Neuroimage.

[60] Koronyo-Hamaoui M, Sheyn J, Hayden EY, Li S, Fuchs DT, Regis GC, Lopes DHJ, Black KL, Bernstein KE, Teplow DB (2020). Peripherally derived angiotensin converting enzyme-enhanced macrophages alleviate Alzheimer-related disease. Brain.

[61] Kromer R, Serbecic N, Hausner L, Froelich L, Aboul-Enein F, Beutelspacher SC (2014). Detection of retinal nerve fiber layer defects in Alzheimer's disease using SD-OCT. Front Psychiatry.

[62] La Morgia C, Ross-Cisneros FN, Koronyo Y, Hannibal J, Gallassi R, Cantalupo G, Sambati L, Pan BX, Tozer KR, Barboni P (2016). Melanopsin retinal ganglion cell loss in Alzheimer disease. Ann Neurol.

[63] Lai-Tim Y, Mugnier L, Krafft L, Chen A, Petit C, Mecê P, Grieve K, Paques M, Meimon S (2020). Super-resolution in vivo retinal imaging using structured illumination ophthalmoscopy. arXiv.

[64] Lambert MP, Barlow AK, Chromy BA, Edwards C, Freed R, Liosatos M, Morgan TE, Rozovsky I, Trommer B, Viola KL (1998). Diffusible, nonfibrillar ligands derived from Abeta1-42 are potent central nervous system neurotoxins. Proc Natl Acad Sci U S A.

[65] Lee S, Jiang K, McIlmoyle B, To E, Xu QA, Hirsch-Reinshagen V, Mackenzie IR, Hsiung GR, Eadie BD, Sarunic MV (2020). Amyloid beta immunoreactivity in the retinal ganglion cell layer of the Alzheimer's eye. Front Neurosci.

[66] Lemmens S, Van Craenendonck T, Van Eijgen J, De Groef L, Bruffaerts R, de Jesus DA, Charle W, Jayapala M, Sunaric-Mégevand G, Standaert A (2020). Combination of snapshot hyperspectral retinal imaging and optical coherence tomography to identify Alzheimer's disease patients. Alzheimers Res Ther.

[67] Leng F, Edison P (2021). Neuroinflammation and microglial activation in Alzheimer disease: where do we go from here?. Nat Rev Neurol.

[68] Li S, Hayden EY, Garcia VJ, Fuchs D-T, Sheyn J, Daley DA, Rentsendorj A, Torbati T, Black KL, Rutishauser U (2020). Activated bone marrow-derived macrophages eradicate Alzheimer's-related Aβ42 oligomers and protect synapses. Front Immunol.

[69] Liddelow SA, Guttenplan KA, Clarke LE, Bennett FC, Bohlen CJ, Schirmer L, Bennett ML, Münch AE, Chung WS, Peterson TC (2017). Neurotoxic reactive astrocytes are induced by activated microglia. Nature.

[70] Lucey BP, Wisch J, Boerwinkle AH, Landsness EC, Toedebusch CD, McLeland JS, Butt OH, Hassenstab J, Morris JC, Ances BM (2021). Sleep and longitudinal cognitive performance in preclinical and early symptomatic Alzheimer's disease. Brain.

[71] Mandrekar S, Jiang Q, Lee CY, Koenigsknecht-Talboo J, Holtzman DM, Landreth GE (2009). Microglia mediate the clearance of soluble Abeta through fluid phase macropinocytosis. J Neurosci.

[72] Marshak DR, Pesce SA, Stanley LC, Griffin WS (1992). Increased S100 beta neurotrophic activity in Alzheimer's disease temporal lobe. Neurobiol Aging.

[73] McGeer PL, Itagaki S, Tago H, McGeer EG (1987). Reactive microglia in patients with senile dementia of the Alzheimer type are positive for the histocompatibility glycoprotein HLA-DR. Neurosci Lett.

[74] Meli G, Lecci A, Manca A, Krako N, Albertini V, Benussi L, Ghidoni R, Cattaneo A (2014). Conformational targeting of intracellular Abeta oligomers demonstrates their pathological oligomerization inside the endoplasmic reticulum. Nat Commun.

[75] Meli G, Visintin M, Cannistraci I, Cattaneo A (2009). Direct in vivo intracellular selection of conformation-sensitive antibody domains targeting Alzheimer's amyloid-beta oligomers. J Mol Biol.

[76] Mirra SS, Heyman A, McKeel D, Sumi SM, Crain BJ, Brownlee LM, Vogel FS, Hughes JP, van Belle G, Berg L (1991). The consortium to establish a registry for Alzheimer's disease (CERAD). Part II. Standardization of the neuropathologic assessment of Alzheimer's disease. Neurology.

[77] Mirzaei N, Shi H, Oviatt M, Doustar J, Rentsendorj A, Fuchs DT, Sheyn J, Black KL, Koronyo Y, Koronyo-Hamaoui M (2020). Alzheimer's retinopathy: seeing disease in the eyes. Front Neurosci.

[78] Moloney CM, Lowe VJ, Murray ME (2021). Visualization of neurofibrillary tangle maturity in Alzheimer's disease: a clinicopathologic perspective for biomarker research. Alzheimers Dement.

[79] Montine TJ, Phelps CH, Beach TG, Bigio EH, Cairns NJ, Dickson DW, Duyckaerts C, Frosch MP, Masliah E, Mirra SS (2012). National institute on aging-Alzheimer's association guidelines for the neuropathologic assessment of Alzheimer's disease: a practical approach. Acta Neuropathol.

[80] More SS, Beach JM, McClelland C, Mokhtarzadeh A, Vince R (2019). In vivo assessment of retinal biomarkers by hyperspectral imaging: early detection of Alzheimer's disease. ACS Chem Neurosci.

[81] Morris JC (1993). The clinical dementia rating (CDR): current version and scoring rules. Neurology.

[82] Murrell J, Ghetti B, Cochran E, Macias-Islas MA, Medina L, Varpetian A, Cummings JL, Mendez MF, Kawas C, Chui H (2006). The A431E mutation in PSEN1 causing familial Alzheimer's disease originating in Jalisco State, Mexico: an additional fifteen families. Neurogenetics.

[83] Ngolab J, Donohue M, Belsha A, Salazar J, Cohen P, Jaiswal S, Tan V, Gessert D, Korouri S, Aggarwal NT (2021). Feasibility study for detection of retinal amyloid in clinical trials: the anti-amyloid treatment in asymptomatic Alzheimer's disease (A4) trial. Alzheimers Dement (Amst).

[84] O’Bryhim B, Apte RS, Kung N, Coble D, Van Stavern GP (2018). Association of preclinical Alzheimer disease with optical coherence tomographic angiography findings. JAMA Ophthalmol.

[85] Patton N, Aslam T, Macgillivray T, Pattie A, Deary IJ, Dhillon B (2005). Retinal vascular image analysis as a potential screening tool for cerebrovascular disease: a rationale based on homology between cerebral and retinal microvasculatures. J Anat.

[86] Perez-Nievas BG, Serrano-Pozo A (2018). Deciphering the astrocyte reaction in Alzheimer's disease. Front Aging Neurosci.

[87] Purves D, Augustine GJ, Fitzpatrick D, Katz LC, LaMantia A-S, McNamara JO, Williams SM (2018). Neuroscience.

[88] Qiu Y, Jin T, Mason E, Campbell MCW (2020). Predicting thioflavin fluorescence of retinal amyloid deposits associated with Alzheimer's disease from their polarimetric properties. Transl Vis Sci Technol.

[89] Risacher SL, WuDunn D, Tallman EF, West JD, Gao S, Farlow MR, Brosch JR, Apostolova LG, Saykin AJ (2020). Visual contrast sensitivity is associated with the presence of cerebral amyloid and tau deposition. Brain Communications.

[90] Rogaev EI, Sherrington R, Rogaeva EA, Levesque G, Ikeda M, Liang Y, Chi H, Lin C, Holman K, Tsuda T (1995). Familial Alzheimer's disease in kindreds with missense mutations in a gene on chromosome 1 related to the Alzheimer's disease type 3 gene. Nature.

[91] Rohrschneider K (2004). Determination of the location of the fovea on the fundus. Invest Ophthalmol Vis Sci.

[92] Rossetti HC, Munro Cullum C, Hynan LS, Lacritz LH (2010). The CERAD neuropsychologic battery total score and the progression of Alzheimer disease. Alzheimer Dis Assoc Disord.

[93] Sadun AA, Borchert M, DeVita E, Hinton DR, Bassi CJ (1987). Assessment of visual impairment in patients with Alzheimer's disease. Am J Ophthalmol.

[94] Sathe G, Albert M, Darrow J, Saito A, Troncoso J, Pandey A, Moghekar A (2021). Quantitative proteomic analysis of the frontal cortex in Alzheimer's disease. J Neurochem.

[95] Schon C, Hoffmann NA, Ochs SM, Burgold S, Filser S, Steinbach S, Seeliger MW, Arzberger T, Goedert M, Kretzschmar HA (2012). Long-term in vivo imaging of fibrillar tau in the retina of P301S transgenic mice. PLoS One.

[96] Schultz N, Byman E, Wennström M (2020). Levels of retinal amyloid-β correlate with levels of retinal IAPP and hippocampal amyloid-β in neuropathologically evaluated individuals. J Alzheimers Dis.

[97] Scopa C, Marrocco F, Latina V, Ruggeri F, Corvaglia V, La Regina F, Ammassari-Teule M, Middei S, Amadoro G, Meli G (2020). Impaired adult neurogenesis is an early event in Alzheimer’s disease neurodegeneration, mediated by intracellular Aβ oligomers. Cell Death Differ.

[98] Selkoe DJ (2008). Soluble oligomers of the amyloid beta-protein impair synaptic plasticity and behavior. Behav Brain Res.

[99] Shankar GM, Li S, Mehta TH, Garcia-Munoz A, Shepardson NE, Smith I, Brett FM, Farrell MA, Rowan MJ, Lemere CA (2008). Amyloid-beta protein dimers isolated directly from Alzheimer's brains impair synaptic plasticity and memory. Nat Med.

[100] Shi H, Koronyo Y, Rentsendorj A, Fuchs DT, Sheyn J, Black KL, Mirzaei N, Koronyo-Hamaoui M (2021). Retinal vasculopathy in Alzheimer's disease. Front Neurosci.

[101] Shi H, Koronyo Y, Rentsendorj A, Regis GC, Sheyn J, Fuchs DT, Kramerov AA, Ljubimov AV, Dumitrascu OM, Rodriguez AR (2020). Identification of early pericyte loss and vascular amyloidosis in Alzheimer's disease retina. Acta Neuropathol.

[102] Shi H, Yin Z, Koronyo Y, Fuchs DT, Sheyn J, Davis MR, Wilson JW, Margeta MA, Pitts KM, Herron S (2022). Regulating microglial miR-155 transcriptional phenotype alleviates Alzheimer's-induced retinal vasculopathy by limiting Clec7a/Galectin-3(+) neurodegenerative microglia. Acta Neuropathol Commun.

[103] Shi J, Zhao Y, Wang K, Shi X, Wang Y, Huang H, Zhuang Y, Cai T, Wang F, Shao F (2015). Cleavage of GSDMD by inflammatory caspases determines pyroptotic cell death. Nature.

[104] Singer MB, Ringman JM, Chu Z, Zhou X, Jiang X, Shahidzadeh A, Wang RK, Kashani AH (2021). Abnormal retinal capillary blood flow in autosomal dominant Alzheimer's disease. Alzheimers Dement (Amst).

[105] Snyder PJ, Alber J, Alt C, Bain LJ, Bouma BE, Bouwman FH, DeBuc DC, Campbell MCW, Carrillo MC, Chew EY (2021). Retinal imaging in Alzheimer's and neurodegenerative diseases. Alzheimers Dement.

[106] Snyder PJ, Johnson LN, Lim YY, Santos CY, Alber J, Maruff P, Fernandez B (2016). Nonvascular retinal imaging markers of preclinical Alzheimer's disease. Alzheimers Dement (Amst).

[107] Sperling RA, Donohue MC, Raman R, Sun CK, Yaari R, Holdridge K, Siemers E, Johnson KA, Aisen PS (2020). Association of factors with elevated amyloid burden in clinically normal older individuals. JAMA Neurol.

[108] Tadokoro K, Yamashita T, Kimura S, Nomura E, Ohta Y, Omote Y, Takemoto M, Hishikawa N, Morihara R, Morizane Y (2021). Retinal amyloid imaging for screening Alzheimer's disease. J Alzheimers Dis.

[109] Thal DR, Rüb U, Orantes M, Braak H (2002). Phases of A beta-deposition in the human brain and its relevance for the development of AD. Neurology.

[110] Tu S, Okamoto S, Lipton SA, Xu H (2014). Oligomeric Aβ-induced synaptic dysfunction in Alzheimer's disease. Mol Neurodegener.

[111] Uchihara T (2007). Silver diagnosis in neuropathology: principles, practice and revised interpretation. Acta Neuropathol.

[112] Vecino E, Rodriguez FD, Ruzafa N, Pereiro X, Sharma SC (2016). Glia-neuron interactions in the mammalian retina. Prog Retin Eye Res.

[113] Vit JP, Fuchs DT, Angel A, Levy A, Lamensdorf I, Black KL, Koronyo Y, Koronyo-Hamaoui M (2021). Color and contrast vision in mouse models of aging and Alzheimer's disease using a novel visual-stimuli four-arm maze. Sci Rep.

[114] Wang M, Beckmann ND, Roussos P, Wang E, Zhou X, Wang Q, Ming C, Neff R, Ma W, Fullard JF (2018). The Mount Sinai cohort of large-scale genomic, transcriptomic and proteomic data in Alzheimer's disease. Sci Data.

[115] Wang W, Zhao F, Ma X, Perry G, Zhu X (2020). Mitochondria dysfunction in the pathogenesis of Alzheimer's disease: recent advances. Mol Neurodegener.

[116] Winer JR, Deters KD, Kennedy G, Jin M, Goldstein-Piekarski A, Poston KL, Mormino EC (2021). Association of short and long sleep duration with amyloid-beta burden and cognition in aging. JAMA Neurol.

[117] Wyss-Coray T, Rogers J (2012). Inflammation in Alzheimer disease-a brief review of the basic science and clinical literature. Cold Spring Harb Perspect Med.

[118] Xie F, Luo W, Zhang Z, Sun D (2012). In vivo molecular imaging in retinal disease. J Ophthalmol.

[119] Xu QA, Boerkoel P, Hirsch-Reinshagen V, Mackenzie IR, Hsiung GR, Charm G, To EF, Liu AQ, Schwab K, Jiang K (2022). Muller cell degeneration and microglial dysfunction in the Alzheimer's retina. Acta Neuropathol Commun.

[120] Yasuda Y, Tateishi N, Shimoda T, Satoh S, Ogitani E, Fujita S (2004). Relationship between S100beta and GFAP expression in astrocytes during infarction and glial scar formation after mild transient ischemia. Brain Res.

[121] Zuroff L, Daley D, Black KL, Koronyo-Hamaoui M (2017). Clearance of cerebral Abeta in Alzheimer's disease: reassessing the role of microglia and monocytes. Cell Mol Life Sci.

