# Supplementary material for: Retinal pathological features and proteome signatures of Alzheimer’s disease
Source: Acta Neuropathol. 2023 Feb 11;145(4):409–38. doi: 10.1007/s00401-023-02548-2 (PMC10020290; doi:10.1007/s00401-023-02548-2)
Supplement: Supplementary file 1 — Supplementary file1 (PDF 10451 KB) [file 401_2023_2548_MOESM1_ESM.pdf]

Supplementary file

Retinal Pathological Features and Proteome Signatures of Alzheimer's Disease

Acta Neuropathologica

Yosef Koronyo, MSc, Altan Rentsendorj, PhD, Nazanin Mirzaei, PhD, Giovanna C. Regis, BSc, Julia Sheyn, BSc, Haoshen Shi, PhD, Ernesto Barron, MSc, Galen Cook-Wiens, MSc, Anthony R. Rodriguez, BSc, Rodrigo Medeiros, PhD, Joao A. Paulo, PhD, Veer B. Gupta, PhD, Andrei A. Kramerov, MD, PhD, Alexander V. Ljubimov, PhD, Jennifer E. Van Eyk, PhD, Stuart L. Graham, MD, Vivek K. Gupta, PhD, John M. Ringman, MD, David R. Hinton, MD, PhD, Carol A. Miller, MD, Keith L. Black, MD, Antonino Cattaneo, MD, Giovanni Meli, PhD, Mehdi Mirzaei, PhD, Dieu-Trang Fuchs, MSc, and Maya Koronyo-Hamaoui, PhD

Corresponding author: Maya Koronyo-Hamaoui, PhD, Cedars-Sinai Medical Center, 127 S. San Vicente Blvd., Los Angeles, CA, USA 90048. E-mail: maya.koronyo@csmc.edu

Supplementary Table 1. List of human donors whose postmortem retinas and paired brains were used for histology and proteins.
Supplementary Table 2. List of human donors whose postmortem brains were used for mass spectrometry.
Supplementary Table 3. List of antibodies for immunohistochemistry.
Supplementary Fig. 1. Brain NFT and NT severity scores assigned based on Gallyas silver stain and immunofluorescence of retinal $A\beta_{42}$ and associated gliosis per subregion in human donors.
Supplementary Fig. 2. Extended data on retinal $A\beta_{42}$ deposition in relation to sex, age, and PMI in MCI and AD patients compared to NC controls.
Supplementary Fig. 3. Extended TEM analysis of retinal $A\beta_{42}$ deposits in AD patients.
Supplementary Fig. 4. Spatial distribution of $A\beta_{42}$ burden in retinal subregions and correlations with various brain regions and cognition.
Supplementary Fig. 5. Extended data on the intracellular scFvA13^{+}-Aβ oligomers (AβOi) distribution in the retinas of MCI and AD patients compared to retinas of NC controls and correlations with cerebral pathology and cognition.
Supplementary Fig. 6. Extended data on retinal S100β^{+} and GFAP^{+} macrogliosis distribution in MCI and AD patients and correlations to cerebral pathology and cognition.
Supplementary Fig. 7. Extended data on spatial distribution of retinal IBA1^{+} microgliosis and correlations with retina and brain pathology and cognition.
Supplementary Fig. 8. Extended data on retinal atrophy and correlations with other cerebral biomarkers.
Supplementary Fig. 9 Extended global proteomics data in retinas and brains from AD patients and NC controls.
Supplementary Table 4. Extended mass spectrometry data on significantly up-regulated proteins in the retina of Alzheimer's patients compared to cognitively normal controls.
Supplementary Table 5. Extended mass spectrometry data on significantly down-regulated proteins in the retina of Alzheimer's patients compared to cognitively normal controls.
Supplementary Table 6. Extended mass spectrometry data on significantly up-regulated proteins in the temporal cortex of Alzheimer's patients compared to cognitively normal controls.
Supplementary Table 7. Extended mass spectrometry data on significantly down-regulated proteins in the temporal cortex of Alzheimer's patients compared to cognitively normal controls
Supplementary Table 8. Extended mass spectrometry data on significantly up-regulated proteins in the hippocampus of Alzheimer's patients compared to cognitively normal controls.
Supplementary Table 9. Extended mass spectrometry data on significantly down-regulated proteins in the hippocampus of Alzheimer's patients compared to cognitively normal controls.
Supplementary Table 10. Extended mass spectrometry data on significantly up-regulated proteins in the cerebellum of Alzheimer's patients compared to cognitively normal controls.
Supplementary Table 11. Extended mass spectrometry data on significantly down-regulated proteins in the cerebellum of Alzheimer's patients compared to cognitively normal controls.
Supplementary Table 12. Common upregulated DEPs with previous brain datasets.
Supplementary Table 13. Common downregulated DEPs with previous brain datasets.

Supplementary Table 1. List of human donors whose postmortem retinas and paired brains were used for histology and proteins.

Retinas	Diagnosis	Sex	Race	Age at death	Thal-A	Braak-B	CERAD-C	CAA	Co-morbid. [LB/ASVD]	Braak Stage	Braak Stage average	CDR score	MMSE score	APOE status	Study type
1	AD	F	W	63	3	3	3	0	−/+	V	5	n.a.	9	n.a.	His
2	AD	F	W	90	2	3	3	0	−/+	IV	4	n.a.	16	n.a.	His
3	AD	F	B	94	3	3	3	II	−/+	V	5	n.a.	n.a.	n.a.	His
4	AD	F	A	89	2	3	2	II	−/+	V-VI	5.5	n.a.	6	n.a.	His
5	AD	F	W	64	3	3	2	0	−/+	VI	6	3	5	n.a.	His
6	AD	F	W	90	2	3	3	II	−/+	V	5	2	9	n.a.	His
7	AD	F	A	93	3	2	2	0	−/+	IV	4	3	n.a.	e3/e3	His
8	AD	F	W	81	3	3	3	II	−/+	VI	6	n.a.	4	n.a.	His
9	AD	F	W	65	3	3	3	II	−/+	V-VI	5.5	n.a.	n.a.	n.a.	His
10	AD	F	B	90	3	3	3	II	−/+	V-VI	5.5	n.a.	n.a.	n.a.	His
11	AD	F	W	97	2	3	2	0	−/+	V	5	n.a.	4	n.a.	His
12	AD	F	W	86	3	3	2	I	−/+	V-VI	5.5	n.a.	n.a.	n.a.	His
13	AD	M	W	85	3	2	3	I	−/+	IV	4	n.a.	n.a.	n.a.	His
14	AD	M	W	83	3	2	3	II	−/+	IV	4	n.a.	15	n.a.	His
15	AD	M	W	88	2	2	2	0	−/+	III-IV	3.5	n.a.	4	n.a.	His
16	AD	M	W	75	2	2	2	0	−/+	III-IV	3.5	n.a.	4	n.a.	His
17	AD	M	W	71	3	3	3	II	−/n.a.	VI	6	n.a.	10	n.a.	His
18	AD	M	W	90	3	3	2	II	−/+	V	5	2	18	n.a.	His
19	AD	M	W	66	3	3	3	I-II	−/+	V	5	3	n.a.	n.a.	His
20	AD/AD	M	A	40	3	2	3	I	−/−	III-IV	3.5	3	n.a.	e3/e3	His
21	AD*	M	W	85	n.a.	n.a.	n.a.	n.a.	−/n.a.	n.a.	n.a.	n.a.	n.a.	n.a.	His
22	AD*	M	W	79	n.a.	n.a.	n.a.	n.a.	n.a./+	n.a.	n.a.	n.a.	n.a.	n.a.	His
23	AD*	M	W	59	n.a.	n.a.	n.a.	n.a.	n.a./+	n.a.	n.a.	n.a.	n.a.	n.a.	His
24	AD	M	W	88	3	3	3	I-II	−/+	V-VI	5.5	n.a.	n.a.	n.a.	His
25	MCI	F	W	91	2	2	2	0	−/+	III-IV	3.5	n.a.	29	n.a.	His
26	MCI	F	W	80	3	3	3	I	variant/n.a.	V	5	n.a.	n.a.	n.a.	His
27	MCI	F	W	88	3	3	2	II	−/+	V	5	2	15	n.a.	His
28	MCI	F	W	76	3	3	3	II	−/+	V	5	n.a.	26	e3/e4	His
29	MCI	F	W	93	3	2	2	II	−/+	IV	4	3	n.a.	e3/e3	His
30	MCI	F	W	86	3	1	3	0	−/−	I-II	1.5	0	26	e3/e4	His
31	MCI	F	W	87	3	3	3	I-II	−/+	V-VI	5.5	3	n.a.	e3/e3	His
32	MCI	M	W	83	2	3	2	I-II	−/+	III-IV	3.5	n.a.	28	n.a.	His
33	MCI	M	W	93	2	0	2	0	−/+	0	0	3	n.a.	n.a.	His
34	MCI	M	H	80	3	3	2	I	−/+	V	5	3	n.a.	e3/e3	His

35	MCI	M	W	97	2	3	3	I	−/+	V	5	n.a.	n.a.	e3/e3	His
36	NC*	F	W	71	n.a.	n.a.	n.a.	n.a.	n.a.	n.a.	n.a.	n.a.	n.a.	n.a.	His
37	NC*	F	W	86	n.a.	n.a.	n.a.	n.a.	n.a.	n.a.	n.a.	n.a.	n.a.	n.a.	His
38	NC	F	W	92	1	1	1	I	−/n.a.	I-II	1.5	n.a.	25	n.a.	His
39	NC*	F	W	70	n.a.	n.a.	n.a.	n.a.	n.a.	n.a.	n.a.	n.a.	n.a.	n.a.	His
40	NC	F	W	95	3	3	2	I	−/+	V	5	0	30	e3/e3	His
41	NC*	F	W	75	n.a.	n.a.	n.a.	n.a.	n.a.	n.a.	n.a.	n.a.	n.a.	n.a.	His
42	NC*	F	W	76	n.a.	n.a.	n.a.	n.a.	n.a.	n.a.	n.a.	n.a.	n.a.	n.a.	His
43	NC*	F	W	58	n.a.	n.a.	n.a.	n.a.	n.a.	n.a.	n.a.	n.a.	^30	n.a.	His
44	NC*	F	B	73	n.a.	n.a.	n.a.	n.a.	n.a.	n.a.	n.a.	n.a.	n.a.	n.a.	His
45	NC	F	H	85	2	1	2	0	−/+	I-II	1.5	0	30	e3/e3	His
46	NC	F	W	93	3	2	3	0	−/+	III-IV	3.5	1	n.a.	e2/e3	His
47	NC	F	W	95	1	0	0	I	−/−	I	1	0	30	n.a.	His
48	NC	M	W	88	2	1	2	0	−/n.a.	II	2	0	30	n.a.	His
49	NC*	M	W	77	n.a.	n.a.	n.a.	n.a.	n.a./+	n.a.	n.a.	n.a.	^30	n.a.	His
50	NC*	M	W	66	n.a.	n.a.	n.a.	n.a.	n.a.	n.a.	n.a.	n.a.	n.a.	n.a.	His
51	NC*	M	W	84	n.a.	n.a.	n.a.	n.a.	n.a.	n.a.	n.a.	n.a.	^30	n.a.	His
52	NC	M	W	70	n.a.	n.a.	n.a.	n.a.	n.a.	n.a.	n.a.	n.a.	30	n.a.	His
53	NC	M	W	69	0	0	1	0	−/+	I	1	1	28	n.a.	His
54	NC*	M	W	87	n.a.	n.a.	n.a.	n.a.	n.a.	n.a.	n.a.	n.a.	^30	n.a.	His
55	AD/AD	F	H	48	3	3	3	I-III	−/n.a.	V	5	n.a.	n.a.	n.a.	P/MS
56	AD	F	W	81	3	3	3	0	−/−	V	5	n.a.	12	n.a.	P/MS
57	AD	F	W	93	2	3	3	I	−/n.a.	V	5	3	n.a.	n.a.	P/MS
58	AD	M	W	88	2	3	2.	I	−/+	V-VI	5.5	1	18	n.a.	P/MS
59	AD	M	W	88	1	2	2	0	−/+	III	3	n.a.	n.a.	n.a.	P/MS
60	AD	F	W	94	3	3	3	0	−/+	V-VI	5.5	3	n.a.	n.a.	P/MS
61	AD	F	W	100	2	3	3	0	−/+	V-VI	5.5	2	16	n.a.	P/MS
62	NC*	F	W	75	n.a.	n.a.	n.a.	n.a.	n.a.	n.a.	n.a.	n.a.	n.a.	n.a.	P/MS
63	NC*	F	W	72	n.a.	n.a.	n.a.	n.a.	n.a.	n.a.	n.a.	n.a.	n.a.	n.a.	P/MS
64	NC	M	H	81	3	1	2	0	−/n.a.	I-II	1.5	0	23	n.a.	P/MS
65	NC*	M	W	69	n.a.	n.a.	n.a.	n.a.	n.a.	n.a.	n.a.	n.a.	n.a.	n.a.	P/MS
66	NC*	F	W	79	n.a.	n.a.	n.a.	n.a.	n.a.	n.a.	n.a.	n.a.	n.a.	n.a.	P/MS
67	NC*	F	W	76	n.a.	n.a.	n.a.	n.a.	n.a.	n.a.	n.a.	n.a.	n.a.	n.a.	P/MS
68	NC	M	W	85	0	1	3	I	−/+	I-II	1.5	0.5	n.a.	n.a.	P/MS

AD, Alzheimer's disease dementia; AD/AD, patient #20 with A260V mutation in presenilin-1 (PSEN1) and patient #55 with A431E mutation in PSEN1; MCI, mild cognitive impairment; NC, normal cognition (no dementia); F, female; M, male; A, Asian; B, Black; H, Hispanic; W, White; His, histology; P, proteins for ELISA; MS, mass spectrometry. ABC scores determined as: A, Aβ plaque score modified from Thal; B, NFT stage modified from Braak; C, Neuritic plaque score modified from CERAD; CAA, Cerebral amyloid angiopathy; LB, Lewy bodies; ASVD. Atherosclerosis; CDR, Clinical dementia rating; MMSE, Mini-Mental State Examination; ^Normal MMSE assigned per absence of dementia in NDRI cases in correlation analyses; n.a., not available; +: present; –: none; APOE, apolipoprotein alleles. Human donor tissues obtained from USC-ADRC, and *cases obtained from NDRI.

Supplementary Table 2. List of human donors whose postmortem brains were used for mass spectrometry.

Brains	Diagnosis	Sex	Race	Age at death	Plaque Stage	Braak Stage	Neuritic Plaque	Prion	Co-morbidity	MMSE score	APOE status	Study type
69	AD	M	W	86	C	6	4	M/M	none	19	3/4	MS
70	AD	F	W	89	C	6	4	n.a.	none	15	3/4	MS
71	AD	F	W	95	C	6	3	M/M	none	n.a.	2/3	MS
72	AD	F	W	93	C	6	4	M/M	none	13	3/3	MS
73	AD	F	W	88	C	5	4	V/V	HYCEPH	10	3/3	MS
74	AD	M	W	92	C	5	4	M/V	none	12	3/3	MS
75	AD	M	H	86	C	5	4	M/V	none	n.a.	3/3	MS
76	AD	F	W	98	C	6	4	M/V	none	14	3/3	MS
77	AD	F	W	91	C	6	4	M/V	none	17	3/3	MS
78	AD	F	W	82	C	6	4	n.a.	none	17	3/3	MS
79	NC	F	W	87	0	2	1	M/V	none	30	2/3	MS
80	NC	F	W	91	0	4	1	M/V	none	29	2/3	MS
81	NC	F	H/AI	95	0	2	1	V/V	none	n.a.	3/3	MS
82	NC	F	W	90	B	3	3	n.a.	LBD; DEP	18	3/3	MS
83	NC	M	W	96	0	2	1	M/V	none	29	2/3	MS
84	NC	M	W	94	0	1	1	n.a.	LBD; DEP	27	3/3	MS
85	NC	M	W	86	0	2	2	V/V	none	27	3/3	MS
86	NC	F	W	91	A	2	2	M/M	none	29	3/3	MS

AD, Alzheimer's disease; NC, normal cognition; F, female; M, male; AI, American Indian; H, Hispanic; W, White; MS, mass spectrometry; MMSE, Mini-Mental State Examination; n.a., not available. Plaque Stage: 0, none; A, phase I-II; B, phase III; C, phase IV-V; Neuritic Plaque (NP) scores: 4, frequent NP; 3, moderate NP; 2, sparce NP; 1, no NPs. Prion polymorphisms: methionine/methionine (M/M), methionine/valine (M/V), valine/valine (V/V). Co-morbidities: LBD, Lewy body disease; Dep, Depression; HYCEPH, Normal-pressure hydrocephalus. APOE, apolipoprotein alleles. All human donor tissues obtained from UCI-ADRC.

Supplementary Table 3. List of antibodies for immunohistochemistry.

Antibodies	**Source Species**	**Dilution**	**Application**	**Source**	**Catalog. #**
Primary antibody					
$A\beta_{42}$-12F4 mAb	Mouse	1:500	IF & Peroxidase	Biolegend	805502
GFAP mAb	Rat	1:500	IF	Invitrogen	13-0300
Iba-1 mAb	Rabbit	1:200	IF	Wako	019-19741
AβOi - scFvA13 mAb	Mouse*	1:450	IF	G. Meli Lab	scFvA13
β-III Tubulin pAb	Rabbit	1:500	IF	Abcam	ab18207
S100β mAb	Rabbit	1:150	IF	Abcam	ab52642
4G8 mAb	Mouse	1:250	IF	Biolegend	800702
$A\beta_{42}$ – JRF/cAβ 42/26	Mouse	1:5000	Peroxidase	Janssen	8151
JRF /Aβtot/17	Mouse	1:3500	Peroxidase	Janssen	Pur 117-120
CCasp3 pAb	Rabbit	1:250	IF	Cell Signaling	9661
Secondary antibody					
Cy2 (anti-rat, anti-rabbit)	Donkey	1:200	IF	Jackson ImmunoResearch Laboratories	711-225-152<br>712-225-150
Cy3 (anti-mouse, anti-rat, anti-rabbit)	Donkey	1:200	IF	Jackson ImmunoResearch Laboratories	715-165-150<br>712-165-153<br>711-165-152
Cy5 (anti-mouse, anti-rabbit)	Donkey	1:200	IF	Jackson ImmunoResearch Laboratories	715-175-150<br>711-175-152
Anti-V5 Tag mAb Intermediate	Mouse	1:200	IF	Invitrogen	R960-25
anti-mouse Ab - HRP	Goat	Drop/25ul	Peroxidase	Dako Envision	K4000/1
anti-mouse 10nm gold conjugate (for EM)	Goat	1:100	Immunogold labelling	Ted Pella, Inc.	15736

Aβ – amyloid β-protein; $A\beta_{42}$ – 42 amino acid-long Aβ alloform; AβOi – intracellular Aβ oligomers; EM – electron microscopy; GFAP – glial fibrillary acidic protein; Iba1 – ionized calcium binding adaptor molecule 1; CCasp3 – Cleaved Caspase-3; IF – immunofluorescence; mAb – monoclonal antibody; pAb – polyclonal antibody; Peroxidase – peroxidase-based immunostaining; S100β – S100 calcium-binding protein B; scFv – single chain Fv fragment; *mouse recombinant antibody fragment.

Supplementary Fig. 1. Brain NFT and NT severity scores assigned based on Gallyas silver stain and immunofluorescence of retinal Aβ_{42} and associated gliosis per subregion in human donors.

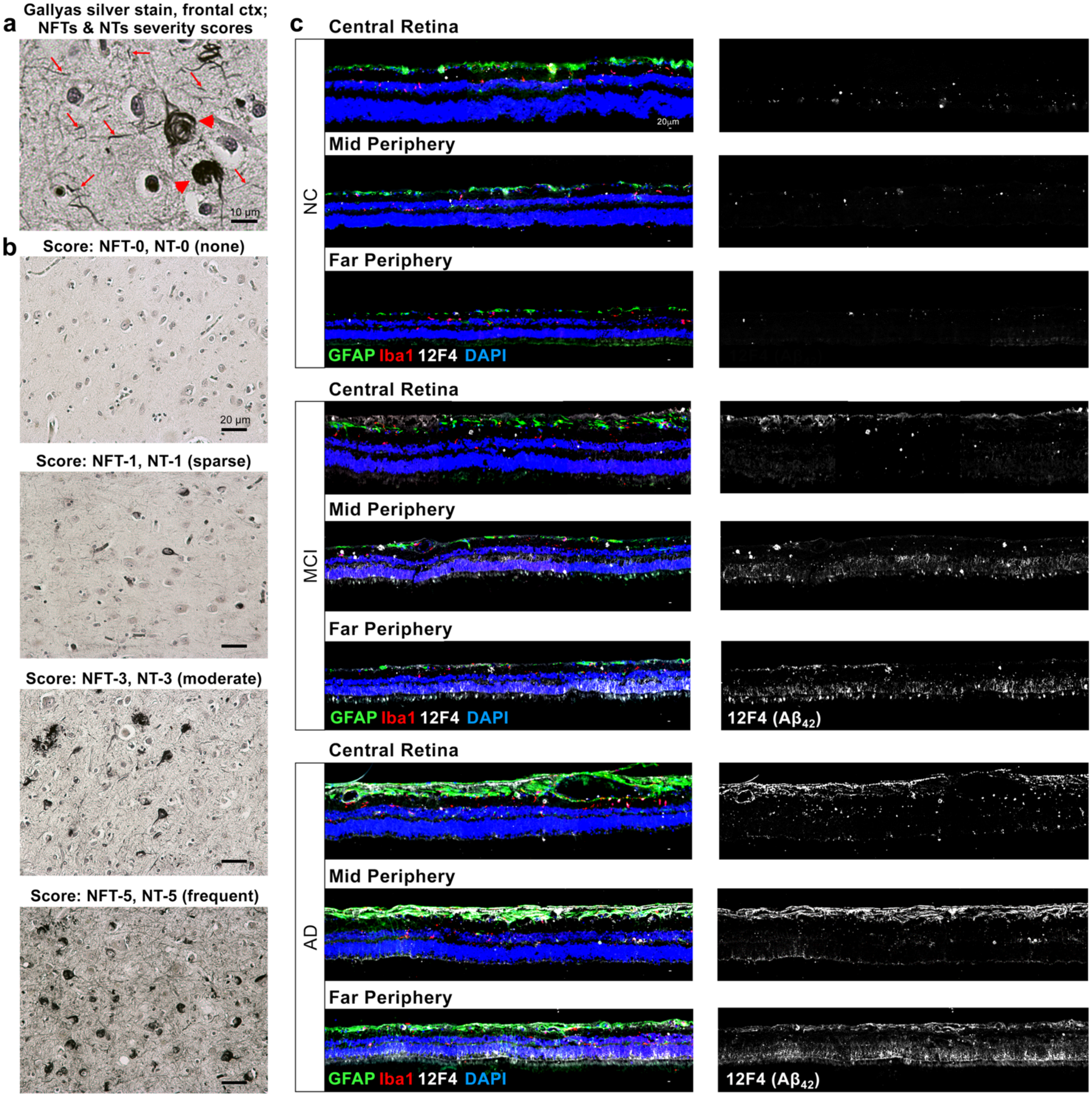

a. Gallyas silver stain detection of neurofibrillary tangles (NFTs, red arrowheads) and neuropil threads (NTs, red arrows) in a representative high-magnification microscopic image from the frontal cortex (ctx; superior frontal gyrus of the frontal lobe) of an AD patient. Scale bar: 10 µm. **b**. Brain NFT or NT severity scoring system was derived from observed burden of these AD neuropathologic changes detected here by Gallyas silver stain and measured per 1 mm^2 brain area. The assigned NFT or NT scores are as following: 0 = none; 1 = sparse (mild burden); 3 = moderate (intermediate burden); or 5 = frequent (severe burden). Representative microscopic images (20x; total magnification of 200x) of frontal cortices with assigned scores for each NFT or NT burdens, from NC subject (NFT-0, NT-0), MCI patient (NFT-1, NT-1), AD patient (NFT-3, NT-3), and another AD patient (NFT-5, NT-5). Scale bars: 20 µm. **c**. Representative tile scan fluorescence micrographs of retinal cross-sections from individuals with NC, MCI, or AD. Samples were immunolabeled for GFAP$^+$-macroglia (green), Iba1$^+$-microglia (red), 12F4$^+$-Aβ_{42} (white), and nuclei (DAPI, blue), and are shown separately in central, mid- and far peripheral retinal subregions of the retina. AD = Alzheimer's disease, MCI = mild cognitive impairment, NC = normal cognition. Merge channels (left column), 12F4$^+$-Aβ_{42} channel (white, right column). Scale bars = 20 µm.

Supplementary Fig. 2. Extended data on retinal $A\beta_{42}$ deposition in relation to sex, age, and PMI in MCI and AD patients compared to NC controls.

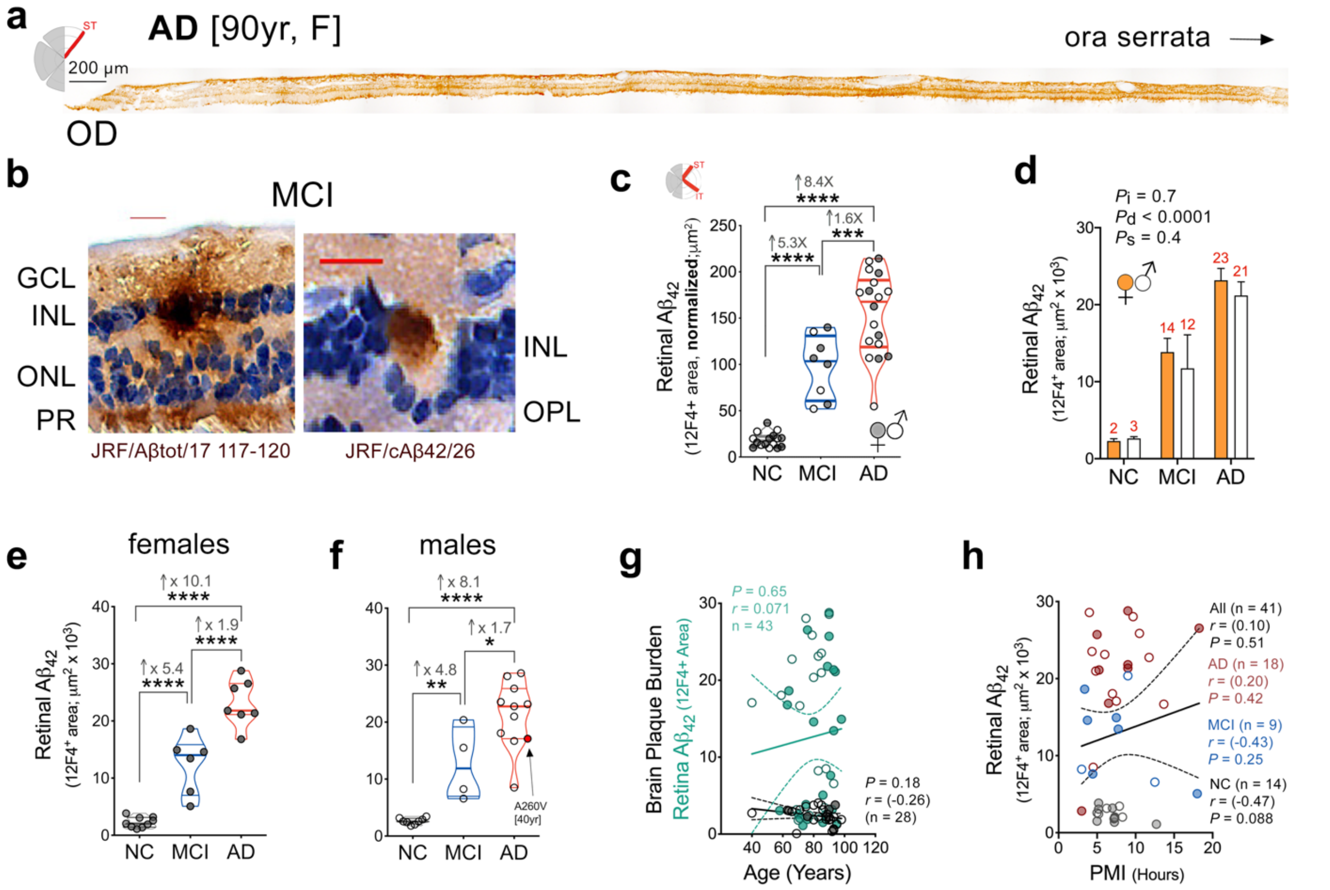

a. Representative tile image of the ST region extending from the optic disc (OD) to the ora serrata stained with 12F4 ($A\beta_{42}$) and peroxidase-based DAB. **b**. Micrographs of retinal cross-sections from an MCI patient that were stained with JRF/Aβtot/17 117-120 mAb (specific to the N′-terminus of Aβ; left image) and JRF/c$A\beta_{42/26}$ mAb (specific to the C′-terminus of $A\beta_{42}$; right image) with peroxidase-based DAB. Retinal Aβ plaques are seen in the INL. Scale bar: 20 µm. **c**. Quantitative IHC analysis of the retinal $A\beta_{42}$-immunoractive (IR) area normalized to retinal thickness in AD (n=18), MCI (n=8), or NC (n=16) patients. **d**. Bar graph displays retinal $A\beta_{42}$ burden separated by sex in the same cohort. Pd–diagnostic groups; Ps–females vs. males (sex groups); Pi–interactions. **e, f.** Quantitative retinal $A\beta_{42}$-IR areas in females with AD (n=7), MCI (n=6) or NC (n=9) and in males with AD (n=11), MCI (n=4), or NC (n=8). Red circle represents an ADAD patient with an A260V mutation in presenilin-1 (PSEN1). **g, h**. Pearson's correlation coefficient (*r*) analysis of (**g**) ages plotted against brain plaque burden or retina 12F4^{+}-$A\beta_{42}$ IR area and (**h**) the retinal 12F4^{+}-$A\beta_{42}$ IR area plotted against the postmortem interval (PMI). Violin plots show median and lower and upper quartiles, and individual data points are presented. $^{*}P < 0.05$, $^{**}P < 0.01$, $^{***}P < 0.001$, $^{****}P < 0.0001$, by one-or two-way ANOVA and Tukey's post-hoc multiple comparison test. Filled and empty circles represent women and men, respectively.

Supplementary Fig. 3. Extended TEM analysis of retinal $A\beta_{42}$ deposits in AD patients.

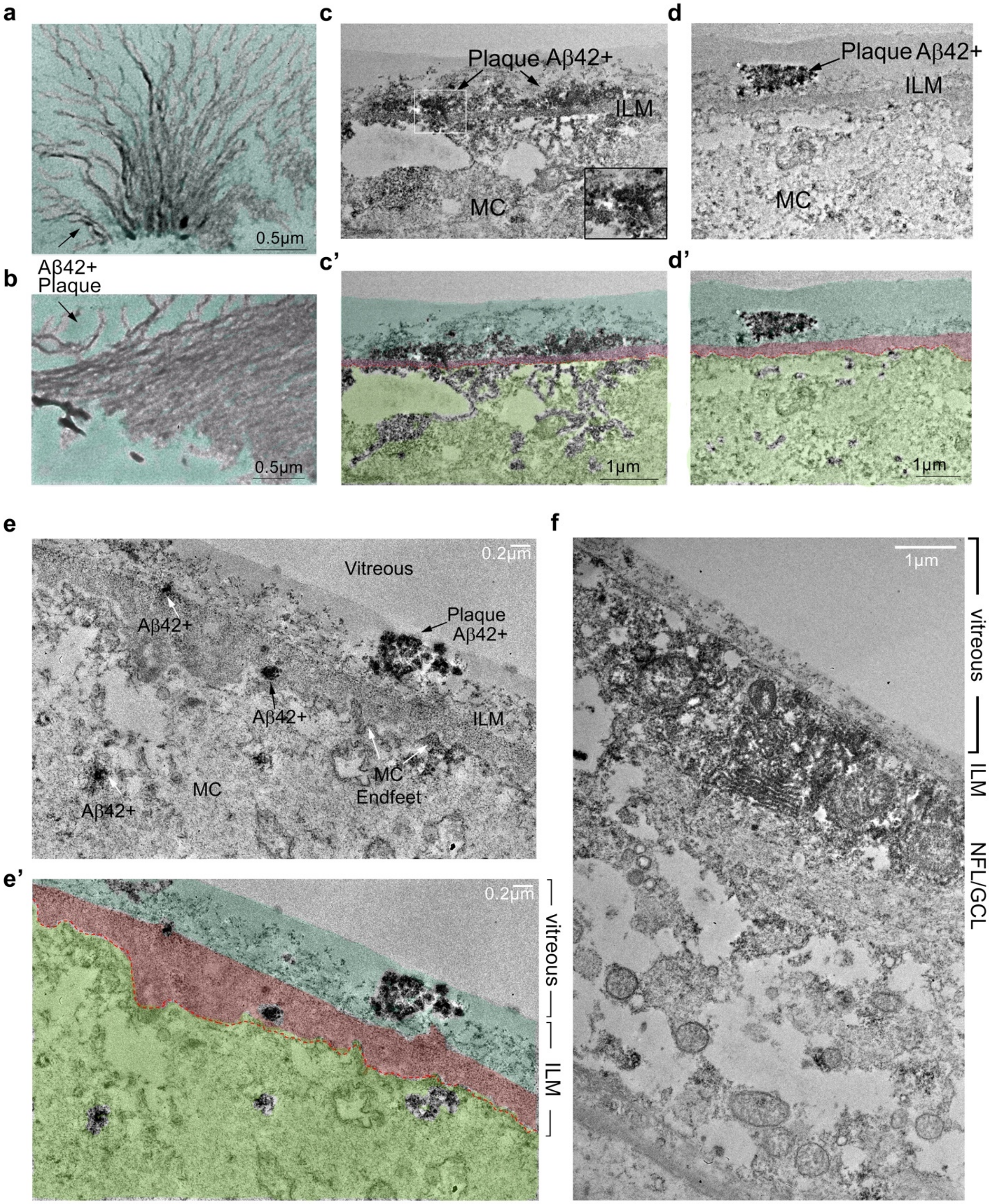

Human retinas from AD patients were pre-stained with anti-$A\beta_{42}$ mAb (12F4)-DAB. Transmission electron microscopy (TEM) analysis reveals the ultrastructure and location of retinal plaque $A\beta_{42}^{+}$ signals inside the inner limiting membrane (ILM) and Müller cell (MC) end feet (**a– e'**) as well as in the endoplasmic reticulum organelle in the GCL - ganglion cell layer (**f**). Pseudo colors were used to distinguish between the layers. Scale bars are indicated on each image. NFL = nerve fiber layer.

Supplementary Fig. 4. Spatial distribution of Aβ42 burden in retinal subregions and correlations with various brain regions and cognition.

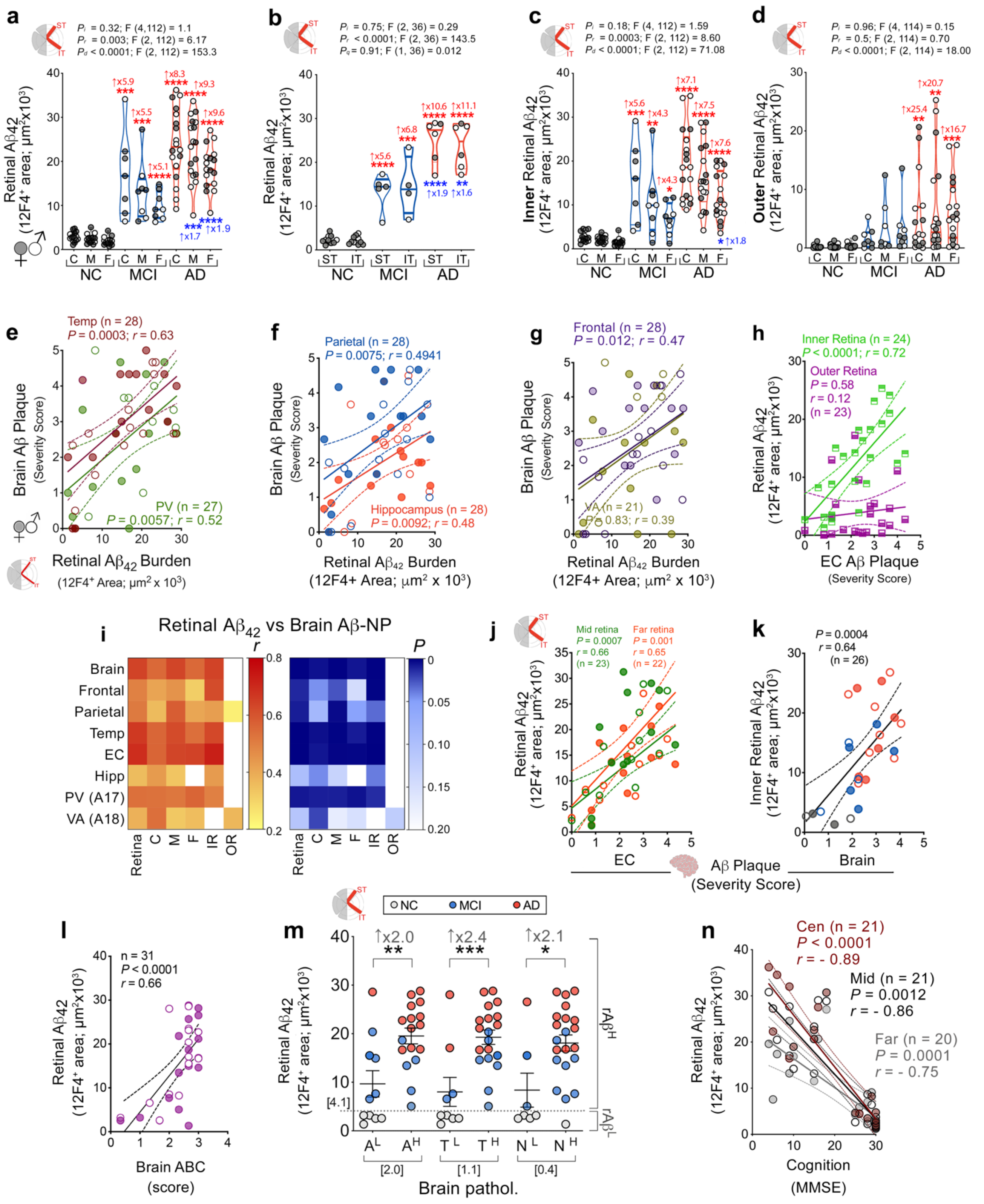

Stereological assessment of ST and IT retinal Aβ_{42} (12F4^{+}-IR area) in age- and sex-matched patients with NC (n=15), MCI (n = 7, 8), or AD (n = 17, 18) within predetermined geometric subregions: **a**. Quantitative 12F4^{+}-IR from central (C), mid- (M), and far peripheral (F) retinal subregions. **b**. Retinal 12F4^{+}-IR area in ST versus IT regions in a subset of donors with NC (n=11), MCI (n=4, 5) or AD (n=6). **c**. Quantitative inner retinal Aβ_{42}-IR area extending from the inner limiting membrane to the inner nuclear layer per C/M/F subregions. **d**. Quantitative outer retinal 12F4^{+}-IR area extending from the outer plexiform layer to the outer limiting membrane per C/M/F subregions in the same cohort. Pd–diagnostic groups; Pr–regions; Pi–interactions. **e-g**. Pearson's correlation coefficient (r) analysis of retinal 12F4^{+}-IR area plotted against brain Aβ plaque burden in (**e)** the superior temporal (Temp) and the primary visual (PV) cortices, (**f**) the superior parietal cortex and hippocampus, and (**g)** the superior frontal and visual association (VA) cortices. **h**. Correlation analyses of inner or outer retinal Aβ_{42} burden versus Aβ plaque in the entorhinal cortex (EC). **i**. Heatmaps summarizing Pearson's correlation coefficient (*r*) analyses between retinal Aβ_{42} and the cerebral Aβ neuritic plaque (NP) score in all analyzed regions of the brain and retina (n=25); pseudo-color grading illustrates the strength and significance of 'r' (red) and 'P' (blue) values, respectively. **j-l**. Pearson's correlation coefficient (*r*) analysis of (**j**) the retinal 12F4^{+}-IR area in the mid- and far peripheral subregions plotted against Aβ plaque burden in the EC, (**k**) the inner retinal 12F4^{+}-IR area plotted against Aβ plaque burden in the brain, and (**l**) the retinal 12F4^{+}-IR area plotted against the cerebral ABC score derived from a 4-point scale based on the following brain pathologies: amyloid plaque – A, NFT by Braak – B, and NP by CERAD – C. **m**. Retinal Aβ_{42} burden in patients stratified based on high (H) or low (L) brain ATN histopathology (severity scores) and color-labeled per diagnostic group (n=28); the extrapolated dotted-gray line marks the retinal Aβ_{42} level separating ATN^{H} from ATN^{L} individuals. Individual data points are presented with group means ± SEMs. **n**. Pearson's correlations between retinal Aβ_{42} burden per geometric subregion and MMSE cognitive scores. ST = superior-temporal, IT = inferior-temporal region of retina. Violin plots show median and lower and upper quartiles, and individual data points are presented. **P* < 0.05, ***P* < 0.01, ****P* < 0.001, *****P* < 0.0001, by one- or two-way ANOVA and Tukey's post-hoc multiple comparison test. Red and blue asterisks mark significance relative to NC and MCI, respectively. Filled and empty circles represent women and men, respectively.

Supplementary Fig. 5. Extended data on the intracellular scFvA13^{+}-Aβ oligomers (AβOi) distribution in the retinas of MCI and AD patients compared to retinas of NC controls and correlations with cerebral pathology and cognition.

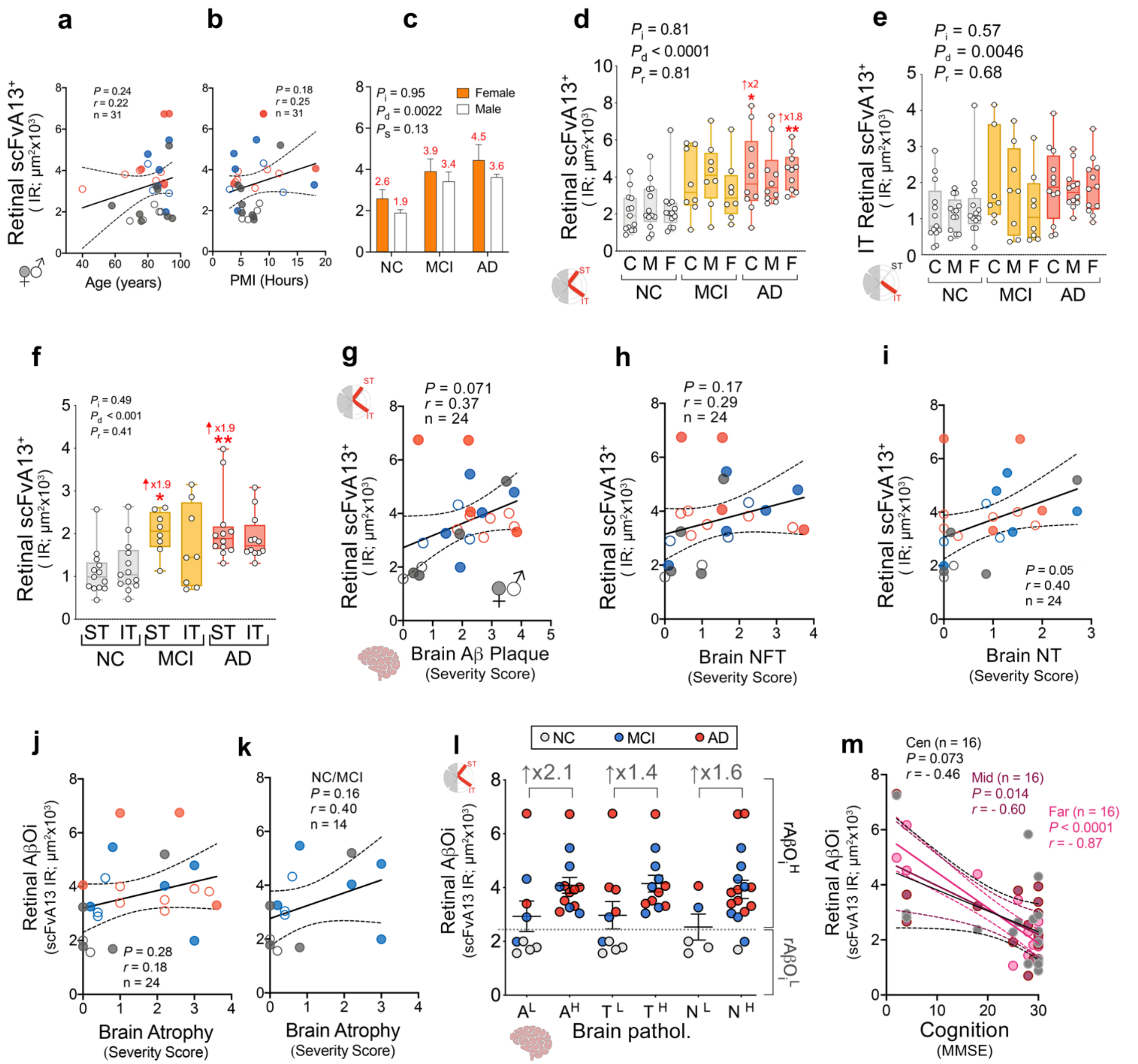

a-**b**. Pearson's correlation coefficient (r) analysis of retinal scFvA13^{+} (AβOi)-IR versus (**a**) age and (**b**) PMI. **c.** Bar graph displays the retinal scFvA13-burden separated by sex in AD (n=12) and MCI (n=8) patients vs. NC controls (n=13). Pd–diagnostic groups; Ps–females vs. males (sex groups); Pi–interactions. **d**-**f**. Mapping of retinal scFvA13^{+} in NC, MCI, and AD patient cohorts (n = 31–33) for (**d**) total ST/IT retina and (**e**) total IT, per C/ M/ F subregions, and (**f**) ST versus IT regions. Pd–diagnostic groups; Pr–regions; Pi–interactions. **g–k**. Pearson's correlation coefficient (*r*) analyses of retinal AβOi plotted against (**g**) cerebral Aβ plaque burden for all groups, (**h**) cerebral neurofibrillary tangle (NFT) pathology for all groups, (**i**) cerebral neuropil threads (NT) for all groups, (**j**) brain atrophy for all groups, and (**k**) NC and MCI groups. **l**. Analysis of the scFvA13^{+}-AβOi area in human retinas stratified based on high (H) or low (L) brain ATN histopathology and color-labeled per diagnostic group (n=22); extrapolated dotted-gray line marks the retinal AβOi level separating ATN^{H} from ATN^{L} individuals. **m**. Pearson's correlation between retinal AβOi per C/M/F subregions and MMSE cognitive scores. Median and lower and upper quartiles are indicated on each box plot, and individual data points are presented. $*P < 0.05$, $**P < 0.01$, by one- or two- way ANOVA and Tukey's post-hoc multiple comparison test. Red asterisks mark significance relative to NC. Filled and empty circles represent women and men, respectively. AβOi: intraneuronal Aβ oligomers.

Supplementary Fig. 6. Extended data on retinal S100β^{+} and GFAP^{+} macrogliosis distribution in MCI and AD patients and correlations to cerebral pathology and cognition.

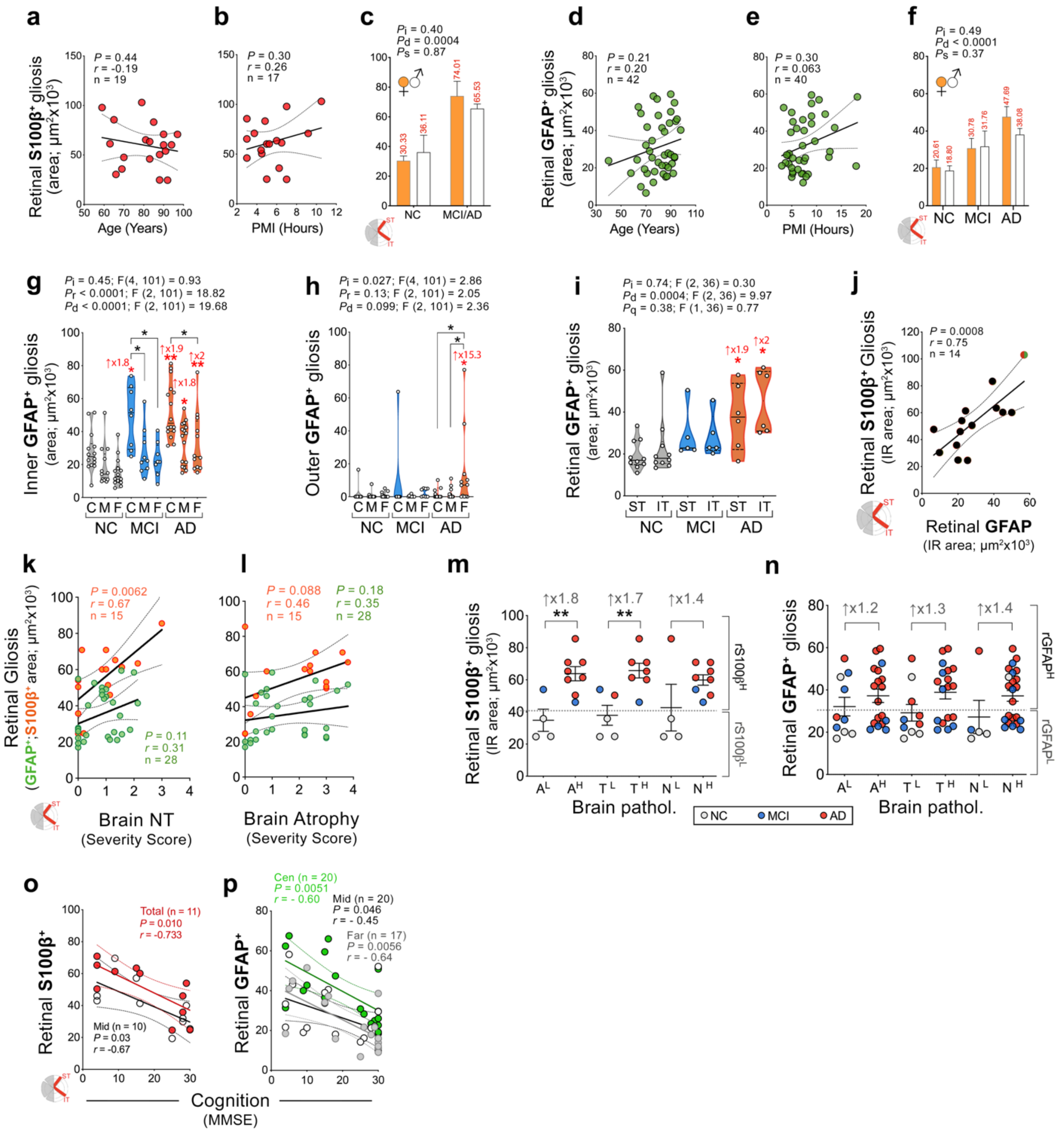

a, b. Pearson's correlation coefficient (*r*) analyses of the retinal gliosis S100β^{+}-IR area vs. (**a**) age and (**b**) PMI. **c**. Quantitative retinal S100β^{+}-IR area in males compared to females in NC (n = 5) vs. MCI/AD groups (n = 10-12). **d, e** Pearson's correlation coefficient (*r*) analyses of the retinal gliosis GFAP^{+}-IR area vs. (**d**) age and (**e**) PMI**. f**. Quantitative retinal GFAP^{+}-IR area in males compared to females in NC (n = 16), MCI (n = 9) and AD (n = 17) groups. **g–i**. Mapping of the retinal GFAP^{+} gliosis-IR area in (**g**) the inner retinal layer from C/M/F subregions, (**h**) the outer retinal layer from C/M/F subregions, and (**i**) the ST vs. IT retina in the same cohort. Pd–diagnostic groups; Pr–regions; Pi–interactions. **j-l**. Pearson's correlation coefficient (r) analyses in a subset of NC, MCI, and AD patients between (**j**) reactive gliosis S100β^{+} against GFAP^{+}, reactive gliosis (S100β^{+}; orange or GFAP^{+}; green) area against (**k**) brain NT, and (**l**) brain atrophy score. **m, n**. Retinal (**m**) S100β^{+}-IR area and (**n**) GFAP^{+}-IR area data in human donors stratified according to high (H) and low (L) brain ATN histopathology (severity scores) and color-labeled per diagnostic group (S100β^{+}, n=12; GFAP^{+}, n=28); extrapolated dotted-gray line signifies the level of retinal gliosis separating ATN^{H} and ATN^{L} individuals. **o, p**. Pearson's correlation coefficient (r) analyses between the retinal (**o**) S100β^{+} area or (**p**) GFAP^{+} area per C/M/F regions vs. MMSE score. Violin plots show median and lower and upper quartiles, and individual data points are presented. *$P < 0.05$, **$P < 0.01$ by one- or two- way ANOVA and Tukey's post-hoc multiple comparison test. Red asterisks mark significance relative to NC.

Supplementary Fig. 7. Extended data on spatial distribution of retinal IBA1$^+$ microgliosis and correlations with retina and brain pathology and cognition.

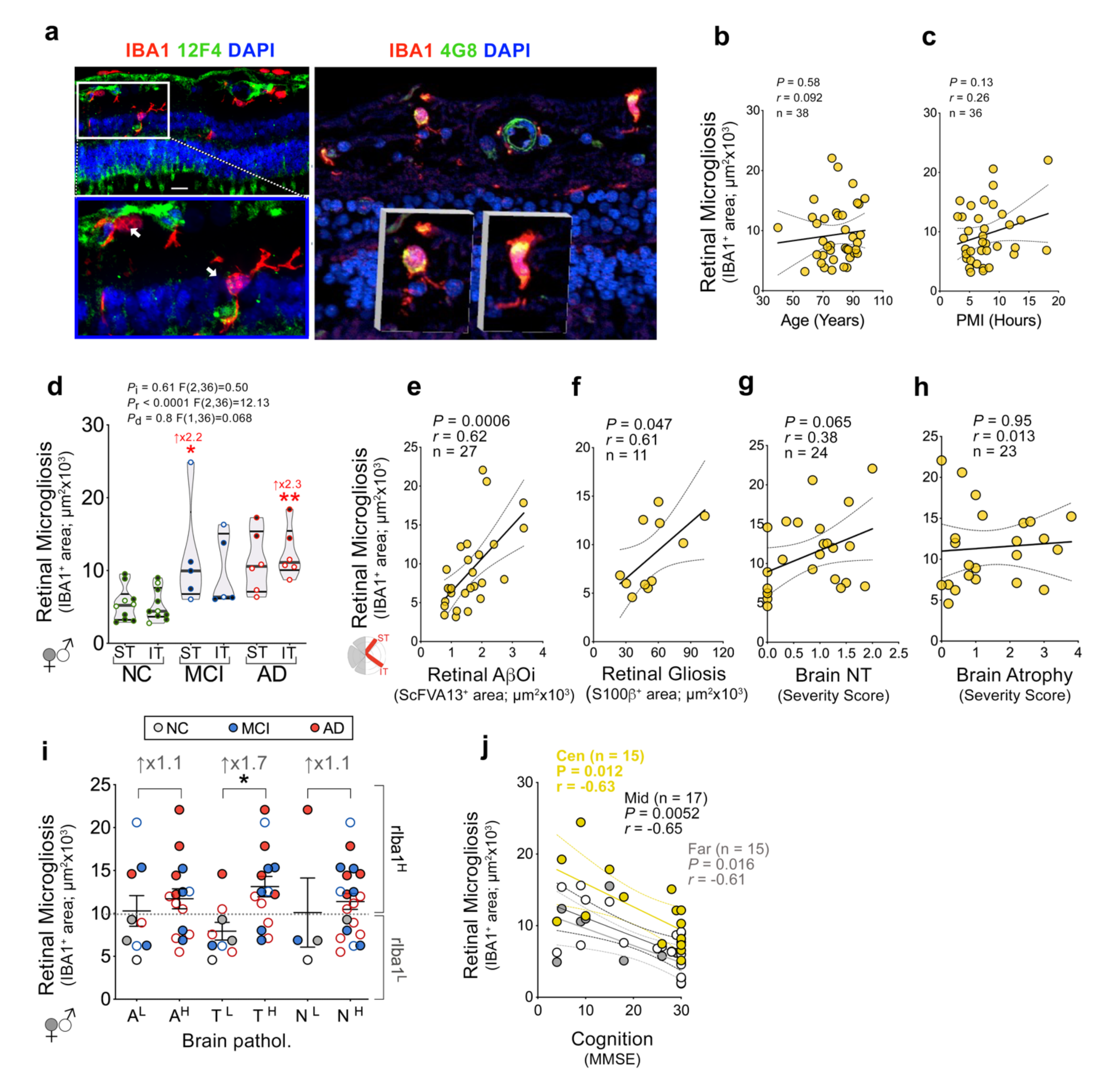

a. Representative immunofluorescence images of retinal cross-sections from AD patients showing IBA1$^+$-microgliosis (red), 12F4$^+$-Aβ_{42} or 4G8$^+$-Aβ_{42} deposits (green), and nuclei (DAPI, blue). Enlarged image depicts retinal microglia engulfing Aβ_{42} deposits (white arrows). **b, c**. Pearson's correlation coefficient (r) analyses between Iba1$^+$ -microgliosis plotted against (**b)** age and (**c**) PMI in cohorts of donors with AD, MCI, or NC (n=36–38). **d**. Mapping of the retinal IBA1$^+$-IR area in the ST compared to the IT in NC controls (n=10) and MCI (n=5) and AD (n=6) patients. Pd–diagnostic groups; Pr–regions; Pi–interactions. **e-h.** Pearson's correlation coefficient (r) analyses of retinal IBA1$^+$-microgliosis in ST/IT regions plotted against (**e**) retinal AβOi, (**f**) retinal S100β$^+$ gliosis, (**g**) retinal brain neuropil threads (NT), and (**h)** brain atrophy scores**. i**. Analysis of the retinal IBA1$^+$-microgliosis area in individuals stratified according to high (H) or low (L) brain ATN histopathology and color-labeled per diagnostic group (n = 24); extrapolated dotted-gray line marks the level of retinal Iba1$^+$-microgliosis separating ATN^H and ATN^L individuals. **j**. Pearson's correlation between retinal IBA1 microgliosis per C/M/F subregions and MMSE cognitive score. Median and lower and upper quartiles are indicated on each violin plot, and individual data points are presented. *$P < 0.05$, **$P < 0.01$ by one- or two-way ANOVA and Tukey's post-hoc multiple comparison test. Red asterisks mark significance relative to NC group. Filled and empty circles represent women and men, respectively.

Supplementary Figure 8. Extended data on retinal atrophy and correlations with other cerebral biomarkers.

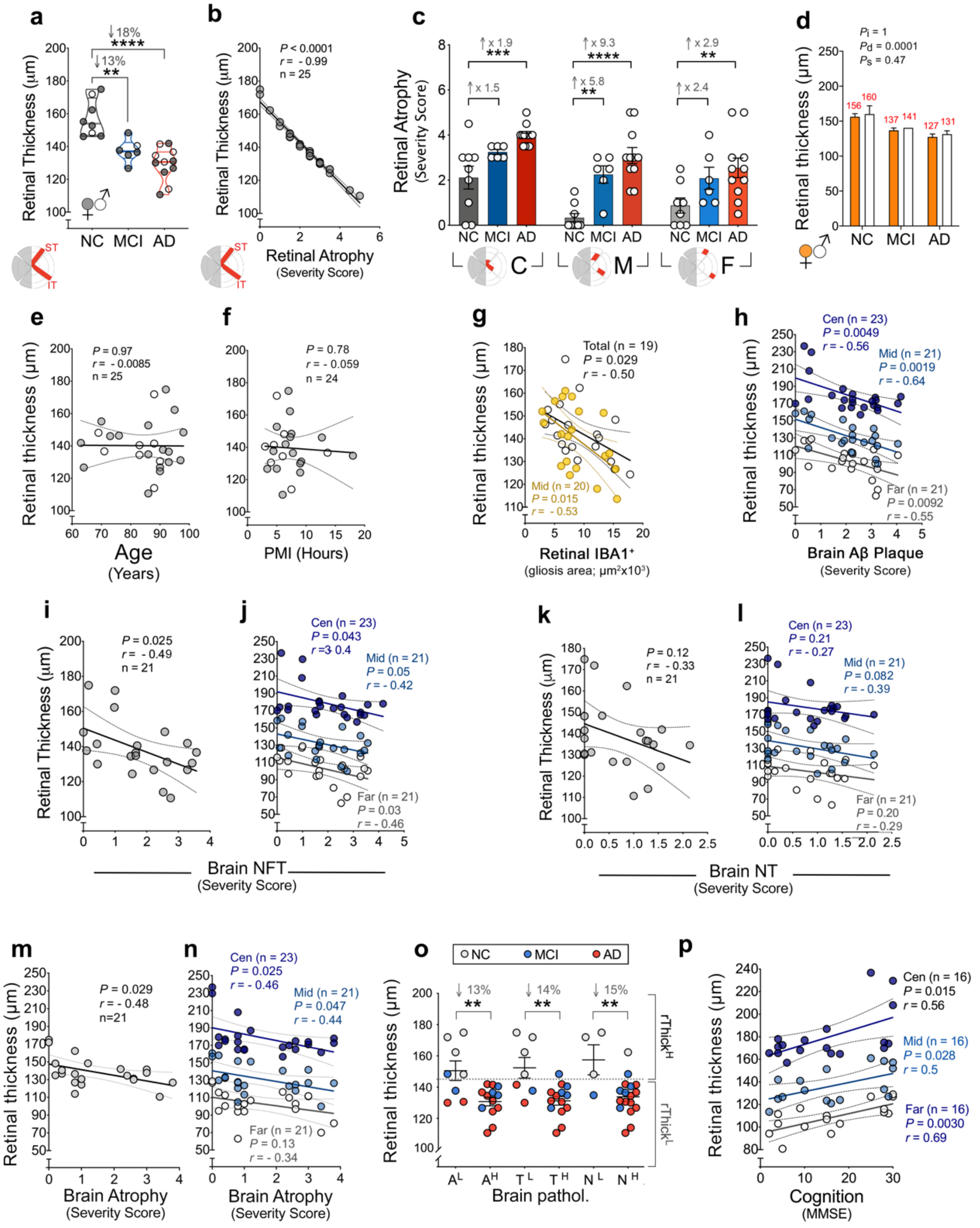

a. Quantification of retinal thickness (µm) in MCI and AD (n=17) patients and age- and sex-matched NC controls (n=8). **b**. Pearson's correlation coefficient (*r*) analysis of retinal thickness versus retinal atrophy severity score. **c**. Mapping of ST/IT retinal atrophy in this human cohort (n=26) in pre-defined central (C), mid- (M), and far (F) peripheral subregions. **d**. Quantitative retinal thickness per sex. **e-n**. Pearson's correlation coefficient (*r*) analysis of retinal thickness (µm) vs. (**e**) age, (**f**) PMI, (**g**) retinal IBA1^{+}-IR, (**h**) cerebral Aβ plaque burden per C/M/F subregions, (**i**) brain neurofibrillary tangles (NFTs)**,** (**j**) brain NFTs per C/M/F subregions, (**k**) brain neuropil threads, (**l**) brain neuropil threads per C/M/F subregions, (**m**) brain atrophy and **(n)** brain atrophy per C/M/F subregions. **o**. Analysis of retinal thickness stratified by high (H) or low (L) brain ATN histopathology and color-labeled per diagnostic group (n = 21); extrapolated dotted-gray line marks the level of retinal thickness (µm) separating ATN^{H} and ATN^{L} individuals. **p**. Correlations of retinal thickness in the 3 subregions vs. MMSE cognitive scores. Violin plots show median, lower and upper quartiles, and individual data points are presented. $^{**}P < 0.01$, $^{***}P < 0.001$, $^{****}P < 0.0001$ by one-or two-way ANOVA and Tukey's post-hoc multiple comparison test. Individual data points are presented with group means ± SEMs. Filled and empty circles represent women and men, respectively.

Supplementary Fig. 9. Extended global proteomics data in retinas and brains from AD patients and NC controls.

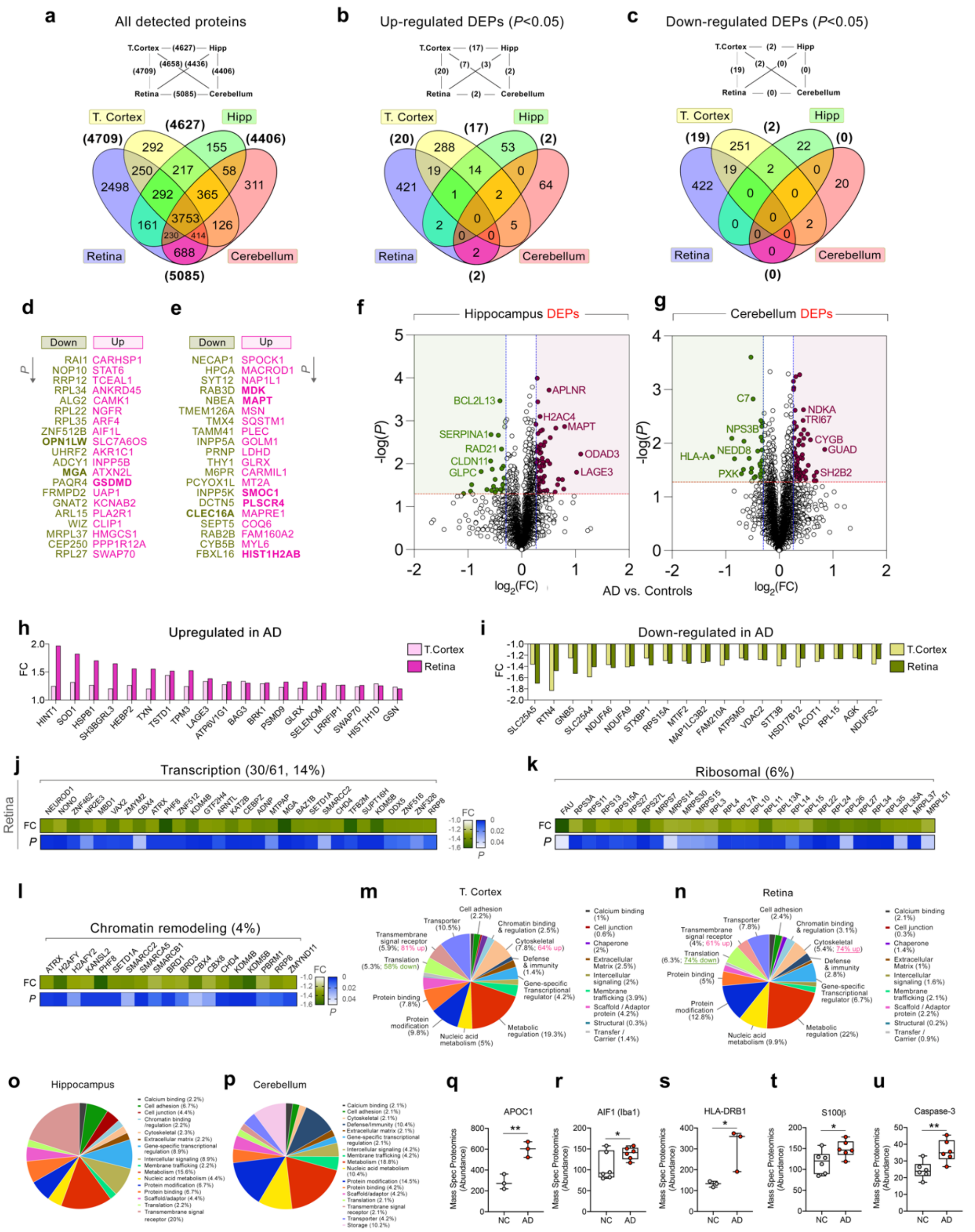

a–**c**. Venn diagrams showing (**a**) the total number of proteins detected by mass spectrometry and overlap between 3 CNS tissues as well as significantly (**b**) upregulated and (**c**) downregulated differentially expressed proteins (DEPs), based on statistical significance of $P<0.05$ and 1.2-fold change (FC) threshold criteria, with overlap between the 4 analyzed CNS tissues. The number of overlapping DEPs between each pair of CNS tissues is shown bolded in parentheses. **d**, **e**. Top 20 most significant (by p-value) upregulated (pink) and downregulated (green) proteins in the (**d**) temporal-hemiretina retina and (**e**) temporal cortex (T.Cortex) of AD patients compared to those of NC controls. DEPs are listed by ascending *P* values, with the upper DEPs having the lowest *P* values (most significant). Among these, the proteins most changed by FC are highlighted by bolded text. **f, g**. Volcano plots of all detected proteins in (**f**) the hippocampus and (**g**) cerebellum of AD patients compared to those in NC controls, with DEPs shown in red. **h, i.** Common (**h**) up- or (**i**) downregulated proteins between the retina and temporal cortex in AD patients versus controls. **j-l.** DAVID pathway analysis of AD retinas of top downregulated DEPs by FC related to the following functions: (**j**) transcription, (**k**) ribosome, and (**l**) chromatin remodeling, % proteins in a certain function of total downregulated DEPs). **m–p**. Pie charts from PANTHER functional classification analysis showing percentage of significant DEPs in (**m**) the temporal cortex, (**n**) retina, (**o**) hippocampus, and (**p**) cerebellum of AD patients compared to those in NC controls, grouped by protein class category. **q**–**u**. Box plot analysis of individual AD-associated DEPs—(**q**) APOC1, (**r**) AIF1/Iba1, (**s**) HLA-DRB1, (**t**) S100β, and (**u**) Caspase-3—related to neuroinflammation and atrophy in the retinas of AD patients vs. retinas of NC controls. Box and whiskers plots show median, lower and upper quartiles, and individual data points are presented. $^{*}P < 0.05$, $^{**}P < 0.01$ by unpaired two-tailed Student's test.

Supplementary Table 4. Extended mass spectrometry data on significantly up-regulated proteins in the retina of Alzheimer's patients compared to cognitively normal controls.

Accession	Description	AD (Means)	NC (Means)	FC	*P*
Q9Y2V2	Calcium-regulated heat-stable protein 1 OS=Homo sapiens OX=9606 GN=CARHSP1 PE=1 SV=2	262.44	153.78	1.71	8.643E-05
P42226	Signal transducer and activator of transcription 6 OS=Homo sapiens OX=9606 GN=STAT6 PE=1 SV=1	126.47	99.89	1.27	9.612E-05
Q15170	Isoform 2 of Transcription elongation factor A protein-like 1 OS=Homo sapiens OX=9606 GN=TCEAL1	121.35	76.26	1.59	0.00030
Q5TZF3	Isoform 2 of Ankyrin repeat domain-containing protein 45 OS=Homo sapiens OX=9606 GN=ANKRD45	22.38	14.49	1.54	0.00032
Q14012	Calcium/calmodulin-dependent protein kinase type 1 OS=Homo sapiens OX=9606 GN=CAMK1 PE=1 SV=1	143.38	108.58	1.32	0.00032
P08138	Isoform 2 of Tumor necrosis factor receptor superfamily member 16 OS=Homo sapiens OX=9606 GN=NGFR	40.44	24.72	1.64	0.00065
P18085	ADP-ribosylation factor 4 OS=Homo sapiens OX=9606 GN=ARF4 PE=1 SV=3	883.54	645.81	1.37	0.00065
Q9BQI0	Allograft inflammatory factor 1-like OS=Homo sapiens OX=9606 GN=AIF1L PE=1 SV=1	231.24	162.04	1.43	0.00066
Q96CW6	Probable RNA polymerase II nuclear localization protein SLC7A6OS OS=Homo sapiens OX=9606 GN=SLC7A6OS PE=1 SV=2	55.22	35.09	1.57	0.00070
Q04828	Aldo-keto reductase family 1 member C1 OS=Homo sapiens OX=9606 GN=AKR1C1 PE=1 SV=1	400.78	269.55	1.49	0.00074
P32019	Type II inositol 1,4,5-trisphosphate 5-phosphatase OS=Homo sapiens OX=9606 GN=INPP5B PE=1 SV=4	15.26	9.54	1.60	0.00080
H3BUF6	Ataxin-2-like protein OS=Homo sapiens OX=9606 GN=ATXN2L PE=1 SV=1	30.04	21.65	1.39	0.00080
G3V1A6	Gasdermin domain containing 1, isoform CRA_d OS=Homo sapiens OX=9606 GN=GSDMD PE=1 SV=1	31.58	15.62	2.02	0.00090
Q16222	UDP-N-acetylhexosamine pyrophosphorylase OS=Homo sapiens OX=9606 GN=UAP1 PE=1 SV=3	14.51	9.33	1.55	0.00099
Q13303	Isoform 3 of Voltage-gated potassium channel subunit beta-2 OS=Homo sapiens OX=9606 GN=KCNAB2	122.11	83.92	1.46	0.00100
Q13018	Secretory phospholipase A2 receptor OS=Homo sapiens OX=9606 GN=PLA2R1 PE=1 SV=2	154.38	127.41	1.21	0.00112
P30622	CAP-Gly domain-containing linker protein 1 OS=Homo sapiens OX=9606 GN=CLIP1 PE=1 SV=2	1073.32	883.48	1.21	0.00124
Q01581	Hydroxymethylglutaryl-CoA synthase, cytoplasmic OS=Homo sapiens OX=9606 GN=HMGCS1 PE=1 SV=2	123.62	90.34	1.37	0.00126
O14974	Protein phosphatase 1 regulatory subunit 12A OS=Homo sapiens OX=9606 GN=PPP1R12A PE=1 SV=1	1259.97	1048.38	1.20	0.00128
Q9UH65	Switch-associated protein 70 OS=Homo sapiens OX=9606 GN=SWAP70 PE=1 SV=1	440.63	348.41	1.26	0.00147

E9PSH4	Repressor of RNA polymerase III transcription MAF1 OS=Homo sapiens OX=9606 GN=MAF1 PE=1 SV=1	57.64	36.07	1.60	0.00156
O15514	DNA-directed RNA polymerase II subunit RPB4 OS=Homo sapiens OX=9606 GN=POLR2D PE=1 SV=1	48.86	37.89	1.29	0.00158
Q9H1P3	Oxysterol-binding protein-related protein 2 OS=Homo sapiens OX=9606 GN=OSBPL2 PE=1 SV=1	475.60	393.03	1.21	0.00161
Q9NRV9	Heme-binding protein 1 OS=Homo sapiens OX=9606 GN=HEBP1 PE=1 SV=1	930.77	667.70	1.39	0.00171
Q12841	Follistatin-related protein 1 OS=Homo sapiens OX=9606 GN=FSTL1 PE=1 SV=1	114.74	90.64	1.27	0.00171
Q9P287	BRCA2 and CDKN1A-interacting protein OS=Homo sapiens OX=9606 GN=BCCIP PE=1 SV=1	260.96	149.99	1.74	0.00179
A0A087WY62	Neuroblastoma breakpoint family member 26 OS=Homo sapiens OX=9606 GN=NBPF26 PE=4 SV=3	99.18	59.84	1.66	0.00190
Q14657	EKC/KEOPS complex subunit LAGE3 OS=Homo sapiens OX=9606 GN=LAGE3 PE=1 SV=2	32.15	23.23	1.38	0.00202
Q92995	Ubiquitin carboxyl-terminal hydrolase 13 OS=Homo sapiens OX=9606 GN=USP13 PE=1 SV=2	14.83	10.03	1.48	0.00213
A0A087X091	WD repeat-containing protein 13 (Fragment) OS=Homo sapiens OX=9606 GN=WDR13 PE=1 SV=1	78.88	63.41	1.24	0.00234
O15355	Protein phosphatase 1G OS=Homo sapiens OX=9606 GN=PPM1G PE=1 SV=1	747.14	588.94	1.27	0.00251
Q9UKW4	Isoform 4 of Guanine nucleotide exchange factor VAV3 OS=Homo sapiens OX=9606 GN=VAV3	164.63	137.13	1.20	0.00272
Q14376	UDP-glucose 4-epimerase OS=Homo sapiens OX=9606 GN=GALE PE=1 SV=2	439.78	293.33	1.50	0.00279
Q9H6X2	Anthrax toxin receptor 1 OS=Homo sapiens OX=9606 GN=ANTXR1 PE=1 SV=2	52.50	40.64	1.29	0.00298
Q9UK22	F-box only protein 2 OS=Homo sapiens OX=9606 GN=FBXO2 PE=1 SV=2	982.04	778.07	1.26	0.00300
P62330	ADP-ribosylation factor 6 OS=Homo sapiens OX=9606 GN=ARF6 PE=1 SV=2	431.76	298.76	1.45	0.00312
P00915	Carbonic anhydrase 1 OS=Homo sapiens OX=9606 GN=CA1 PE=1 SV=2	2538.21	1397.54	1.82	0.00313
Q9BW83	Intraflagellar transport protein 27 homolog OS=Homo sapiens OX=9606 GN=IFT27 PE=1 SV=1	556.96	395.36	1.41	0.00316
P02489	Alpha-crystallin A chain OS=Homo sapiens OX=9606 GN=CRYAA PE=1 SV=2	13374.84	8743.41	1.53	0.00324
P02042	Hemoglobin subunit delta OS=Homo sapiens OX=9606 GN=HBD PE=1 SV=2	3439.58	1703.27	2.02	0.00331
P05413	Fatty acid-binding protein, heart OS=Homo sapiens OX=9606 GN=FABP3 PE=1 SV=4	918.93	601.21	1.53	0.00340
O15405	TOX high mobility group box family member 3 OS=Homo sapiens OX=9606 GN=TOX3 PE=1 SV=2	77.89	61.67	1.26	0.00354
O15212	Prefoldin subunit 6 OS=Homo sapiens OX=9606 GN=PFDN6 PE=1 SV=1	653.96	376.98	1.73	0.00357
Q9Y5K3	Choline-phosphate cytidylyltransferase B OS=Homo sapiens OX=9606 GN=PCYT1B PE=1 SV=1	166.76	121.81	1.37	0.00359
P09497	Isoform Non-brain of Clathrin light chain B OS=Homo sapiens OX=9606 GN=CLTB	462.23	249.08	1.86	0.00362

O00233	26S proteasome non-ATPase regulatory subunit 9 OS=Homo sapiens OX=9606 GN=PSMD9 PE=1 SV=3	565.79	427.45	1.32	0.00370
P09936	Ubiquitin carboxyl-terminal hydrolase isozyme L1 OS=Homo sapiens OX=9606 GN=UCHL1 PE=1 SV=2	5218.63	3210.77	1.63	0.00379
P68871	Hemoglobin subunit beta OS=Homo sapiens OX=9606 GN=HBB PE=1 SV=2	13260.96	6808.23	1.95	0.00384
P30085	UMP-CMP kinase OS=Homo sapiens OX=9606 GN=CMPK1 PE=1 SV=3	1046.10	626.46	1.67	0.00386
P36021	Monocarboxylate transporter 8 OS=Homo sapiens OX=9606 GN=SLC16A2 PE=1 SV=2	25.83	20.70	1.25	0.00409
Q14574	Isoform 3B of Desmocollin-3 OS=Homo sapiens OX=9606 GN=DSC3	67.06	41.13	1.63	0.00419
A6ZKI3	Retrotransposon Gag-like protein 8C OS=Homo sapiens OX=9606 GN=RTL8C PE=1 SV=1	340.77	232.88	1.46	0.00422
Q99584	Protein S100-A13 OS=Homo sapiens OX=9606 GN=S100A13 PE=1 SV=1	1730.80	1073.04	1.61	0.00426
O15217	Glutathione S-transferase A4 OS=Homo sapiens OX=9606 GN=GSTA4 PE=1 SV=1	135.42	94.80	1.43	0.00435
Q7Z449	Cytochrome P450 2U1 OS=Homo sapiens OX=9606 GN=CYP2U1 PE=1 SV=1	133.60	108.12	1.24	0.00441
A0A0U1RRM6	Protein enabled homolog OS=Homo sapiens OX=9606 GN=ENAH PE=1 SV=1	310.29	235.12	1.32	0.00443
P31749	RAC-alpha serine/threonine-protein kinase OS=Homo sapiens OX=9606 GN=AKT1 PE=1 SV=2	506.93	420.35	1.21	0.00448
Q96QA5	Gasdermin-A OS=Homo sapiens OX=9606 GN=GSDMA PE=1 SV=4	125.32	65.57	1.91	0.00451
Q9NX08	COMM domain-containing protein 8 OS=Homo sapiens OX=9606 GN=COMMD8 PE=1 SV=1	45.31	33.39	1.36	0.00454
A0A2R8Y7C0	Hemoglobin subunit alpha (Fragment) OS=Homo sapiens OX=9606 GN=HBA2 PE=1 SV=1	5429.62	2843.25	1.91	0.00456
A0A087X0M8	Neural cell adhesion molecule L1-like protein OS=Homo sapiens OX=9606 GN=CHL1 PE=1 SV=1	255.41	200.14	1.28	0.00459
Q15370	Elongin-B OS=Homo sapiens OX=9606 GN=ELOB PE=1 SV=1	1449.87	1066.65	1.36	0.00464
P19971	Isoform 2 of Thymidine phosphorylase OS=Homo sapiens OX=9606 GN=TYMP	141.34	92.93	1.52	0.00524
Q9BV86	N-terminal Xaa-Pro-Lys N-methyltransferase 1 OS=Homo sapiens OX=9606 GN=NTMT1 PE=1 SV=3	154.88	102.59	1.51	0.00525
P04792	Heat shock protein beta-1 OS=Homo sapiens OX=9606 GN=HSPB1 PE=1 SV=2	3569.10	2093.53	1.70	0.00539
O00499	Isoform IIB of Myc box-dependent-interacting protein 1 OS=Homo sapiens OX=9606 GN=BIN1	79.67	61.50	1.30	0.00562
P52566	Rho GDP-dissociation inhibitor 2 OS=Homo sapiens OX=9606 GN=ARHGDIB PE=1 SV=3	393.32	281.16	1.40	0.00565
O75368	SH3 domain-binding glutamic acid-rich-like protein OS=Homo sapiens OX=9606 GN=SH3BGRL PE=1 SV=1	1705.57	856.93	1.99	0.00568
Q9H299	SH3 domain-binding glutamic acid-rich-like protein 3 OS=Homo sapiens OX=9606 GN=SH3BGRL3 PE=1 SV=1	818.47	495.54	1.65	0.00577

Q8IYD1	Eukaryotic peptide chain release factor GTP-binding subunit ERF3B OS=Homo sapiens OX=9606 GN=GSPT2 PE=1 SV=2	112.56	90.67	1.24	0.00584
O00273	DNA fragmentation factor subunit alpha OS=Homo sapiens OX=9606 GN=DFFA PE=1 SV=1	501.47	380.95	1.32	0.00602
H7C394	Calcium/calmodulin-dependent protein kinase type II subunit beta (Fragment) OS=Homo sapiens OX=9606 GN=CAMK2B PE=1 SV=1	54.35	41.07	1.32	0.00602
P67936	Tropomyosin alpha-4 chain OS=Homo sapiens OX=9606 GN=TPM4 PE=1 SV=3	2478.28	1531.27	1.62	0.00605
Q9UJ68	Mitochondrial peptide methionine sulfoxide reductase OS=Homo sapiens OX=9606 GN=MSRA PE=1 SV=1	77.57	62.20	1.25	0.00606
P69905	Hemoglobin subunit alpha OS=Homo sapiens OX=9606 GN=HBA1 PE=1 SV=2	463.25	198.84	2.33	0.00613
H7BZ14	Peptidyl-prolyl cis-trans isomerase (Fragment) OS=Homo sapiens OX=9606 GN=PPIL3 PE=1 SV=1	345.89	237.99	1.45	0.00616
J3QRF4	RNA binding protein fox-1 homolog OS=Homo sapiens OX=9606 GN=RBFOX3 PE=1 SV=1	33.06	26.92	1.23	0.00623
Q9NZ08	Isoform 2 of Endoplasmic reticulum aminopeptidase 1 OS=Homo sapiens OX=9606 GN=ERAP1	40.72	31.92	1.28	0.00651
G8JLG2	CDSN OS=Homo sapiens OX=9606 GN=CDSN PE=1 SV=1	126.01	78.81	1.60	0.00658
P52306	Isoform 4 of Rap1 GTPase-GDP dissociation stimulator 1 OS=Homo sapiens OX=9606 GN=RAP1GDS1	2217.84	1630.59	1.36	0.00663
A8MXB7	Sorting nexin-24 OS=Homo sapiens OX=9606 GN=SNX24 PE=1 SV=1	54.56	37.15	1.47	0.00664
P20472	Parvalbumin alpha OS=Homo sapiens OX=9606 GN=PVALB PE=1 SV=2	2478.41	1017.20	2.44	0.00666
P05154	Plasma serine protease inhibitor OS=Homo sapiens OX=9606 GN=SERPINA5 PE=1 SV=3	52.47	32.42	1.62	0.00686
Q9Y5F8	Protocadherin gamma-B7 OS=Homo sapiens OX=9606 GN=PCDHGB7 PE=2 SV=1	18.30	14.01	1.31	0.00687
P48436	Transcription factor SOX-9 OS=Homo sapiens OX=9606 GN=SOX9 PE=1 SV=1	64.74	49.97	1.30	0.00689
P17812	CTP synthase 1 OS=Homo sapiens OX=9606 GN=CTPS1 PE=1 SV=2	372.46	300.89	1.24	0.00697
Q9BRT3	Migration and invasion enhancer 1 OS=Homo sapiens OX=9606 GN=MIEN1 PE=1 SV=1	374.40	266.73	1.40	0.00719
Q9Y6K9	Isoform 2 of NF-kappa-B essential modulator OS=Homo sapiens OX=9606 GN=IKBKG	320.50	251.71	1.27	0.00721
P42574	Caspase-3 OS=Homo sapiens OX=9606 GN=CASP3 PE=1 SV=2	35.44	24.03	1.47	0.00733
Q9BRQ8	Apoptosis-inducing factor 2 OS=Homo sapiens OX=9606 GN=AIFM2 PE=1 SV=1	16.21	11.42	1.42	0.00744
O75821	Eukaryotic translation initiation factor 3 subunit G OS=Homo sapiens OX=9606 GN=EIF3G PE=1 SV=2	802.62	656.36	1.22	0.00772
P04003	C4b-binding protein alpha chain OS=Homo sapiens OX=9606 GN=C4BPA PE=1 SV=2	178.85	146.54	1.22	0.00793
Q9BU02	Thiamine-triphosphatase OS=Homo sapiens OX=9606 GN=THTPA PE=1 SV=3	135.79	109.04	1.25	0.00821
Q8NDI1	Isoform 2 of EH domain-binding protein 1 OS=Homo sapiens OX=9606 GN=EHBP1	86.38	64.60	1.34	0.00849

Q32MZ4	Isoform 2 of Leucine-rich repeat flightless-interacting protein 1 OS=Homo sapiens OX=9606 GN=LRRFIP1	406.39	320.01	1.27	0.00869
Q9Y5B0	RNA polymerase II subunit A C-terminal domain phosphatase OS=Homo sapiens OX=9606 GN=CTDP1 PE=1 SV=3	60.00	44.41	1.35	0.00870
Q59EK9	Isoform 2 of RUN domain-containing protein 3A OS=Homo sapiens OX=9606 GN=RUNDC3A	129.38	88.76	1.46	0.00873
O95433	Activator of 90 kDa heat shock protein ATPase homolog 1 OS=Homo sapiens OX=9606 GN=AHSA1 PE=1 SV=1	1320.18	944.06	1.40	0.00895
A0A0A0MSS3	SLAIN motif-containing protein 1 OS=Homo sapiens OX=9606 GN=SLAIN1 PE=1 SV=1	32.99	18.03	1.83	0.00896
P62166	Neuronal calcium sensor 1 OS=Homo sapiens OX=9606 GN=NCS1 PE=1 SV=2	124.19	91.92	1.35	0.00898
Q8WWX9	Selenoprotein M OS=Homo sapiens OX=9606 GN=SELENOM PE=1 SV=3	61.70	47.38	1.30	0.00926
P02654	Apolipoprotein C-I OS=Homo sapiens OX=9606 GN=APOC1 PE=1 SV=1	596.18	278.36	2.14	0.00931
P45974	Ubiquitin carboxyl-terminal hydrolase 5 OS=Homo sapiens OX=9606 GN=USP5 PE=1 SV=2	2537.62	2076.73	1.22	0.00934
Q96BH1	E3 ubiquitin-protein ligase RNF25 OS=Homo sapiens OX=9606 GN=RNF25 PE=1 SV=1	74.03	59.99	1.23	0.00935
Q8IYB5	Isoform 2 of Stromal membrane-associated protein 1 OS=Homo sapiens OX=9606 GN=SMAP1	238.94	174.64	1.37	0.00942
Q96P47	Isoform 4 of Arf-GAP with GTPase, ANK repeat and PH domain-containing protein 3 OS=Homo sapiens OX=9606 GN=AGAP3	179.44	144.58	1.24	0.00975
D3DSM0	Integrin beta OS=Homo sapiens OX=9606 GN=ITGB2 PE=1 SV=1	49.29	39.12	1.26	0.00981
Q9NRX4	14 kDa phosphohistidine phosphatase OS=Homo sapiens OX=9606 GN=PHPT1 PE=1 SV=1	1108.29	619.01	1.79	0.00988
Q96RR4	Calcium/calmodulin-dependent protein kinase kinase 2 OS=Homo sapiens OX=9606 GN=CAMKK2 PE=1 SV=2	19.25	15.08	1.28	0.00997
Q15102	Platelet-activating factor acetylhydrolase IB subunit gamma OS=Homo sapiens OX=9606 GN=PAFAH1B3 PE=1 SV=1	780.04	522.27	1.49	0.00998
Q96DG6	Carboxymethylenebutenolidase homolog OS=Homo sapiens OX=9606 GN=CMBL PE=1 SV=1	403.88	278.56	1.45	0.01008
P15374	Ubiquitin carboxyl-terminal hydrolase isozyme L3 OS=Homo sapiens OX=9606 GN=UCHL3 PE=1 SV=1	227.05	156.66	1.45	0.01010
Q86TP1	Exopolyphosphatase PRUNE1 OS=Homo sapiens OX=9606 GN=PRUNE1 PE=1 SV=2	227.16	178.09	1.28	0.01022
Q5T7Y6	Protein S100 OS=Homo sapiens OX=9606 GN=S100A1 PE=1 SV=1	800.46	411.02	1.95	0.01028
P49773	Histidine triad nucleotide-binding protein 1 OS=Homo sapiens OX=9606 GN=HINT1 PE=1 SV=2	1325.92	673.28	1.97	0.01029
Q96S19	Methyltransferase-like 26 OS=Homo sapiens OX=9606 GN=METTL26 PE=1 SV=2	133.15	105.62	1.26	0.01046
Q9BWD1	Acetyl-CoA acetyltransferase, cytosolic OS=Homo sapiens OX=9606 GN=ACAT2 PE=1 SV=2	1137.07	859.36	1.32	0.01052

Q9UNZ2	Isoform 3 of NSFL1 cofactor p47 OS=Homo sapiens OX=9606 GN=NSFL1C	2258.72	1688.00	1.34	0.01053
Q5QJ74	Tubulin-specific chaperone cofactor E-like protein OS=Homo sapiens OX=9606 GN=TBCEL PE=1 SV=2	54.46	41.05	1.33	0.01058
Q13526	Peptidyl-prolyl cis-trans isomerase NIMA-interacting 1 OS=Homo sapiens OX=9606 GN=PIN1 PE=1 SV=1	1031.81	719.37	1.43	0.01061
P48507	Glutamate--cysteine ligase regulatory subunit OS=Homo sapiens OX=9606 GN=GCLM PE=1 SV=1	261.02	202.56	1.29	0.01062
O75832	26S proteasome non-ATPase regulatory subunit 10 OS=Homo sapiens OX=9606 GN=PSMD10 PE=1 SV=1	627.63	494.69	1.27	0.01077
P51636	Caveolin-2 OS=Homo sapiens OX=9606 GN=CAV2 PE=1 SV=2	116.44	85.53	1.36	0.01078
O76003	Glutaredoxin-3 OS=Homo sapiens OX=9606 GN=GLRX3 PE=1 SV=2	466.75	356.01	1.31	0.01078
O75312	Zinc finger protein ZPR1 OS=Homo sapiens OX=9606 GN=ZPR1 PE=1 SV=1	42.66	28.75	1.48	0.01081
P54725	Isoform 3 of UV excision repair protein RAD23 homolog A OS=Homo sapiens OX=9606 GN=RAD23A	591.74	457.20	1.29	0.01098
P0DI81	Isoform 3 of Trafficking protein particle complex subunit 2 OS=Homo sapiens OX=9606 GN=TRAPPC2	67.81	51.34	1.32	0.01099
Q92696	Geranylgeranyl transferase type-2 subunit alpha OS=Homo sapiens OX=9606 GN=RABGGTA PE=1 SV=2	550.76	451.60	1.22	0.01109
Q13107	Ubiquitin carboxyl-terminal hydrolase 4 OS=Homo sapiens OX=9606 GN=USP4 PE=1 SV=3	226.73	185.92	1.22	0.01115
Q6FI81	Anamorsin OS=Homo sapiens OX=9606 GN=CIAPIN1 PE=1 SV=2	868.18	605.79	1.43	0.01122
Q8TCC7	Isoform 2 of Solute carrier family 22 member 8 OS=Homo sapiens OX=9606 GN=SLC22A8	32.50	16.95	1.92	0.01126
Q5SYC1	Clavesin-2 OS=Homo sapiens OX=9606 GN=CLVS2 PE=2 SV=1	301.23	211.87	1.42	0.01128
Q15785	Mitochondrial import receptor subunit TOM34 OS=Homo sapiens OX=9606 GN=TOMM34 PE=1 SV=2	483.95	402.79	1.20	0.01128
Q8TDH9	Biogenesis of lysosome-related organelles complex 1 subunit 5 OS=Homo sapiens OX=9606 GN=BLOC1S5 PE=1 SV=1	53.76	42.00	1.28	0.01128
Q6NW29	RWD domain-containing protein 4 OS=Homo sapiens OX=9606 GN=RWDD4 PE=1 SV=3	121.98	90.50	1.35	0.01149
P40123	Adenylyl cyclase-associated protein 2 OS=Homo sapiens OX=9606 GN=CAP2 PE=1 SV=1	842.21	692.64	1.22	0.01153
Q9UNI6	Dual specificity protein phosphatase 12 OS=Homo sapiens OX=9606 GN=DUSP12 PE=1 SV=1	37.12	28.13	1.32	0.01162
Q9NWV4	UPF0587 protein C1orf123 OS=Homo sapiens OX=9606 GN=C1orf123 PE=1 SV=1	192.40	128.29	1.50	0.01177
Q96PE1	Adhesion G protein-coupled receptor A2 OS=Homo sapiens OX=9606 GN=ADGRA2 PE=1 SV=2	400.09	309.54	1.29	0.01177
Q9UNN5	FAS-associated factor 1 OS=Homo sapiens OX=9606 GN=FAF1 PE=1 SV=2	514.75	428.62	1.20	0.01182
Q68CZ2	Tensin-3 OS=Homo sapiens OX=9606 GN=TNS3 PE=1 SV=2	453.91	371.91	1.22	0.01185

Q14515	SPARC-like protein 1 OS=Homo sapiens OX=9606 GN=SPARCL1 PE=1 SV=2	552.86	420.47	1.31	0.01188
Q15555	Isoform 5 of Microtubule-associated protein RP/EB family member 2 OS=Homo sapiens OX=9606 GN=MAPRE2	1854.81	1469.12	1.26	0.01191
P06732	Creatine kinase M-type OS=Homo sapiens OX=9606 GN=CKM PE=1 SV=2	336.46	177.58	1.89	0.01205
A0A0A0MSS8	Aldo-keto reductase family 1 member C3 OS=Homo sapiens OX=9606 GN=AKR1C3 PE=1 SV=1	5088.56	3237.40	1.57	0.01209
O43399	Isoform 7 of Tumor protein D54 OS=Homo sapiens OX=9606 GN=TPD52L2	642.20	487.69	1.32	0.01245
Q92882	Osteoclast-stimulating factor 1 OS=Homo sapiens OX=9606 GN=OSTF1 PE=1 SV=2	257.38	206.19	1.25	0.01257
Q01469	Fatty acid-binding protein 5 OS=Homo sapiens OX=9606 GN=FABP5 PE=1 SV=3	2053.51	1176.32	1.75	0.01262
P43487	Ran-specific GTPase-activating protein OS=Homo sapiens OX=9606 GN=RANBP1 PE=1 SV=1	1344.99	875.65	1.54	0.01280
P35754	Glutaredoxin-1 OS=Homo sapiens OX=9606 GN=GLRX PE=1 SV=2	27.47	20.68	1.33	0.01285
E5RJR5	S-phase kinase-associated protein 1 OS=Homo sapiens OX=9606 GN=SKP1 PE=1 SV=1	2046.62	1575.94	1.30	0.01306
O15498	Synaptobrevin homolog YKT6 OS=Homo sapiens OX=9606 GN=YKT6 PE=1 SV=1	655.96	467.21	1.40	0.01312
Q6GMV3	Putative peptidyl-tRNA hydrolase PTRHD1 OS=Homo sapiens OX=9606 GN=PTRHD1 PE=1 SV=1	472.66	336.48	1.40	0.01328
P10599	Thioredoxin OS=Homo sapiens OX=9606 GN=TXN PE=1 SV=3	2044.74	1314.22	1.56	0.01340
Q9BUT1	3-hydroxybutyrate dehydrogenase type 2 OS=Homo sapiens OX=9606 GN=BDH2 PE=1 SV=2	1449.11	1073.46	1.35	0.01342
Q8IVM0	Isoform 2 of Coiled-coil domain-containing protein 50 OS=Homo sapiens OX=9606 GN=CCDC50	329.00	233.10	1.41	0.01345
P62256	Ubiquitin-conjugating enzyme E2 H OS=Homo sapiens OX=9606 GN=UBE2H PE=1 SV=1	294.12	211.92	1.39	0.01359
P06753	Isoform 6 of Tropomyosin alpha-3 chain OS=Homo sapiens OX=9606 GN=TPM3	125.65	89.61	1.40	0.01373
P68032	Actin, alpha cardiac muscle 1 OS=Homo sapiens OX=9606 GN=ACTC1 PE=1 SV=1	4806.37	3608.74	1.33	0.01384
P12429	Annexin A3 OS=Homo sapiens OX=9606 GN=ANXA3 PE=1 SV=3	944.41	763.07	1.24	0.01416
Q96IU4	Protein ABHD14B OS=Homo sapiens OX=9606 GN=ABHD14B PE=1 SV=1	487.88	288.37	1.69	0.01417
Q96NT1	Nucleosome assembly protein 1-like 5 OS=Homo sapiens OX=9606 GN=NAP1L5 PE=1 SV=1	420.40	268.61	1.57	0.01436
P04259	Keratin, type II cytoskeletal 6B OS=Homo sapiens OX=9606 GN=KRT6B PE=1 SV=5	361.94	208.21	1.74	0.01447
Q8NFU3	Thiosulfate:glutathione sulfurtransferase OS=Homo sapiens OX=9606 GN=TSTD1 PE=1 SV=3	481.54	316.75	1.52	0.01448
P62942	Peptidyl-prolyl cis-trans isomerase FKBP1A OS=Homo sapiens OX=9606 GN=FKBP1A PE=1 SV=2	209.95	120.26	1.75	0.01491
Q6UUV9	CREB-regulated transcription coactivator 1 OS=Homo sapiens OX=9606 GN=CRTC1 PE=1 SV=2	83.47	54.48	1.53	0.01508

A0AVT1	Ubiquitin-like modifier-activating enzyme 6 OS=Homo sapiens OX=9606 GN=UBA6 PE=1 SV=1	1134.19	862.00	1.32	0.01533
Q8WUY3	Isoform 3 of Protein prune homolog 2 OS=Homo sapiens OX=9606 GN=PRUNE2	194.34	153.39	1.27	0.01535
P00441	Superoxide dismutase [Cu-Zn] OS=Homo sapiens OX=9606 GN=SOD1 PE=1 SV=2	8941.29	4901.83	1.82	0.01536
O00287	Regulatory factor X-associated protein OS=Homo sapiens OX=9606 GN=RFXAP PE=1 SV=1	11.53	7.35	1.57	0.01536
P50225	Sulfotransferase 1A1 OS=Homo sapiens OX=9606 GN=SULT1A1 PE=1 SV=3	1246.23	687.94	1.81	0.01557
A0A0B4J2C3	Translationally-controlled tumor protein OS=Homo sapiens OX=9606 GN=TPT1 PE=1 SV=1	1235.16	822.45	1.50	0.01565
Q9Y3C6	Peptidyl-prolyl cis-trans isomerase-like 1 OS=Homo sapiens OX=9606 GN=PPIL1 PE=1 SV=1	1666.88	1372.13	1.21	0.01575
Q9NZN3	EH domain-containing protein 3 OS=Homo sapiens OX=9606 GN=EHD3 PE=1 SV=2	1388.28	1149.33	1.21	0.01590
P55283	Cadherin-4 OS=Homo sapiens OX=9606 GN=CDH4 PE=2 SV=2	32.24	25.09	1.28	0.01591
Q8WUW1	Isoform 2 of Protein BRICK1 OS=Homo sapiens OX=9606 GN=BRK1	71.36	54.48	1.31	0.01610
P31946	14-3-3 protein beta/alpha OS=Homo sapiens OX=9606 GN=YWHAB PE=1 SV=3	3109.42	2450.13	1.27	0.01634
Q9BVJ7	Dual specificity protein phosphatase 23 OS=Homo sapiens OX=9606 GN=DUSP23 PE=1 SV=1	167.45	137.47	1.22	0.01645
Q13542	Eukaryotic translation initiation factor 4E-binding protein 2 OS=Homo sapiens OX=9606 GN=EIF4EBP2 PE=1 SV=1	18.90	11.30	1.67	0.01650
P31939	Bifunctional purine biosynthesis protein PURH OS=Homo sapiens OX=9606 GN=ATIC PE=1 SV=3	3324.72	2451.98	1.36	0.01663
F8WBA3	Serine/threonine-protein kinase OS=Homo sapiens OX=9606 GN=PRKD1 PE=1 SV=1	21.28	17.00	1.25	0.01666
O00764	Pyridoxal kinase OS=Homo sapiens OX=9606 GN=PDXK PE=1 SV=1	878.05	692.86	1.27	0.01667
Q13200	26S proteasome non-ATPase regulatory subunit 2 OS=Homo sapiens OX=9606 GN=PSMD2 PE=1 SV=3	5510.45	4577.83	1.20	0.01678
Q9NPA8	Transcription and mRNA export factor ENY2 OS=Homo sapiens OX=9606 GN=ENY2 PE=1 SV=1	223.02	174.99	1.27	0.01687
Q15404	Ras suppressor protein 1 OS=Homo sapiens OX=9606 GN=RSU1 PE=1 SV=3	775.98	646.36	1.20	0.01694
Q9Y5N5	Methyltransferase N6AMT1 OS=Homo sapiens OX=9606 GN=N6AMT1 PE=1 SV=4	63.32	46.73	1.36	0.01699
P23763	Vesicle-associated membrane protein 1 OS=Homo sapiens OX=9606 GN=VAMP1 PE=1 SV=1	157.11	117.05	1.34	0.01719
P09493	Isoform 9 of Tropomyosin alpha-1 chain OS=Homo sapiens OX=9606 GN=TPM1	5738.17	3811.32	1.51	0.01721
Q16515	Acid-sensing ion channel 2 OS=Homo sapiens OX=9606 GN=ASIC2 PE=1 SV=1	37.57	30.30	1.24	0.01722
Q8WVJ2	NudC domain-containing protein 2 OS=Homo sapiens OX=9606 GN=NUDCD2 PE=1 SV=1	281.85	166.20	1.70	0.01737
J3KN67	Tropomyosin alpha-3 chain OS=Homo sapiens OX=9606 GN=TPM3 PE=1 SV=1	2389.74	1563.25	1.53	0.01743

P07738	Bisphosphoglycerate mutase OS=Homo sapiens OX=9606 GN=BPGM PE=1 SV=2	543.27	427.15	1.27	0.01747
P0C6S8	Leucine-rich repeat and immunoglobulin-like domain-containing nogo receptor-interacting protein 3 OS=Homo sapiens OX=9606 GN=LINGO3 PE=3 SV=1		29.85	22.76	1.31173004
E9PAV3	Nascent polypeptide-associated complex subunit alpha, muscle-specific form OS=Homo sapiens OX=9606 GN=NACA PE=1 SV=1	541.53	402.79	1.34	0.01754
Q6PUV4	Complexin-2 OS=Homo sapiens OX=9606 GN=CPLX2 PE=2 SV=2	213.96	131.18	1.63	0.01767
Q96EI5	Isoform 2 of Transcription elongation factor A protein-like 4 OS=Homo sapiens OX=9606 GN=TCEAL4	304.94	213.65	1.43	0.01769
O75487	Glypican-4 OS=Homo sapiens OX=9606 GN=GPC4 PE=1 SV=4	39.10	20.86	1.87	0.01785
Q99733	Isoform 2 of Nucleosome assembly protein 1-like 4 OS=Homo sapiens OX=9606 GN=NAP1L4	2060.27	1689.41	1.22	0.01786
P52594	Isoform 4 of Arf-GAP domain and FG repeat-containing protein 1 OS=Homo sapiens OX=9606 GN=AGFG1	600.42	494.60	1.21	0.01788
P52434	DNA-directed RNA polymerases I, II, and III subunit RPABC3 OS=Homo sapiens OX=9606 GN=POLR2H PE=1 SV=4	58.16	42.61	1.37	0.01789
Q96RU3	Formin-binding protein 1 OS=Homo sapiens OX=9606 GN=FNBP1 PE=1 SV=2	199.10	158.72	1.25	0.01846
P30043	Flavin reductase (NADPH) OS=Homo sapiens OX=9606 GN=BLVRB PE=1 SV=3	679.07	533.46	1.27	0.01847
O95777	U6 snRNA-associated Sm-like protein LSm8 OS=Homo sapiens OX=9606 GN=LSM8 PE=1 SV=3	526.83	419.00	1.26	0.01848
O94888	UBX domain-containing protein 7 OS=Homo sapiens OX=9606 GN=UBXN7 PE=1 SV=2	537.48	406.04	1.32	0.01851
Q96FC7	Phytanoyl-CoA hydroxylase-interacting protein-like OS=Homo sapiens OX=9606 GN=PHYHIPL PE=1 SV=3	3052.33	2221.45	1.37	0.01868
Q9Y2Z0	Protein SGT1 homolog OS=Homo sapiens OX=9606 GN=SUGT1 PE=1 SV=3	889.01	636.82	1.40	0.01871
P13747	HLA class I histocompatibility antigen, alpha chain E OS=Homo sapiens OX=9606 GN=HLA-E PE=1 SV=4	66.11	48.76	1.36	0.01884
P46934	Isoform 4 of E3 ubiquitin-protein ligase NEDD4 OS=Homo sapiens OX=9606 GN=NEDD4	19.32	14.43	1.34	0.01893
P33241	Isoform 3 of Lymphocyte-specific protein 1 OS=Homo sapiens OX=9606 GN=LSP1	68.78	46.00	1.50	0.01901
Q96EB1	Elongator complex protein 4 OS=Homo sapiens OX=9606 GN=ELP4 PE=1 SV=2	83.97	68.63	1.22	0.01904
O43765	Small glutamine-rich tetratricopeptide repeat-containing protein alpha OS=Homo sapiens OX=9606 GN=SGTA PE=1 SV=1	1357.54	1074.89	1.26	0.01906
Q08188	Protein-glutamine gamma-glutamyltransferase E OS=Homo sapiens OX=9606 GN=TGM3 PE=1 SV=4	46.19	34.73	1.33	0.01917
P16402	Histone H1.3 OS=Homo sapiens OX=9606 GN=HIST1H1D PE=1 SV=2	82.42	65.56	1.26	0.01924
P14324	Farnesyl pyrophosphate synthase OS=Homo sapiens OX=9606 GN=FDPS PE=1 SV=4	944.67	660.04	1.43	0.01939
Q8WVV4	Isoform 1 of Protein POF1B OS=Homo sapiens OX=9606 GN=POF1B	152.81	75.08	2.04	0.01951

J3KPJ3	Calcium/calmodulin-dependent protein kinase kinase 1 OS=Homo sapiens OX=9606 GN=CAMKK1 PE=1 SV=1	264.32	210.62	1.25	0.01971
Q9UN36	Protein NDRG2 OS=Homo sapiens OX=9606 GN=NDRG2 PE=1 SV=2	1514.06	1220.32	1.24	0.01987
Q7LG56	Isoform 6 of Ribonucleoside-diphosphate reductase subunit M2 B OS=Homo sapiens OX=9606 GN=RRM2B	420.22	339.50	1.24	0.01993
Q9H2J4	Phosducin-like protein 3 OS=Homo sapiens OX=9606 GN=PDCL3 PE=1 SV=1	115.08	81.65	1.41	0.01997
Q6NZI2	Caveolae-associated protein 1 OS=Homo sapiens OX=9606 GN=CAVIN1 PE=1 SV=1	1133.86	913.06	1.24	0.02000
F5H211	Ataxin-3 OS=Homo sapiens OX=9606 GN=ATXN3 PE=1 SV=2	36.80	25.58	1.44	0.02005
O60493	Sorting nexin-3 OS=Homo sapiens OX=9606 GN=SNX3 PE=1 SV=3	317.87	239.98	1.32	0.02008
P51668	Ubiquitin-conjugating enzyme E2 D1 OS=Homo sapiens OX=9606 GN=UBE2D1 PE=1 SV=1	173.55	130.03	1.33	0.02017
P08397	Porphobilinogen deaminase OS=Homo sapiens OX=9606 GN=HMBS PE=1 SV=2	11.04	7.30	1.51	0.02027
Q9GZP9	Derlin-2 OS=Homo sapiens OX=9606 GN=DERL2 PE=1 SV=1	77.25	60.74	1.27	0.02029
Q9P2X3	Protein IMPACT OS=Homo sapiens OX=9606 GN=IMPACT PE=1 SV=2	174.19	137.56	1.27	0.02031
Q9HBL8	NmrA-like family domain-containing protein 1 OS=Homo sapiens OX=9606 GN=NMRAL1 PE=1 SV=1	132.05	95.42	1.38	0.02032
Q14643	Inositol 1,4,5-trisphosphate receptor type 1 OS=Homo sapiens OX=9606 GN=ITPR1 PE=1 SV=3	14.20	10.66	1.33	0.02056
Q8N806	Putative E3 ubiquitin-protein ligase UBR7 OS=Homo sapiens OX=9606 GN=UBR7 PE=1 SV=2	342.00	277.05	1.23	0.02058
Q6ZV70	LanC-like protein 3 OS=Homo sapiens OX=9606 GN=LANCL3 PE=2 SV=2	9.15	4.55	2.01	0.02059
Q9Y2Y0	ADP-ribosylation factor-like protein 2-binding protein OS=Homo sapiens OX=9606 GN=ARL2BP PE=1 SV=1	126.64	98.19	1.29	0.02062
Q96NL8	Protein C8orf37 OS=Homo sapiens OX=9606 GN=C8orf37 PE=1 SV=1	205.81	132.73	1.55	0.02063
Q05209	Tyrosine-protein phosphatase non-receptor type 12 OS=Homo sapiens OX=9606 GN=PTPN12 PE=1 SV=3	35.49	27.45	1.29	0.02085
P62328	Thymosin beta-4 OS=Homo sapiens OX=9606 GN=TMSB4X PE=1 SV=2	295.95	153.78	1.92	0.02087
Q5SQI0	Alpha-tubulin N-acetyltransferase 1 OS=Homo sapiens OX=9606 GN=ATAT1 PE=1 SV=1	231.81	175.67	1.32	0.02093
I3L504	Eukaryotic translation initiation factor 5A-1 OS=Homo sapiens OX=9606 GN=EIF5A PE=1 SV=1	1338.85	956.16	1.40	0.02110
P01040	Cystatin-A OS=Homo sapiens OX=9606 GN=CSTA PE=1 SV=1	198.20	141.32	1.40	0.02124
Q9UHD9	Ubiquilin-2 OS=Homo sapiens OX=9606 GN=UBQLN2 PE=1 SV=2	988.40	801.92	1.23	0.02130
A0A1B0GUS4	Ubiquitin-conjugating enzyme E2 L5 OS=Homo sapiens OX=9606 GN=UBE2L5 PE=2 SV=1	43.70	29.83	1.47	0.02141
Q969E8	Pre-rRNA-processing protein TSR2 homolog OS=Homo sapiens OX=9606 GN=TSR2 PE=1 SV=1	310.82	236.58	1.31	0.02154

O15145	Actin-related protein 2/3 complex subunit 3 OS=Homo sapiens OX=9606 GN=ARPC3 PE=1 SV=3	334.10	276.82	1.21	0.02161
Q9BXS9	Solute carrier family 26 member 6 OS=Homo sapiens OX=9606 GN=SLC26A6 PE=1 SV=1	9.46	6.65	1.42	0.02163
Q9UI26	Isoform 2 of Importin-11 OS=Homo sapiens OX=9606 GN=IPO11	99.11	76.42	1.30	0.02165
O75223	Gamma-glutamylcyclotransferase OS=Homo sapiens OX=9606 GN=GGCT PE=1 SV=1	838.28	511.58	1.64	0.02171
P25391	Laminin subunit alpha-1 OS=Homo sapiens OX=9606 GN=LAMA1 PE=1 SV=2	24.89	20.10	1.24	0.02178
Q9UK45	U6 snRNA-associated Sm-like protein LSm7 OS=Homo sapiens OX=9606 GN=LSM7 PE=1 SV=1	432.90	345.80	1.25	0.02185
Q04323	UBX domain-containing protein 1 OS=Homo sapiens OX=9606 GN=UBXN1 PE=1 SV=2	662.96	485.60	1.37	0.02188
A6NGJ0	Dynein light chain Tctex-type 3 OS=Homo sapiens OX=9606 GN=DYNLT3 PE=1 SV=1	8.39	4.77	1.76	0.02209
Q9HAB8	Phosphopantothenate--cysteine ligase OS=Homo sapiens OX=9606 GN=PPCS PE=1 SV=2	456.39	327.88	1.39	0.02219
Q96GX2	Ataxin-7-like protein 3B OS=Homo sapiens OX=9606 GN=ATXN7L3B PE=1 SV=2	51.49	40.80	1.26	0.02219
Q99961	Endophilin-A2 OS=Homo sapiens OX=9606 GN=SH3GL1 PE=1 SV=1	565.35	453.68	1.25	0.02228
P31751	RAC-beta serine/threonine-protein kinase OS=Homo sapiens OX=9606 GN=AKT2 PE=1 SV=2	118.23	97.80	1.21	0.02233
O95817	BAG family molecular chaperone regulator 3 OS=Homo sapiens OX=9606 GN=BAG3 PE=1 SV=3	1493.09	1144.22	1.30	0.02252
O14933	Ubiquitin/ISG15-conjugating enzyme E2 L6 OS=Homo sapiens OX=9606 GN=UBE2L6 PE=1 SV=4	54.85	40.67	1.35	0.02254
Q8N9V3	WD repeat, SAM and U-box domain-containing protein 1 OS=Homo sapiens OX=9606 GN=WDSUB1 PE=1 SV=3	48.04	32.99	1.46	0.02270
Q9UJY1	Heat shock protein beta-8 OS=Homo sapiens OX=9606 GN=HSPB8 PE=1 SV=1	1119.54	905.68	1.24	0.02284
Q9NR19	Isoform 2 of Acetyl-coenzyme A synthetase, cytoplasmic OS=Homo sapiens OX=9606 GN=ACSS2	264.03	211.87	1.25	0.02297
P34913	Bifunctional epoxide hydrolase 2 OS=Homo sapiens OX=9606 GN=EPHX2 PE=1 SV=2	3171.92	2341.45	1.35	0.02298
P42773	Cyclin-dependent kinase 4 inhibitor C OS=Homo sapiens OX=9606 GN=CDKN2C PE=1 SV=1	62.40	45.01	1.39	0.02301
Q8TAA9	Vang-like protein 1 OS=Homo sapiens OX=9606 GN=VANGL1 PE=1 SV=1	41.58	30.67	1.36	0.02301
P20039	HLA class II histocompatibility antigen, DRB1-11 beta chain OS=Homo sapiens OX=9606 GN=HLA-DRB1 PE=1 SV=1	294.89	130.80	2.25	0.02316
Q13158	FAS-associated death domain protein OS=Homo sapiens OX=9606 GN=FADD PE=1 SV=1	118.59	85.52	1.39	0.02337
Q14118	Dystroglycan OS=Homo sapiens OX=9606 GN=DAG1 PE=1 SV=2	436.47	355.64	1.23	0.02420

P51965	Ubiquitin-conjugating enzyme E2 E1 OS=Homo sapiens OX=9606 GN=UBE2E1 PE=1 SV=1	369.01	239.14	1.54	0.02422
P20591	Interferon-induced GTP-binding protein Mx1 OS=Homo sapiens OX=9606 GN=MX1 PE=1 SV=4	70.00	57.46	1.22	0.02423
P43250	Isoform GRK6B of G protein-coupled receptor kinase 6 OS=Homo sapiens OX=9606 GN=GRK6	34.21	26.81	1.28	0.02435
Q7Z6K5	Isoform C15orf38-AP3S2 of Arpin OS=Homo sapiens OX=9606 GN=ARPIN	561.07	426.69	1.31	0.02442
A6NKN8	Purkinje cell protein 4-like protein 1 OS=Homo sapiens OX=9606 GN=PCP4L1 PE=3 SV=3	251.18	145.43	1.73	0.02462
A0A2R8YFE2	Actin, cytoplasmic 1 (Fragment) OS=Homo sapiens OX=9606 GN=ACTB PE=1 SV=1	211.72	160.99	1.32	0.02468
Q9BTV5	Fibronectin type III and SPRY domain-containing protein 1 OS=Homo sapiens OX=9606 GN=FSD1 PE=1 SV=1	280.54	228.71	1.23	0.02473
P13647	Keratin, type II cytoskeletal 5 OS=Homo sapiens OX=9606 GN=KRT5 PE=1 SV=3	4832.84	2921.26	1.65	0.02473
Q9NUP1	Biogenesis of lysosome-related organelles complex 1 subunit 4 OS=Homo sapiens OX=9606 GN=BLOC1S4 PE=1 SV=1	133.99	110.25	1.22	0.02490
Q86WV7	CCDC43 protein OS=Homo sapiens OX=9606 GN=CCDC43 PE=1 SV=1	276.11	179.55	1.54	0.02493
Q93045	Isoform 2 of Stathmin-2 OS=Homo sapiens OX=9606 GN=STMN2	47.31	39.33	1.20	0.02551
Q5JS54	Isoform 2 of Proteasome assembly chaperone 4 OS=Homo sapiens OX=9606 GN=PSMG4	9.78	6.98	1.40	0.02556
O76070	Gamma-synuclein OS=Homo sapiens OX=9606 GN=SNCG PE=1 SV=2	3826.18	2568.81	1.49	0.02565
P40855	Peroxisomal biogenesis factor 19 OS=Homo sapiens OX=9606 GN=PEX19 PE=1 SV=1	664.76	477.10	1.39	0.02573
Q8WV99	AN1-type zinc finger protein 2B OS=Homo sapiens OX=9606 GN=ZFAND2B PE=1 SV=1	26.35	21.03	1.25	0.02601
Q9UBE0	SUMO-activating enzyme subunit 1 OS=Homo sapiens OX=9606 GN=SAE1 PE=1 SV=1	1328.42	976.11	1.36	0.02615
A6NDU8	UPF0600 protein C5orf51 OS=Homo sapiens OX=9606 GN=C5orf51 PE=1 SV=1	237.13	180.63	1.31	0.02620
O60443	Gasdermin-E OS=Homo sapiens OX=9606 GN=GSDME PE=1 SV=2	172.99	135.62	1.28	0.02642
A0MZ66	Isoform 3 of Shootin-1 OS=Homo sapiens OX=9606 GN=SHTN1	774.52	609.52	1.27	0.02644
Q712K3	Ubiquitin-conjugating enzyme E2 R2 OS=Homo sapiens OX=9606 GN=UBE2R2 PE=1 SV=1	125.34	91.63	1.37	0.02652
Q96K17	Transcription factor BTF3 homolog 4 OS=Homo sapiens OX=9606 GN=BTF3L4 PE=1 SV=1	118.58	83.83	1.41	0.02659
P84085	ADP-ribosylation factor 5 OS=Homo sapiens OX=9606 GN=ARF5 PE=1 SV=2	489.18	398.02	1.23	0.02666
Q9UI15	Transgelin-3 OS=Homo sapiens OX=9606 GN=TAGLN3 PE=1 SV=2	2887.73	2231.51	1.29	0.02692
Q8WZA0	Isoform 2 of Protein LZIC OS=Homo sapiens OX=9606 GN=LZIC	875.67	513.65	1.70	0.02697
Q9UPR0	Inactive phospholipase C-like protein 2 OS=Homo sapiens OX=9606 GN=PLCL2 PE=1 SV=2	71.79	59.11	1.21	0.02699

P81605	Dermcidin OS=Homo sapiens OX=9606 GN=DCD PE=1 SV=2	344.22	228.39	1.51	0.02764
Q15056	Eukaryotic translation initiation factor 4H OS=Homo sapiens OX=9606 GN=EIF4H PE=1 SV=5	1466.23	1015.93	1.44	0.02798
U3KQP1	Asparagine synthetase domain-containing protein 1 (Fragment) OS=Homo sapiens OX=9606 GN=ASNSD1 PE=1 SV=1	175.68	97.40	1.80	0.02798
E9PGN7	Plasma protease C1 inhibitor OS=Homo sapiens OX=9606 GN=SERPING1 PE=1 SV=1	413.30	290.92	1.42	0.02801
Q13228	Isoform 4 of Methanethiol oxidase OS=Homo sapiens OX=9606 GN=SELENBP1	3216.48	2278.79	1.41	0.02813
P07992	DNA excision repair protein ERCC-1 OS=Homo sapiens OX=9606 GN=ERCC1 PE=1 SV=1	61.09	42.17	1.45	0.02819
P55899	IgG receptor FcRn large subunit p51 OS=Homo sapiens OX=9606 GN=FCGRT PE=1 SV=1	30.23	19.50	1.55	0.02823
P28676	Grancalcin OS=Homo sapiens OX=9606 GN=GCA PE=1 SV=2	574.29	465.20	1.23	0.02870
J3KR44	Ubiquitin thioesterase OS=Homo sapiens OX=9606 GN=OTUB1 PE=1 SV=2	789.81	587.72	1.34	0.02879
O60927	E3 ubiquitin-protein ligase PPP1R11 OS=Homo sapiens OX=9606 GN=PPP1R11 PE=1 SV=1	156.67	126.28	1.24	0.02885
P52564	Dual specificity mitogen-activated protein kinase kinase 6 OS=Homo sapiens OX=9606 GN=MAP2K6 PE=1 SV=1	755.76	564.72	1.34	0.02923
P45985	Isoform 2 of Dual specificity mitogen-activated protein kinase kinase 4 OS=Homo sapiens OX=9606 GN=MAP2K4	463.04	359.87	1.29	0.02933
P78560	Death domain-containing protein CRADD OS=Homo sapiens OX=9606 GN=CRADD PE=1 SV=1	95.79	74.65	1.28	0.02935
F8W031	DUF3456 domain-containing protein (Fragment) OS=Homo sapiens OX=9606 PE=1 SV=1	1160.13	915.99	1.27	0.02938
O14907	Tax1-binding protein 3 OS=Homo sapiens OX=9606 GN=TAX1BP3 PE=1 SV=2	89.59	70.08	1.28	0.02945
P02538	Keratin, type II cytoskeletal 6A OS=Homo sapiens OX=9606 GN=KRT6A PE=1 SV=3	4034.84	2131.10	1.89	0.02950
P14317	Hematopoietic lineage cell-specific protein OS=Homo sapiens OX=9606 GN=HCLS1 PE=1 SV=3	93.07	64.47	1.44	0.02969
P60842	Eukaryotic initiation factor 4A-I OS=Homo sapiens OX=9606 GN=EIF4A1 PE=1 SV=1	2488.42	2027.15	1.23	0.03003
P31944	Caspase-14 OS=Homo sapiens OX=9606 GN=CASP14 PE=1 SV=2	76.33	57.56	1.33	0.03062
Q15714	TSC22 domain family protein 1 OS=Homo sapiens OX=9606 GN=TSC22D1 PE=1 SV=3	426.74	307.19	1.39	0.03065
Q15121	Isoform 2 of Astrocytic phosphoprotein PEA-15 OS=Homo sapiens OX=9606 GN=PEA15	1974.43	1233.39	1.60	0.03080
A8MUM1	EARP and GARP complex-interacting protein 1 OS=Homo sapiens OX=9606 GN=EIPR1 PE=1 SV=2	510.04	421.59	1.21	0.03091
Q9BRG1	Vacuolar protein-sorting-associated protein 25 OS=Homo sapiens OX=9606 GN=VPS25 PE=1 SV=1	534.56	440.77	1.21	0.03113

Q9BPY3	Protein FAM118B OS=Homo sapiens OX=9606 GN=FAM118B PE=1 SV=1	145.27	119.26	1.22	0.03134
P46439	Glutathione S-transferase Mu 5 OS=Homo sapiens OX=9606 GN=GSTM5 PE=1 SV=3	560.41	354.95	1.58	0.03136
E9PSI1	Transmembrane 9 superfamily member OS=Homo sapiens OX=9606 PE=3 SV=1	313.85	233.32	1.35	0.03162
Q9H425	Uncharacterized protein C1orf198 OS=Homo sapiens OX=9606 GN=C1orf198 PE=1 SV=1	134.06	105.33	1.27	0.03174
Q8WUX9	Charged multivesicular body protein 7 OS=Homo sapiens OX=9606 GN=CHMP7 PE=1 SV=1	8.28	5.60	1.48	0.03192
P13797	Plastin-3 OS=Homo sapiens OX=9606 GN=PLS3 PE=1 SV=4	1562.55	1183.35	1.32	0.03218
P11215	Isoform 2 of Integrin alpha-M OS=Homo sapiens OX=9606 GN=ITGAM	79.11	62.72	1.26	0.03222
A0A1B0GWK2	Kazrin OS=Homo sapiens OX=9606 GN=KAZN PE=1 SV=1	265.05	210.82	1.26	0.03224
Q969G6	Riboflavin kinase OS=Homo sapiens OX=9606 GN=RFK PE=1 SV=2	277.71	213.14	1.30	0.03226
P00966	Argininosuccinate synthase OS=Homo sapiens OX=9606 GN=ASS1 PE=1 SV=2	1994.42	1544.51	1.29	0.03241
Q8WTS6	Histone-lysine N-methyltransferase SETD7 OS=Homo sapiens OX=9606 GN=SETD7 PE=1 SV=1	195.29	151.71	1.29	0.03250
P98172	Ephrin-B1 OS=Homo sapiens OX=9606 GN=EFNB1 PE=1 SV=1	168.84	139.67	1.21	0.03252
P06396	Gelsolin OS=Homo sapiens OX=9606 GN=GSN PE=1 SV=1	8635.00	7169.94	1.20	0.03259
Q02413	Desmoglein-1 OS=Homo sapiens OX=9606 GN=DSG1 PE=1 SV=2	434.04	298.91	1.45	0.03271
P51570	Isoform 2 of Galactokinase OS=Homo sapiens OX=9606 GN=GALK1	726.70	535.20	1.36	0.03295
P13645	Keratin, type I cytoskeletal 10 OS=Homo sapiens OX=9606 GN=KRT10 PE=1 SV=6	10437.18	6494.34	1.61	0.03298
Q9Y342	Plasmolipin OS=Homo sapiens OX=9606 GN=PLLP PE=1 SV=1	35.61	29.30	1.22	0.03380
Q86TA1	MOB kinase activator 3B OS=Homo sapiens OX=9606 GN=MOB3B PE=1 SV=2	30.99	20.28	1.53	0.03385
O60268	Uncharacterized protein KIAA0513 OS=Homo sapiens OX=9606 GN=KIAA0513 PE=1 SV=1	871.30	720.07	1.21	0.03400
P48539	Calmodulin regulator protein PCP4 OS=Homo sapiens OX=9606 GN=PCP4 PE=1 SV=3	514.38	317.59	1.62	0.03410
P35908	Keratin, type II cytoskeletal 2 epidermal OS=Homo sapiens OX=9606 GN=KRT2 PE=1 SV=2	10383.09	6164.24	1.68	0.03418
Q15126	Phosphomevalonate kinase OS=Homo sapiens OX=9606 GN=PMVK PE=1 SV=3	660.06	481.19	1.37	0.03432
P61204	ADP-ribosylation factor 3 OS=Homo sapiens OX=9606 GN=ARF3 PE=1 SV=2	2786.01	1835.24	1.52	0.03440
Q8TED1	Probable glutathione peroxidase 8 OS=Homo sapiens OX=9606 GN=GPX8 PE=1 SV=2	82.24	62.44	1.32	0.03469
Q14240	Isoform 2 of Eukaryotic initiation factor 4A-II OS=Homo sapiens OX=9606 GN=EIF4A2	1031.39	789.42	1.31	0.03475
J3QLJ5	MIF4G domain-containing protein OS=Homo sapiens OX=9606 GN=MIF4GD PE=1 SV=1	130.29	104.18	1.25	0.03497

Q9BTL3	RNA guanine-N7 methyltransferase activating subunit OS=Homo sapiens OX=9606 GN=RAMAC PE=1 SV=1	224.64	120.09	1.87	0.03497
Q9UHV9	Prefoldin subunit 2 OS=Homo sapiens OX=9606 GN=PFDN2 PE=1 SV=1	1293.85	975.68	1.33	0.03500
Q99447	Isoform 3 of Ethanolamine-phosphate cytidylyltransferase OS=Homo sapiens OX=9606 GN=PCYT2	693.88	528.01	1.31	0.03510
P02533	Keratin, type I cytoskeletal 14 OS=Homo sapiens OX=9606 GN=KRT14 PE=1 SV=4	6367.18	3638.68	1.75	0.03515
P40616	ADP-ribosylation factor-like protein 1 OS=Homo sapiens OX=9606 GN=ARL1 PE=1 SV=1	165.49	114.22	1.45	0.03521
Q9Y5Z4	Heme-binding protein 2 OS=Homo sapiens OX=9606 GN=HEBP2 PE=1 SV=1	1379.74	884.12	1.56	0.03524
Q6ZVX7	F-box only protein 50 OS=Homo sapiens OX=9606 GN=NCCRP1 PE=1 SV=1	59.00	45.25	1.30	0.03524
Q9UBT2	SUMO-activating enzyme subunit 2 OS=Homo sapiens OX=9606 GN=UBA2 PE=1 SV=2	1166.84	902.58	1.29	0.03551
A0AVF1	Intraflagellar transport protein 56 OS=Homo sapiens OX=9606 GN=TTC26 PE=2 SV=1	13.21	8.35	1.58	0.03556
P12277	Creatine kinase B-type OS=Homo sapiens OX=9606 GN=CKB PE=1 SV=1	35126.72	23959.06	1.47	0.03582
P19256	Lymphocyte function-associated antigen 3 OS=Homo sapiens OX=9606 GN=CD58 PE=1 SV=1	70.69	58.24	1.21	0.03586
P13646	Keratin, type I cytoskeletal 13 OS=Homo sapiens OX=9606 GN=KRT13 PE=1 SV=4	253.72	180.84	1.40	0.03613
O95999	B-cell lymphoma/leukemia 10 OS=Homo sapiens OX=9606 GN=BCL10 PE=1 SV=1	23.49	18.33	1.28	0.03646
P35527	Keratin, type I cytoskeletal 9 OS=Homo sapiens OX=9606 GN=KRT9 PE=1 SV=3	9921.25	5593.39	1.77	0.03667
Q5JVS0	Intracellular hyaluronan-binding protein 4 OS=Homo sapiens OX=9606 GN=HABP4 PE=1 SV=1	116.04	90.64	1.28	0.03670
O75506	Heat shock factor-binding protein 1 OS=Homo sapiens OX=9606 GN=HSBP1 PE=1 SV=1	17.43	9.84	1.77	0.03691
P30414	NK-tumor recognition protein OS=Homo sapiens OX=9606 GN=NKTR PE=1 SV=2	20.08	15.05	1.33	0.03692
Q9NV35	Nucleotide triphosphate diphosphatase NUDT15 OS=Homo sapiens OX=9606 GN=NUDT15 PE=1 SV=1	71.20	38.17	1.87	0.03697
P0C870	Bifunctional peptidase and (3S)-lysyl hydroxylase JMJD7 OS=Homo sapiens OX=9606 GN=JMJD7 PE=1 SV=1	186.36	134.88	1.38	0.03718
Q9H3K6	BolA-like protein 2 OS=Homo sapiens OX=9606 GN=BOLA2 PE=1 SV=1	306.06	241.59	1.27	0.03720
Q8NFH8	RalBP1-associated Eps domain-containing protein 2 OS=Homo sapiens OX=9606 GN=REPS2 PE=1 SV=2	89.44	35.85	2.49	0.03721
Q9NP86	Calcium-binding protein 5 OS=Homo sapiens OX=9606 GN=CABP5 PE=1 SV=1	46.90	38.17	1.23	0.03723
Q86YZ3	Hornerin OS=Homo sapiens OX=9606 GN=HRNR PE=1 SV=2	113.61	62.79	1.81	0.03737
Q5D862	Filaggrin-2 OS=Homo sapiens OX=9606 GN=FLG2 PE=1 SV=1	128.65	78.32	1.64	0.03746
O14618	Copper chaperone for superoxide dismutase OS=Homo sapiens OX=9606 GN=CCS PE=1 SV=1	1308.90	962.41	1.36	0.03752

O43657	Tetraspanin-6 OS=Homo sapiens OX=9606 GN=TSPAN6 PE=1 SV=1	99.96	73.20	1.37	0.03809
B0QZ18	Copine-1 OS=Homo sapiens OX=9606 GN=CPNE1 PE=1 SV=1	610.40	477.34	1.28	0.03824
Q9Y259	Choline/ethanolamine kinase OS=Homo sapiens OX=9606 GN=CHKB PE=1 SV=3	176.56	143.65	1.23	0.03848
P15531	Isoform 2 of Nucleoside diphosphate kinase A OS=Homo sapiens OX=9606 GN=NME1	146.42	83.44	1.75	0.03862
B8ZZ50	Eukaryotic translation initiation factor 4E type 2 OS=Homo sapiens OX=9606 GN=EIF4E2 PE=1 SV=1	156.85	112.61	1.39	0.03869
P13929	Isoform 2 of Beta-enolase OS=Homo sapiens OX=9606 GN=ENO3	20.90	17.15	1.22	0.03876
P23443	Ribosomal protein S6 kinase beta-1 OS=Homo sapiens OX=9606 GN=RPS6KB1 PE=1 SV=2	113.58	87.96	1.29	0.03893
P36404	ADP-ribosylation factor-like protein 2 OS=Homo sapiens OX=9606 GN=ARL2 PE=1 SV=4	433.15	339.88	1.27	0.03916
P36639	7,8-dihydro-8-oxoguanine triphosphatase OS=Homo sapiens OX=9606 GN=NUDT1 PE=1 SV=3	58.19	36.59	1.59	0.03938
E9PNP3	Mth938 domain-containing protein OS=Homo sapiens OX=9606 GN=AAMDC PE=1 SV=1	214.37	115.64	1.85	0.03961
Q5VW32	BRO1 domain-containing protein BROX OS=Homo sapiens OX=9606 GN=BROX PE=1 SV=1	640.64	470.31	1.36	0.03991
P23490	Loricrin OS=Homo sapiens OX=9606 GN=LOR PE=1 SV=2	93.17	46.84	1.99	0.03991
P24666	Isoform 2 of Low molecular weight phosphotyrosine protein phosphatase OS=Homo sapiens OX=9606 GN=ACP1	8.23	5.08	1.62	0.03997
Q9Y243	RAC-gamma serine/threonine-protein kinase OS=Homo sapiens OX=9606 GN=AKT3 PE=1 SV=1	123.26	94.52	1.30	0.04008
A0A087WSV8	Nucleobindin 2, isoform CRA_b OS=Homo sapiens OX=9606 GN=NUCB2 PE=1 SV=1	1430.99	1120.89	1.28	0.04018
P29762	Cellular retinoic acid-binding protein 1 OS=Homo sapiens OX=9606 GN=CRABP1 PE=1 SV=2	14415.60	8282.52	1.74	0.04022
P51452	Dual specificity protein phosphatase 3 OS=Homo sapiens OX=9606 GN=DUSP3 PE=1 SV=1	475.50	334.88	1.42	0.04037
P28161	Glutathione S-transferase Mu 2 OS=Homo sapiens OX=9606 GN=GSTM2 PE=1 SV=2	3962.76	2277.58	1.74	0.04060
P12004	Proliferating cell nuclear antigen OS=Homo sapiens OX=9606 GN=PCNA PE=1 SV=1	1124.53	833.21	1.35	0.04061
P47813	Eukaryotic translation initiation factor 1A, X-chromosomal OS=Homo sapiens OX=9606 GN=EIF1AX PE=1 SV=2	1280.23	961.91	1.33	0.04073
P09668	Pro-cathepsin H OS=Homo sapiens OX=9606 GN=CTSH PE=1 SV=4	104.62	82.52	1.27	0.04103
Q5TIG5	Afadin OS=Homo sapiens OX=9606 GN=AFDN PE=1 SV=1	8.50	5.74	1.48	0.04111
Q9NYB0	Telomeric repeat-binding factor 2-interacting protein 1 OS=Homo sapiens OX=9606 GN=TERF2IP PE=1 SV=1	1488.03	1190.90	1.25	0.04114

A0A0B4J2B5	Immunoglobulin heavy variable 3/OR16-9 (non-functional) (Fragment) OS=Homo sapiens OX=9606 GN=IGHV3OR16-9 PE=1 SV=1	168.23	105.74	1.59	0.04116
P35813	Isoform 3 of Protein phosphatase 1A OS=Homo sapiens OX=9606 GN=PPM1A	1577.44	1205.51	1.31	0.04121
P49189	Isoform 3 of 4-trimethylaminobutyraldehyde dehydrogenase OS=Homo sapiens OX=9606 GN=ALDH9A1	24.97	20.39	1.22	0.04135
Q9HAT2	Sialate O-acetylesterase OS=Homo sapiens OX=9606 GN=SIAE PE=1 SV=1	151.30	119.24	1.27	0.04142
P07858	Cathepsin B OS=Homo sapiens OX=9606 GN=CTSB PE=1 SV=3	1220.13	1010.73	1.21	0.04143
P43034	Platelet-activating factor acetylhydrolase IB subunit alpha OS=Homo sapiens OX=9606 GN=PAFAH1B1 PE=1 SV=2	2589.92	2101.44	1.23	0.04143
Q9NT62	Ubiquitin-like-conjugating enzyme ATG3 OS=Homo sapiens OX=9606 GN=ATG3 PE=1 SV=1	412.08	329.13	1.25	0.04156
E9PC47	LIM domain kinase 1 OS=Homo sapiens OX=9606 GN=LIMK1 PE=1 SV=1	38.26	30.56	1.25	0.04167
Q13043	Serine/threonine-protein kinase 4 OS=Homo sapiens OX=9606 GN=STK4 PE=1 SV=2	83.53	68.29	1.22	0.04175
A0A0A0MS69	Phosphodiesterase OS=Homo sapiens OX=9606 GN=PDE1C PE=1 SV=1	105.93	86.04	1.23	0.04178
Q5KU26	Collectin-12 OS=Homo sapiens OX=9606 GN=COLEC12 PE=1 SV=3	802.09	544.68	1.47	0.04204
A0A087X0R6	Sorting nexin-12 OS=Homo sapiens OX=9606 GN=SNX12 PE=1 SV=1	711.04	536.29	1.33	0.04230
P60981	Destrin OS=Homo sapiens OX=9606 GN=DSTN PE=1 SV=3	1974.07	1553.94	1.27	0.04231
P36507	Dual specificity mitogen-activated protein kinase kinase 2 OS=Homo sapiens OX=9606 GN=MAP2K2 PE=1 SV=1	1446.42	1144.63	1.26	0.04235
O95372	Acyl-protein thioesterase 2 OS=Homo sapiens OX=9606 GN=LYPLA2 PE=1 SV=1	649.73	526.50	1.23	0.04250
P49354	Protein farnesyltransferase/geranylgeranyltransferase type-1 subunit alpha OS=Homo sapiens OX=9606 GN=FNTA PE=1 SV=1	141.23	108.31	1.30	0.04259
O15540	Fatty acid-binding protein, brain OS=Homo sapiens OX=9606 GN=FABP7 PE=1 SV=3	5465.08	3199.43	1.71	0.04267
Q00169	Phosphatidylinositol transfer protein alpha isoform OS=Homo sapiens OX=9606 GN=PITPNA PE=1 SV=2	2235.76	1794.18	1.25	0.04282
P54727	UV excision repair protein RAD23 homolog B OS=Homo sapiens OX=9606 GN=RAD23B PE=1 SV=1	2026.91	1628.90	1.24	0.04306
Q9NWY4	Histone PARylation factor 1 OS=Homo sapiens OX=9606 GN=HPF1 PE=1 SV=2	194.51	152.21	1.28	0.04307
A0A087WYT3	Prostaglandin E synthase 3 OS=Homo sapiens OX=9606 GN=PTGES3 PE=1 SV=1	1395.13	931.29	1.50	0.04363
P55285	Cadherin-6 OS=Homo sapiens OX=9606 GN=CDH6 PE=1 SV=1	53.52	38.05	1.41	0.04370
P49441	Inositol polyphosphate 1-phosphatase OS=Homo sapiens OX=9606 GN=INPP1 PE=1 SV=1	429.04	321.00	1.34	0.04405
P20930	Filaggrin OS=Homo sapiens OX=9606 GN=FLG PE=1 SV=3	237.67	167.91	1.42	0.04424
J3KPD3	RNA binding motif protein 7, isoform CRA_c OS=Homo sapiens OX=9606 GN=RBM7 PE=1 SV=1	57.02	39.37	1.45	0.04429

Q96MH2	Protein HEXIM2 OS=Homo sapiens OX=9606 GN=HEXIM2 PE=1 SV=1	47.02	38.79	1.21	0.04438
O95154	Aflatoxin B1 aldehyde reductase member 3 OS=Homo sapiens OX=9606 GN=AKR7A3 PE=1 SV=2	207.44	153.24	1.35	0.04463
P12271	Retinaldehyde-binding protein 1 OS=Homo sapiens OX=9606 GN=RLBP1 PE=1 SV=2	12553.86	8152.54	1.54	0.04464
P29508	Serpin B3 OS=Homo sapiens OX=9606 GN=SERPINB3 PE=1 SV=2	142.21	102.68	1.38	0.04472
P20810	Isoform 6 of Calpastatin OS=Homo sapiens OX=9606 GN=CAST	1405.33	943.52	1.49	0.04493
Q08554	Isoform 1B of Desmocollin-1 OS=Homo sapiens OX=9606 GN=DSC1	197.63	106.71	1.85	0.04497
O75348	V-type proton ATPase subunit G 1 OS=Homo sapiens OX=9606 GN=ATP6V1G1 PE=1 SV=3	1372.85	1032.46	1.33	0.04513
O43396	Thioredoxin-like protein 1 OS=Homo sapiens OX=9606 GN=TXNL1 PE=1 SV=3	1165.52	951.25	1.23	0.04514
P23434	Glycine cleavage system H protein, mitochondrial OS=Homo sapiens OX=9606 GN=GCSH PE=1 SV=2	468.64	370.74	1.26	0.04516
Q6NYC1	Isoform 3 of Bifunctional arginine demethylase and lysyl-hydroxylase JMJD6 OS=Homo sapiens OX=9606 GN=JMJD6	145.67	120.56	1.21	0.04525
Q8NBR6	Ubiquitin carboxyl-terminal hydrolase MINDY-2 OS=Homo sapiens OX=9606 GN=MINDY2 PE=1 SV=2	74.04	61.16	1.21	0.04547
P04271	Protein S100-B OS=Homo sapiens OX=9606 GN=S100B PE=1 SV=2	147.67	114.52	1.29	0.04580
P06753	Isoform 4 of Tropomyosin alpha-3 chain OS=Homo sapiens OX=9606 GN=TPM3	503.78	379.93	1.33	0.04591
O43852	Isoform 3 of Calumenin OS=Homo sapiens OX=9606 GN=CALU	1210.20	910.59	1.33	0.04595
A0A2R8Y5P1	Receptor expression-enhancing protein (Fragment) OS=Homo sapiens OX=9606 GN=REEP1 PE=1 SV=1	137.34	99.00	1.39	0.04603
Q5STX8	Allograft inflammatory factor 1 (Fragment) OS=Homo sapiens OX=9606 GN=AIF1 PE=1 SV=1	138.67	103.75	1.34	0.04615
Q96EQ0	Small glutamine-rich tetratricopeptide repeat-containing protein beta OS=Homo sapiens OX=9606 GN=SGTB PE=1 SV=1	263.09	201.41	1.31	0.04647
P35270	Sepiapterin reductase OS=Homo sapiens OX=9606 GN=SPR PE=1 SV=1	709.60	540.19	1.31	0.04672
O60437	Periplakin OS=Homo sapiens OX=9606 GN=PPL PE=1 SV=4	965.19	753.12	1.28	0.04682
P21266	Glutathione S-transferase Mu 3 OS=Homo sapiens OX=9606 GN=GSTM3 PE=1 SV=3	4161.69	2492.01	1.67	0.04685
Q9HAF1	Isoform 3 of Chromatin modification-related protein MEAF6 OS=Homo sapiens OX=9606 GN=MEAF6	164.49	122.30	1.34	0.04688
P55327	Isoform 4 of Tumor protein D52 OS=Homo sapiens OX=9606 GN=TPD52	852.68	562.49	1.52	0.04733
Q9BZ67	FERM domain-containing protein 8 OS=Homo sapiens OX=9606 GN=FRMD8 PE=1 SV=1	57.05	46.56	1.23	0.04764
P47224	Guanine nucleotide exchange factor MSS4 OS=Homo sapiens OX=9606 GN=RABIF PE=1 SV=2	91.18	71.52	1.27	0.04791
P09467	Fructose-1,6-bisphosphatase 1 OS=Homo sapiens OX=9606 GN=FBP1 PE=1 SV=5	220.21	168.40	1.31	0.04794

P30047	GTP cyclohydrolase 1 feedback regulatory protein OS=Homo sapiens OX=9606 GN=GCHFR PE=1 SV=3	272.18	208.34	1.31	0.04839
P27482	Calmodulin-like protein 3 OS=Homo sapiens OX=9606 GN=CALML3 PE=1 SV=2	150.30	116.71	1.29	0.04846
Q06323	Proteasome activator complex subunit 1 OS=Homo sapiens OX=9606 GN=PSME1 PE=1 SV=1	2321.33	1673.46	1.39	0.04868
Q99497	Protein/nucleic acid deglycase DJ-1 OS=Homo sapiens OX=9606 GN=PARK7 PE=1 SV=2	10619.07	6552.39	1.62	0.04878
Q7Z4H3	HD domain-containing protein 2 OS=Homo sapiens OX=9606 GN=HDDC2 PE=1 SV=1	377.34	265.61	1.42	0.04890
Q8WVY7	Ubiquitin-like domain-containing CTD phosphatase 1 OS=Homo sapiens OX=9606 GN=UBLCP1 PE=1 SV=2	864.37	640.17	1.35	0.04901
Q86V88	Magnesium-dependent phosphatase 1 OS=Homo sapiens OX=9606 GN=MDP1 PE=1 SV=1	99.17	77.10	1.29	0.04918
Q9HB40	Retinoid-inducible serine carboxypeptidase OS=Homo sapiens OX=9606 GN=SCPEP1 PE=1 SV=1	62.53	39.79	1.57	0.04926
Q96IZ7	Serine/Arginine-related protein 53 OS=Homo sapiens OX=9606 GN=RSRC1 PE=1 SV=1	30.55	24.64	1.24	0.04982
P41219	Isoform 2 of Peripherin OS=Homo sapiens OX=9606 GN=PRPH	1227.43	731.78	1.68	0.04994

AD, Alzheimer's disease; FC, fold-change (FC>1.2 included); NC, cognitively normal controls; $P<0.05$

Supplementary Table 5. Extended mass spectrometry data on significantly down-regulated proteins in the retina of Alzheimer's patients compared to cognitively normal controls.

**Accession**	**Description**	**AD (Means)**	**NC (Means)**	**FC (*-1)**	***P***
Q7Z5J4	Retinoic acid-induced protein 1 OS=Homo sapiens OX=9606 GN=RAI1 PE=1 SV=2	137.76	191.14	1.39	2.211E-05
Q9NPE3	H/ACA ribonucleoprotein complex subunit 3 OS=Homo sapiens OX=9606 GN=NOP10 PE=1 SV=1	45.27	59.72	1.32	2.673E-05
Q5JTH9	RRP12-like protein OS=Homo sapiens OX=9606 GN=RRP12 PE=1 SV=2	346.19	524.04	1.51	4.516E-05
P49207	60S ribosomal protein L34 OS=Homo sapiens OX=9606 GN=RPL34 PE=1 SV=3	1135.67	1669.64	1.47	0.00013
Q9H553	Alpha-1,3/1,6-mannosyltransferase ALG2 OS=Homo sapiens OX=9606 GN=ALG2 PE=1 SV=1	593.09	755.64	1.27	0.00018
P35268	60S ribosomal protein L22 OS=Homo sapiens OX=9606 GN=RPL22 PE=1 SV=2	429.55	613.96	1.43	0.00019
P42766	60S ribosomal protein L35 OS=Homo sapiens OX=9606 GN=RPL35 PE=1 SV=2	1558.92	2123.74	1.36	0.00022
Q96KM6	Zinc finger protein 512B OS=Homo sapiens OX=9606 GN=ZNF512B PE=1 SV=1	336.70	441.77	1.31	0.00029
P04000	Long-wave-sensitive opsin 1 OS=Homo sapiens OX=9606 GN=OPN1LW PE=1 SV=1	821.14	1552.94	1.89	0.00035
Q96PU4	E3 ubiquitin-protein ligase UHRF2 OS=Homo sapiens OX=9606 GN=UHRF2 PE=1 SV=1	251.73	337.09	1.34	0.00038
Q08828	Adenylate cyclase type 1 OS=Homo sapiens OX=9606 GN=ADCY1 PE=1 SV=2	394.82	582.32	1.47	0.00038
Q8IWI9	MAX gene-associated protein OS=Homo sapiens OX=9606 GN=MGA PE=1 SV=4	15.10	31.80	2.11	0.00046
Q8N4S7	Progestin and adipoQ receptor family member 4 OS=Homo sapiens OX=9606 GN=PAQR4 PE=2 SV=3	213.99	279.32	1.31	0.00047
A0A1B0GUQ4	FERM and PDZ domain-containing protein 2 OS=Homo sapiens OX=9606 GN=FRMPD2 PE=1 SV=1	242.91	351.85	1.45	0.00048
P19087	Guanine nucleotide-binding protein G(t) subunit alpha-2 OS=Homo sapiens OX=9606 GN=GNAT2 PE=2 SV=4	1071.16	1600.60	1.49	0.00052
A0A087WUW9	ADP-ribosylation factor-like protein 15 OS=Homo sapiens OX=9606 GN=ARL15 PE=1 SV=1	30.52	41.99	1.38	0.00052
M0QXA7	Protein Wiz OS=Homo sapiens OX=9606 GN=WIZ PE=1 SV=1	160.17	216.70	1.35	0.00056
Q9BZE1	39S ribosomal protein L37, mitochondrial OS=Homo sapiens OX=9606 GN=MRPL37 PE=1 SV=2	287.75	387.20	1.35	0.00064
Q9BV73	Centrosome-associated protein CEP250 OS=Homo sapiens OX=9606 GN=CEP250 PE=1 SV=2	227.57	326.12	1.43	0.00065
P61353	60S ribosomal protein L27 OS=Homo sapiens OX=9606 GN=RPL27 PE=1 SV=2	1376.63	1992.62	1.45	0.00069
Q15233	Non-POU domain-containing octamer-binding protein OS=Homo sapiens OX=9606 GN=NONO PE=1 SV=4	7052.67	9062.23	1.28	0.00069
H0YMV8	40S ribosomal protein S27 OS=Homo sapiens OX=9606 GN=RPS27L PE=1 SV=1	64.34	96.38	1.50	0.00078
Q96QC0	Serine/threonine-protein phosphatase 1 regulatory subunit 10 OS=Homo sapiens OX=9606 GN=PPP1R10 PE=1 SV=1	310.60	405.29	1.30	0.00083

O75916	Isoform 3 of Regulator of G-protein signaling 9 OS=Homo sapiens OX=9606 GN=RGS9	1385.37	3024.08	2.18	0.00086
A0A0A0MS41	Sidoreflexin OS=Homo sapiens OX=9606 GN=SFXN3 PE=1 SV=1	2188.85	3028.99	1.38	0.00086
A0A0C4DH72	Immunoglobulin kappa variable 1-6 OS=Homo sapiens OX=9606 GN=IGKV1-6 PE=3 SV=1	17.62	34.62	1.97	0.00088
Q9BZV3	Interphotoreceptor matrix proteoglycan 2 OS=Homo sapiens OX=9606 GN=IMPG2 PE=1 SV=3	3876.18	9847.49	2.54	0.00092
Q14028	Isoform GARP2 of Cyclic nucleotide-gated cation channel beta-1 OS=Homo sapiens OX=9606 GN=CNGB1	115.79	212.66	1.84	0.00096
Q13523	Serine/threonine-protein kinase PRP4 homolog OS=Homo sapiens OX=9606 GN=PRPF4B PE=1 SV=3	1300.35	1696.36	1.30	0.00100
Q14721	Potassium voltage-gated channel subfamily B member 1 OS=Homo sapiens OX=9606 GN=KCNB1 PE=1 SV=2	568.65	763.06	1.34	0.00103
P39210	Protein Mpv17 OS=Homo sapiens OX=9606 GN=MPV17 PE=1 SV=1	70.41	91.18	1.29	0.00103
Q96JM3	Chromosome alignment-maintaining phosphoprotein 1 OS=Homo sapiens OX=9606 GN=CHAMP1 PE=1 SV=2	885.42	1210.08	1.37	0.00107
O60721	Isoform 2 of Sodium/potassium/calcium exchanger 1 OS=Homo sapiens OX=9606 GN=SLC24A1	278.87	658.72	2.36	0.00109
Q86TX2	Acyl-coenzyme A thioesterase 1 OS=Homo sapiens OX=9606 GN=ACOT1 PE=1 SV=1	1406.26	1781.28	1.27	0.00117
O60725	Protein-S-isoprenylcysteine O-methyltransferase OS=Homo sapiens OX=9606 GN=ICMT PE=1 SV=1	56.43	90.29	1.60	0.00119
P78363	Retinal-specific phospholipid-transporting ATPase ABCA4 OS=Homo sapiens OX=9606 GN=ABCA4 PE=1 SV=3	4963.73	9186.12	1.85	0.00119
O14775	Guanine nucleotide-binding protein subunit beta-5 OS=Homo sapiens OX=9606 GN=GNB5 PE=1 SV=2	1144.84	1751.26	1.53	0.00119
D3DWX8	Family with sequence similarity 3, member A, isoform CRA_c OS=Homo sapiens OX=9606 GN=FAM3A PE=1 SV=1	40.98	62.95	1.54	0.00123
P61247	40S ribosomal protein S3a OS=Homo sapiens OX=9606 GN=RPS3A PE=1 SV=2	3383.30	4305.64	1.27	0.00124
Q92759	General transcription factor IIH subunit 4 OS=Homo sapiens OX=9606 GN=GTF2H4 PE=1 SV=1	42.86	57.46	1.34	0.00124
Q6ZXV5	Protein O-mannosyl-transferase TMTC3 OS=Homo sapiens OX=9606 GN=TMTC3 PE=1 SV=2	411.20	528.65	1.29	0.00126
Q15067	Peroxisomal acyl-coenzyme A oxidase 1 OS=Homo sapiens OX=9606 GN=ACOX1 PE=1 SV=3	745.13	953.53	1.28	0.00128
Q14318	Isoform 2 of Peptidyl-prolyl cis-trans isomerase FKBP8 OS=Homo sapiens OX=9606 GN=FKBP8	1404.77	1803.87	1.28	0.00131
Q8NCY6	Myb/SANT-like DNA-binding domain-containing protein 4 OS=Homo sapiens OX=9606 GN=MSANTD4 PE=1 SV=1	54.76	70.46	1.29	0.00135
P51531	Probable global transcription activator SNF2L2 OS=Homo sapiens OX=9606 GN=SMARCA2 PE=1 SV=2	1653.49	2089.63	1.26	0.00136
Q9H4B0	Probable tRNA N6-adenosine threonylcarbamoyltransferase, mitochondrial OS=Homo sapiens OX=9606 GN=OSGEPL1 PE=2 SV=2	115.66	160.62	1.39	0.00136

Q5T1C6	Acyl-coenzyme A thioesterase THEM4 OS=Homo sapiens OX=9606 GN=THEM4 PE=1 SV=1	267.13	385.44	1.44	0.00136
P61764	Syntaxin-binding protein 1 OS=Homo sapiens OX=9606 GN=STXBP1 PE=1 SV=1	1209.71	1670.29	1.38	0.00141
Q5T3J3	Ligand-dependent nuclear receptor-interacting factor 1 OS=Homo sapiens OX=9606 GN=LRIF1 PE=1 SV=1	239.56	304.88	1.27	0.00143
Q9Y5B9	FACT complex subunit SPT16 OS=Homo sapiens OX=9606 GN=SUPT16H PE=1 SV=1	1721.89	2382.39	1.38	0.00144
Q9UGL1	Isoform 2 of Lysine-specific demethylase 5B OS=Homo sapiens OX=9606 GN=KDM5B	454.20	707.33	1.56	0.00146
Q96AG4	Leucine-rich repeat-containing protein 59 OS=Homo sapiens OX=9606 GN=LRRC59 PE=1 SV=1	1202.76	1693.98	1.41	0.00148
F5GX28	Lysine-specific demethylase 4B OS=Homo sapiens OX=9606 GN=KDM4B PE=1 SV=2	243.38	360.78	1.48	0.00151
Q6NUK1	Calcium-binding mitochondrial carrier protein SCaMC-1 OS=Homo sapiens OX=9606 GN=SLC25A24 PE=1 SV=2	3060.37	5023.55	1.64	0.00152
Q02880	Isoform Beta-1 of DNA topoisomerase 2-beta OS=Homo sapiens OX=9606 GN=TOP2B	6484.20	9188.47	1.42	0.00160
P63211	Guanine nucleotide-binding protein G(T) subunit gamma-T1 OS=Homo sapiens OX=9606 GN=GNGT1 PE=1 SV=2	1963.86	3028.85	1.54	0.00163
P29973	Isoform 2 of cGMP-gated cation channel alpha-1 OS=Homo sapiens OX=9606 GN=CNGA1	792.46	1841.01	2.32	0.00164
K7EKE6	Lon protease homolog, mitochondrial OS=Homo sapiens OX=9606 GN=LONP1 PE=1 SV=1	2977.31	4024.30	1.35	0.00164
Q13562	Neurogenic differentiation factor 1 OS=Homo sapiens OX=9606 GN=NEUROD1 PE=1 SV=3	19.95	27.43	1.38	0.00165
Q92831	Histone acetyltransferase KAT2B OS=Homo sapiens OX=9606 GN=KAT2B PE=1 SV=3	132.01	190.27	1.44	0.00166
Q08945	FACT complex subunit SSRP1 OS=Homo sapiens OX=9606 GN=SSRP1 PE=1 SV=1	1160.96	1471.93	1.27	0.00170
Q17R60	Interphotoreceptor matrix proteoglycan 1 OS=Homo sapiens OX=9606 GN=IMPG1 PE=1 SV=2	4962.91	11887.91	2.40	0.00171
P19367	Hexokinase-1 OS=Homo sapiens OX=9606 GN=HK1 PE=1 SV=3	159.78	258.75	1.62	0.00174
O94806	Serine/threonine-protein kinase D3 OS=Homo sapiens OX=9606 GN=PRKD3 PE=1 SV=1	25.74	34.32	1.33	0.00179
Q9BSJ5	Uncharacterized protein C17orf80 OS=Homo sapiens OX=9606 GN=C17orf80 PE=2 SV=2	278.25	364.65	1.31	0.00180
Q03701	CCAAT/enhancer-binding protein zeta OS=Homo sapiens OX=9606 GN=CEBPZ PE=1 SV=3	56.18	75.44	1.34	0.00187
Q5SSJ5	Heterochromatin protein 1-binding protein 3 OS=Homo sapiens OX=9606 GN=HP1BP3 PE=1 SV=1	7121.93	10412.01	1.46	0.00187
Q8TCG5	Carnitine O-palmitoyltransferase 1, brain isoform OS=Homo sapiens OX=9606 GN=CPT1C PE=1 SV=1	378.60	488.72	1.29	0.00189
P22087	rRNA 2'-O-methyltransferase fibrillarin OS=Homo sapiens OX=9606 GN=FBL PE=1 SV=2	762.38	1061.22	1.39	0.00189
P83731	60S ribosomal protein L24 OS=Homo sapiens OX=9606 GN=RPL24 PE=1 SV=1	1601.02	2353.30	1.47	0.00195
P35914	Hydroxymethylglutaryl-CoA lyase, mitochondrial OS=Homo sapiens OX=9606 GN=HMGCL PE=1 SV=2	741.00	939.73	1.27	0.00195

I3L4I4	TOM1-like protein 1 OS=Homo sapiens OX=9606 GN=TOM1L1 PE=1 SV=1	156.18	260.00	1.66	0.00198
Q9NVH0	Exonuclease 3'-5' domain-containing protein 2 OS=Homo sapiens OX=9606 GN=EXD2 PE=1 SV=2	216.29	270.72	1.25	0.00201
Q12923	Isoform 4 of Tyrosine-protein phosphatase non-receptor type 13 OS=Homo sapiens OX=9606 GN=PTPN13	969.04	1487.03	1.53	0.00202
O94874	E3 UFM1-protein ligase 1 OS=Homo sapiens OX=9606 GN=UFL1 PE=1 SV=2	2551.36	3285.43	1.29	0.00205
Q4R9M9	Kinesin family member 1Bbeta isoform II OS=Homo sapiens OX=9606 GN=KIF1B PE=1 SV=1	763.96	982.72	1.29	0.00207
A6NHR9	Structural maintenance of chromosomes flexible hinge domain-containing protein 1 OS=Homo sapiens OX=9606 GN=SMCHD1 PE=1 SV=2	1687.32	2233.41	1.32	0.00207
Q8TB37	Iron-sulfur protein NUBPL OS=Homo sapiens OX=9606 GN=NUBPL PE=1 SV=3	115.06	147.24	1.28	0.00208
Q7L592	Protein arginine methyltransferase NDUFAF7, mitochondrial OS=Homo sapiens OX=9606 GN=NDUFAF7 PE=1 SV=1	355.20	461.61	1.30	0.00210
Q9BUJ2	Heterogeneous nuclear ribonucleoprotein U-like protein 1 OS=Homo sapiens OX=9606 GN=HNRNPUL1 PE=1 SV=2	2473.33	3273.79	1.32	0.00215
Q9H2P0	Activity-dependent neuroprotector homeobox protein OS=Homo sapiens OX=9606 GN=ADNP PE=1 SV=1	1163.70	1657.90	1.42	0.00220
Q9UIG0	Tyrosine-protein kinase BAZ1B OS=Homo sapiens OX=9606 GN=BAZ1B PE=1 SV=2	1923.85	2682.48	1.39	0.00222
P17480	Nucleolar transcription factor 1 OS=Homo sapiens OX=9606 GN=UBTF PE=1 SV=1	1107.77	1431.42	1.29	0.00225
O60264	SWI/SNF-related matrix-associated actin-dependent regulator of chromatin subfamily A member 5 OS=Homo sapiens OX=9606 GN=SMARCA5 PE=1 SV=1	3371.65	4562.95	1.35	0.00225
P38432	Coilin OS=Homo sapiens OX=9606 GN=COIL PE=1 SV=1	88.63	132.66	1.50	0.00228
Q6UB98	Ankyrin repeat domain-containing protein 12 OS=Homo sapiens OX=9606 GN=ANKRD12 PE=1 SV=3	33.32	43.13	1.29	0.00237
Q8IXT5	RNA-binding protein 12B OS=Homo sapiens OX=9606 GN=RBM12B PE=1 SV=2	652.16	901.76	1.38	0.00238
Q9HCE3	Zinc finger protein 532 OS=Homo sapiens OX=9606 GN=ZNF532 PE=1 SV=2	32.92	42.14	1.28	0.00241
Q96CQ1	Solute carrier family 25 member 36 OS=Homo sapiens OX=9606 GN=SLC25A36 PE=1 SV=1	78.74	103.28	1.31	0.00242
Q15717	ELAV-like protein 1 OS=Homo sapiens OX=9606 GN=ELAVL1 PE=1 SV=2	2237.03	2854.27	1.28	0.00249
Q9NTN3	UDP-glucuronic acid/UDP-N-acetylgalactosamine transporter OS=Homo sapiens OX=9606 GN=SLC35D1 PE=2 SV=1	510.77	696.77	1.36	0.00253
Q49A26	Putative oxidoreductase GLYR1 OS=Homo sapiens OX=9606 GN=GLYR1 PE=1 SV=3	800.43	1027.47	1.28	0.00254
Q96I24	Far upstream element-binding protein 3 OS=Homo sapiens OX=9606 GN=FUBP3 PE=1 SV=2	1206.80	1519.38	1.26	0.00260
Q8NCC3	Group XV phospholipase A2 OS=Homo sapiens OX=9606 GN=PLA2G15 PE=1 SV=2	55.19	88.42	1.60	0.00263
Q9NW64	Pre-mRNA-splicing factor RBM22 OS=Homo sapiens OX=9606 GN=RBM22 PE=1 SV=1	852.61	1098.69	1.29	0.00263
Q96SK2	Isoform 2 of Transmembrane protein 209 OS=Homo sapiens OX=9606 GN=TMEM209	280.86	371.59	1.32	0.00265

Q6KCM7	Isoform 5 of Calcium-binding mitochondrial carrier protein SCaMC-2 OS=Homo sapiens OX=9606 GN=SLC25A25	1590.61	2373.72	1.49	0.00268
P55265	Isoform 4 of Double-stranded RNA-specific adenosine deaminase OS=Homo sapiens OX=9606 GN=ADAR	3695.65	4739.66	1.28	0.00272
Q02846	Retinal guanylyl cyclase 1 OS=Homo sapiens OX=9606 GN=GUCY2D PE=1 SV=2	1519.22	2334.86	1.54	0.00277
P23942	Peripherin-2 OS=Homo sapiens OX=9606 GN=PRPH2 PE=1 SV=1	1926.55	5266.71	2.73	0.00279
Q9P1Z0	Zinc finger and BTB domain-containing protein 4 OS=Homo sapiens OX=9606 GN=ZBTB4 PE=1 SV=3	99.21	130.59	1.32	0.00280
Q14690	Protein RRP5 homolog OS=Homo sapiens OX=9606 GN=PDCD11 PE=1 SV=3	129.96	183.72	1.41	0.00280
O75367	Core histone macro-H2A.1 OS=Homo sapiens OX=9606 GN=H2AFY PE=1 SV=4	9523.66	14529.77	1.53	0.00282
Q9NPI0	Transmembrane protein 138 OS=Homo sapiens OX=9606 GN=TMEM138 PE=1 SV=1	40.63	54.75	1.35	0.00285
Q14839	Isoform 2 of Chromodomain-helicase-DNA-binding protein 4 OS=Homo sapiens OX=9606 GN=CHD4	3338.60	4536.76	1.36	0.00290
L0R6Q1	SLC35A4 upstream open reading frame protein OS=Homo sapiens OX=9606 GN=SLC35A4 PE=3 SV=1	494.59	641.62	1.30	0.00291
Q7Z3K3	Pogo transposable element with ZNF domain OS=Homo sapiens OX=9606 GN=POGZ PE=1 SV=2	670.13	880.37	1.31	0.00294
P08100	Rhodopsin OS=Homo sapiens OX=9606 GN=RHO PE=1 SV=1	4543.76	11529.48	2.54	0.00307
P03999	Short-wave-sensitive opsin 1 OS=Homo sapiens OX=9606 GN=OPN1SW PE=1 SV=1	214.41	336.65	1.57	0.00316
P40429	60S ribosomal protein L13a OS=Homo sapiens OX=9606 GN=RPL13A PE=1 SV=2	1913.84	2466.83	1.29	0.00320
Q9Y426	C2 domain-containing protein 2 OS=Homo sapiens OX=9606 GN=C2CD2 PE=1 SV=2	812.55	1051.76	1.29	0.00320
P62913	60S ribosomal protein L11 OS=Homo sapiens OX=9606 GN=RPL11 PE=1 SV=2	1122.02	1642.98	1.46	0.00322
Q9BT22	Chitobiosyldiphosphodolichol beta-mannosyltransferase OS=Homo sapiens OX=9606 GN=ALG1 PE=1 SV=2	387.48	496.90	1.28	0.00331
Q9NVJ2	ADP-ribosylation factor-like protein 8B OS=Homo sapiens OX=9606 GN=ARL8B PE=1 SV=1	870.22	1105.28	1.27	0.00334
Q8NDX5	Isoform 7 of Polyhomeotic-like protein 3 OS=Homo sapiens OX=9606 GN=PHC3	171.23	233.13	1.36	0.00337
Q53GS7	Nucleoporin GLE1 OS=Homo sapiens OX=9606 GN=GLE1 PE=1 SV=2	51.66	71.76	1.39	0.00337
Q15118	[Pyruvate dehydrogenase (acetyl-transferring)] kinase isozyme 1, mitochondrial OS=Homo sapiens OX=9606 GN=PDK1 PE=1 SV=1	715.14	924.65	1.29	0.00358
Q56VL3	OCIA domain-containing protein 2 OS=Homo sapiens OX=9606 GN=OCIAD2 PE=1 SV=1	366.92	543.96	1.48	0.00360
Q9GZT6	Coiled-coil domain-containing protein 90B, mitochondrial OS=Homo sapiens OX=9606 GN=CCDC90B PE=1 SV=2	624.64	814.13	1.30	0.00363
Q6N043	Zinc finger protein 280D OS=Homo sapiens OX=9606 GN=ZNF280D PE=1 SV=3	120.61	155.48	1.29	0.00364
O43264	Centromere/kinetochore protein zw10 homolog OS=Homo sapiens OX=9606 GN=ZW10 PE=1 SV=3	471.75	602.10	1.28	0.00366

P62277	40S ribosomal protein S13 OS=Homo sapiens OX=9606 GN=RPS13 PE=1 SV=2	1696.70	2212.39	1.30	0.00367
O15047	Histone-lysine N-methyltransferase SETD1A OS=Homo sapiens OX=9606 GN=SETD1A PE=1 SV=3	46.59	64.30	1.38	0.00373
Q96ME7	Zinc finger protein 512 OS=Homo sapiens OX=9606 GN=ZNF512 PE=1 SV=2	1137.00	1553.94	1.37	0.00380
P36578	60S ribosomal protein L4 OS=Homo sapiens OX=9606 GN=RPL4 PE=1 SV=5	3224.08	4413.76	1.37	0.00380
Q8WWQ0	PH-interacting protein OS=Homo sapiens OX=9606 GN=PHIP PE=1 SV=2	1291.81	1646.77	1.27	0.00382
O14832	Phytanoyl-CoA dioxygenase, peroxisomal OS=Homo sapiens OX=9606 GN=PHYH PE=1 SV=1	160.07	213.78	1.34	0.00384
P46100	Transcriptional regulator ATRX OS=Homo sapiens OX=9606 GN=ATRX PE=1 SV=5	1007.01	1349.78	1.34	0.00388
F8VX10	KAT8 regulatory NSL complex subunit 2 OS=Homo sapiens OX=9606 GN=KANSL2 PE=1 SV=1	53.23	68.44	1.29	0.00389
Q9UPP1	Histone lysine demethylase PHF8 OS=Homo sapiens OX=9606 GN=PHF8 PE=1 SV=3	121.42	194.70	1.60	0.00393
O95696	Isoform 2 of Bromodomain-containing protein 1 OS=Homo sapiens OX=9606 GN=BRD1	194.32	250.07	1.29	0.00393
P54819	Adenylate kinase 2, mitochondrial OS=Homo sapiens OX=9606 GN=AK2 PE=1 SV=2	2347.29	3595.15	1.53	0.00398
P56715	Oxygen-regulated protein 1 OS=Homo sapiens OX=9606 GN=RP1 PE=1 SV=1	459.79	694.24	1.51	0.00401
P52272	Heterogeneous nuclear ribonucleoprotein M OS=Homo sapiens OX=9606 GN=HNRNPM PE=1 SV=3	13023.51	17928.54	1.38	0.00408
P61313	60S ribosomal protein L15 OS=Homo sapiens OX=9606 GN=RPL15 PE=1 SV=2	890.01	1127.90	1.27	0.00427
Q9UH99	Isoform 2 of SUN domain-containing protein 2 OS=Homo sapiens OX=9606 GN=SUN2	564.44	736.67	1.31	0.00440
A0A087WXF8	Nucleolar protein of 40 kDa OS=Homo sapiens OX=9606 GN=ZCCHC17 PE=1 SV=1	68.44	87.62	1.28	0.00446
P56182	Ribosomal RNA processing protein 1 homolog A OS=Homo sapiens OX=9606 GN=RRP1 PE=1 SV=1	80.54	108.00	1.34	0.00448
Q2TB10	Zinc finger protein 800 OS=Homo sapiens OX=9606 GN=ZNF800 PE=1 SV=1	147.53	192.00	1.30	0.00455
A0A087X117	Nodal modulator 1 OS=Homo sapiens OX=9606 GN=NOMO1 PE=1 SV=1	64.14	96.00	1.50	0.00473
Q1KMD3	Heterogeneous nuclear ribonucleoprotein U-like protein 2 OS=Homo sapiens OX=9606 GN=HNRNPUL2 PE=1 SV=1	4147.45	5206.04	1.26	0.00478
P61026	Ras-related protein Rab-10 OS=Homo sapiens OX=9606 GN=RAB10 PE=1 SV=1	1555.61	2012.13	1.29	0.00480
Q14683	Structural maintenance of chromosomes protein 1A OS=Homo sapiens OX=9606 GN=SMC1A PE=1 SV=2	4639.03	5827.76	1.26	0.00487
P27635	60S ribosomal protein L10 OS=Homo sapiens OX=9606 GN=RPL10 PE=1 SV=4	1193.92	1574.73	1.32	0.00491
P62424	60S ribosomal protein L7a OS=Homo sapiens OX=9606 GN=RPL7A PE=1 SV=2	2268.97	2942.17	1.30	0.00496
K7ELF7	Retinol dehydrogenase 8 OS=Homo sapiens OX=9606 GN=RDH8 PE=1 SV=1	256.17	439.26	1.71	0.00500
Q9UIS9	Isoform 9 of Methyl-CpG-binding domain protein 1 OS=Homo sapiens OX=9606 GN=MBD1	508.26	672.28	1.32	0.00503
Q03395	Rod outer segment membrane protein 1 OS=Homo sapiens OX=9606 GN=ROM1 PE=1 SV=2	1005.87	1942.72	1.93	0.00505

Q9H936	Mitochondrial glutamate carrier 1 OS=Homo sapiens OX=9606 GN=SLC25A22 PE=1 SV=1	2796.50	3992.80	1.43	0.00506
P35610	Sterol O-acyltransferase 1 OS=Homo sapiens OX=9606 GN=SOAT1 PE=1 SV=3	25.93	33.75	1.30	0.00507
Q9NXE4	Isoform 4 of Sphingomyelin phosphodiesterase 4 OS=Homo sapiens OX=9606 GN=SMPD4	279.95	363.42	1.30	0.00508
Q9HCC8	Isoform 3 of Glycerophosphoinositol inositolphosphodiesterase GDPD2 OS=Homo sapiens OX=9606 GN=GDPD2	153.18	199.04	1.30	0.00515
Q9Y6A9	Signal peptidase complex subunit 1 OS=Homo sapiens OX=9606 GN=SPCS1 PE=1 SV=4	243.63	343.80	1.41	0.00519
A3KMH1	von Willebrand factor A domain-containing protein 8 OS=Homo sapiens OX=9606 GN=VWA8 PE=1 SV=2	957.82	1287.97	1.34	0.00522
Q9BQ39	ATP-dependent RNA helicase DDX50 OS=Homo sapiens OX=9606 GN=DDX50 PE=1 SV=1	528.68	718.44	1.36	0.00524
Q9HBH5	Retinol dehydrogenase 14 OS=Homo sapiens OX=9606 GN=RDH14 PE=1 SV=1	353.01	481.91	1.37	0.00525
Q9UBW7	Zinc finger MYM-type protein 2 OS=Homo sapiens OX=9606 GN=ZMYM2 PE=1 SV=1	306.51	406.62	1.33	0.00526
Q96JM2	Isoform 3 of Zinc finger protein 462 OS=Homo sapiens OX=9606 GN=ZNF462	168.94	249.91	1.48	0.00529
Q8IZD4	mRNA-decapping enzyme 1B OS=Homo sapiens OX=9606 GN=DCP1B PE=1 SV=2	16.36	21.55	1.32	0.00541
Q6ZS82	Regulator of G-protein signaling 9-binding protein OS=Homo sapiens OX=9606 GN=RGS9BP PE=2 SV=1	180.83	331.88	1.84	0.00544
O95159	Zinc finger protein-like 1 OS=Homo sapiens OX=9606 GN=ZFPL1 PE=1 SV=2	101.53	146.00	1.44	0.00544
P21397	Amine oxidase [flavin-containing] A OS=Homo sapiens OX=9606 GN=MAOA PE=1 SV=1	2747.05	3780.61	1.38	0.00549
Q9UG56	Phosphatidylserine decarboxylase proenzyme, mitochondrial OS=Homo sapiens OX=9606 GN=PISD PE=2 SV=4	145.74	192.42	1.32	0.00563
A0A0A0MSV9	Tapasin OS=Homo sapiens OX=9606 GN=TAPBP PE=1 SV=1	292.01	375.63	1.29	0.00565
J3QLS3	28S ribosomal protein S7, mitochondrial OS=Homo sapiens OX=9606 GN=MRPS7 PE=1 SV=1	389.96	523.36	1.34	0.00566
O94826	Mitochondrial import receptor subunit TOM70 OS=Homo sapiens OX=9606 GN=TOMM70 PE=1 SV=1	5428.78	7579.92	1.40	0.00567
Q96NR8	Retinol dehydrogenase 12 OS=Homo sapiens OX=9606 GN=RDH12 PE=1 SV=3	1829.75	3089.46	1.69	0.00567
Q00839	Isoform 2 of Heterogeneous nuclear ribonucleoprotein U OS=Homo sapiens OX=9606 GN=HNRNPU	3442.14	4906.01	1.43	0.00569
Q96NB2	Sideroflexin-2 OS=Homo sapiens OX=9606 GN=SFXN2 PE=1 SV=2	46.59	75.61	1.62	0.00572
Q6YN16	Hydroxysteroid dehydrogenase-like protein 2 OS=Homo sapiens OX=9606 GN=HSDL2 PE=1 SV=1	1468.35	1968.12	1.34	0.00575
Q8IXI1	Mitochondrial Rho GTPase 2 OS=Homo sapiens OX=9606 GN=RHOT2 PE=1 SV=2	834.56	1215.25	1.46	0.00577
Q9UIW0	Ventral anterior homeobox 2 OS=Homo sapiens OX=9606 GN=VAX2 PE=1 SV=1	35.11	51.94	1.48	0.00590
A0A0A0MRM8	Unconventional myosin-VI OS=Homo sapiens OX=9606 GN=MYO6 PE=1 SV=1	1038.10	1382.20	1.33	0.00597

Q9HC21	Mitochondrial thiamine pyrophosphate carrier OS=Homo sapiens OX=9606 GN=SLC25A19 PE=1 SV=1	58.27	75.05	1.29	0.00600
Q15326	Zinc finger MYND domain-containing protein 11 OS=Homo sapiens OX=9606 GN=ZMYND11 PE=1 SV=2	205.80	269.15	1.31	0.00606
P42677	40S ribosomal protein S27 OS=Homo sapiens OX=9606 GN=RPS27 PE=1 SV=3	516.62	683.11	1.32	0.00608
Q96E39	RNA binding motif protein, X-linked-like-1 OS=Homo sapiens OX=9606 GN=RBMXL1 PE=1 SV=1	593.13	820.41	1.38	0.00610
P51160	Cone cGMP-specific 3',5'-cyclic phosphodiesterase subunit alpha' OS=Homo sapiens OX=9606 GN=PDE6C PE=1 SV=2	1073.08	1638.32	1.53	0.00618
P05141	ADP/ATP translocase 2 OS=Homo sapiens OX=9606 GN=SLC25A5 PE=1 SV=7	3059.45	5217.90	1.71	0.00626
P62280	40S ribosomal protein S11 OS=Homo sapiens OX=9606 GN=RPS11 PE=1 SV=3	1944.23	2800.11	1.44	0.00630
Q9H5Q4	Dimethyladenosine transferase 2, mitochondrial OS=Homo sapiens OX=9606 GN=TFB2M PE=1 SV=1	12.56	20.09	1.60	0.00633
Q7Z3H4	Sterile alpha motif domain-containing protein 7 OS=Homo sapiens OX=9606 GN=SAMD7 PE=1 SV=1	161.75	218.59	1.35	0.00650
Q5VZL5	Zinc finger MYM-type protein 4 OS=Homo sapiens OX=9606 GN=ZMYM4 PE=1 SV=1	163.47	258.68	1.58	0.00652
Q13151	Heterogeneous nuclear ribonucleoprotein A0 OS=Homo sapiens OX=9606 GN=HNRNPA0 PE=1 SV=1	2351.07	3023.49	1.29	0.00667
Q9GZY4	Cytochrome c oxidase assembly factor 1 homolog OS=Homo sapiens OX=9606 GN=COA1 PE=1 SV=1	54.72	75.69	1.38	0.00667
Q96A26	Protein FAM162A OS=Homo sapiens OX=9606 GN=FAM162A PE=1 SV=2	1199.35	1739.09	1.45	0.00670
O75152	Zinc finger CCCH domain-containing protein 11A OS=Homo sapiens OX=9606 GN=ZC3H11A PE=1 SV=3	1022.93	1358.09	1.33	0.00687
P32243	Homeobox protein OTX2 OS=Homo sapiens OX=9606 GN=OTX2 PE=1 SV=1	170.27	242.89	1.43	0.00703
P62244	40S ribosomal protein S15a OS=Homo sapiens OX=9606 GN=RPS15A PE=1 SV=2	801.52	1082.17	1.35	0.00710
Q8NBN7	Retinol dehydrogenase 13 OS=Homo sapiens OX=9606 GN=RDH13 PE=1 SV=2	561.69	818.18	1.46	0.00716
Q53GQ0	Very-long-chain 3-oxoacyl-CoA reductase OS=Homo sapiens OX=9606 GN=HSD17B12 PE=1 SV=2	2307.29	2907.23	1.26	0.00716
Q969S2	Endonuclease 8-like 2 OS=Homo sapiens OX=9606 GN=NEIL2 PE=1 SV=3	224.86	285.29	1.27	0.00718
P51659	Peroxisomal multifunctional enzyme type 2 OS=Homo sapiens OX=9606 GN=HSD17B4 PE=1 SV=3	2684.32	3420.04	1.27	0.00741
Q9NQC3	Isoform C of Reticulon-4 OS=Homo sapiens OX=9606 GN=RTN4	290.93	430.93	1.48	0.00761
O95831	Apoptosis-inducing factor 1, mitochondrial OS=Homo sapiens OX=9606 GN=AIFM1 PE=1 SV=1	3094.56	4916.30	1.59	0.00763
Q9HC07	Transmembrane protein 165 OS=Homo sapiens OX=9606 GN=TMEM165 PE=1 SV=1	96.07	132.91	1.38	0.00772
O00327	Aryl hydrocarbon receptor nuclear translocator-like protein 1 OS=Homo sapiens OX=9606 GN=ARNTL PE=1 SV=2	73.43	99.38	1.35	0.00782
P49916	DNA ligase 3 OS=Homo sapiens OX=9606 GN=LIG3 PE=1 SV=2	694.19	874.70	1.26	0.00790

Q6ZNB6	NF-X1-type zinc finger protein NFXL1 OS=Homo sapiens OX=9606 GN=NFXL1 PE=1 SV=2	136.94	180.31	1.32	0.00796
Q96CB8	Integrator complex subunit 12 OS=Homo sapiens OX=9606 GN=INTS12 PE=1 SV=1	150.70	211.05	1.40	0.00803
Q5SY16	Polynucleotide 5'-hydroxyl-kinase NOL9 OS=Homo sapiens OX=9606 GN=NOL9 PE=1 SV=1	64.82	90.73	1.40	0.00837
P42126	Enoyl-CoA delta isomerase 1, mitochondrial OS=Homo sapiens OX=9606 GN=ECI1 PE=1 SV=1	1261.55	1674.27	1.33	0.00852
A0A286YEX2	Synaptotagmin VI, isoform CRA_b OS=Homo sapiens OX=9606 GN=SYT6 PE=1 SV=1	28.57	44.37	1.55	0.00880
M0R2Z9	SURP and G-patch domain-containing protein 2 OS=Homo sapiens OX=9606 GN=SUGP2 PE=1 SV=1	3446.26	4619.53	1.34	0.00888
Q9H6K4	Optic atrophy 3 protein OS=Homo sapiens OX=9606 GN=OPA3 PE=1 SV=1	277.06	383.94	1.39	0.00892
Q13557	Isoform Delta 12 of Calcium/calmodulin-dependent protein kinase type II subunit delta OS=Homo sapiens OX=9606 GN=CAMK2D	75.28	115.98	1.54	0.00901
A0A0J9YXJ0	CUGBP Elav-like family member 2 OS=Homo sapiens OX=9606 GN=CELF2 PE=1 SV=1	825.94	1112.66	1.35	0.00904
O43913	Origin recognition complex subunit 5 OS=Homo sapiens OX=9606 GN=ORC5 PE=1 SV=1	209.90	264.16	1.26	0.00916
O95573	Long-chain-fatty-acid--CoA ligase 3 OS=Homo sapiens OX=9606 GN=ACSL3 PE=1 SV=3	3396.69	4474.53	1.32	0.00919
Q9UEG4	Zinc finger protein 629 OS=Homo sapiens OX=9606 GN=ZNF629 PE=1 SV=2	438.75	577.93	1.32	0.00927
P16499	Rod cGMP-specific 3',5'-cyclic phosphodiesterase subunit alpha OS=Homo sapiens OX=9606 GN=PDE6A PE=1 SV=4	5665.39	9688.89	1.71	0.00936
Q7Z7K6	Isoform 3 of Centromere protein V OS=Homo sapiens OX=9606 GN=CENPV	1164.57	1456.85	1.25	0.00940
Q6UX98	Probable palmitoyltransferase ZDHHC24 OS=Homo sapiens OX=9606 GN=ZDHHC24 PE=1 SV=1	60.01	79.57	1.33	0.00958
P12236	ADP/ATP translocase 3 OS=Homo sapiens OX=9606 GN=SLC25A6 PE=1 SV=4	3963.69	5987.79	1.51	0.00963
O95563	Mitochondrial pyruvate carrier 2 OS=Homo sapiens OX=9606 GN=MPC2 PE=1 SV=1	788.34	1117.64	1.42	0.00976
Q9UI40	Sodium/potassium/calcium exchanger 2 OS=Homo sapiens OX=9606 GN=SLC24A2 PE=1 SV=1	255.79	376.11	1.47	0.00984
Q5T3F8	CSC1-like protein 2 OS=Homo sapiens OX=9606 GN=TMEM63B PE=1 SV=1	445.29	593.43	1.33	0.00985
Q8WTQ7	Rhodopsin kinase OS=Homo sapiens OX=9606 GN=GRK7 PE=1 SV=1	339.33	448.62	1.32	0.00986
Q8N5G0	Isoform 2 of Small integral membrane protein 20 OS=Homo sapiens OX=9606 GN=SMIM20	217.36	293.38	1.35	0.00988
Q8TCJ2	Dolichyl-diphosphooligosaccharide--protein glycosyltransferase subunit STT3B OS=Homo sapiens OX=9606 GN=STT3B PE=1 SV=1	787.93	1002.15	1.27	0.00999
Q9BQQ3	Golgi reassembly-stacking protein 1 OS=Homo sapiens OX=9606 GN=GORASP1 PE=1 SV=3	55.20	70.65	1.28	0.01004
Q10471	Polypeptide N-acetylgalactosaminyltransferase 2 OS=Homo sapiens OX=9606 GN=GALNT2 PE=1 SV=1	871.64	1205.72	1.38	0.01006
Q96KR6	Protein FAM210B, mitochondrial OS=Homo sapiens OX=9606 GN=FAM210B PE=1 SV=2	10.54	20.83	1.98	0.01010

P0C7P0	CDGSH iron-sulfur domain-containing protein 3, mitochondrial OS=Homo sapiens OX=9606 GN=CISD3 PE=1 SV=1	175.58	229.71	1.31	0.01014
A0A2R8YFG2	Nicotinamide-nucleotide adenylyltransferase OS=Homo sapiens OX=9606 GN=NMNAT3 PE=1 SV=1	131.44	199.89	1.52	0.01023
Q9NPJ3	Acyl-coenzyme A thioesterase 13 OS=Homo sapiens OX=9606 GN=ACOT13 PE=1 SV=1	581.91	786.90	1.35	0.01028
O43521	Bcl-2-like protein 11 OS=Homo sapiens OX=9606 GN=BCL2L11 PE=1 SV=1	8.04	13.14	1.63	0.01032
Q96SI9	Spermatid perinuclear RNA-binding protein OS=Homo sapiens OX=9606 GN=STRBP PE=1 SV=1	1235.05	1555.53	1.26	0.01046
O43159	Ribosomal RNA-processing protein 8 OS=Homo sapiens OX=9606 GN=RRP8 PE=1 SV=2	285.81	388.10	1.36	0.01072
Q9UHQ9	NADH-cytochrome b5 reductase 1 OS=Homo sapiens OX=9606 GN=CYB5R1 PE=1 SV=1	1946.11	2486.74	1.28	0.01083
P12532	Creatine kinase U-type, mitochondrial OS=Homo sapiens OX=9606 GN=CKMT1A PE=1 SV=1	4658.90	6782.95	1.46	0.01085
A0A0D9SEI0	Uncharacterized protein C6orf203 OS=Homo sapiens OX=9606 GN=C6orf203 PE=1 SV=1	31.75	41.87	1.32	0.01091
I3L0E3	HCG1984214, isoform CRA_a OS=Homo sapiens OX=9606 GN=hCG_1984214 PE=4 SV=1	146.35	194.17	1.33	0.01110
J3KTA4	Probable ATP-dependent RNA helicase DDX5 OS=Homo sapiens OX=9606 GN=DDX5 PE=1 SV=1	3427.64	4738.96	1.38	0.01116
Q6PD62	RNA polymerase-associated protein CTR9 homolog OS=Homo sapiens OX=9606 GN=CTR9 PE=1 SV=1	599.73	755.58	1.26	0.01125
P84243	Histone H3.3 OS=Homo sapiens OX=9606 GN=H3F3A PE=1 SV=2	10890.96	14793.45	1.36	0.01127
O76081	Regulator of G-protein signaling 20 OS=Homo sapiens OX=9606 GN=RGS20 PE=1 SV=4	219.53	294.44	1.34	0.01149
Q9UKU0	Long-chain-fatty-acid--CoA ligase 6 OS=Homo sapiens OX=9606 GN=ACSL6 PE=2 SV=4	2997.24	3799.15	1.27	0.01152
O00567	Nucleolar protein 56 OS=Homo sapiens OX=9606 GN=NOP56 PE=1 SV=4	1421.10	1826.79	1.29	0.01154
F8VVT9	Arf-GAP with GTPase, ANK repeat and PH domain-containing protein 2 OS=Homo sapiens OX=9606 GN=AGAP2 PE=1 SV=1	17.50	24.49	1.40	0.01159
A0A140TA86	MICOS complex subunit MIC13 OS=Homo sapiens OX=9606 GN=C19orf70 PE=1 SV=1	689.77	1028.51	1.49	0.01160
Q9BPW8	Protein NipSnap homolog 1 OS=Homo sapiens OX=9606 GN=NIPSNAP1 PE=1 SV=1	2389.62	3133.30	1.31	0.01170
Q96AN5	Transmembrane protein 143 OS=Homo sapiens OX=9606 GN=TMEM143 PE=2 SV=1	116.48	149.12	1.28	0.01194
O60506	Isoform 3 of Heterogeneous nuclear ribonucleoprotein Q OS=Homo sapiens OX=9606 GN=SYNCRIP	26.41	33.51	1.27	0.01195
P21675	Isoform 4 of Transcription initiation factor TFIID subunit 1 OS=Homo sapiens OX=9606 GN=TAF1	83.91	109.09	1.30	0.01198
Q8WUY8	N-acetyltransferase 14 OS=Homo sapiens OX=9606 GN=NAT14 PE=1 SV=1	455.46	571.80	1.26	0.01198
Q5T8D3	Acyl-CoA-binding domain-containing protein 5 OS=Homo sapiens OX=9606 GN=ACBD5 PE=1 SV=1	621.92	782.82	1.26	0.01204
Q9UNL2	Isoform 2 of Translocon-associated protein subunit gamma OS=Homo sapiens OX=9606 GN=SSR3	148.11	186.53	1.26	0.01207

Q9NSE4	Isoleucine--tRNA ligase, mitochondrial OS=Homo sapiens OX=9606 GN=IARS2 PE=1 SV=2	4132.84	5203.64	1.26	0.01233
Q08211	ATP-dependent RNA helicase A OS=Homo sapiens OX=9606 GN=DHX9 PE=1 SV=4	7425.00	9505.02	1.28	0.01234
Q8N4Q0	Prostaglandin reductase 3 OS=Homo sapiens OX=9606 GN=ZADH2 PE=1 SV=1	311.09	426.11	1.37	0.01250
Q8N4T8	Carbonyl reductase family member 4 OS=Homo sapiens OX=9606 GN=CBR4 PE=1 SV=3	734.05	962.15	1.31	0.01254
Q96HJ9	Protein FMC1 homolog OS=Homo sapiens OX=9606 GN=FMC1 PE=1 SV=2	87.75	120.80	1.38	0.01263
Q12824	SWI/SNF-related matrix-associated actin-dependent regulator of chromatin subfamily B member 1 OS=Homo sapiens OX=9606 GN=SMARCB1 PE=1 SV=2	208.95	261.32	1.25	0.01265
Q9H7B2	Ribosome production factor 2 homolog OS=Homo sapiens OX=9606 GN=RPF2 PE=1 SV=2	40.52	68.28	1.68	0.01270
Q92618	Zinc finger protein 516 OS=Homo sapiens OX=9606 GN=ZNF516 PE=1 SV=1	128.54	177.34	1.38	0.01273
O95168	NADH dehydrogenase [ubiquinone] 1 beta subcomplex subunit 4 OS=Homo sapiens OX=9606 GN=NDUFB4 PE=1 SV=3	974.42	1348.82	1.38	0.01275
P00505	Aspartate aminotransferase, mitochondrial OS=Homo sapiens OX=9606 GN=GOT2 PE=1 SV=3	9965.94	12975.28	1.30	0.01277
J3QR07	YTH domain-containing protein 1 OS=Homo sapiens OX=9606 GN=YTHDC1 PE=1 SV=1	838.93	1065.40	1.27	0.01278
P15880	40S ribosomal protein S2 OS=Homo sapiens OX=9606 GN=RPS2 PE=1 SV=2	2971.84	3731.89	1.26	0.01295
P11488	Guanine nucleotide-binding protein G(t) subunit alpha-1 OS=Homo sapiens OX=9606 GN=GNAT1 PE=1 SV=5	6093.27	8679.41	1.42	0.01295
Q14938	Isoform 6 of Nuclear factor 1 X-type OS=Homo sapiens OX=9606 GN=NFIX	85.11	108.55	1.28	0.01314
Q5TZA2	Rootletin OS=Homo sapiens OX=9606 GN=CROCC PE=1 SV=1	10601.83	15821.05	1.49	0.01323
Q96T37	RNA-binding protein 15 OS=Homo sapiens OX=9606 GN=RBM15 PE=1 SV=2	673.82	845.85	1.26	0.01327
Q86U86	Protein polybromo-1 OS=Homo sapiens OX=9606 GN=PBRM1 PE=1 SV=1	585.33	734.28	1.25	0.01344
Q9NP73	Isoform 2 of Putative bifunctional UDP-N-acetylglucosamine transferase and deubiquitinase ALG13 OS=Homo sapiens OX=9606 GN=ALG13		267.18	346.60	1.29724745
P35913	Rod cGMP-specific 3',5'-cyclic phosphodiesterase subunit beta OS=Homo sapiens OX=9606 GN=PDE6B PE=1 SV=2	1415.37	2168.90	1.53	0.01370
Q96AQ8	Mitochondrial calcium uniporter regulator 1 OS=Homo sapiens OX=9606 GN=MCUR1 PE=1 SV=1	159.84	263.64	1.65	0.01385
Q02318	Sterol 26-hydroxylase, mitochondrial OS=Homo sapiens OX=9606 GN=CYP27A1 PE=1 SV=1	485.52	652.06	1.34	0.01392
P42167	Lamina-associated polypeptide 2, isoforms beta/gamma OS=Homo sapiens OX=9606 GN=TMPO PE=1 SV=2	260.55	338.35	1.30	0.01394
Q9Y5A9	YTH domain-containing family protein 2 OS=Homo sapiens OX=9606 GN=YTHDF2 PE=1 SV=2	278.90	378.80	1.36	0.01399
O95164	Ubiquitin-like protein 3 OS=Homo sapiens OX=9606 GN=UBL3 PE=1 SV=1	174.64	225.64	1.29	0.01409
E7ESZ7	NADH dehydrogenase [ubiquinone] 1 alpha subcomplex subunit 10, mitochondrial OS=Homo sapiens OX=9606 GN=NDUFA10 PE=1 SV=1	1518.87	2342.73	1.54	0.01411

Q5SNT2	Transmembrane protein 201 OS=Homo sapiens OX=9606 GN=TMEM201 PE=1 SV=1	685.19	923.77	1.35	0.01415
Q15059	Bromodomain-containing protein 3 OS=Homo sapiens OX=9606 GN=BRD3 PE=1 SV=1	697.01	889.63	1.28	0.01417
P51553	Isocitrate dehydrogenase [NAD] subunit gamma, mitochondrial OS=Homo sapiens OX=9606 GN=IDH3G PE=1 SV=1	1546.46	2003.09	1.30	0.01424
A0A0G2JPF8	Heterogeneous nuclear ribonucleoprotein C-like 4 OS=Homo sapiens OX=9606 GN=HNRNPCL4 PE=4 SV=1	334.34	463.55	1.39	0.01441
Q96RK4	Isoform 2 of Bardet-Biedl syndrome 4 protein OS=Homo sapiens OX=9606 GN=BBS4	242.29	307.05	1.27	0.01448
P20700	Lamin-B1 OS=Homo sapiens OX=9606 GN=LMNB1 PE=1 SV=2	8343.65	11350.53	1.36	0.01464
Q9GZR5	Elongation of very long chain fatty acids protein 4 OS=Homo sapiens OX=9606 GN=ELOVL4 PE=1 SV=1	557.56	740.06	1.33	0.01482
P50914	60S ribosomal protein L14 OS=Homo sapiens OX=9606 GN=RPL14 PE=1 SV=4	925.47	1194.18	1.29	0.01501
P50416	Carnitine O-palmitoyltransferase 1, liver isoform OS=Homo sapiens OX=9606 GN=CPT1A PE=1 SV=2	954.47	1271.01	1.33	0.01507
P12235	ADP/ATP translocase 1 OS=Homo sapiens OX=9606 GN=SLC25A4 PE=1 SV=4	2006.89	2831.13	1.41	0.01507
Q14119	Vascular endothelial zinc finger 1 OS=Homo sapiens OX=9606 GN=VEZF1 PE=1 SV=2	412.55	539.88	1.31	0.01522
Q5VU43	Isoform 2 of Myomegalin OS=Homo sapiens OX=9606 GN=PDE4DIP	681.84	856.97	1.26	0.01524
Q9ULX7	Carbonic anhydrase 14 OS=Homo sapiens OX=9606 GN=CA14 PE=1 SV=1	1337.80	1812.15	1.35	0.01530
Q9H5V9	UPF0428 protein CXorf56 OS=Homo sapiens OX=9606 GN=CXorf56 PE=1 SV=1	118.56	148.28	1.25	0.01552
A0A0R4J2E9	Interphotoreceptor matrix proteoglycan 1 OS=Homo sapiens OX=9606 GN=IMPG1 PE=1 SV=1	92.73	139.98	1.51	0.01560
O14681	Etoposide-induced protein 2.4 homolog OS=Homo sapiens OX=9606 GN=EI24 PE=1 SV=4	75.86	109.58	1.44	0.01571
Q8TDN2	Potassium voltage-gated channel subfamily V member 2 OS=Homo sapiens OX=9606 GN=KCNV2 PE=1 SV=1	249.16	371.18	1.49	0.01576
Q02040	A-kinase anchor protein 17A OS=Homo sapiens OX=9606 GN=AKAP17A PE=1 SV=2	245.87	311.72	1.27	0.01597
O75880	Protein SCO1 homolog, mitochondrial OS=Homo sapiens OX=9606 GN=SCO1 PE=1 SV=1	394.97	515.12	1.30	0.01611
Q9NV79	Protein-L-isoaspartate O-methyltransferase domain-containing protein 2 OS=Homo sapiens OX=9606 GN=PCMTD2 PE=2 SV=2	161.27	226.06	1.40	0.01646
Q9UGM6	Tryptophan--tRNA ligase, mitochondrial OS=Homo sapiens OX=9606 GN=WARS2 PE=1 SV=1	169.56	234.18	1.38	0.01650
A8MXP9	Matrin-3 OS=Homo sapiens OX=9606 GN=MATR3 PE=1 SV=1	8964.81	11239.55	1.25	0.01651
Q9BXC9	Bardet-Biedl syndrome 2 protein OS=Homo sapiens OX=9606 GN=BBS2 PE=1 SV=1	567.02	709.82	1.25	0.01659
B4DR61	cDNA FLJ59739, highly similar to Protein transport protein Sec61 subunit alpha isoform 1 OS=Homo sapiens OX=9606 GN=SEC61A1 PE=1 SV=1	456.85	600.92	1.32	0.01687
Q8TBF4	Zinc finger CCHC-type and RNA-binding motif-containing protein 1 OS=Homo sapiens OX=9606 GN=ZCRB1 PE=1 SV=2	113.95	153.35	1.35	0.01704

O43766	Lipoyl synthase, mitochondrial OS=Homo sapiens OX=9606 GN=LIAS PE=1 SV=3	9.67	12.79	1.32	0.01710
Q96ER9	Coiled-coil domain-containing protein 51 OS=Homo sapiens OX=9606 GN=CCDC51 PE=1 SV=2	278.69	350.81	1.26	0.01732
P51970	NADH dehydrogenase [ubiquinone] 1 alpha subcomplex subunit 8 OS=Homo sapiens OX=9606 GN=NDUFA8 PE=1 SV=3	1087.43	1631.25	1.50	0.01732
Q8IW92	Beta-galactosidase-1-like protein 2 OS=Homo sapiens OX=9606 GN=GLB1L2 PE=2 SV=1	146.73	194.41	1.32	0.01743
P62917	60S ribosomal protein L8 OS=Homo sapiens OX=9606 GN=RPL8 PE=1 SV=2	2031.33	2556.63	1.26	0.01744
Q6ZU65	Ubinuclein-2 OS=Homo sapiens OX=9606 GN=UBN2 PE=1 SV=2	70.68	90.12	1.27	0.01750
P43897	Elongation factor Ts, mitochondrial OS=Homo sapiens OX=9606 GN=TSFM PE=1 SV=2	821.75	1267.79	1.54	0.01779
Q9NVH1	DnaJ homolog subfamily C member 11 OS=Homo sapiens OX=9606 GN=DNAJC11 PE=1 SV=2	1604.84	2040.62	1.27	0.01787
P48047	ATP synthase subunit O, mitochondrial OS=Homo sapiens OX=9606 GN=ATP5PO PE=1 SV=1	4586.82	6567.65	1.43	0.01806
Q99766	ATP synthase subunit s, mitochondrial OS=Homo sapiens OX=9606 GN=DMAC2L PE=1 SV=3	453.22	590.28	1.30	0.01809
Q8TC12	Retinol dehydrogenase 11 OS=Homo sapiens OX=9606 GN=RDH11 PE=1 SV=2	1330.02	1692.86	1.27	0.01819
Q8IV63	Inactive serine/threonine-protein kinase VRK3 OS=Homo sapiens OX=9606 GN=VRK3 PE=1 SV=2	200.20	261.20	1.30	0.01822
P09874	Poly [ADP-ribose] polymerase 1 OS=Homo sapiens OX=9606 GN=PARP1 PE=1 SV=4	9120.96	12247.68	1.34	0.01829
Q12791	Isoform 7 of Calcium-activated potassium channel subunit alpha-1 OS=Homo sapiens OX=9606 GN=KCNMA1	69.85	125.61	1.80	0.01870
O15537	Retinoschisin OS=Homo sapiens OX=9606 GN=RS1 PE=1 SV=2	3679.26	5144.49	1.40	0.01879
Q8NBU5	ATPase family AAA domain-containing protein 1 OS=Homo sapiens OX=9606 GN=ATAD1 PE=1 SV=1	986.91	1262.03	1.28	0.01941
H7C1N3	BET1 homolog (Fragment) OS=Homo sapiens OX=9606 GN=BET1 PE=1 SV=1	232.18	304.81	1.31	0.01943
P56381	ATP synthase subunit epsilon, mitochondrial OS=Homo sapiens OX=9606 GN=ATP5F1E PE=1 SV=2	574.24	840.50	1.46	0.01944
Q9NVV4	Poly(A) RNA polymerase, mitochondrial OS=Homo sapiens OX=9606 GN=MTPAP PE=1 SV=1	344.21	485.88	1.41	0.01955
P49821	NADH dehydrogenase [ubiquinone] flavoprotein 1, mitochondrial OS=Homo sapiens OX=9606 GN=NDUFV1 PE=1 SV=4	4213.15	5757.12	1.37	0.01958
Q9Y6M9	NADH dehydrogenase [ubiquinone] 1 beta subcomplex subunit 9 OS=Homo sapiens OX=9606 GN=NDUFB9 PE=1 SV=3	946.06	1199.22	1.27	0.01970
F5GXJ1	NADH dehydrogenase [ubiquinone] iron-sulfur protein 7, mitochondrial OS=Homo sapiens OX=9606 GN=NDUFS7 PE=1 SV=1	1174.69	1632.12	1.39	0.01986
Q01538	Isoform 2 of Myelin transcription factor 1 OS=Homo sapiens OX=9606 GN=MYT1	150.39	190.15	1.26	0.02007
P50213	Isocitrate dehydrogenase [NAD] subunit alpha, mitochondrial OS=Homo sapiens OX=9606 GN=IDH3A PE=1 SV=1	3685.57	4729.34	1.28	0.02011

P40926	Malate dehydrogenase, mitochondrial OS=Homo sapiens OX=9606 GN=MDH2 PE=1 SV=3	7026.53	9236.31	1.31	0.02024
Q5BKZ1	DBIRD complex subunit ZNF326 OS=Homo sapiens OX=9606 GN=ZNF326 PE=1 SV=2	818.26	1115.44	1.36	0.02031
Q9NW81	Isoform 4 of Distal membrane-arm assembly complex protein 2 OS=Homo sapiens OX=9606 GN=DMAC2	103.53	140.94	1.36	0.02037
P21589	5'-nucleotidase OS=Homo sapiens OX=9606 GN=NT5E PE=1 SV=1	1411.74	1832.40	1.30	0.02112
P35250	Replication factor C subunit 2 OS=Homo sapiens OX=9606 GN=RFC2 PE=1 SV=3	168.94	221.13	1.31	0.02117
A0A0G2JPT5	Cell cycle checkpoint protein RAD17 OS=Homo sapiens OX=9606 GN=RAD17 PE=1 SV=1	44.77	60.08	1.34	0.02117
Q53H12	Acylglycerol kinase, mitochondrial OS=Homo sapiens OX=9606 GN=AGK PE=1 SV=2	1586.43	2012.75	1.27	0.02132
P55809	Succinyl-CoA:3-ketoacid coenzyme A transferase 1, mitochondrial OS=Homo sapiens OX=9606 GN=OXCT1 PE=1 SV=1	3830.84	5097.67	1.33	0.02135
Q9Y5X4	Photoreceptor-specific nuclear receptor OS=Homo sapiens OX=9606 GN=NR2E3 PE=1 SV=1	350.86	493.98	1.41	0.02165
P09669	Cytochrome c oxidase subunit 6C OS=Homo sapiens OX=9606 GN=COX6C PE=1 SV=2	1630.25	2263.91	1.39	0.02173
P24539	ATP synthase F(0) complex subunit B1, mitochondrial OS=Homo sapiens OX=9606 GN=ATP5PB PE=1 SV=2	3354.29	4531.22	1.35	0.02187
Q7Z3D6	Isoform 2 of D-glutamate cyclase, mitochondrial OS=Homo sapiens OX=9606 GN=DGLUCY	838.71	1135.88	1.35	0.02196
P39023	60S ribosomal protein L3 OS=Homo sapiens OX=9606 GN=RPL3 PE=1 SV=2	3527.34	4503.28	1.28	0.02196
P24310	Cytochrome c oxidase subunit 7A1, mitochondrial OS=Homo sapiens OX=9606 GN=COX7A1 PE=1 SV=2	142.06	185.56	1.31	0.02204
Q8N8Y2	V-type proton ATPase subunit d 2 OS=Homo sapiens OX=9606 GN=ATP6V0D2 PE=2 SV=1	12.85	18.61	1.45	0.02232
P21912	Succinate dehydrogenase [ubiquinone] iron-sulfur subunit, mitochondrial OS=Homo sapiens OX=9606 GN=SDHB PE=1 SV=3	2373.88	3233.29	1.36	0.02284
Q9BQ75	Protein CMSS1 OS=Homo sapiens OX=9606 GN=CMSS1 PE=1 SV=2	198.62	248.66	1.25	0.02287
A0A087WUC6	Signal peptidase complex subunit 2 OS=Homo sapiens OX=9606 GN=SPCS2 PE=1 SV=1	1038.68	1341.35	1.29	0.02325
Q9P0M6	Core histone macro-H2A.2 OS=Homo sapiens OX=9606 GN=H2AFY2 PE=1 SV=3	2055.40	2670.11	1.30	0.02333
P14866	Heterogeneous nuclear ribonucleoprotein L OS=Homo sapiens OX=9606 GN=HNRNPL PE=1 SV=2	8054.42	10695.98	1.33	0.02345
Q9NP92	39S ribosomal protein S30, mitochondrial OS=Homo sapiens OX=9606 GN=MRPS30 PE=1 SV=2	237.94	303.61	1.28	0.02381
P82914	28S ribosomal protein S15, mitochondrial OS=Homo sapiens OX=9606 GN=MRPS15 PE=1 SV=1	359.90	463.56	1.29	0.02389
Q9BYV7	Beta,beta-carotene 9',10'-oxygenase OS=Homo sapiens OX=9606 GN=BCO2 PE=1 SV=5	173.67	226.24	1.30	0.02406
H3BN98	Uncharacterized protein (Fragment) OS=Homo sapiens OX=9606 PE=4 SV=2	1218.14	1550.85	1.27	0.02423
Q9Y2D8	Afadin- and alpha-actinin-binding protein OS=Homo sapiens OX=9606 GN=SSX2IP PE=1 SV=3	440.93	580.45	1.32	0.02441

Q8N565	Isoform 2 of Melanoregulin OS=Homo sapiens OX=9606 GN=MREG	47.78	62.05	1.30	0.02465
O96000	NADH dehydrogenase [ubiquinone] 1 beta subcomplex subunit 10 OS=Homo sapiens OX=9606 GN=NDUFB10 PE=1 SV=3	1689.40	2336.24	1.38	0.02466
Q16795	NADH dehydrogenase [ubiquinone] 1 alpha subcomplex subunit 9, mitochondrial OS=Homo sapiens OX=9606 GN=NDUFA9 PE=1 SV=2	3946.99	5509.63	1.40	0.02477
Q9BVA0	Katanin p80 WD40 repeat-containing subunit B1 OS=Homo sapiens OX=9606 GN=KATNB1 PE=1 SV=1	41.81	52.88	1.26	0.02491
P28331	Isoform 2 of NADH-ubiquinone oxidoreductase 75 kDa subunit, mitochondrial OS=Homo sapiens OX=9606 GN=NDUFS1	7383.60	9577.38	1.30	0.02493
Q16718	NADH dehydrogenase [ubiquinone] 1 alpha subcomplex subunit 5 OS=Homo sapiens OX=9606 GN=NDUFA5 PE=1 SV=3	908.78	1300.59	1.43	0.02503
O43324	Eukaryotic translation elongation factor 1 epsilon-1 OS=Homo sapiens OX=9606 GN=EEF1E1 PE=1 SV=1	308.89	392.12	1.27	0.02537
Q08AF3	Schlafen family member 5 OS=Homo sapiens OX=9606 GN=SLFN5 PE=1 SV=1	64.58	88.01	1.36	0.02542
Q9HC52	Chromobox protein homolog 8 OS=Homo sapiens OX=9606 GN=CBX8 PE=1 SV=3	746.02	942.06	1.26	0.02548
Q14149	MORC family CW-type zinc finger protein 3 OS=Homo sapiens OX=9606 GN=MORC3 PE=1 SV=3	16.25	23.32	1.44	0.02568
P35398	Isoform 2 of Nuclear receptor ROR-alpha OS=Homo sapiens OX=9606 GN=RORA	80.34	108.53	1.35	0.02570
D6RGC4	E3 ubiquitin-protein ligase MARCH1 OS=Homo sapiens OX=9606 GN=MARCH1 PE=1 SV=1	22.26	31.00	1.39	0.02579
Q9H7N4	Splicing factor, arginine/serine-rich 19 OS=Homo sapiens OX=9606 GN=SCAF1 PE=1 SV=3	649.15	824.12	1.27	0.02640
Q9NRC6	Spectrin beta chain, non-erythrocytic 5 OS=Homo sapiens OX=9606 GN=SPTBN5 PE=1 SV=2	1119.69	1456.17	1.30	0.02720
P25445	Tumor necrosis factor receptor superfamily member 6 OS=Homo sapiens OX=9606 GN=FAS PE=1 SV=1	64.73	93.16	1.44	0.02726
Q96A23-2	Isoform 2 of Copine-4 OS=Homo sapiens OX=9606 GN=CPNE4	20.00	28.66	1.43	0.02766
A0A0B4J2F0	Protein PIGBOS1 OS=Homo sapiens OX=9606 GN=PIGBOS1 PE=3 SV=1	83.66	186.85	2.23	0.02767
Q01850	Cerebellar degeneration-related protein 2 OS=Homo sapiens OX=9606 GN=CDR2 PE=1 SV=2	138.95	193.95	1.40	0.02770
Q9BVV7	Mitochondrial import inner membrane translocase subunit Tim21 OS=Homo sapiens OX=9606 GN=TIMM21 PE=1 SV=1	118.01	150.15	1.27	0.02781
P86397	Hydroxyacyl-thioester dehydratase type 2, mitochondrial OS=Homo sapiens OX=9606 GN=HTD2 PE=1 SV=1	124.44	165.96	1.33	0.02806
A0A2U3TZT0	Zinc finger protein 385B OS=Homo sapiens OX=9606 GN=ZNF385B PE=1 SV=1	300.66	384.93	1.28	0.02864
Q8N8R7	ARL14 effector protein OS=Homo sapiens OX=9606 GN=ARL14EP PE=1 SV=1	114.81	158.66	1.38	0.02867
Q96A72	Protein mago nashi homolog 2 OS=Homo sapiens OX=9606 GN=MAGOHB PE=1 SV=1	725.32	953.53	1.31	0.02889
Q8TAQ2	SWI/SNF complex subunit SMARCC2 OS=Homo sapiens OX=9606 GN=SMARCC2 PE=1 SV=1	17.85	24.79	1.39	0.02903

A0A075B6Q7	Disrupted in schizophrenia 1 protein OS=Homo sapiens OX=9606 GN=DISC1 PE=1 SV=1	53.56	71.24	1.33	0.02923
D6RDH5	Guanine nucleotide-binding protein subunit gamma OS=Homo sapiens OX=9606 GN=GNGT2 PE=1 SV=1	493.66	664.79	1.35	0.02937
F8W930	Insulin-like growth factor 2 mRNA-binding protein 2 OS=Homo sapiens OX=9606 GN=IGF2BP2 PE=1 SV=1	1514.45	1975.42	1.30	0.02956
Q9NX40	OCIA domain-containing protein 1 OS=Homo sapiens OX=9606 GN=OCIAD1 PE=1 SV=1	896.12	1142.06	1.27	0.02982
P17568	NADH dehydrogenase [ubiquinone] 1 beta subcomplex subunit 7 OS=Homo sapiens OX=9606 GN=NDUFB7 PE=1 SV=4	991.53	1313.44	1.32	0.02995
Q8IWN7	Retinitis pigmentosa 1-like 1 protein OS=Homo sapiens OX=9606 GN=RP1L1 PE=1 SV=5	374.50	477.47	1.27	0.03000
O43920	NADH dehydrogenase [ubiquinone] iron-sulfur protein 5 OS=Homo sapiens OX=9606 GN=NDUFS5 PE=1 SV=3	551.80	750.25	1.36	0.03162
P49411	Elongation factor Tu, mitochondrial OS=Homo sapiens OX=9606 GN=TUFM PE=1 SV=2	4369.87	6022.78	1.38	0.03172
Q9ULU4	Isoform 10 of Protein kinase C-binding protein 1 OS=Homo sapiens OX=9606 GN=ZMYND8	776.07	995.12	1.28	0.03182
P18077	60S ribosomal protein L35a OS=Homo sapiens OX=9606 GN=RPL35A PE=1 SV=2	858.11	1158.87	1.35	0.03214
O95182	NADH dehydrogenase [ubiquinone] 1 alpha subcomplex subunit 7 OS=Homo sapiens OX=9606 GN=NDUFA7 PE=1 SV=3	1481.76	1968.81	1.33	0.03217
Q9BWE0	Isoform 2 of Replication initiator 1 OS=Homo sapiens OX=9606 GN=REPIN1	246.48	353.16	1.43	0.03234
P61254	60S ribosomal protein L26 OS=Homo sapiens OX=9606 GN=RPL26 PE=1 SV=1	790.13	1117.43	1.41	0.03242
Q9BVG8	Isoform 5 of Kinesin-like protein KIFC3 OS=Homo sapiens OX=9606 GN=KIFC3	60.30	79.70	1.32	0.03247
O00257	E3 SUMO-protein ligase CBX4 OS=Homo sapiens OX=9606 GN=CBX4 PE=1 SV=3	121.17	175.49	1.45	0.03304
Q96ND0	Protein FAM210A OS=Homo sapiens OX=9606 GN=FAM210A PE=1 SV=2	525.75	675.19	1.28	0.03305
A0A2Y9D025	NADH dehydrogenase [ubiquinone] 1 alpha subcomplex subunit 6 OS=Homo sapiens OX=9606 GN=NDUFA6 PE=4 SV=1	1085.90	1548.14	1.43	0.03310
Q8IY26	Phospholipid phosphatase 6 OS=Homo sapiens OX=9606 GN=PLPP6 PE=1 SV=3	40.46	51.61	1.28	0.03337
A6NDA9	Isoform 2 of Leucine-rich repeat, immunoglobulin-like domain and transmembrane domain-containing protein 2 OS=Homo sapiens OX=9606 GN=LRIT2	190.45	301.63	1.58	0.03376
E9PN58	NAD-dependent protein deacetylase sirtuin-3, mitochondrial OS=Homo sapiens OX=9606 GN=SIRT3 PE=1 SV=1	14.48	19.64	1.36	0.03418
Q96JP9	Cadherin-related family member 1 OS=Homo sapiens OX=9606 GN=CDHR1 PE=1 SV=2	93.20	133.60	1.43	0.03424
A6NCE7	Microtubule-associated proteins 1A/1B light chain 3 beta 2 OS=Homo sapiens OX=9606 GN=MAP1LC3B2 PE=2 SV=1	758.11	997.87	1.32	0.03443
Q9UIV1	CCR4-NOT transcription complex subunit 7 OS=Homo sapiens OX=9606 GN=CNOT7 PE=1 SV=3	35.00	44.95	1.28	0.03469
P61129	Zinc finger CCCH domain-containing protein 6 OS=Homo sapiens OX=9606 GN=ZC3H6 PE=1 SV=2	41.59	54.47	1.31	0.03528
P23786	Carnitine O-palmitoyltransferase 2, mitochondrial OS=Homo sapiens OX=9606 GN=CPT2 PE=1 SV=2	1846.74	2412.11	1.31	0.03554

H0Y886	NADH dehydrogenase [ubiquinone] 1 beta subcomplex subunit 5, mitochondrial (Fragment) OS=Homo sapiens OX=9606 GN=NDUFB5 PE=1 SV=1	686.26	930.55	1.36	0.03583
O75306	NADH dehydrogenase [ubiquinone] iron-sulfur protein 2, mitochondrial OS=Homo sapiens OX=9606 GN=NDUFS2 PE=1 SV=2	3339.43	4239.08	1.27	0.03609
A6NCL7	Ankyrin repeat domain-containing protein 33B OS=Homo sapiens OX=9606 GN=ANKRD33B PE=3 SV=1	112.12	143.49	1.28	0.03611
Q4U2R6	39S ribosomal protein L51, mitochondrial OS=Homo sapiens OX=9606 GN=MRPL51 PE=1 SV=1	65.30	82.45	1.26	0.03621
Q5T9L3	Isoform 2 of Protein wntless homolog OS=Homo sapiens OX=9606 GN=WLS	209.52	265.35	1.27	0.03652
Q96DA6	Mitochondrial import inner membrane translocase subunit TIM14 OS=Homo sapiens OX=9606 GN=DNAJC19 PE=1 SV=3	659.50	831.13	1.26	0.03684
Q6UW78	Ubiquinol-cytochrome-c reductase complex assembly factor 3 OS=Homo sapiens OX=9606 GN=UQCC3 PE=1 SV=2	4.60	6.78	1.47	0.03693
Q96EK4	THAP domain-containing protein 11 OS=Homo sapiens OX=9606 GN=THAP11 PE=1 SV=2	40.34	53.56	1.33	0.03707
Q9Y6M7	Isoform 13 of Sodium bicarbonate cotransporter 3 OS=Homo sapiens OX=9606 GN=SLC4A7	986.63	1253.29	1.27	0.03721
Q9H0M0	NEDD4-like E3 ubiquitin-protein ligase WWP1 OS=Homo sapiens OX=9606 GN=WWP1 PE=1 SV=1	58.12	73.06	1.26	0.03769
O43490	Isoform 6 of Prominin-1 OS=Homo sapiens OX=9606 GN=PROM1	1373.75	2156.33	1.57	0.03829
P14415	Sodium/potassium-transporting ATPase subunit beta-2 OS=Homo sapiens OX=9606 GN=ATP1B2 PE=1 SV=3	3856.77	4906.22	1.27	0.03844
Q96FJ2	Dynein light chain 2, cytoplasmic OS=Homo sapiens OX=9606 GN=DYNLL2 PE=1 SV=1	1738.55	2206.07	1.27	0.03844
Q5TEC6	Histone H3 OS=Homo sapiens OX=9606 GN=HIST2H3PS2 PE=1 SV=1	1995.02	2639.12	1.32	0.03846
Q92522	Histone H1x OS=Homo sapiens OX=9606 GN=H1FX PE=1 SV=1	3692.40	4733.97	1.28	0.03874
P46199	Translation initiation factor IF-2, mitochondrial OS=Homo sapiens OX=9606 GN=MTIF2 PE=1 SV=2	298.78	403.10	1.35	0.03885
O60783	28S ribosomal protein S14, mitochondrial OS=Homo sapiens OX=9606 GN=MRPS14 PE=1 SV=1	15.81	20.10	1.27	0.03888
P45880	Isoform 1 of Voltage-dependent anion-selective channel protein 2 OS=Homo sapiens OX=9606 GN=VDAC2	9309.14	11981.46	1.29	0.03903
P13073	Cytochrome c oxidase subunit 4 isoform 1, mitochondrial OS=Homo sapiens OX=9606 GN=COX4I1 PE=1 SV=1	3735.93	5071.00	1.36	0.03960
O94925	Glutaminase kidney isoform, mitochondrial OS=Homo sapiens OX=9606 GN=GLS PE=1 SV=1	2301.15	3158.11	1.37	0.03970
P03928	ATP synthase protein 8 OS=Homo sapiens OX=9606 GN=MT-ATP8 PE=1 SV=1	607.08	835.02	1.38	0.04006
Q9UJS0	Calcium-binding mitochondrial carrier protein Aralar2 OS=Homo sapiens OX=9606 GN=SLC25A13 PE=1 SV=2	2378.74	3155.24	1.33	0.04022
Q9BWS9	Isoform 2 of Chitinase domain-containing protein 1 OS=Homo sapiens OX=9606 GN=CHID1	417.77	559.38	1.34	0.04092

O14730	Serine/threonine-protein kinase RIO3 OS=Homo sapiens OX=9606 GN=RIOK3 PE=1 SV=2	7.62	11.06	1.45	0.04094
Q9NWT8	Aurora kinase A-interacting protein OS=Homo sapiens OX=9606 GN=AURKAIP1 PE=1 SV=1	96.36	137.27	1.42	0.04117
P38159	RNA-binding motif protein, X chromosome OS=Homo sapiens OX=9606 GN=RBMX PE=1 SV=3	6283.71	8055.48	1.28	0.04129
O43676	NADH dehydrogenase [ubiquinone] 1 beta subcomplex subunit 3 OS=Homo sapiens OX=9606 GN=NDUFB3 PE=1 SV=3	632.75	888.93	1.40	0.04177
C9JFV4	Proline-, glutamic acid- and leucine-rich protein 1 OS=Homo sapiens OX=9606 GN=PELP1 PE=1 SV=2	296.23	383.23	1.29	0.04289
Q5JTZ9	Alanine--tRNA ligase, mitochondrial OS=Homo sapiens OX=9606 GN=AARS2 PE=1 SV=1	905.91	1152.73	1.27	0.04309
Q9ULG6	Isoform 5 of Cell cycle progression protein 1 OS=Homo sapiens OX=9606 GN=CCPG1	26.84	45.87	1.71	0.04313
Q9BWF3	RNA-binding protein 4 OS=Homo sapiens OX=9606 GN=RBM4 PE=1 SV=1	106.36	139.05	1.31	0.04350
E9PR30	40S ribosomal protein S30 OS=Homo sapiens OX=9606 GN=FAU PE=1 SV=1	182.70	295.32	1.62	0.04362
O94929	Actin-binding LIM protein 3 OS=Homo sapiens OX=9606 GN=ABLIM3 PE=1 SV=3	558.81	736.30	1.32	0.04386
O95139	NADH dehydrogenase [ubiquinone] 1 beta subcomplex subunit 6 OS=Homo sapiens OX=9606 GN=NDUFB6 PE=1 SV=3	1285.82	1611.76	1.25	0.04495
O60563	Cyclin-T1 OS=Homo sapiens OX=9606 GN=CCNT1 PE=1 SV=1	19.04	24.51	1.29	0.04542
Q13627	Isoform 1 of Dual specificity tyrosine-phosphorylation-regulated kinase 1A OS=Homo sapiens OX=9606 GN=DYRK1A	206.01	259.04	1.26	0.04595
A8CG34	Nuclear envelope pore membrane protein POM 121C OS=Homo sapiens OX=9606 GN=POM121C PE=1 SV=3	153.47	195.31	1.27	0.04671
O75964	ATP synthase subunit g, mitochondrial OS=Homo sapiens OX=9606 GN=ATP5MG PE=1 SV=3	1412.52	1819.90	1.29	0.04704
Q5VST6	Isoform 2 of Alpha/beta hydrolase domain-containing protein 17B OS=Homo sapiens OX=9606 GN=ABHD17B	29.79	42.10	1.41	0.04774
Q03001	Isoform 8 of Dystonin OS=Homo sapiens OX=9606 GN=DST	159.52	215.65	1.35	0.04826
O15042	U2 snRNP-associated SURP motif-containing protein OS=Homo sapiens OX=9606 GN=U2SURP PE=1 SV=2	1291.63	1624.79	1.26	0.04839
O60907	Isoform 2 of F-box-like/WD repeat-containing protein TBL1X OS=Homo sapiens OX=9606 GN=TBL1X	27.97	35.49	1.27	0.04851
O15523	ATP-dependent RNA helicase DDX3Y OS=Homo sapiens OX=9606 GN=DDX3Y PE=1 SV=2	98.58	187.93	1.91	0.04900
Q15696	U2 small nuclear ribonucleoprotein auxiliary factor 35 kDa subunit-related protein 2 OS=Homo sapiens OX=9606 GN=ZRSR2 PE=1 SV=2	61.37	82.41	1.34	0.04906
P11169	Solute carrier family 2, facilitated glucose transporter member 3 OS=Homo sapiens OX=9606 GN=SLC2A3 PE=1 SV=1	586.42	762.08	1.30	0.04975

AD, Alzheimer's disease; FC, fold-change (FC>1.2 included); NC, cognitively normal controls; $P<0.05$

Supplementary Table 6. Extended mass spectrometry data on significantly up-regulated proteins in the temporal cortex of Alzheimer's patients compared to cognitively normal controls.

Accession	Description	AD (Means)	NC (Means)	FC	*P*
Q08629	Testican-1 OS=Homo sapiens OX=9606 GN=SPOCK1 PE=1 SV=1	105.77	75.72	1.40	3.473E-06
Q9BQ69	ADP-ribose glycohydrolase MACROD1 OS=Homo sapiens OX=9606 GN=MACROD1 PE=1 SV=2	536.38	352.00	1.52	7.918E-06
P55209	Nucleosome assembly protein 1-like 1 OS=Homo sapiens OX=9606 GN=NAP1L1 PE=1 SV=1	898.47	724.28	1.24	1.185E-05
E9PLM6	Midkine OS=Homo sapiens OX=9606 GN=MDK PE=1 SV=1	252.71	64.77	3.90	1.708E-05
P10636	Isoform Tau-B of Microtubule-associated protein tau OS=Homo sapiens OX=9606 GN=MAPT	1314.76	775.57	1.70	4.838E-05
P26038	Moesin OS=Homo sapiens OX=9606 GN=MSN PE=1 SV=3	5392.89	4063.81	1.33	4.862E-05
Q13501	Sequestosome-1 OS=Homo sapiens OX=9606 GN=SQSTM1 PE=1 SV=1	569.61	384.19	1.48	8.192E-05
Q15149	Isoform 3 of Plectin OS=Homo sapiens OX=9606 GN=PLEC	54659.35	45015.76	1.21	8.867E-05
Q8NBJ4	Golgi membrane protein 1 OS=Homo sapiens OX=9606 GN=GOLM1 PE=1 SV=1	113.85	85.93	1.32	9.491E-05
Q86WU2	Probable D-lactate dehydrogenase, mitochondrial OS=Homo sapiens OX=9606 GN=LDHD PE=1 SV=1	267.92	212.95	1.26	0.00010
P35754	Glutaredoxin-1 OS=Homo sapiens OX=9606 GN=GLRX PE=1 SV=2	137.25	112.96	1.22	0.00014
Q5VZK9	F-actin-uncapping protein LRRC16A OS=Homo sapiens OX=9606 GN=CARMIL1 PE=1 SV=1	290.16	241.33	1.20	0.00015
P02795	Metallothionein-2 OS=Homo sapiens OX=9606 GN=MT2A PE=1 SV=1	1151.26	742.94	1.55	0.00016
Q9H4F8	Isoform 2 of SPARC-related modular calcium-binding protein 1 OS=Homo sapiens OX=9606 GN=SMOC1	502.37	316.93	1.59	0.00017
Q9NRQ2	Phospholipid scramblase 4 OS=Homo sapiens OX=9606 GN=PLSCR4 PE=1 SV=2	25.40	15.74	1.61	0.00019
Q15691	Microtubule-associated protein RP/EB family member 1 OS=Homo sapiens OX=9606 GN=MAPRE1 PE=1 SV=3	205.72	168.37	1.22	0.00020
Q9Y2Z9	Ubiquinone biosynthesis monooxygenase COQ6, mitochondrial OS=Homo sapiens OX=9606 GN=COQ6 PE=1 SV=2	91.33	69.77	1.31	0.00025
Q8N612	Isoform 2 of FTS and Hook-interacting protein OS=Homo sapiens OX=9606 GN=FAM160A2	56.64	41.74	1.36	0.00029
G3V1V0	Myosin light polypeptide 6 OS=Homo sapiens OX=9606 GN=MYL6 PE=1 SV=1	2766.74	2069.34	1.34	0.00031
P04908	Histone H2A type 1-B/E OS=Homo sapiens OX=9606 GN=HIST1H2AB PE=1 SV=2	1078.32	570.92	1.89	0.00031
Q9Y4J8	Isoform 10 of Dystrobrevin alpha OS=Homo sapiens OX=9606 GN=DTNA	313.27	246.66	1.27	0.00034
Q9ULH7	Myocardin-related transcription factor B OS=Homo sapiens OX=9606 GN=MRTFB PE=1 SV=3	1149.42	945.51	1.22	0.00035
F6UJY1	Putative hydroxypyruvate isomerase OS=Homo sapiens OX=9606 GN=HYI PE=1 SV=1	111.91	89.75	1.25	0.00040

Q96AQ6	Pre-B-cell leukemia transcription factor-interacting protein 1 OS=Homo sapiens OX=9606 GN=PBXIP1 PE=1 SV=1	1994.79	1570.55	1.27	0.00044
P50552	Vasodilator-stimulated phosphoprotein OS=Homo sapiens OX=9606 GN=VASP PE=1 SV=3	293.35	238.10	1.23	0.00046
O75348	V-type proton ATPase subunit G 1 OS=Homo sapiens OX=9606 GN=ATP6V1G1 PE=1 SV=3	645.45	504.41	1.28	0.00047
F6SYF8	Dickkopf-related protein 3 OS=Homo sapiens OX=9606 GN=DKK3 PE=1 SV=1	662.78	496.04	1.34	0.00047
J3KPP4	Cisplatin resistance-associated overexpressed protein, isoform CRA_b OS=Homo sapiens OX=9606 GN=LUC7L3 PE=1 SV=1	93.64	70.94	1.32	0.00049
P14649	Myosin light chain 6B OS=Homo sapiens OX=9606 GN=MYL6B PE=1 SV=1	1100.43	915.51	1.20	0.00052
O14558	Heat shock protein beta-6 OS=Homo sapiens OX=9606 GN=HSPB6 PE=1 SV=2	348.88	275.31	1.27	0.00053
A0A0G2JPD5	Microtubule-associated protein OS=Homo sapiens OX=9606 GN=MAPT PE=1 SV=1	17974.26	13117.27	1.37	0.00055
Q9H299	SH3 domain-binding glutamic acid-rich-like protein 3 OS=Homo sapiens OX=9606 GN=SH3BGRL3 PE=1 SV=1	673.91	559.75	1.20	0.00057
P21246	Pleiotrophin OS=Homo sapiens OX=9606 GN=PTN PE=1 SV=1	342.76	241.05	1.42	0.00058
P24043	Laminin subunit alpha-2 OS=Homo sapiens OX=9606 GN=LAMA2 PE=1 SV=4	496.79	400.82	1.24	0.00059
P56277	Cx9C motif-containing protein 4 OS=Homo sapiens OX=9606 GN=CMC4 PE=1 SV=1	232.85	190.25	1.22	0.00062
P02792	Ferritin light chain OS=Homo sapiens OX=9606 GN=FTL PE=1 SV=2	3409.31	2727.13	1.25	0.00064
Q9NWH9	SAFB-like transcription modulator OS=Homo sapiens OX=9606 GN=SLTM PE=1 SV=2	163.59	122.92	1.33	0.00064
P24593	Insulin-like growth factor-binding protein 5 OS=Homo sapiens OX=9606 GN=IGFBP5 PE=1 SV=1	105.30	68.40	1.54	0.00065
Q9UGM6	Tryptophan--tRNA ligase, mitochondrial OS=Homo sapiens OX=9606 GN=WARS2 PE=1 SV=1	183.36	152.28	1.20	0.00069
Q6DD88	Atlastin-3 OS=Homo sapiens OX=9606 GN=ATL3 PE=1 SV=1	216.04	173.65	1.24	0.00070
H7BYT1	Casein kinase I isoform delta OS=Homo sapiens OX=9606 GN=CSNK1D PE=1 SV=2	262.03	211.19	1.24	0.00071
Q9UQE7	Structural maintenance of chromosomes protein 3 OS=Homo sapiens OX=9606 GN=SMC3 PE=1 SV=2	810.43	672.43	1.21	0.00072
Q9Y2R0	Cytochrome c oxidase assembly factor 3 homolog, mitochondrial OS=Homo sapiens OX=9606 GN=COA3 PE=1 SV=1	512.25	411.97	1.24	0.00072
Q96DF8	Splicing factor ESS-2 homolog OS=Homo sapiens OX=9606 GN=ESS2 PE=1 SV=1	52.93	38.36	1.38	0.00074
P07108	Isoform 5 of Acyl-CoA-binding protein OS=Homo sapiens OX=9606 GN=DBI	5776.54	4084.47	1.41	0.00083
F8VWL3	FYVE, RhoGEF and PH domain-containing protein 4 OS=Homo sapiens OX=9606 GN=FGD4 PE=1 SV=1	224.62	187.00	1.20	0.00086
P08670	Vimentin OS=Homo sapiens OX=9606 GN=VIM PE=1 SV=4	24462.10	16885.08	1.45	0.00089
P28838	Cytosol aminopeptidase OS=Homo sapiens OX=9606 GN=LAP3 PE=1 SV=3	2818.43	2321.38	1.21	0.00091
Q14019	Coactosin-like protein OS=Homo sapiens OX=9606 GN=COTL1 PE=1 SV=3	1528.92	1197.70	1.28	0.00093
O15061	Synemin OS=Homo sapiens OX=9606 GN=SYNM PE=1 SV=2	1298.68	985.61	1.32	0.00093

Q96ME1	F-box/LRR-repeat protein 18 OS=Homo sapiens OX=9606 GN=FBXL18 PE=1 SV=2	401.29	286.78	1.40	0.00095
A0A1B0GV68	FERM, ARHGEF and pleckstrin domain-containing protein 1 OS=Homo sapiens OX=9606 GN=FARP1 PE=1 SV=1	138.96	107.24	1.30	0.00101
Q15149	Isoform 8 of Plectin OS=Homo sapiens OX=9606 GN=PLEC	299.89	212.07	1.41	0.00102
O75882	Attractin OS=Homo sapiens OX=9606 GN=ATRN PE=1 SV=2	161.48	126.25	1.28	0.00103
P17931	Galectin-3 OS=Homo sapiens OX=9606 GN=LGALS3 PE=1 SV=5	918.05	727.10	1.26	0.00112
P26599	Isoform 3 of Polypyrimidine tract-binding protein 1 OS=Homo sapiens OX=9606 GN=PTBP1	403.90	306.40	1.32	0.00125
O00461	Golgi integral membrane protein 4 OS=Homo sapiens OX=9606 GN=GOLIM4 PE=1 SV=1	25.52	15.70	1.63	0.00133
P00441	Superoxide dismutase [Cu-Zn] OS=Homo sapiens OX=9606 GN=SOD1 PE=1 SV=2	10641.47	8078.98	1.32	0.00141
O94779	Contactin-5 OS=Homo sapiens OX=9606 GN=CNTN5 PE=1 SV=2	169.94	124.36	1.37	0.00142
O00115	Deoxyribonuclease-2-alpha OS=Homo sapiens OX=9606 GN=DNASE2 PE=1 SV=2	328.90	265.01	1.24	0.00148
Q9UPY8	Isoform 2 of Microtubule-associated protein RP/EB family member 3 OS=Homo sapiens OX=9606 GN=MAPRE3	131.86	107.13	1.23	0.00149
P05067	Amyloid-beta A4 protein OS=Homo sapiens OX=9606 GN=APP PE=1 SV=3	2645.65	2009.13	1.32	0.00154
Q9UIK5	Tomoregulin-2 OS=Homo sapiens OX=9606 GN=TMEFF2 PE=1 SV=1	124.38	103.22	1.21	0.00157
Q6UXK2	Immunoglobulin superfamily containing leucine-rich repeat protein 2 OS=Homo sapiens OX=9606 GN=ISLR2 PE=2 SV=1	240.09	188.73	1.27	0.00165
Q09470	Potassium voltage-gated channel subfamily A member 1 OS=Homo sapiens OX=9606 GN=KCNA1 PE=1 SV=2	91.20	73.72	1.24	0.00165
Q9Y6M5	Zinc transporter 1 OS=Homo sapiens OX=9606 GN=SLC30A1 PE=1 SV=3	177.86	137.04	1.30	0.00176
P08294	Extracellular superoxide dismutase [Cu-Zn] OS=Homo sapiens OX=9606 GN=SOD3 PE=1 SV=2	483.50	398.39	1.21	0.00177
Q53GG5	PDZ and LIM domain protein 3 OS=Homo sapiens OX=9606 GN=PDLIM3 PE=1 SV=1	75.93	49.61	1.53	0.00179
Q641Q2	WASH complex subunit 2A OS=Homo sapiens OX=9606 GN=WASHC2A PE=1 SV=3	562.14	460.26	1.22	0.00179
Q9GZM7	Tubulointerstitial nephritis antigen-like OS=Homo sapiens OX=9606 GN=TINAGL1 PE=1 SV=1	128.70	92.63	1.39	0.00182
Q9Y4J8	Isoform 15 of Dystrobrevin alpha OS=Homo sapiens OX=9606 GN=DTNA	1226.01	930.39	1.32	0.00185
P16403	Histone H1.2 OS=Homo sapiens OX=9606 GN=HIST1H1C PE=1 SV=2	793.01	534.84	1.48	0.00188
Q96KQ4	Apoptosis-stimulating of p53 protein 1 OS=Homo sapiens OX=9606 GN=PPP1R13B PE=1 SV=3	144.02	115.17	1.25	0.00191
Q92522	Histone H1x OS=Homo sapiens OX=9606 GN=H1FX PE=1 SV=1	1349.25	1089.10	1.24	0.00193
Q15651	High mobility group nucleosome-binding domain-containing protein 3 OS=Homo sapiens OX=9606 GN=HMGN3 PE=1 SV=2	57.15	41.13	1.39	0.00197
P05362	Intercellular adhesion molecule 1 OS=Homo sapiens OX=9606 GN=ICAM1 PE=1 SV=2	433.15	251.28	1.72	0.00204
Q9NQ92	Coordinator of PRMT5 and differentiation stimulator OS=Homo sapiens OX=9606 GN=COPRS PE=1 SV=3	104.51	75.32	1.39	0.00207

Q9HCB6	Spondin-1 OS=Homo sapiens OX=9606 GN=SPON1 PE=1 SV=2	107.07	74.17	1.44	0.00209
Q8IX12	Cell division cycle and apoptosis regulator protein 1 OS=Homo sapiens OX=9606 GN=CCAR1 PE=1 SV=2	46.09	36.75	1.25	0.00223
Q9Y676	28S ribosomal protein S18b, mitochondrial OS=Homo sapiens OX=9606 GN=MRPS18B PE=1 SV=1	86.96	64.83	1.34	0.00226
O43294	Transforming growth factor beta-1-induced transcript 1 protein OS=Homo sapiens OX=9606 GN=TGFB1I1 PE=1 SV=2	149.57	122.97	1.22	0.00227
A0A0G2JPP5	Protein scribble homolog OS=Homo sapiens OX=9606 GN=SCRIB PE=1 SV=1	266.82	218.88	1.22	0.00227
Q92562	Polyphosphoinositide phosphatase OS=Homo sapiens OX=9606 GN=FIG4 PE=1 SV=1	186.94	142.23	1.31	0.00235
Q9Y3D0	Cytosolic iron-sulfur assembly component 2B OS=Homo sapiens OX=9606 GN=CIAO2B PE=1 SV=1	81.17	61.81	1.31	0.00239
Q8NHP8	Putative phospholipase B-like 2 OS=Homo sapiens OX=9606 GN=PLBD2 PE=1 SV=2	40.04	25.05	1.60	0.00255
P09493	Tropomyosin alpha-1 chain OS=Homo sapiens OX=9606 GN=TPM1 PE=1 SV=2	266.25	209.96	1.27	0.00257
P02545	Prelamin-A/C OS=Homo sapiens OX=9606 GN=LMNA PE=1 SV=1	8480.83	6570.30	1.29	0.00261
Q8N5G2	Macoilin OS=Homo sapiens OX=9606 GN=MACO1 PE=1 SV=1	118.75	91.21	1.30	0.00264
P62072	Mitochondrial import inner membrane translocase subunit Tim10 OS=Homo sapiens OX=9606 GN=TIMM10 PE=1 SV=1	924.94	728.11	1.27	0.00269
B7Z1P2	F-box only protein 44 OS=Homo sapiens OX=9606 GN=FBXO44 PE=1 SV=1	116.35	95.64	1.22	0.00271
Q9C0C2	182 kDa tankyrase-1-binding protein OS=Homo sapiens OX=9606 GN=TNKS1BP1 PE=1 SV=4	772.31	640.65	1.21	0.00288
P04733	Metallothionein-1F OS=Homo sapiens OX=9606 GN=MT1F PE=1 SV=1	1251.57	847.80	1.48	0.00288
P55290	Isoform 4 of Cadherin-13 OS=Homo sapiens OX=9606 GN=CDH13	1432.05	1057.68	1.35	0.00292
P0C0S5	Histone H2A.Z OS=Homo sapiens OX=9606 GN=H2AFZ PE=1 SV=2	235.78	162.07	1.45	0.00304
Q93052	Lipoma-preferred partner OS=Homo sapiens OX=9606 GN=LPP PE=1 SV=1	401.76	292.98	1.37	0.00319
P04080	Cystatin-B OS=Homo sapiens OX=9606 GN=CSTB PE=1 SV=2	1288.63	1019.59	1.26	0.00337
J3KN67	Tropomyosin alpha-3 chain OS=Homo sapiens OX=9606 GN=TPM3 PE=1 SV=1	1647.11	1325.05	1.24	0.00348
Q9ULL4	Isoform 2 of Plexin-B3 OS=Homo sapiens OX=9606 GN=PLXNB3	29.04	21.77	1.33	0.00354
P10412	Histone H1.4 OS=Homo sapiens OX=9606 GN=HIST1H1E PE=1 SV=2	4345.19	3164.51	1.37	0.00355
A0A2R8Y4T1	Tensin-1 OS=Homo sapiens OX=9606 GN=TNS1 PE=1 SV=1	1133.38	939.53	1.21	0.00358
P62266	40S ribosomal protein S23 OS=Homo sapiens OX=9606 GN=RPS23 PE=1 SV=3	253.83	198.87	1.28	0.00361
Q8TE77	Protein phosphatase Slingshot homolog 3 OS=Homo sapiens OX=9606 GN=SSH3 PE=1 SV=2	50.20	32.70	1.54	0.00372
Q9H5X1	Cytosolic iron-sulfur assembly component 2A OS=Homo sapiens OX=9606 GN=CIAO2A PE=1 SV=1	102.59	76.99	1.33	0.00380
P16519	Neuroendocrine convertase 2 OS=Homo sapiens OX=9606 GN=PCSK2 PE=1 SV=2	217.32	174.37	1.25	0.00405
P04792	Heat shock protein beta-1 OS=Homo sapiens OX=9606 GN=HSPB1 PE=1 SV=2	3745.17	2957.65	1.27	0.00406

Q8WWX9	Selenoprotein M OS=Homo sapiens OX=9606 GN=SELENOM PE=1 SV=3	102.86	81.96	1.26	0.00420
Q9BRA0	Isoform 2 of N-alpha-acetyltransferase 38, NatC auxiliary subunit OS=Homo sapiens OX=9606 GN=NAA38	20.37	13.55	1.50	0.00422
P06753	Isoform 4 of Tropomyosin alpha-3 chain OS=Homo sapiens OX=9606 GN=TPM3	2977.57	2445.64	1.22	0.00427
U3KQK0	Histone H2B OS=Homo sapiens OX=9606 GN=HIST1H2BN PE=1 SV=1	7643.94	5760.87	1.33	0.00437
Q9NQZ5	StAR-related lipid transfer protein 7, mitochondrial OS=Homo sapiens OX=9606 GN=STARD7 PE=1 SV=2	226.13	180.73	1.25	0.00441
P27987	Inositol-trisphosphate 3-kinase B OS=Homo sapiens OX=9606 GN=ITPKB PE=1 SV=5	455.55	343.11	1.33	0.00450
E7ERK9	Translation initiation factor eIF-2B subunit delta OS=Homo sapiens OX=9606 GN=EIF2B4 PE=1 SV=1	33.34	16.25	2.05	0.00451
A0A0U1RRH7	Histone H2A OS=Homo sapiens OX=9606 PE=3 SV=1	4150.26	3332.62	1.25	0.00475
Q4VCS5	Angiomotin OS=Homo sapiens OX=9606 GN=AMOT PE=1 SV=1	232.50	187.82	1.24	0.00475
O00625	Pirin OS=Homo sapiens OX=9606 GN=PIR PE=1 SV=1	220.75	170.52	1.29	0.00492
P98160	Basement membrane-specific heparan sulfate proteoglycan core protein OS=Homo sapiens OX=9606 GN=HSPG2 PE=1 SV=4	1143.43	845.82	1.35	0.00494
O60245	Isoform B of Protocadherin-7 OS=Homo sapiens OX=9606 GN=PCDH7	194.80	150.86	1.29	0.00505
Q96HC4	PDZ and LIM domain protein 5 OS=Homo sapiens OX=9606 GN=PDLIM5 PE=1 SV=5	732.52	583.87	1.25	0.00511
P40121	Macrophage-capping protein OS=Homo sapiens OX=9606 GN=CAPG PE=1 SV=2	1477.09	1199.39	1.23	0.00524
Q9BPY8	Isoform 3 of Homeodomain-only protein OS=Homo sapiens OX=9606 GN=HOPX	290.22	231.42	1.25	0.00530
G3V3A4	SNW domain-containing protein 1 OS=Homo sapiens OX=9606 GN=SNW1 PE=1 SV=1	247.85	193.32	1.28	0.00537
Q9BZ72	Membrane-associated phosphatidylinositol transfer protein 2 OS=Homo sapiens OX=9606 GN=PITPNM2 PE=1 SV=1	364.36	282.29	1.29	0.00556
Q02779	Mitogen-activated protein kinase kinase kinase 10 OS=Homo sapiens OX=9606 GN=MAP3K10 PE=1 SV=3	43.93	32.24	1.36	0.00563
Q9H019	Mitochondrial fission regulator 1-like OS=Homo sapiens OX=9606 GN=MTFR1L PE=1 SV=2	118.16	94.57	1.25	0.00574
Q9UII2	ATPase inhibitor, mitochondrial OS=Homo sapiens OX=9606 GN=ATP5IF1 PE=1 SV=1	3307.04	2618.00	1.26	0.00574
H0YI08	Protein MRVI1 OS=Homo sapiens OX=9606 GN=MRVI1 PE=1 SV=1	78.42	60.34	1.30	0.00575
P34896	Serine hydroxymethyltransferase, cytosolic OS=Homo sapiens OX=9606 GN=SHMT1 PE=1 SV=1	148.39	119.92	1.24	0.00589
P31483	Nucleolysin TIA-1 isoform p40 OS=Homo sapiens OX=9606 GN=TIA1 PE=1 SV=3	103.45	77.69	1.33	0.00598
P09486	SPARC OS=Homo sapiens OX=9606 GN=SPARC PE=1 SV=1	56.82	43.96	1.29	0.00600
Q9UH65	Switch-associated protein 70 OS=Homo sapiens OX=9606 GN=SWAP70 PE=1 SV=1	267.43	215.74	1.24	0.00601
Q9NR28	Diablo homolog, mitochondrial OS=Homo sapiens OX=9606 GN=DIABLO PE=1 SV=1	591.61	488.40	1.21	0.00607
P56385	ATP synthase subunit e, mitochondrial OS=Homo sapiens OX=9606 GN=ATP5ME PE=1 SV=2	837.30	660.79	1.27	0.00620
Q8NHG7	Small VCP/p97-interacting protein OS=Homo sapiens OX=9606 GN=SVIP PE=1 SV=1	744.07	577.43	1.29	0.00628

A1X283	SH3 and PX domain-containing protein 2B OS=Homo sapiens OX=9606 GN=SH3PXD2B PE=1 SV=3	120.52	97.61	1.23	0.00628
Q8TEB1	DDB1- and CUL4-associated factor 11 OS=Homo sapiens OX=9606 GN=DCAF11 PE=1 SV=1	12.07	6.16	1.96	0.00638
P19838	Isoform 2 of Nuclear factor NF-kappa-B p105 subunit OS=Homo sapiens OX=9606 GN=NFKB1	21.67	16.23	1.34	0.00640
Q96RT1	Isoform 8 of Erbin OS=Homo sapiens OX=9606 GN=ERBIN	383.82	315.25	1.22	0.00651
P10909	Isoform 2 of Clusterin OS=Homo sapiens OX=9606 GN=CLU	3019.46	2366.84	1.28	0.00652
Q9UBI6	Guanine nucleotide-binding protein G(I)/G(S)/G(O) subunit gamma-12 OS=Homo sapiens OX=9606 GN=GNG12 PE=1 SV=3	436.75	344.54	1.27	0.00665
Q9NRR5	Ubiquilin-4 OS=Homo sapiens OX=9606 GN=UBQLN4 PE=1 SV=2	325.87	257.88	1.26	0.00672
Q96QE2	Proton myo-inositol cotransporter OS=Homo sapiens OX=9606 GN=SLC2A13 PE=1 SV=3	282.08	222.62	1.27	0.00673
P49773	Histidine triad nucleotide-binding protein 1 OS=Homo sapiens OX=9606 GN=HINT1 PE=1 SV=2	1430.14	1145.91	1.25	0.00683
Q8NEY1	Neuron navigator 1 OS=Homo sapiens OX=9606 GN=NAV1 PE=1 SV=2	249.58	196.94	1.27	0.00692
P04839	Cytochrome b-245 heavy chain OS=Homo sapiens OX=9606 GN=CYBB PE=1 SV=2	126.87	101.88	1.25	0.00702
M0QXZ5	Zinc finger protein 428 (Fragment) OS=Homo sapiens OX=9606 GN=ZNF428 PE=1 SV=1	23.62	16.38	1.44	0.00739
K7EMC7	Cytoglobin OS=Homo sapiens OX=9606 GN=CYGB PE=1 SV=1	41.76	28.36	1.47	0.00751
Q9UEE9	Craniofacial development protein 1 OS=Homo sapiens OX=9606 GN=CFDP1 PE=1 SV=1	403.16	325.66	1.24	0.00792
P41227	N-alpha-acetyltransferase 10 OS=Homo sapiens OX=9606 GN=NAA10 PE=1 SV=1	295.04	242.33	1.22	0.00794
Q15375	Ephrin type-A receptor 7 OS=Homo sapiens OX=9606 GN=EPHA7 PE=1 SV=3	71.15	45.81	1.55	0.00817
Q9Y371	Endophilin-B1 OS=Homo sapiens OX=9606 GN=SH3GLB1 PE=1 SV=1	53.42	43.46	1.23	0.00829
Q9Y2S6	Translation machinery-associated protein 7 OS=Homo sapiens OX=9606 GN=TMA7 PE=1 SV=1	412.93	248.99	1.66	0.00873
Q9NWB6	Arginine and glutamate-rich protein 1 OS=Homo sapiens OX=9606 GN=ARGLU1 PE=1 SV=1	739.84	607.26	1.22	0.00887
Q9Y5Z4	Heme-binding protein 2 OS=Homo sapiens OX=9606 GN=HEBP2 PE=1 SV=1	1155.34	913.17	1.27	0.00888
F5H7S3	Tropomyosin alpha-1 chain OS=Homo sapiens OX=9606 GN=TPM1 PE=1 SV=2	1690.85	1276.31	1.32	0.00900
Q08379	Golgin subfamily A member 2 OS=Homo sapiens OX=9606 GN=GOLGA2 PE=1 SV=3	86.25	58.64	1.47	0.00902
Q9HBT6	Cadherin-20 OS=Homo sapiens OX=9606 GN=CDH20 PE=2 SV=2	39.15	30.94	1.27	0.00918
A6NIW2	Dedicator of cytokinesis protein 11 OS=Homo sapiens OX=9606 GN=DOCK11 PE=1 SV=2	59.82	43.36	1.38	0.00938
Q8N1K5	Isoform 4 of Protein THEMIS OS=Homo sapiens OX=9606 GN=THEMIS	149.35	121.33	1.23	0.00961
O75509	Tumor necrosis factor receptor superfamily member 21 OS=Homo sapiens OX=9606 GN=TNFRSF21 PE=1 SV=1	27.65	17.28	1.60	0.00969
P02462	Collagen alpha-1(IV) chain OS=Homo sapiens OX=9606 GN=COL4A1 PE=1 SV=4	60.61	48.38	1.25	0.00980
P08123	Collagen alpha-2(I) chain OS=Homo sapiens OX=9606 GN=COL1A2 PE=1 SV=7	1642.41	1157.67	1.42	0.00987

P06703	Protein S100-A6 OS=Homo sapiens OX=9606 GN=S100A6 PE=1 SV=1	905.02	676.92	1.34	0.00992
P20309	Muscarinic acetylcholine receptor M3 OS=Homo sapiens OX=9606 GN=CHRM3 PE=1 SV=1	109.77	90.56	1.21	0.01004
M0QZI3	F-BAR domain only protein 1 OS=Homo sapiens OX=9606 GN=FCHO1 PE=1 SV=1	257.16	165.14	1.56	0.01007
O00233	26S proteasome non-ATPase regulatory subunit 9 OS=Homo sapiens OX=9606 GN=PSMD9 PE=1 SV=3	1094.79	890.46	1.23	0.01021
A0A096LNH6	Dedicator of cytokinesis protein 1 OS=Homo sapiens OX=9606 GN=DOCK1 PE=1 SV=1	459.34	371.19	1.24	0.01022
Q9NVK5	FGFR1 oncogene partner 2 OS=Homo sapiens OX=9606 GN=FGFR1OP2 PE=1 SV=1	123.61	97.52	1.27	0.01064
Q9BZL4	Protein phosphatase 1 regulatory subunit 12C OS=Homo sapiens OX=9606 GN=PPP1R12C PE=1 SV=1	93.48	64.74	1.44	0.01066
Q96E39	RNA binding motif protein, X-linked-like-1 OS=Homo sapiens OX=9606 GN=RBMXL1 PE=1 SV=1	607.57	454.38	1.34	0.01074
Q8IY22	C-Maf-inducing protein OS=Homo sapiens OX=9606 GN=CMIP PE=1 SV=3	111.00	89.49	1.24	0.01096
Q9NR46	Isoform 2 of Endophilin-B2 OS=Homo sapiens OX=9606 GN=SH3GLB2	495.62	400.03	1.24	0.01105
O95817	BAG family molecular chaperone regulator 3 OS=Homo sapiens OX=9606 GN=BAG3 PE=1 SV=3	645.72	482.68	1.34	0.01114
A0A0X1KG71	Negative elongation factor B OS=Homo sapiens OX=9606 GN=NELFB PE=1 SV=1	145.38	112.41	1.29	0.01114
Q9H098	Isoform 2 of Protein FAM107B OS=Homo sapiens OX=9606 GN=FAM107B	191.85	138.38	1.39	0.01156
Q8NDI1	Isoform 3 of EH domain-binding protein 1 OS=Homo sapiens OX=9606 GN=EHBP1	677.85	564.60	1.20	0.01168
Q9H0D6	5'-3' exoribonuclease 2 OS=Homo sapiens OX=9606 GN=XRN2 PE=1 SV=1	68.30	47.22	1.45	0.01188
Q9NX24	H/ACA ribonucleoprotein complex subunit 2 OS=Homo sapiens OX=9606 GN=NHP2 PE=1 SV=1	65.03	50.06	1.30	0.01196
Q86YR7	Probable guanine nucleotide exchange factor MCF2L2 OS=Homo sapiens OX=9606 GN=MCF2L2 PE=2 SV=3	38.25	28.07	1.36	0.01228
P06396	Gelsolin OS=Homo sapiens OX=9606 GN=GSN PE=1 SV=1	9802.51	7948.33	1.23	0.01232
Q9UPY6	Isoform 2 of Wiskott-Aldrich syndrome protein family member 3 OS=Homo sapiens OX=9606 GN=WASF3	40.78	27.09	1.51	0.01235
Q9UJU6	Isoform 3 of Drebrin-like protein OS=Homo sapiens OX=9606 GN=DBNL	77.29	60.10	1.29	0.01236
A0A2R8Y760	Collagen alpha-1(XXV) chain OS=Homo sapiens OX=9606 GN=COL25A1 PE=1 SV=1	87.37	39.07	2.24	0.01248
P61956	Small ubiquitin-related modifier 2 OS=Homo sapiens OX=9606 GN=SUMO2 PE=1 SV=3	1105.54	894.88	1.24	0.01321
Q10589	Bone marrow stromal antigen 2 OS=Homo sapiens OX=9606 GN=BST2 PE=1 SV=1	99.37	77.18	1.29	0.01341
Q15052	Rho guanine nucleotide exchange factor 6 OS=Homo sapiens OX=9606 GN=ARHGEF6 PE=1 SV=2	193.39	161.09	1.20	0.01347
Q13433	Zinc transporter ZIP6 OS=Homo sapiens OX=9606 GN=SLC39A6 PE=1 SV=3	73.51	59.38	1.24	0.01351
Q9Y3D2	Methionine-R-sulfoxide reductase B2, mitochondrial OS=Homo sapiens OX=9606 GN=MSRB2 PE=1 SV=2	73.45	60.11	1.22	0.01360
Q9ULX6	A-kinase anchor protein 8-like OS=Homo sapiens OX=9606 GN=AKAP8L PE=1 SV=4	85.25	66.17	1.29	0.01372

A0A087X0I6	Non-specific serine/threonine protein kinase OS=Homo sapiens OX=9606 GN=MARK1 PE=1 SV=1	20.66	15.49	1.33	0.01374
P21333	Filamin-A OS=Homo sapiens OX=9606 GN=FLNA PE=1 SV=4	10917.17	8601.05	1.27	0.01383
P09429	High mobility group protein B1 OS=Homo sapiens OX=9606 GN=HMGB1 PE=1 SV=3	1200.72	986.44	1.22	0.01386
O95263	High affinity cAMP-specific and IBMX-insensitive 3',5'-cyclic phosphodiesterase 8B OS=Homo sapiens OX=9606 GN=PDE8B PE=1 SV=2	338.01	280.25	1.21	0.01410
Q96CT7	Coiled-coil domain-containing protein 124 OS=Homo sapiens OX=9606 GN=CCDC124 PE=1 SV=1	325.57	266.67	1.22	0.01425
P15259	Phosphoglycerate mutase 2 OS=Homo sapiens OX=9606 GN=PGAM2 PE=1 SV=3	143.77	100.85	1.43	0.01456
Q9HC36	rRNA methyltransferase 3, mitochondrial OS=Homo sapiens OX=9606 GN=MRM3 PE=1 SV=2	173.08	126.78	1.37	0.01482
P12110	Collagen alpha-2(VI) chain OS=Homo sapiens OX=9606 GN=COL6A2 PE=1 SV=4	368.85	229.80	1.61	0.01538
Q12797	Aspartyl/asparaginyl beta-hydroxylase OS=Homo sapiens OX=9606 GN=ASPH PE=1 SV=3	463.92	384.07	1.21	0.01560
Q6GQQ9	OTU domain-containing protein 7B OS=Homo sapiens OX=9606 GN=OTUD7B PE=1 SV=1	293.06	244.05	1.20	0.01576
Q13177	Serine/threonine-protein kinase PAK 2 OS=Homo sapiens OX=9606 GN=PAK2 PE=1 SV=3	372.16	307.63	1.21	0.01607
Q9UJC3	Protein Hook homolog 1 OS=Homo sapiens OX=9606 GN=HOOK1 PE=1 SV=2	64.12	52.87	1.21	0.01630
Q8N4Q1	Isoform 2 of Mitochondrial intermembrane space import and assembly protein 40 OS=Homo sapiens OX=9606 GN=CHCHD4	269.77	198.29	1.36	0.01640
Q8NFU3	Thiosulfate:glutathione sulfurtransferase OS=Homo sapiens OX=9606 GN=TSTD1 PE=1 SV=3	278.44	192.97	1.44	0.01720
Q9H9J2	39S ribosomal protein L44, mitochondrial OS=Homo sapiens OX=9606 GN=MRPL44 PE=1 SV=1	162.78	135.22	1.20	0.01736
O14497	AT-rich interactive domain-containing protein 1A OS=Homo sapiens OX=9606 GN=ARID1A PE=1 SV=3	175.22	127.30	1.38	0.01742
E9PND2	Cysteine and glycine-rich protein 1 (Fragment) OS=Homo sapiens OX=9606 GN=CSRP1 PE=1 SV=1	66.56	48.51	1.37	0.01781
Q8NFW9	Rab effector MyRIP OS=Homo sapiens OX=9606 GN=MYRIP PE=1 SV=2	89.26	69.25	1.29	0.01785
P53384	Cytosolic Fe-S cluster assembly factor NUBP1 OS=Homo sapiens OX=9606 GN=NUBP1 PE=1 SV=2	23.66	13.58	1.74	0.01787
G3V4P8	Glia maturation factor beta (Fragment) OS=Homo sapiens OX=9606 GN=GMFB PE=1 SV=1	1596.94	1322.68	1.21	0.01792
P28906	Hematopoietic progenitor cell antigen CD34 OS=Homo sapiens OX=9606 GN=CD34 PE=1 SV=2	29.13	20.15	1.45	0.01836
P10599	Thioredoxin OS=Homo sapiens OX=9606 GN=TXN PE=1 SV=3	3058.50	2544.00	1.20	0.01852
Q96JN2	Isoform 4 of Coiled-coil domain-containing protein 136 OS=Homo sapiens OX=9606 GN=CCDC136	126.85	104.73	1.21	0.01868
Q86Y22	Collagen alpha-1(XXIII) chain OS=Homo sapiens OX=9606 GN=COL23A1 PE=1 SV=1	345.92	200.10	1.73	0.01869

O43291	Kunitz-type protease inhibitor 2 OS=Homo sapiens OX=9606 GN=SPINT2 PE=1 SV=2	81.30	55.93	1.45	0.01870
Q7Z422	SUZ domain-containing protein 1 OS=Homo sapiens OX=9606 GN=SZRD1 PE=1 SV=1	29.44	23.45	1.26	0.01872
Q8NFD5	Isoform 3 of AT-rich interactive domain-containing protein 1B OS=Homo sapiens OX=9606 GN=ARID1B	63.25	48.71	1.30	0.01926
Q96EU6	Ribosomal RNA processing protein 36 homolog OS=Homo sapiens OX=9606 GN=RRP36 PE=1 SV=1	67.09	48.32	1.39	0.01953
Q09666	Neuroblast differentiation-associated protein AHNAK OS=Homo sapiens OX=9606 GN=AHNAK PE=1 SV=2	13004.97	10807.21	1.20	0.01953
P82914	28S ribosomal protein S15, mitochondrial OS=Homo sapiens OX=9606 GN=MRPS15 PE=1 SV=1	214.40	170.46	1.26	0.01965
Q15847	Adipogenesis regulatory factor OS=Homo sapiens OX=9606 GN=ADIRF PE=1 SV=1	718.18	531.69	1.35	0.01973
Q9BRP8	Partner of Y14 and mago OS=Homo sapiens OX=9606 GN=PYM1 PE=1 SV=1	292.11	227.37	1.28	0.01993
A0A2R8Y596	Breast cancer anti-estrogen resistance protein 1 OS=Homo sapiens OX=9606 GN=BCAR1 PE=1 SV=1	71.70	50.43	1.42	0.02006
P62847	Isoform 4 of 40S ribosomal protein S24 OS=Homo sapiens OX=9606 GN=RPS24	660.47	547.04	1.21	0.02007
O75167	Isoform 4 of Phosphatase and actin regulator 2 OS=Homo sapiens OX=9606 GN=PHACTR2	56.71	41.84	1.36	0.02046
A0A1B0GTU4	Paxillin OS=Homo sapiens OX=9606 GN=PXN PE=1 SV=1	237.13	188.40	1.26	0.02047
Q9UGU0	Transcription factor 20 OS=Homo sapiens OX=9606 GN=TCF20 PE=1 SV=3	53.46	42.93	1.25	0.02064
Q13523	Serine/threonine-protein kinase PRP4 homolog OS=Homo sapiens OX=9606 GN=PRPF4B PE=1 SV=3	171.33	142.33	1.20	0.02084
O75369	Isoform 8 of Filamin-B OS=Homo sapiens OX=9606 GN=FLNB	2012.73	1608.61	1.25	0.02119
Q96Q15	Serine/threonine-protein kinase SMG1 OS=Homo sapiens OX=9606 GN=SMG1 PE=1 SV=3	122.24	98.57	1.24	0.02132
P57721	Poly(rC)-binding protein 3 OS=Homo sapiens OX=9606 GN=PCBP3 PE=2 SV=2	92.24	74.44	1.24	0.02139
P61254	60S ribosomal protein L26 OS=Homo sapiens OX=9606 GN=RPL26 PE=1 SV=1	212.64	173.55	1.23	0.02140
Q96SZ5	2-aminoethanethiol dioxygenase OS=Homo sapiens OX=9606 GN=ADO PE=1 SV=2	451.48	376.04	1.20	0.02145
P02649	Apolipoprotein E OS=Homo sapiens OX=9606 GN=APOE PE=1 SV=1	4225.28	3443.42	1.23	0.02198
E5RJD8	Tubulin-specific chaperone A OS=Homo sapiens OX=9606 GN=TBCA PE=1 SV=1	2039.19	1302.25	1.57	0.02208
P49619	Diacylglycerol kinase gamma OS=Homo sapiens OX=9606 GN=DGKG PE=2 SV=3	385.46	320.26	1.20	0.02211
B7Z2U2	TOM1-like protein 2 OS=Homo sapiens OX=9606 GN=TOM1L2 PE=1 SV=1	26.20	19.17	1.37	0.02237
Q8WVF1	Protein OSCP1 OS=Homo sapiens OX=9606 GN=OSCP1 PE=1 SV=4	126.70	102.63	1.23	0.02243
O95631	Netrin-1 OS=Homo sapiens OX=9606 GN=NTN1 PE=1 SV=2	26.30	18.76	1.40	0.02275
A0A2R8YDH4	Tight junction protein ZO-2 OS=Homo sapiens OX=9606 GN=TJP2 PE=3 SV=1	1288.19	1016.10	1.27	0.02290
Q15287	RNA-binding protein with serine-rich domain 1 OS=Homo sapiens OX=9606 GN=RNPS1 PE=1 SV=1	208.50	166.66	1.25	0.02315
Q5TZA2	Rootletin OS=Homo sapiens OX=9606 GN=CROCC PE=1 SV=1	237.49	197.77	1.20	0.02331

Q5GH76	XK-related protein 4 OS=Homo sapiens OX=9606 GN=XKR4 PE=2 SV=1	350.38	289.48	1.21	0.02335
Q14657	EKC/KEOPS complex subunit LAGE3 OS=Homo sapiens OX=9606 GN=LAGE3 PE=1 SV=2	31.65	23.71	1.34	0.02355
E9PGZ1	Caldesmon OS=Homo sapiens OX=9606 GN=CALD1 PE=1 SV=1	1532.30	1154.65	1.33	0.02362
P07919	Cytochrome b-c1 complex subunit 6, mitochondrial OS=Homo sapiens OX=9606 GN=UQCRH PE=1 SV=2	925.28	695.52	1.33	0.02379
A0A0J9YY34	NHS-like protein 2 OS=Homo sapiens OX=9606 GN=NHSL2 PE=1 SV=1	33.37	26.39	1.26	0.02417
Q15796	Mothers against decapentaplegic homolog 2 OS=Homo sapiens OX=9606 GN=SMAD2 PE=1 SV=1	75.95	55.92	1.36	0.02439
P28288	ATP-binding cassette sub-family D member 3 OS=Homo sapiens OX=9606 GN=ABCD3 PE=1 SV=1	142.36	115.42	1.23	0.02506
A0A096LP25	AP2-associated protein kinase 1 (Fragment) OS=Homo sapiens OX=9606 GN=AAK1 PE=1 SV=1	19.54	15.66	1.25	0.02549
Q14254	Flotillin-2 OS=Homo sapiens OX=9606 GN=FLOT2 PE=1 SV=2	2909.79	2115.91	1.38	0.02637
O95490	Isoform 6 of Adhesion G protein-coupled receptor L2 OS=Homo sapiens OX=9606 GN=ADGRL2	52.06	40.47	1.29	0.02662
Q86UU1	Pleckstrin homology-like domain family B member 1 OS=Homo sapiens OX=9606 GN=PHLDB1 PE=1 SV=1	315.93	262.95	1.20	0.02682
Q17R31	Isoform 3 of Putative deoxyribonuclease TATDN3 OS=Homo sapiens OX=9606 GN=TATDN3	137.61	111.81	1.23	0.02718
O43815	Striatin OS=Homo sapiens OX=9606 GN=STRN PE=1 SV=4	109.04	78.70	1.39	0.02751
P14209	CD99 antigen OS=Homo sapiens OX=9606 GN=CD99 PE=1 SV=1	392.15	290.47	1.35	0.02758
O15230	Laminin subunit alpha-5 OS=Homo sapiens OX=9606 GN=LAMA5 PE=1 SV=8	226.87	185.75	1.22	0.02767
Q6ZU35	Uncharacterized protein KIAA1211 OS=Homo sapiens OX=9606 GN=KIAA1211 PE=1 SV=3	26.62	19.81	1.34	0.02778
Q6KB66	Isoform 3 of Keratin, type II cytoskeletal 80 OS=Homo sapiens OX=9606 GN=KRT80	27.64	20.25	1.36	0.02785
Q53FA7	Quinone oxidoreductase PIG3 OS=Homo sapiens OX=9606 GN=TP53I3 PE=1 SV=2	234.87	177.64	1.32	0.02817
P20933	N(4)-(beta-N-acetylglucosaminyl)-L-asparaginase OS=Homo sapiens OX=9606 GN=AGA PE=1 SV=2	55.56	44.60	1.25	0.02830
Q96DC8	Enoyl-CoA hydratase domain-containing protein 3, mitochondrial OS=Homo sapiens OX=9606 GN=ECHDC3 PE=1 SV=2	108.00	77.52	1.39	0.02861
H0Y449	Nuclease-sensitive element-binding protein 1 (Fragment) OS=Homo sapiens OX=9606 GN=YBX1 PE=1 SV=1	32.69	21.10	1.55	0.02864
A1L188	NADH dehydrogenase [ubiquinone] 1 alpha subcomplex assembly factor 8 OS=Homo sapiens OX=9606 GN=NDUFAF8 PE=1 SV=1	18.25	14.23	1.28	0.02870
Q86WG5	Myotubularin-related protein 13 OS=Homo sapiens OX=9606 GN=SBF2 PE=1 SV=1	108.24	88.79	1.22	0.02916
Q9Y6N7	Isoform 2 of Roundabout homolog 1 OS=Homo sapiens OX=9606 GN=ROBO1	47.52	36.66	1.30	0.02950
Q13409	Cytoplasmic dynein 1 intermediate chain 2 OS=Homo sapiens OX=9606 GN=DYNC1I2 PE=1 SV=3	1582.12	1282.21	1.23	0.02958

P10636	Isoform Tau-F of Microtubule-associated protein tau OS=Homo sapiens OX=9606 GN=MAPT	11.55	6.87	1.68	0.03021
F5H757	Cytochrome b561 OS=Homo sapiens OX=9606 GN=CYB561 PE=1 SV=1	114.48	94.49	1.21	0.03062
Q9H446	RWD domain-containing protein 1 OS=Homo sapiens OX=9606 GN=RWDD1 PE=1 SV=1	319.45	252.05	1.27	0.03073
Q9UD71	Protein phosphatase 1 regulatory subunit 1B OS=Homo sapiens OX=9606 GN=PPP1R1B PE=1 SV=2	313.57	260.38	1.20	0.03085
P48509	CD151 antigen OS=Homo sapiens OX=9606 GN=CD151 PE=1 SV=3	120.07	85.33	1.41	0.03120
Q9BQT9	Isoform 2 of Calsyntenin-3 OS=Homo sapiens OX=9606 GN=CLSTN3	159.67	132.35	1.21	0.03143
P98095	Isoform 2 of Fibulin-2 OS=Homo sapiens OX=9606 GN=FBLN2	12.35	6.36	1.94	0.03149
Q9H3Q1	Cdc42 effector protein 4 OS=Homo sapiens OX=9606 GN=CDC42EP4 PE=1 SV=1	949.90	775.98	1.22	0.03150
Q9P2U8	Vesicular glutamate transporter 2 OS=Homo sapiens OX=9606 GN=SLC17A6 PE=2 SV=1	79.35	41.12	1.93	0.03160
Q9UJU6	Drebrin-like protein OS=Homo sapiens OX=9606 GN=DBNL PE=1 SV=1	2030.02	1533.91	1.32	0.03201
Q8WUW1	Isoform 2 of Protein BRICK1 OS=Homo sapiens OX=9606 GN=BRK1	268.72	207.54	1.29	0.03204
P17612	cAMP-dependent protein kinase catalytic subunit alpha OS=Homo sapiens OX=9606 GN=PRKACA PE=1 SV=2	93.89	70.94	1.32	0.03256
Q92835	Phosphatidylinositol 3,4,5-trisphosphate 5-phosphatase 1 OS=Homo sapiens OX=9606 GN=INPP5D PE=1 SV=2	77.16	60.45	1.28	0.03304
A0A087WY08	Thrombospondin type-1 domain-containing protein 7A OS=Homo sapiens OX=9606 GN=THSD7A PE=1 SV=1	62.46	49.99	1.25	0.03359
E3W994	CLIP-associating protein 2 OS=Homo sapiens OX=9606 GN=CLASP2 PE=1 SV=2	19.87	16.52	1.20	0.03377
Q5VWP3	Isoform 3 of Muscular LMNA-interacting protein OS=Homo sapiens OX=9606 GN=MLIP	205.69	169.18	1.22	0.03429
A0A0J9YX94	Paraneoplastic antigen Ma6F OS=Homo sapiens OX=9606 GN=PNMA6F PE=4 SV=1	56.30	46.67	1.21	0.03479
G3V164	Glutamate receptor 4 OS=Homo sapiens OX=9606 GN=GRIA4 PE=1 SV=1	21.78	10.11	2.15	0.03511
Q6UX71	Plexin domain-containing protein 2 OS=Homo sapiens OX=9606 GN=PLXDC2 PE=1 SV=1	38.71	28.01	1.38	0.03603
Q8IUE6	Histone H2A type 2-B OS=Homo sapiens OX=9606 GN=HIST2H2AB PE=1 SV=3	88.21	64.16	1.37	0.03616
Q96JN8	Neuralized-like protein 4 OS=Homo sapiens OX=9606 GN=NEURL4 PE=1 SV=2	43.64	34.52	1.26	0.03662
Q68CP4	Heparan-alpha-glucosaminide N-acetyltransferase OS=Homo sapiens OX=9606 GN=HGSNAT PE=1 SV=2	33.64	25.17	1.34	0.03689
Q9H1U4	Multiple epidermal growth factor-like domains protein 9 OS=Homo sapiens OX=9606 GN=MEGF9 PE=2 SV=3	42.11	31.89	1.32	0.03697
A0A0U1RQV1	Endogenous retrovirus group K3 member 1 (Fragment) OS=Homo sapiens OX=9606 GN=ERVK3-1 PE=1 SV=1	229.56	181.78	1.26	0.03708
O75347	Tubulin-specific chaperone A OS=Homo sapiens OX=9606 GN=TBCA PE=1 SV=3	699.36	524.96	1.33	0.03721
P30876	DNA-directed RNA polymerase II subunit RPB2 OS=Homo sapiens OX=9606 GN=POLR2B PE=1 SV=1	65.86	51.30	1.28	0.03732
Q7Z2W4	Zinc finger CCCH-type antiviral protein 1 OS=Homo sapiens OX=9606 GN=ZC3HAV1 PE=1 SV=3	270.06	214.66	1.26	0.03739

O60551	Glycylpeptide N-tetradecanoyltransferase 2 OS=Homo sapiens OX=9606 GN=NMT2 PE=1 SV=1	607.23	496.98	1.22	0.03809
Q13938	Calcyphosin OS=Homo sapiens OX=9606 GN=CAPS PE=1 SV=2	761.24	557.10	1.37	0.03854
E9PFU5	Pituitary adenylate cyclase-activating polypeptide type I receptor OS=Homo sapiens OX=9606 GN=ADCYAP1R1 PE=1 SV=1	88.83	68.21	1.30	0.03876
Q5VZ89	Isoform 2 of DENN domain-containing protein 4C OS=Homo sapiens OX=9606 GN=DENND4C	8.76	5.69	1.54	0.03926
P09132	Signal recognition particle 19 kDa protein OS=Homo sapiens OX=9606 GN=SRP19 PE=1 SV=3	341.06	273.35	1.25	0.03980
A0A0U1RQN6	Ankyrin-2 (Fragment) OS=Homo sapiens OX=9606 GN=ANK2 PE=1 SV=2	147.91	115.07	1.29	0.04001
P16402	Histone H1.3 OS=Homo sapiens OX=9606 GN=HIST1H1D PE=1 SV=2	2552.69	1973.97	1.29	0.04020
P29279	CCN family member 2 OS=Homo sapiens OX=9606 GN=CCN2 PE=1 SV=2	98.74	73.66	1.34	0.04143
P18615	Isoform 2 of Negative elongation factor E OS=Homo sapiens OX=9606 GN=NELFE	37.79	29.01	1.30	0.04213
Q5T200	Zinc finger CCCH domain-containing protein 13 OS=Homo sapiens OX=9606 GN=ZC3H13 PE=1 SV=1	68.32	54.12	1.26	0.04221
A0A0U1RQS6	Transmembrane protein 275 OS=Homo sapiens OX=9606 GN=TMEM275 PE=3 SV=1	53.00	43.14	1.23	0.04230
Q7Z7L8	Uncharacterized protein C11orf96 OS=Homo sapiens OX=9606 GN=C11orf96 PE=1 SV=3	168.41	138.38	1.22	0.04295
P09012	U1 small nuclear ribonucleoprotein A OS=Homo sapiens OX=9606 GN=SNRPA PE=1 SV=3	475.51	395.11	1.20	0.04390
P63092	Isoform 4 of Guanine nucleotide-binding protein G(s) subunit alpha isoforms short OS=Homo sapiens OX=9606 GN=GNAS	151.91	125.14	1.21	0.04413
Q15437	Protein transport protein Sec23B OS=Homo sapiens OX=9606 GN=SEC23B PE=1 SV=2	98.58	81.55	1.21	0.04440
B0YIW2	Apolipoprotein C-III variant 1 OS=Homo sapiens OX=9606 GN=APOC3 PE=1 SV=1	23.51	14.11	1.67	0.04444
Q69YU3	Ankyrin repeat domain-containing protein 34A OS=Homo sapiens OX=9606 GN=ANKRD34A PE=2 SV=2	32.05	26.37	1.22	0.04448
P12109	Collagen alpha-1(VI) chain OS=Homo sapiens OX=9606 GN=COL6A1 PE=1 SV=3	716.08	484.40	1.48	0.04494
Q32MZ4	Isoform 2 of Leucine-rich repeat flightless-interacting protein 1 OS=Homo sapiens OX=9606 GN=LRRFIP1	153.01	120.93	1.27	0.04524
P00519	Isoform IB of Tyrosine-protein kinase ABL1 OS=Homo sapiens OX=9606 GN=ABL1	97.88	75.82	1.29	0.04546
Q5T011	KICSTOR complex protein SZT2 OS=Homo sapiens OX=9606 GN=SZT2 PE=1 SV=3	57.35	47.61	1.20	0.04552
Q9NPF0	CD320 antigen OS=Homo sapiens OX=9606 GN=CD320 PE=1 SV=1	88.70	73.91	1.20	0.04577
A0A2R8YFG2	Nicotinamide-nucleotide adenylyltransferase OS=Homo sapiens OX=9606 GN=NMNAT3 PE=1 SV=1	194.50	155.89	1.25	0.04587
P08908	5-hydroxytryptamine receptor 1A OS=Homo sapiens OX=9606 GN=HTR1A PE=1 SV=3	61.01	41.17	1.48	0.04596
O60925	Prefoldin subunit 1 OS=Homo sapiens OX=9606 GN=PFDN1 PE=1 SV=2	269.32	223.33	1.21	0.04661
Q63ZY3	Isoform 2 of KN motif and ankyrin repeat domain-containing protein 2 OS=Homo sapiens OX=9606 GN=KANK2	88.53	73.71	1.20	0.04664

A0A087WTE6	SRC kinase-signaling inhibitor 1 (Fragment) OS=Homo sapiens OX=9606 GN=SRCIN1 PE=1 SV=1	33.93	23.51	1.44	0.04729
P04083	Annexin A1 OS=Homo sapiens OX=9606 GN=ANXA1 PE=1 SV=2	1124.28	910.62	1.23	0.04789
A9Z1X7	Serine/arginine repetitive matrix protein 1 OS=Homo sapiens OX=9606 GN=SRRM1 PE=1 SV=1	111.47	89.16	1.25	0.04791
Q96AE4	Far upstream element-binding protein 1 OS=Homo sapiens OX=9606 GN=FUBP1 PE=1 SV=3	806.68	669.36	1.21	0.04795
Q9BQ70	Transcription factor 25 OS=Homo sapiens OX=9606 GN=TCF25 PE=1 SV=1	107.48	87.96	1.22	0.04824
A0A1X7SBR1	Prickle-like protein 2 OS=Homo sapiens OX=9606 GN=PRICKLE2 PE=1 SV=1	263.97	195.51	1.35	0.04872
P05107	Integrin beta-2 OS=Homo sapiens OX=9606 GN=ITGB2 PE=1 SV=2	203.83	167.00	1.22	0.04913
Q9NRX2	39S ribosomal protein L17, mitochondrial OS=Homo sapiens OX=9606 GN=MRPL17 PE=1 SV=1	215.06	175.83	1.22	0.04915
P84101	Small EDRK-rich factor 2 OS=Homo sapiens OX=9606 GN=SERF2 PE=1 SV=1	453.48	356.96	1.27	0.04951
Q14247	Isoform 3 of Src substrate cortactin OS=Homo sapiens OX=9606 GN=CTTN	223.90	141.65	1.58	0.04953
Q9Y3Q8	TSC22 domain family protein 4 OS=Homo sapiens OX=9606 GN=TSC22D4 PE=1 SV=2	36.88	28.00	1.32	0.04994

AD, Alzheimer's disease; FC, fold-change (FC>1.2 included); NC, cognitively normal controls; $P<0.05$

Supplementary Table 7. Extended mass spectrometry data on significantly down-regulated proteins in the temporal cortex of Alzheimer's patients compared to cognitively normal controls.

Accession	Description	AD (Means)	NC (Means)	FC (*-1)	*P*
Q8NC96	Adaptin ear-binding coat-associated protein 1 OS=Homo sapiens OX=9606 GN=NECAP1 PE=1 SV=2	822.75	1065.81	1.30	1.894E-06
P84074	Neuron-specific calcium-binding protein hippocalcin OS=Homo sapiens OX=9606 GN=HPCA PE=1 SV=2	947.51	1322.95	1.40	9.479E-06
Q8IV01	Synaptotagmin-12 OS=Homo sapiens OX=9606 GN=SYT12 PE=1 SV=1	248.15	356.33	1.44	6.293E-05
O95716	Ras-related protein Rab-3D OS=Homo sapiens OX=9606 GN=RAB3D PE=1 SV=1	242.01	330.27	1.36	9.729E-05
F5GXV7	Neurobeachin OS=Homo sapiens OX=9606 GN=NBEA PE=1 SV=2	619.75	776.31	1.25	0.00017
Q9H061	Transmembrane protein 126A OS=Homo sapiens OX=9606 GN=TMEM126A PE=1 SV=1	600.37	839.21	1.40	0.00019
Q9H1E5	Thioredoxin-related transmembrane protein 4 OS=Homo sapiens OX=9606 GN=TMX4 PE=1 SV=1	82.50	114.56	1.39	0.00023
Q96BW9	Phosphatidate cytidylyltransferase, mitochondrial OS=Homo sapiens OX=9606 GN=TAMM41 PE=1 SV=2	154.42	238.23	1.54	0.00029
Q14642	Inositol polyphosphate-5-phosphatase A OS=Homo sapiens OX=9606 GN=INPP5A PE=1 SV=1	388.11	512.79	1.32	0.00030
P04156	Major prion protein OS=Homo sapiens OX=9606 GN=PRNP PE=1 SV=1	949.10	1207.63	1.27	0.00034
E9PNQ8	Thy-1 membrane glycoprotein (Fragment) OS=Homo sapiens OX=9606 GN=THY1 PE=1 SV=1	763.29	995.79	1.30	0.00042
P20645	Cation-dependent mannose-6-phosphate receptor OS=Homo sapiens OX=9606 GN=M6PR PE=1 SV=1	476.46	597.18	1.25	0.00045
Q8NBM8	Prenylcysteine oxidase-like OS=Homo sapiens OX=9606 GN=PCYOX1L PE=1 SV=2	140.59	189.15	1.35	0.00045
Q9BT40	Inositol polyphosphate 5-phosphatase K OS=Homo sapiens OX=9606 GN=INPP5K PE=1 SV=3	133.11	169.93	1.28	0.00046
Q9BTE1	Dynactin subunit 5 OS=Homo sapiens OX=9606 GN=DCTN5 PE=1 SV=1	249.45	312.29	1.25	0.00047
Q2KHT3	Protein CLEC16A OS=Homo sapiens OX=9606 GN=CLEC16A PE=1 SV=2	42.51	78.22	1.84	0.00050
Q99719	Septin-5 OS=Homo sapiens OX=9606 GN=SEPT5 PE=1 SV=1	1643.12	2357.81	1.43	0.00050
P61225	Ras-related protein Rap-2b OS=Homo sapiens OX=9606 GN=RAP2B PE=1 SV=1	862.47	1209.87	1.40	0.00051
J3KNF8	Cytochrome b5 type B (Outer mitochondrial membrane), isoform CRA_a OS=Homo sapiens OX=9606 GN=CYB5B PE=1 SV=1	244.39	327.41	1.34	0.00066
Q8N461	F-box/LRR-repeat protein 16 OS=Homo sapiens OX=9606 GN=FBXL16 PE=2 SV=2	210.97	264.37	1.25	0.00067
Q9H3Z4	DnaJ homolog subfamily C member 5 OS=Homo sapiens OX=9606 GN=DNAJC5 PE=1 SV=1	837.53	1160.09	1.39	0.00071
Q5VT52	Regulation of nuclear pre-mRNA domain-containing protein 2 OS=Homo sapiens OX=9606 GN=RPRD2 PE=1 SV=1	15.48	30.78	1.99	0.00073

P57740	Nuclear pore complex protein Nup107 OS=Homo sapiens OX=9606 GN=NUP107 PE=1 SV=1	116.01	146.03	1.26	0.00073
Q96AA8	Isoform 3 of Janus kinase and microtubule-interacting protein 2 OS=Homo sapiens OX=9606 GN=JAKMIP2	59.96	80.78	1.35	0.00077
P62166	Neuronal calcium sensor 1 OS=Homo sapiens OX=9606 GN=NCS1 PE=1 SV=2	524.46	737.21	1.41	0.00081
O75390	Citrate synthase, mitochondrial OS=Homo sapiens OX=9606 GN=CS PE=1 SV=2	4381.71	5495.39	1.25	0.00092
F2Z2X4	Exportin-4 OS=Homo sapiens OX=9606 GN=XPO4 PE=1 SV=1	27.38	35.98	1.31	0.00093
O95163	Elongator complex protein 1 OS=Homo sapiens OX=9606 GN=ELP1 PE=1 SV=3	21.73	33.41	1.54	0.00094
Q00325	Phosphate carrier protein, mitochondrial OS=Homo sapiens OX=9606 GN=SLC25A3 PE=1 SV=2	4191.96	5823.39	1.39	0.00095
Q93050	V-type proton ATPase 116 kDa subunit a isoform 1 OS=Homo sapiens OX=9606 GN=ATP6V0A1 PE=1 SV=3	4724.70	5996.51	1.27	0.00095
Q9NVA1	Ubiquinol-cytochrome-c reductase complex assembly factor 1 OS=Homo sapiens OX=9606 GN=UQCC1 PE=1 SV=3	157.71	228.43	1.45	0.00097
Q9H2U2	Isoform 2 of Inorganic pyrophosphatase 2, mitochondrial OS=Homo sapiens OX=9606 GN=PPA2	370.66	477.22	1.29	0.00102
P36542	ATP synthase subunit gamma, mitochondrial OS=Homo sapiens OX=9606 GN=ATP5F1C PE=1 SV=1	3478.55	4463.32	1.28	0.00103
P0C7M8	C-type lectin domain family 2 member L OS=Homo sapiens OX=9606 GN=CLEC2L PE=3 SV=1	106.75	243.36	2.28	0.00116
Q9UDW1	Cytochrome b-c1 complex subunit 9 OS=Homo sapiens OX=9606 GN=UQCR10 PE=1 SV=3	197.02	300.30	1.52	0.00129
Q53H12	Acylglycerol kinase, mitochondrial OS=Homo sapiens OX=9606 GN=AGK PE=1 SV=2	1188.82	1488.52	1.25	0.00131
P21579	Synaptotagmin-1 OS=Homo sapiens OX=9606 GN=SYT1 PE=1 SV=1	6183.22	8012.52	1.30	0.00133
J3QSW6	Kalirin OS=Homo sapiens OX=9606 GN=KALRN PE=1 SV=1	233.70	381.69	1.63	0.00148
Q16531	DNA damage-binding protein 1 OS=Homo sapiens OX=9606 GN=DDB1 PE=1 SV=1	2261.76	2948.49	1.30	0.00154
P30048	Thioredoxin-dependent peroxide reductase, mitochondrial OS=Homo sapiens OX=9606 GN=PRDX3 PE=1 SV=3	1718.20	2279.42	1.33	0.00161
Q9UK22	F-box only protein 2 OS=Homo sapiens OX=9606 GN=FBXO2 PE=1 SV=2	795.58	999.13	1.26	0.00170
P42704	Leucine-rich PPR motif-containing protein, mitochondrial OS=Homo sapiens OX=9606 GN=LRPPRC PE=1 SV=3	3261.35	4128.66	1.27	0.00175
Q86UY8	5'-nucleotidase domain-containing protein 3 OS=Homo sapiens OX=9606 GN=NT5DC3 PE=1 SV=1	347.33	506.85	1.46	0.00181
B5MDU6	Lipid droplet-associated hydrolase OS=Homo sapiens OX=9606 GN=LDAH PE=1 SV=1	252.83	329.71	1.30	0.00181
H7BYZ3	Uncharacterized protein OS=Homo sapiens OX=9606 PE=4 SV=1	473.39	736.33	1.56	0.00184
Q8WWI5	Isoform 2 of Choline transporter-like protein 1 OS=Homo sapiens OX=9606 GN=SLC44A1	854.84	1302.93	1.52	0.00188
Q96S97	Myeloid-associated differentiation marker OS=Homo sapiens OX=9606 GN=MYADM PE=1 SV=2	256.66	366.47	1.43	0.00189
Q9NQC3	Isoform C of Reticulon-4 OS=Homo sapiens OX=9606 GN=RTN4	75.50	138.56	1.84	0.00191

P46736	Lys-63-specific deubiquitinase BRCC36 OS=Homo sapiens OX=9606 GN=BRCC3 PE=1 SV=2	61.29	78.33	1.28	0.00192
Q8N9R8	Isoform 2 of Protein SCAI OS=Homo sapiens OX=9606 GN=SCAI	587.99	767.36	1.31	0.00200
Q7L1V2	Vacuolar fusion protein MON1 homolog B OS=Homo sapiens OX=9606 GN=MON1B PE=1 SV=1	35.25	56.22	1.59	0.00207
Q15904	V-type proton ATPase subunit S1 OS=Homo sapiens OX=9606 GN=ATP6AP1 PE=1 SV=2	252.13	368.51	1.46	0.00208
Q9H845	Acyl-CoA dehydrogenase family member 9, mitochondrial OS=Homo sapiens OX=9606 GN=ACAD9 PE=1 SV=1	838.08	1118.02	1.33	0.00210
O75306	NADH dehydrogenase [ubiquinone] iron-sulfur protein 2, mitochondrial OS=Homo sapiens OX=9606 GN=NDUFS2 PE=1 SV=2	1156.89	1576.62	1.36	0.00211
P45880	Isoform 1 of Voltage-dependent anion-selective channel protein 2 OS=Homo sapiens OX=9606 GN=VDAC2	6722.43	8604.01	1.28	0.00211
Q02978	Mitochondrial 2-oxoglutarate/malate carrier protein OS=Homo sapiens OX=9606 GN=SLC25A11 PE=1 SV=3	2289.49	3470.47	1.52	0.00214
P15531	Isoform 2 of Nucleoside diphosphate kinase A OS=Homo sapiens OX=9606 GN=NME1	239.63	307.61	1.28	0.00215
Q16795	NADH dehydrogenase [ubiquinone] 1 alpha subcomplex subunit 9, mitochondrial OS=Homo sapiens OX=9606 GN=NDUFA9 PE=1 SV=2	2107.21	2975.15	1.41	0.00225
O75689	Arf-GAP with dual PH domain-containing protein 1 OS=Homo sapiens OX=9606 GN=ADAP1 PE=1 SV=2	1512.04	1978.18	1.31	0.00229
Q96F07	Isoform 2 of Cytoplasmic FMR1-interacting protein 2 OS=Homo sapiens OX=9606 GN=CYFIP2	3728.54	4723.96	1.27	0.00229
A0A087WT12	Glutathione peroxidase OS=Homo sapiens OX=9606 GN=GPX4 PE=1 SV=1	1217.90	1524.79	1.25	0.00229
P27701	CD82 antigen OS=Homo sapiens OX=9606 GN=CD82 PE=1 SV=1	86.19	165.41	1.92	0.00230
A0A087WWT2	Neuritin OS=Homo sapiens OX=9606 GN=NRN1 PE=1 SV=1	333.47	450.12	1.35	0.00258
Q9NR31	GTP-binding protein SAR1a OS=Homo sapiens OX=9606 GN=SAR1A PE=1 SV=1	722.82	992.88	1.37	0.00270
P23469	Receptor-type tyrosine-protein phosphatase epsilon OS=Homo sapiens OX=9606 GN=PTPRE PE=1 SV=1	108.03	156.94	1.45	0.00273
P61601	Neurocalcin-delta OS=Homo sapiens OX=9606 GN=NCALD PE=1 SV=2	394.03	516.84	1.31	0.00292
P62244	40S ribosomal protein S15a OS=Homo sapiens OX=9606 GN=RPS15A PE=1 SV=2	190.92	248.23	1.30	0.00302
Q969L2	Protein MAL2 OS=Homo sapiens OX=9606 GN=MAL2 PE=1 SV=1	136.09	176.31	1.30	0.00308
P54803	Galactocerebrosidase OS=Homo sapiens OX=9606 GN=GALC PE=1 SV=3	121.16	362.06	2.99	0.00315
Q16186	Proteasomal ubiquitin receptor ADRM1 OS=Homo sapiens OX=9606 GN=ADRM1 PE=1 SV=2	308.10	389.11	1.26	0.00318
Q9GZN7	Protein rogdi homolog OS=Homo sapiens OX=9606 GN=ROGDI PE=1 SV=1	663.05	865.97	1.31	0.00318
P63096	Guanine nucleotide-binding protein G(i) subunit alpha-1 OS=Homo sapiens OX=9606 GN=GNAI1 PE=1 SV=2	937.27	1246.15	1.33	0.00321
Q12981	Isoform 3 of Vesicle transport protein SEC20 OS=Homo sapiens OX=9606 GN=BNIP1	132.39	179.95	1.36	0.00323
Q9Y276	Mitochondrial chaperone BCS1 OS=Homo sapiens OX=9606 GN=BCS1L PE=1 SV=1	429.73	608.00	1.41	0.00324

K7EJH8	Alpha-actinin-4 (Fragment) OS=Homo sapiens OX=9606 GN=ACTN4 PE=1 SV=1	31.74	42.20	1.33	0.00331
J3KQA9	Microtubule-associated tumor suppressor candidate 2 OS=Homo sapiens OX=9606 GN=MTUS2 PE=1 SV=1	26.50	45.22	1.71	0.00336
P0CG08	Golgi pH regulator B OS=Homo sapiens OX=9606 GN=GPR89B PE=1 SV=1	95.98	130.34	1.36	0.00337
A0A2R8YFE2	Actin, cytoplasmic 1 (Fragment) OS=Homo sapiens OX=9606 GN=ACTB PE=1 SV=1	95.52	126.57	1.33	0.00338
J3KR44	Ubiquitin thioesterase OS=Homo sapiens OX=9606 GN=OTUB1 PE=1 SV=2	1233.71	1550.46	1.26	0.00339
Q9Y376	Calcium-binding protein 39 OS=Homo sapiens OX=9606 GN=CAB39 PE=1 SV=1	677.67	986.55	1.46	0.00350
P21796	Voltage-dependent anion-selective channel protein 1 OS=Homo sapiens OX=9606 GN=VDAC1 PE=1 SV=2	9820.98	12600.37	1.28	0.00354
P04179	Superoxide dismutase [Mn], mitochondrial OS=Homo sapiens OX=9606 GN=SOD2 PE=1 SV=3	2948.09	3753.25	1.27	0.00355
P20337	Ras-related protein Rab-3B OS=Homo sapiens OX=9606 GN=RAB3B PE=1 SV=2	31.56	54.85	1.74	0.00362
Q96ND0	Protein FAM210A OS=Homo sapiens OX=9606 GN=FAM210A PE=1 SV=2	91.82	127.12	1.38	0.00370
A6NMU8	Neuroligin 4, Y-linked, isoform CRA_c OS=Homo sapiens OX=9606 GN=NLGN4Y PE=4 SV=1	99.11	127.41	1.29	0.00384
Q16799	Isoform RTN1-C of Reticulon-1 OS=Homo sapiens OX=9606 GN=RTN1	420.65	756.50	1.80	0.00403
P27449	V-type proton ATPase 16 kDa proteolipid subunit OS=Homo sapiens OX=9606 GN=ATP6V0C PE=1 SV=1	560.53	746.40	1.33	0.00426
C9JV77	Alpha-2-HS-glycoprotein OS=Homo sapiens OX=9606 GN=AHSG PE=1 SV=1	172.48	217.79	1.26	0.00443
P32189	Glycerol kinase OS=Homo sapiens OX=9606 GN=GK PE=1 SV=3	616.42	772.26	1.25	0.00479
A0A1X7SBR8	Voltage-dependent calcium channel gamma-8 subunit OS=Homo sapiens OX=9606 GN=CACNG8 PE=1 SV=1	574.17	730.12	1.27	0.00483
Q96DI7	Isoform 2 of U5 small nuclear ribonucleoprotein 40 kDa protein OS=Homo sapiens OX=9606 GN=SNRNP40	176.37	225.54	1.28	0.00486
F8WB74	Selenoprotein W OS=Homo sapiens OX=9606 GN=SELENOW PE=1 SV=1	139.92	177.39	1.27	0.00487
Q9UGP8	Translocation protein SEC63 homolog OS=Homo sapiens OX=9606 GN=SEC63 PE=1 SV=2	19.24	32.63	1.70	0.00516
Q9Y6A2	Cholesterol 24-hydroxylase OS=Homo sapiens OX=9606 GN=CYP46A1 PE=1 SV=1	84.69	109.21	1.29	0.00518
Q86V21	Acetoacetyl-CoA synthetase OS=Homo sapiens OX=9606 GN=AACS PE=1 SV=1	63.67	90.04	1.41	0.00523
Q14232	Translation initiation factor eIF-2B subunit alpha OS=Homo sapiens OX=9606 GN=EIF2B1 PE=1 SV=1	431.34	653.33	1.51	0.00524
E9PFW3	AP-2 complex subunit mu OS=Homo sapiens OX=9606 GN=AP2M1 PE=1 SV=1	4684.31	5868.05	1.25	0.00524
P20336	Ras-related protein Rab-3A OS=Homo sapiens OX=9606 GN=RAB3A PE=1 SV=1	3071.40	4157.31	1.35	0.00524
Q9NUJ1	Mycophenolic acid acyl-glucuronide esterase, mitochondrial OS=Homo sapiens OX=9606 GN=ABHD10 PE=1 SV=1	720.66	998.03	1.38	0.00527
Q9HD34	LYR motif-containing protein 4 OS=Homo sapiens OX=9606 GN=LYRM4 PE=1 SV=1	126.81	161.99	1.28	0.00528
Q96PB7	Noelin-3 OS=Homo sapiens OX=9606 GN=OLFM3 PE=1 SV=1	142.65	180.25	1.26	0.00539

Q5T0D9	Tumor protein p63-regulated gene 1-like protein OS=Homo sapiens OX=9606 GN=TPRG1L PE=1 SV=1	411.43	567.89	1.38	0.00545
P23396	40S ribosomal protein S3 OS=Homo sapiens OX=9606 GN=RPS3 PE=1 SV=2	1401.53	1908.79	1.36	0.00551
P19075	Tetraspanin-8 OS=Homo sapiens OX=9606 GN=TSPAN8 PE=1 SV=1	214.96	338.43	1.57	0.00561
P20339	Ras-related protein Rab-5A OS=Homo sapiens OX=9606 GN=RAB5A PE=1 SV=2	604.03	779.75	1.29	0.00565
P14678	Isoform SM-B1 of Small nuclear ribonucleoprotein-associated proteins B and B' OS=Homo sapiens OX=9606 GN=SNRPB	372.64	500.55	1.34	0.00573
O00232	26S proteasome non-ATPase regulatory subunit 12 OS=Homo sapiens OX=9606 GN=PSMD12 PE=1 SV=3	875.38	1200.39	1.37	0.00582
O15260	Surfeit locus protein 4 OS=Homo sapiens OX=9606 GN=SURF4 PE=1 SV=3	63.36	87.17	1.38	0.00589
A8MUM1	EARP and GARP complex-interacting protein 1 OS=Homo sapiens OX=9606 GN=EIPR1 PE=1 SV=2	439.07	587.81	1.34	0.00591
P18085	ADP-ribosylation factor 4 OS=Homo sapiens OX=9606 GN=ARF4 PE=1 SV=3	159.05	264.41	1.66	0.00600
Q00169	Phosphatidylinositol transfer protein alpha isoform OS=Homo sapiens OX=9606 GN=PITPNA PE=1 SV=2	1270.28	1776.64	1.40	0.00602
O95858	Tetraspanin-15 OS=Homo sapiens OX=9606 GN=TSPAN15 PE=1 SV=1	85.10	134.30	1.58	0.00603
Q13491	Isoform 4 of Neuronal membrane glycoprotein M6-b OS=Homo sapiens OX=9606 GN=GPM6B	1790.54	2280.03	1.27	0.00616
O14775	Guanine nucleotide-binding protein subunit beta-5 OS=Homo sapiens OX=9606 GN=GNB5 PE=1 SV=2	567.68	712.36	1.25	0.00624
P12235	ADP/ATP translocase 1 OS=Homo sapiens OX=9606 GN=SLC25A4 PE=1 SV=4	948.42	1509.10	1.59	0.00632
Q15119	[Pyruvate dehydrogenase (acetyl-transferring)] kinase isozyme 2, mitochondrial OS=Homo sapiens OX=9606 GN=PDK2 PE=1 SV=2	555.80	728.35	1.31	0.00637
A0A1W2PPJ5	Protein cereblon OS=Homo sapiens OX=9606 GN=CRBN PE=1 SV=1	296.85	402.70	1.36	0.00646
P61204	ADP-ribosylation factor 3 OS=Homo sapiens OX=9606 GN=ARF3 PE=1 SV=2	1222.31	1795.71	1.47	0.00652
Q86Y39	NADH dehydrogenase [ubiquinone] 1 alpha subcomplex subunit 11 OS=Homo sapiens OX=9606 GN=NDUFA11 PE=1 SV=3	301.35	498.15	1.65	0.00652
A0A024R4M0	40S ribosomal protein S9 OS=Homo sapiens OX=9606 GN=RPS9 PE=1 SV=1	1279.78	1629.30	1.27	0.00659
Q9Y277	Isoform 2 of Voltage-dependent anion-selective channel protein 3 OS=Homo sapiens OX=9606 GN=VDAC3	549.42	737.88	1.34	0.00668
Q53H82	Endoribonuclease LACTB2 OS=Homo sapiens OX=9606 GN=LACTB2 PE=1 SV=2	16.76	27.02	1.61	0.00679
Q9BXS5	Isoform 2 of AP-1 complex subunit mu-1 OS=Homo sapiens OX=9606 GN=AP1M1	782.34	991.58	1.27	0.00682
Q8TCJ2	Dolichyl-diphosphooligosaccharide--protein glycosyltransferase subunit STT3B OS=Homo sapiens OX=9606 GN=STT3B PE=1 SV=1	286.40	399.42	1.39	0.00694
Q99873	Protein arginine N-methyltransferase 1 OS=Homo sapiens OX=9606 GN=PRMT1 PE=1 SV=3	165.83	240.45	1.45	0.00711
O15144	Actin-related protein 2/3 complex subunit 2 OS=Homo sapiens OX=9606 GN=ARPC2 PE=1 SV=1	1016.03	1343.09	1.32	0.00717
Q96RD7	Pannexin-1 OS=Homo sapiens OX=9606 GN=PANX1 PE=1 SV=4	25.82	42.10	1.63	0.00739

O95197	Isoform 3 of Reticulon-3 OS=Homo sapiens OX=9606 GN=RTN3	102.85	150.41	1.46	0.00763
Q9Y6X5	Bis(5'-adenosyl)-triphosphatase ENPP4 OS=Homo sapiens OX=9606 GN=ENPP4 PE=1 SV=3	107.55	174.91	1.63	0.00769
P22694	cAMP-dependent protein kinase catalytic subunit beta OS=Homo sapiens OX=9606 GN=PRKACB PE=1 SV=2	913.56	1356.86	1.49	0.00780
P56545	Isoform 2 of C-terminal-binding protein 2 OS=Homo sapiens OX=9606 GN=CTBP2	79.41	108.94	1.37	0.00813
A0A0A0MQT0	Retinol binding protein 1, cellular OS=Homo sapiens OX=9606 GN=RBP1 PE=1 SV=1	386.06	484.95	1.26	0.00818
Q9UIQ6	Leucyl-cystinyl aminopeptidase OS=Homo sapiens OX=9606 GN=LNPEP PE=1 SV=3	118.23	170.43	1.44	0.00820
O95772	STARD3 N-terminal-like protein OS=Homo sapiens OX=9606 GN=STARD3NL PE=1 SV=1	105.04	150.44	1.43	0.00823
Q9UBQ5	Eukaryotic translation initiation factor 3 subunit K OS=Homo sapiens OX=9606 GN=EIF3K PE=1 SV=1	366.95	466.17	1.27	0.00823
Q9Y277	Voltage-dependent anion-selective channel protein 3 OS=Homo sapiens OX=9606 GN=VDAC3 PE=1 SV=1	3781.00	5405.35	1.43	0.00830
P05141	ADP/ATP translocase 2 OS=Homo sapiens OX=9606 GN=SLC25A5 PE=1 SV=7	7252.71	9907.02	1.37	0.00833
Q7Z7H5	Transmembrane emp24 domain-containing protein 4 OS=Homo sapiens OX=9606 GN=TMED4 PE=1 SV=1	237.73	311.26	1.31	0.00850
Q7Z5G4	Golgin subfamily A member 7 OS=Homo sapiens OX=9606 GN=GOLGA7 PE=1 SV=2	387.60	491.86	1.27	0.00854
Q9NRZ5	1-acyl-sn-glycerol-3-phosphate acyltransferase delta OS=Homo sapiens OX=9606 GN=AGPAT4 PE=1 SV=1	131.26	175.30	1.34	0.00867
Q13936	Isoform 2 of Voltage-dependent L-type calcium channel subunit alpha-1C OS=Homo sapiens OX=9606 GN=CACNA1C	22.98	35.41	1.54	0.00885
P28676	Grancalcin OS=Homo sapiens OX=9606 GN=GCA PE=1 SV=2	151.92	205.78	1.35	0.00907
Q14240	Eukaryotic initiation factor 4A-II OS=Homo sapiens OX=9606 GN=EIF4A2 PE=1 SV=2	1815.45	2315.76	1.28	0.00916
Q7L1I2	Synaptic vesicle glycoprotein 2B OS=Homo sapiens OX=9606 GN=SV2B PE=1 SV=1	1878.96	2413.81	1.28	0.00957
Q9Y2Q5	Isoform 2 of Ragulator complex protein LAMTOR2 OS=Homo sapiens OX=9606 GN=LAMTOR2	118.52	156.14	1.32	0.00957
Q9NZ01	Very-long-chain enoyl-CoA reductase OS=Homo sapiens OX=9606 GN=TECR PE=1 SV=1	495.10	1013.58	2.05	0.01001
P08247	Synaptophysin OS=Homo sapiens OX=9606 GN=SYP PE=1 SV=3	2118.06	2832.64	1.34	0.01005
Q99643	Succinate dehydrogenase cytochrome b560 subunit, mitochondrial OS=Homo sapiens OX=9606 GN=SDHC PE=1 SV=1	43.79	119.01	2.72	0.01007
Q9UNZ2	Isoform 2 of NSFL1 cofactor p47 OS=Homo sapiens OX=9606 GN=NSFL1C	193.21	242.88	1.26	0.01013
A6NCE7	Microtubule-associated proteins 1A/1B light chain 3 beta 2 OS=Homo sapiens OX=9606 GN=MAP1LC3B2 PE=2 SV=1	251.59	336.37	1.34	0.01024
O75964	ATP synthase subunit g, mitochondrial OS=Homo sapiens OX=9606 GN=ATP5MG PE=1 SV=3	755.61	949.07	1.26	0.01025
Q9Y342	Plasmolipin OS=Homo sapiens OX=9606 GN=PLLP PE=1 SV=1	503.23	746.06	1.48	0.01027

Q53GQ0	Very-long-chain 3-oxoacyl-CoA reductase OS=Homo sapiens OX=9606 GN=HSD17B12 PE=1 SV=2	420.16	594.62	1.42	0.01061
Q6IA86	Isoform 6 of Elongator complex protein 2 OS=Homo sapiens OX=9606 GN=ELP2	29.02	47.17	1.63	0.01071
P52306	Isoform 5 of Rap1 GTPase-GDP dissociation stimulator 1 OS=Homo sapiens OX=9606 GN=RAP1GDS1	1657.36	2515.77	1.52	0.01104
Q8N414	PiggyBac transposable element-derived protein 5 OS=Homo sapiens OX=9606 GN=PGBD5 PE=1 SV=4	188.72	240.35	1.27	0.01139
Q96DZ9	CKLF-like MARVEL transmembrane domain-containing protein 5 OS=Homo sapiens OX=9606 GN=CMTM5 PE=1 SV=2	83.78	131.69	1.57	0.01153
O14656	Torsin-1A OS=Homo sapiens OX=9606 GN=TOR1A PE=1 SV=1	126.66	173.14	1.37	0.01157
A0A0A0MR60	Receptor-type tyrosine-protein phosphatase S OS=Homo sapiens OX=9606 GN=PTPRS PE=1 SV=1	150.33	239.92	1.60	0.01174
Q9P2U7	Vesicular glutamate transporter 1 OS=Homo sapiens OX=9606 GN=SLC17A7 PE=2 SV=1	1505.96	1936.15	1.29	0.01175
Q6PI78	Transmembrane protein 65 OS=Homo sapiens OX=9606 GN=TMEM65 PE=1 SV=2	396.94	524.99	1.32	0.01192
Q7Z4H8	KDEL motif-containing protein 2 OS=Homo sapiens OX=9606 GN=KDELC2 PE=1 SV=2	13.91	19.36	1.39	0.01219
Q0VDG4	Secernin-3 OS=Homo sapiens OX=9606 GN=SCRN3 PE=1 SV=1	299.83	390.26	1.30	0.01244
J9JIE6	Calcium load-activated calcium channel OS=Homo sapiens OX=9606 GN=TMCO1 PE=1 SV=1	142.68	181.48	1.27	0.01248
P62068	Ubiquitin carboxyl-terminal hydrolase 46 OS=Homo sapiens OX=9606 GN=USP46 PE=1 SV=1	72.79	93.27	1.28	0.01261
P43007	Neutral amino acid transporter A OS=Homo sapiens OX=9606 GN=SLC1A4 PE=1 SV=1	458.06	582.91	1.27	0.01268
O15258	Protein RER1 OS=Homo sapiens OX=9606 GN=RER1 PE=1 SV=1	227.66	312.78	1.37	0.01299
A8MWY0	UPF0577 protein KIAA1324-like OS=Homo sapiens OX=9606 GN=KIAA1324L PE=1 SV=2	79.83	115.75	1.45	0.01322
Q9P035	Very-long-chain (3R)-3-hydroxyacyl-CoA dehydratase 3 OS=Homo sapiens OX=9606 GN=HACD3 PE=1 SV=2	728.56	1039.95	1.43	0.01344
Q9NRZ7	Isoform 3 of 1-acyl-sn-glycerol-3-phosphate acyltransferase gamma OS=Homo sapiens OX=9606 GN=AGPAT3	645.42	814.66	1.26	0.01366
P61313	60S ribosomal protein L15 OS=Homo sapiens OX=9606 GN=RPL15 PE=1 SV=2	384.18	486.48	1.27	0.01387
Q8IYU2	E3 ubiquitin-protein ligase HACE1 OS=Homo sapiens OX=9606 GN=HACE1 PE=1 SV=2	39.66	75.53	1.90	0.01391
Q8N1A0	Keratin-like protein KRT222 OS=Homo sapiens OX=9606 GN=KRT222 PE=2 SV=1	79.23	99.07	1.25	0.01405
P61626	Lysozyme C OS=Homo sapiens OX=9606 GN=LYZ PE=1 SV=1	236.28	303.67	1.29	0.01417
Q7L5D6	Golgi to ER traffic protein 4 homolog OS=Homo sapiens OX=9606 GN=GET4 PE=1 SV=1	231.38	314.12	1.36	0.01425
E9PK54	Heat shock cognate 71 kDa protein (Fragment) OS=Homo sapiens OX=9606 GN=HSPA8 PE=1 SV=8	62.82	95.93	1.53	0.01445
Q8IVF5	Isoform 2 of T-lymphoma invasion and metastasis-inducing protein 2 OS=Homo sapiens OX=9606 GN=TIAM2	135.06	170.50	1.26	0.01446
Q9UHG3	Prenylcysteine oxidase 1 OS=Homo sapiens OX=9606 GN=PCYOX1 PE=1 SV=3	1250.40	1708.44	1.37	0.01458

Q8TB96	T-cell immunomodulatory protein OS=Homo sapiens OX=9606 GN=ITFG1 PE=1 SV=1	139.41	220.20	1.58	0.01481
Q15759	Mitogen-activated protein kinase 11 OS=Homo sapiens OX=9606 GN=MAPK11 PE=1 SV=2	27.49	34.87	1.27	0.01489
O75508	Claudin-11 OS=Homo sapiens OX=9606 GN=CLDN11 PE=1 SV=2	931.95	1755.88	1.88	0.01519
O43592	Exportin-T OS=Homo sapiens OX=9606 GN=XPOT PE=1 SV=2	418.12	625.84	1.50	0.01534
Q9BVC4	Target of rapamycin complex subunit LST8 OS=Homo sapiens OX=9606 GN=MLST8 PE=1 SV=1	115.07	174.32	1.51	0.01537
Q5HYK3	2-methoxy-6-polyprenyl-1,4-benzoquinol methylase, mitochondrial OS=Homo sapiens OX=9606 GN=COQ5 PE=1 SV=2	183.50	231.56	1.26	0.01549
P52434	Isoform 4 of DNA-directed RNA polymerases I, II, and III subunit RPABC3 OS=Homo sapiens OX=9606 GN=POLR2H	25.33	38.08	1.50	0.01557
P62495	Eukaryotic peptide chain release factor subunit 1 OS=Homo sapiens OX=9606 GN=ETF1 PE=1 SV=3	428.22	546.80	1.28	0.01593
P47870	Gamma-aminobutyric acid receptor subunit beta-2 OS=Homo sapiens OX=9606 GN=GABRB2 PE=1 SV=2	650.31	835.04	1.28	0.01610
Q9NS86	LanC-like protein 2 OS=Homo sapiens OX=9606 GN=LANCL2 PE=1 SV=1	1029.17	1405.93	1.37	0.01626
O43414	ERI1 exoribonuclease 3 OS=Homo sapiens OX=9606 GN=ERI3 PE=1 SV=2	157.60	241.55	1.53	0.01651
A0A0D9SF30	Neural cell adhesion molecule 1 (Fragment) OS=Homo sapiens OX=9606 GN=NCAM1 PE=1 SV=1	2289.56	2941.53	1.28	0.01659
O00410	Importin-5 OS=Homo sapiens OX=9606 GN=IPO5 PE=1 SV=4	1458.50	1869.17	1.28	0.01682
O43760	Isoform 2 of Synaptogyrin-2 OS=Homo sapiens OX=9606 GN=SYNGR2	81.74	122.19	1.49	0.01763
A0A087WVA1	Selenoprotein T OS=Homo sapiens OX=9606 GN=SELENOT PE=1 SV=1	30.43	46.11	1.52	0.01770
Q969M1	Mitochondrial import receptor subunit TOM40B OS=Homo sapiens OX=9606 GN=TOMM40L PE=1 SV=1	250.31	364.08	1.45	0.01785
E9PCN2	Guanylate cyclase soluble subunit beta-1 OS=Homo sapiens OX=9606 GN=GUCY1B1 PE=1 SV=1	464.20	587.32	1.27	0.01789
H0UI06	Cytochrome c oxidase subunit 7A2, mitochondrial OS=Homo sapiens OX=9606 GN=COX7A2 PE=1 SV=1	578.47	796.18	1.38	0.01793
B4DJ07	Exocyst complex component 7 OS=Homo sapiens OX=9606 GN=EXOC7 PE=1 SV=1	32.24	45.20	1.40	0.01806
P49720	Proteasome subunit beta type-3 OS=Homo sapiens OX=9606 GN=PSMB3 PE=1 SV=2	201.54	301.01	1.49	0.01821
Q8TDQ7	Glucosamine-6-phosphate isomerase 2 OS=Homo sapiens OX=9606 GN=GNPDA2 PE=1 SV=1	463.68	686.17	1.48	0.01843
P07099	Epoxide hydrolase 1 OS=Homo sapiens OX=9606 GN=EPHX1 PE=1 SV=1	956.55	1258.94	1.32	0.01850
Q9NR22	Protein arginine N-methyltransferase 8 OS=Homo sapiens OX=9606 GN=PRMT8 PE=1 SV=2	57.08	115.68	2.03	0.01875
A0A2Y9D025	NADH dehydrogenase [ubiquinone] 1 alpha subcomplex subunit 6 OS=Homo sapiens OX=9606 GN=NDUFA6 PE=4 SV=1	153.59	210.99	1.37	0.01926
P60981	Destrin OS=Homo sapiens OX=9606 GN=DSTN PE=1 SV=3	1122.96	1409.92	1.26	0.01955

Q5T653	39S ribosomal protein L2, mitochondrial OS=Homo sapiens OX=9606 GN=MRPL2 PE=1 SV=2	10.16	15.94	1.57	0.01958
Q9H8T0	Isoform 2 of AKT-interacting protein OS=Homo sapiens OX=9606 GN=AKTIP	42.61	58.39	1.37	0.01995
Q9H3K2	Growth hormone-inducible transmembrane protein OS=Homo sapiens OX=9606 GN=GHITM PE=1 SV=2	368.06	482.18	1.31	0.02028
Q9BZL1	Ubiquitin-like protein 5 OS=Homo sapiens OX=9606 GN=UBL5 PE=1 SV=1	102.03	146.85	1.44	0.02042
Q8NE35	Cytoplasmic polyadenylation element-binding protein 3 OS=Homo sapiens OX=9606 GN=CPEB3 PE=1 SV=2	141.69	180.83	1.28	0.02050
Q15363	Transmembrane emp24 domain-containing protein 2 OS=Homo sapiens OX=9606 GN=TMED2 PE=1 SV=1	161.50	213.96	1.32	0.02053
P47972	Neuronal pentraxin-2 OS=Homo sapiens OX=9606 GN=NPTX2 PE=1 SV=2	249.23	493.62	1.98	0.02064
K7EQD6	Vesicle-fusing ATPase (Fragment) OS=Homo sapiens OX=9606 GN=NSF PE=1 SV=1	88.07	203.20	2.31	0.02143
A0A0A0MRR7	U1 small nuclear ribonucleoprotein C OS=Homo sapiens OX=9606 GN=SNRPC PE=1 SV=1	95.53	129.32	1.35	0.02149
O00487	26S proteasome non-ATPase regulatory subunit 14 OS=Homo sapiens OX=9606 GN=PSMD14 PE=1 SV=1	533.79	668.57	1.25	0.02159
Q6PI48	Aspartate--tRNA ligase, mitochondrial OS=Homo sapiens OX=9606 GN=DARS2 PE=1 SV=1	253.47	320.39	1.26	0.02164
P30536	Translocator protein OS=Homo sapiens OX=9606 GN=TSPO PE=1 SV=3	222.42	369.50	1.66	0.02181
Q9H4L5	Oxysterol-binding protein-related protein 3 OS=Homo sapiens OX=9606 GN=OSBPL3 PE=1 SV=1	4.51	8.23	1.82	0.02204
Q9BR01	Sulfotransferase 4A1 OS=Homo sapiens OX=9606 GN=SULT4A1 PE=1 SV=2	131.92	164.95	1.25	0.02230
A0A0A0MQU3	Ubiquitin-conjugating enzyme E2 D2 OS=Homo sapiens OX=9606 GN=UBE2D2 PE=1 SV=1	282.30	398.02	1.41	0.02239
P60228	Eukaryotic translation initiation factor 3 subunit E OS=Homo sapiens OX=9606 GN=EIF3E PE=1 SV=1	551.09	694.21	1.26	0.02245
J3KNI1	Conserved oligomeric Golgi complex subunit 4 OS=Homo sapiens OX=9606 GN=COG4 PE=1 SV=1	19.00	28.44	1.50	0.02278
J3KNF5	Centrosomal protein of 290 kDa OS=Homo sapiens OX=9606 GN=CEP290 PE=1 SV=1	99.97	169.63	1.70	0.02345
J3KTA1	F-box and leucine-rich repeat protein 20, isoform CRA_a OS=Homo sapiens OX=9606 GN=FBXL20 PE=1 SV=1	96.27	120.86	1.26	0.02442
P17677	Isoform 2 of Neuromodulin OS=Homo sapiens OX=9606 GN=GAP43	7618.54	10551.77	1.39	0.02447
P42658	Dipeptidyl aminopeptidase-like protein 6 OS=Homo sapiens OX=9606 GN=DPP6 PE=1 SV=2	4638.72	6420.29	1.38	0.02502
Q9BQG1	Synaptotagmin-3 OS=Homo sapiens OX=9606 GN=SYT3 PE=2 SV=1	226.08	287.14	1.27	0.02516
Q9Y296	Trafficking protein particle complex subunit 4 OS=Homo sapiens OX=9606 GN=TRAPPC4 PE=1 SV=1	88.93	127.17	1.43	0.02519
O95847	Mitochondrial uncoupling protein 4 OS=Homo sapiens OX=9606 GN=SLC25A27 PE=2 SV=1	84.23	114.89	1.36	0.02695

Q6YP21	Kynurenine--oxoglutarate transaminase 3 OS=Homo sapiens OX=9606 GN=KYAT3 PE=1 SV=1	979.50	1309.13	1.34	0.02707
F8VYN9	ADP-ribosylation factor-like protein 1 OS=Homo sapiens OX=9606 GN=ARL1 PE=1 SV=1	65.35	108.38	1.66	0.02764
Q8TEA7	TBC domain-containing protein kinase-like protein OS=Homo sapiens OX=9606 GN=TBCK PE=1 SV=4	70.76	88.81	1.26	0.02804
Q9Y2J0	Isoform 2 of Rabphilin-3A OS=Homo sapiens OX=9606 GN=RPH3A	2222.47	2952.95	1.33	0.02919
Q5JTD7	Leucine-rich repeat-containing protein 73 OS=Homo sapiens OX=9606 GN=LRRC73 PE=2 SV=1	29.68	38.02	1.28	0.02979
Q8NBI5	Isoform 2 of Solute carrier family 43 member 3 OS=Homo sapiens OX=9606 GN=SLC43A3	74.82	124.45	1.66	0.03018
Q9NRX5	Serine incorporator 1 OS=Homo sapiens OX=9606 GN=SERINC1 PE=1 SV=1	86.64	187.03	2.16	0.03044
Q9NVV5	Androgen-induced gene 1 protein OS=Homo sapiens OX=9606 GN=AIG1 PE=1 SV=2	20.89	34.51	1.65	0.03065
Q9Y2T3	Isoform 3 of Guanine deaminase OS=Homo sapiens OX=9606 GN=GDA	3827.96	5024.03	1.31	0.03069
Q9C0H2	Isoform 4 of Protein tweety homolog 3 OS=Homo sapiens OX=9606 GN=TTYH3	101.45	132.61	1.31	0.03077
Q7KZN9	Cytochrome c oxidase assembly protein COX15 homolog OS=Homo sapiens OX=9606 GN=COX15 PE=1 SV=1	117.01	147.10	1.26	0.03087
P62330	ADP-ribosylation factor 6 OS=Homo sapiens OX=9606 GN=ARF6 PE=1 SV=2	279.67	373.50	1.34	0.03125
P26992	Ciliary neurotrophic factor receptor subunit alpha OS=Homo sapiens OX=9606 GN=CNTFR PE=1 SV=2	197.06	259.76	1.32	0.03147
P42261	Isoform 6 of Glutamate receptor 1 OS=Homo sapiens OX=9606 GN=GRIA1	876.91	1205.72	1.37	0.03164
O43759	Synaptogyrin-1 OS=Homo sapiens OX=9606 GN=SYNGR1 PE=1 SV=2	577.59	726.74	1.26	0.03164
M0QYZ2	AP complex subunit sigma OS=Homo sapiens OX=9606 GN=AP2S1 PE=1 SV=1	311.04	429.95	1.38	0.03174
A0A087WTF6	Neural cell adhesion molecule 1 OS=Homo sapiens OX=9606 GN=NCAM1 PE=1 SV=1	37.17	46.47	1.25	0.03366
Q96GZ6	Isoform 9 of Solute carrier family 41 member 3 OS=Homo sapiens OX=9606 GN=SLC41A3	24.68	34.82	1.41	0.03412
P46199	Translation initiation factor IF-2, mitochondrial OS=Homo sapiens OX=9606 GN=MTIF2 PE=1 SV=2	17.22	22.50	1.31	0.03511
O95470	Sphingosine-1-phosphate lyase 1 OS=Homo sapiens OX=9606 GN=SGPL1 PE=1 SV=3	91.84	151.01	1.64	0.03571
A0A0C4DFZ2	Arylsulfatase A OS=Homo sapiens OX=9606 GN=ARSA PE=1 SV=1	61.63	77.25	1.25	0.03590
Q2M3C7	A-kinase anchor protein SPHKAP OS=Homo sapiens OX=9606 GN=SPHKAP PE=1 SV=1	25.23	33.22	1.32	0.03595
P41217	Isoform 3 of OX-2 membrane glycoprotein OS=Homo sapiens OX=9606 GN=CD200	359.69	481.97	1.34	0.03607
Q75T13	GPI inositol-deacylase OS=Homo sapiens OX=9606 GN=PGAP1 PE=1 SV=1	242.43	345.54	1.43	0.03688
P03915	NADH-ubiquinone oxidoreductase chain 5 OS=Homo sapiens OX=9606 GN=MT-ND5 PE=1 SV=2	283.09	397.29	1.40	0.03701
P63244	Receptor of activated protein C kinase 1 OS=Homo sapiens OX=9606 GN=RACK1 PE=1 SV=3	984.54	1264.63	1.28	0.03710
O15240	Neurosecretory protein VGF OS=Homo sapiens OX=9606 GN=VGF PE=1 SV=2	1011.80	1395.75	1.38	0.03719

Q96DD7	Protein shisa-4 OS=Homo sapiens OX=9606 GN=SHISA4 PE=2 SV=3	148.17	195.11	1.32	0.03807
P61764	Syntaxin-binding protein 1 OS=Homo sapiens OX=9606 GN=STXBP1 PE=1 SV=1	4976.28	6254.46	1.26	0.03904
P00403	Cytochrome c oxidase subunit 2 OS=Homo sapiens OX=9606 GN=MT-CO2 PE=1 SV=1	1863.43	2369.33	1.27	0.03942
O43747	Isoform 2 of AP-1 complex subunit gamma-1 OS=Homo sapiens OX=9606 GN=AP1G1	331.63	433.89	1.31	0.04046
A0A1C7CYV9	Solute carrier family 22 member 17 OS=Homo sapiens OX=9606 GN=SLC22A17 PE=1 SV=1	47.36	63.46	1.34	0.04072
Q5SQH8	Isoform 4 of Uncharacterized protein C6orf136 OS=Homo sapiens OX=9606 GN=C6orf136	62.98	87.02	1.38	0.04158
Q9Y2H6	Fibronectin type-III domain-containing protein 3A OS=Homo sapiens OX=9606 GN=FNDC3A PE=1 SV=4	68.78	88.06	1.28	0.04188
P50583	Bis(5'-nucleosyl)-tetraphosphatase [asymmetrical] OS=Homo sapiens OX=9606 GN=NUDT2 PE=1 SV=3	70.54	94.65	1.34	0.04205
P51674	Neuronal membrane glycoprotein M6-a OS=Homo sapiens OX=9606 GN=GPM6A PE=1 SV=2	1952.66	2523.72	1.29	0.04272
Q9NWU2	Glucose-induced degradation protein 8 homolog OS=Homo sapiens OX=9606 GN=GID8 PE=1 SV=1	4.39	8.46	1.93	0.04432
Q8ND04	Isoform 2 of Protein SMG8 OS=Homo sapiens OX=9606 GN=SMG8	43.98	63.32	1.44	0.04453
Q13530	Serine incorporator 3 OS=Homo sapiens OX=9606 GN=SERINC3 PE=2 SV=2	116.36	162.03	1.39	0.04468
A0A0A0MSJ9	Ras GTPase-activating protein 2 OS=Homo sapiens OX=9606 GN=RASA2 PE=1 SV=1	106.64	136.36	1.28	0.04531
Q8N2U0	Transmembrane protein 256 OS=Homo sapiens OX=9606 GN=TMEM256 PE=3 SV=1	24.16	38.70	1.60	0.04538
O14787	Isoform 2 of Transportin-2 OS=Homo sapiens OX=9606 GN=TNPO2	126.37	189.28	1.50	0.04554
P11172	Uridine 5'-monophosphate synthase OS=Homo sapiens OX=9606 GN=UMPS PE=1 SV=1	92.54	122.05	1.32	0.04556
Q86TX2	Acyl-coenzyme A thioesterase 1 OS=Homo sapiens OX=9606 GN=ACOT1 PE=1 SV=1	1185.78	1561.84	1.32	0.04742
Q8NEZ5	F-box only protein 22 OS=Homo sapiens OX=9606 GN=FBXO22 PE=1 SV=1	29.24	38.81	1.33	0.04765
Q13304	Uracil nucleotide/cysteinyl leukotriene receptor OS=Homo sapiens OX=9606 GN=GPR17 PE=2 SV=2	8.60	15.11	1.76	0.04815
Q9H074	Polyadenylate-binding protein-interacting protein 1 OS=Homo sapiens OX=9606 GN=PAIP1 PE=1 SV=1	362.63	470.83	1.30	0.04875

AD, Alzheimer's disease; FC, fold-change (FC>1.2 included); NC, cognitively normal controls; *P*<0.05

Supplementary Table 8. Extended mass spectrometry data on significantly up-regulated proteins in the hippocampus of Alzheimer's patients compared to cognitively normal controls.

**Accession**	**Description**	**AD (Means)**	**NC (Means)**	**FC**	***P***
Q9HBT6	Cadherin-20 OS=Homo sapiens OX=9606 GN=CDH20 PE=2 SV=2	222.82	182.75	1.22	7.834E-05
P35414	Apelin receptor OS=Homo sapiens OX=9606 GN=APLNR PE=1 SV=1	320.10	225.15	1.42	0.00010
Q7L266	Isoaspartyl peptidase/L-asparaginase OS=Homo sapiens OX=9606 GN=ASRGL1 PE=1 SV=2	1035.82	842.48	1.23	0.00028
Q9H4F8	Isoform 2 of SPARC-related modular calcium-binding protein 1 OS=Homo sapiens OX=9606 GN=SMOC1	151.10	118.73	1.27	0.00068
P04908	Histone H2A type 1-B/E OS=Homo sapiens OX=9606 GN=HIST1H2AB PE=1 SV=2	719.93	448.08	1.61	0.00121
P02795	Metallothionein-2 OS=Homo sapiens OX=9606 GN=MT2A PE=1 SV=1	839.80	644.50	1.30	0.00128
O14939	Phospholipase D2 OS=Homo sapiens OX=9606 GN=PLD2 PE=1 SV=2	55.18	38.29	1.44	0.00137
Q9BWL3	Uncharacterized protein C1orf43 OS=Homo sapiens OX=9606 GN=C1orf43 PE=2 SV=1	43.42	34.07	1.27	0.00146
P10636	Isoform Tau-B of Microtubule-associated protein tau OS=Homo sapiens OX=9606 GN=MAPT	1754.58	1019.13	1.72	0.00152
Q7Z2Z2	Elongation factor-like GTPase 1 OS=Homo sapiens OX=9606 GN=EFL1 PE=1 SV=2	66.41	52.01	1.28	0.00176
A6NCE7	Microtubule-associated proteins 1A/1B light chain 3 beta 2 OS=Homo sapiens OX=9606 GN=MAP1LC3B2 PE=2 SV=1	373.98	279.55	1.34	0.00189
Q504Y0	Zinc transporter ZIP12 OS=Homo sapiens OX=9606 GN=SLC39A12 PE=2 SV=3	53.32	43.24	1.23	0.00198
A5D8V7	Coiled-coil domain-containing protein 151 OS=Homo sapiens OX=9606 GN=CCDC151 PE=1 SV=1	31.35	14.65	2.14	0.00288
Q5T447	E3 ubiquitin-protein ligase HECTD3 OS=Homo sapiens OX=9606 GN=HECTD3 PE=1 SV=1	218.58	171.24	1.28	0.00375
A0A087WZ40	Ensconsin OS=Homo sapiens OX=9606 GN=MAP7 PE=1 SV=1	276.37	225.75	1.22	0.00385
Q6SZW1	Sterile alpha and TIR motif-containing protein 1 OS=Homo sapiens OX=9606 GN=SARM1 PE=1 SV=1	35.75	28.48	1.26	0.00400
P48058	Glutamate receptor 4 OS=Homo sapiens OX=9606 GN=GRIA4 PE=2 SV=2	172.33	141.53	1.22	0.00406
Q02127	Dihydroorotate dehydrogenase (quinone), mitochondrial OS=Homo sapiens OX=9606 GN=DHODH PE=1 SV=3	73.57	59.76	1.23	0.00447
Q5EBM0	UMP-CMP kinase 2, mitochondrial OS=Homo sapiens OX=9606 GN=CMPK2 PE=1 SV=3	90.69	71.83	1.26	0.00448
P05067	Amyloid-beta A4 protein OS=Homo sapiens OX=9606 GN=APP PE=1 SV=3	1830.21	1388.08	1.32	0.00469
Q9HBI6	Phylloquinone omega-hydroxylase CYP4F11 OS=Homo sapiens OX=9606 GN=CYP4F11 PE=1 SV=3	195.21	160.20	1.22	0.00481
Q7L311	Armadillo repeat-containing X-linked protein 2 OS=Homo sapiens OX=9606 GN=ARMCX2 PE=2 SV=1	28.40	20.81	1.36	0.00493

P61960	Ubiquitin-fold modifier 1 OS=Homo sapiens OX=9606 GN=UFM1 PE=1 SV=1	68.87	49.62	1.39	0.00614
Q9BUT1	3-hydroxybutyrate dehydrogenase type 2 OS=Homo sapiens OX=9606 GN=BDH2 PE=1 SV=2	1110.66	913.03	1.22	0.00770
Q96HD9	N-acyl-aromatic-L-amino acid amidohydrolase (carboxylate-forming) OS=Homo sapiens OX=9606 GN=ACY3 PE=1 SV=1	66.95	53.65	1.25	0.00790
P55283	Cadherin-4 OS=Homo sapiens OX=9606 GN=CDH4 PE=2 SV=2	31.41	23.96	1.31	0.00855
Q9HCS7	Pre-mRNA-splicing factor SYF1 OS=Homo sapiens OX=9606 GN=XAB2 PE=1 SV=2	20.12	15.26	1.32	0.00880
P82914	28S ribosomal protein S15, mitochondrial OS=Homo sapiens OX=9606 GN=MRPS15 PE=1 SV=1	141.06	116.02	1.22	0.00904
O00625	Pirin OS=Homo sapiens OX=9606 GN=PIR PE=1 SV=1	139.12	96.97	1.43	0.00933
Q86SK9	Stearoyl-CoA desaturase 5 OS=Homo sapiens OX=9606 GN=SCD5 PE=1 SV=2	114.32	95.01	1.20	0.00974
A0A087WY08	Thrombospondin type-1 domain-containing protein 7A OS=Homo sapiens OX=9606 GN=THSD7A PE=1 SV=1	49.29	37.98	1.30	0.00975
Q9H4G0	Isoform 2 of Band 4.1-like protein 1 OS=Homo sapiens OX=9606 GN=EPB41L1	186.32	139.24	1.34	0.01174
Q9UJ14	Glutathione hydrolase 7 OS=Homo sapiens OX=9606 GN=GGT7 PE=1 SV=2	223.98	178.08	1.26	0.01183
Q56VL3	OCIA domain-containing protein 2 OS=Homo sapiens OX=9606 GN=OCIAD2 PE=1 SV=1	145.16	104.60	1.39	0.01260
E9PLM6	Midkine OS=Homo sapiens OX=9606 GN=MDK PE=1 SV=1	183.42	107.87	1.70	0.01307
Q13905	Isoform 3 of Rap guanine nucleotide exchange factor 1 OS=Homo sapiens OX=9606 GN=RAPGEF1	86.93	71.75	1.21	0.01348
Q7Z7K0	COX assembly mitochondrial protein homolog OS=Homo sapiens OX=9606 GN=CMC1 PE=1 SV=1	40.58	25.95	1.56	0.01349
Q6S8J3	POTE ankyrin domain family member E OS=Homo sapiens OX=9606 GN=POTEE PE=2 SV=3	44.09	34.79	1.27	0.01468
Q14657	EKC/KEOPS complex subunit LAGE3 OS=Homo sapiens OX=9606 GN=LAGE3 PE=1 SV=2	9.29	4.75	1.96	0.01549
B1AKJ5	Nardilysin (N-arginine dibasic convertase), isoform CRA_d OS=Homo sapiens OX=9606 GN=NRDC PE=1 SV=1	15.06	11.16	1.35	0.01617
E7EQY1	Protein FAM136A OS=Homo sapiens OX=9606 GN=FAM136A PE=1 SV=1	148.60	123.10	1.21	0.01757
A0A2R8YFG2	Nicotinamide-nucleotide adenylyltransferase OS=Homo sapiens OX=9606 GN=NMNAT3 PE=1 SV=1	145.78	117.03	1.25	0.01787
Q13642	Isoform 4 of Four and a half LIM domains protein 1 OS=Homo sapiens OX=9606 GN=FHL1	1744.47	1336.11	1.31	0.01813
P46100	Transcriptional regulator ATRX OS=Homo sapiens OX=9606 GN=ATRX PE=1 SV=5	26.16	20.26	1.29	0.01964
Q9BPY8	Isoform 3 of Homeodomain-only protein OS=Homo sapiens OX=9606 GN=HOPX	95.27	77.20	1.23	0.02008
Q92982	Ninjurin-1 OS=Homo sapiens OX=9606 GN=NINJ1 PE=1 SV=2	66.20	43.84	1.51	0.02175
Q3B8N2	Galectin-9B OS=Homo sapiens OX=9606 GN=LGALS9B PE=1 SV=3	152.59	120.51	1.27	0.02352
A0A0G2JH68	Protein diaphanous homolog 1 OS=Homo sapiens OX=9606 GN=DIAPH1 PE=1 SV=1	55.73	43.74	1.27	0.02624

Q8N339	Metallothionein-1M OS=Homo sapiens OX=9606 GN=MT1M PE=3 SV=2	22.46	13.67	1.64	0.02734
Q8NHP8	Putative phospholipase B-like 2 OS=Homo sapiens OX=9606 GN=PLBD2 PE=1 SV=2	117.64	96.94	1.21	0.02778
P04733	Metallothionein-1F OS=Homo sapiens OX=9606 GN=MT1F PE=1 SV=1	356.64	269.30	1.32	0.02931
Q8NF37	Lysophosphatidylcholine acyltransferase 1 OS=Homo sapiens OX=9606 GN=LPCAT1 PE=1 SV=2	70.92	42.18	1.68	0.03070
Q14956	Transmembrane glycoprotein NMB OS=Homo sapiens OX=9606 GN=GPNMB PE=1 SV=2	182.47	136.61	1.34	0.03151
P31431	Syndecan-4 OS=Homo sapiens OX=9606 GN=SDC4 PE=1 SV=2	53.32	43.88	1.22	0.03279
H3BM91	COMM domain-containing protein 4 (Fragment) OS=Homo sapiens OX=9606 GN=COMMD4 PE=1 SV=1	33.88	27.22	1.24	0.03327
Q14005	Pro-interleukin-16 OS=Homo sapiens OX=9606 GN=IL16 PE=1 SV=4	271.98	188.05	1.45	0.03369
Q13255	Metabotropic glutamate receptor 1 OS=Homo sapiens OX=9606 GN=GRM1 PE=1 SV=3	223.76	184.32	1.21	0.03552
A0A1X7SBR3	Glial fibrillary acidic protein OS=Homo sapiens OX=9606 GN=GFAP PE=1 SV=1	174675.95	131497.94	1.33	0.03597
P98179	RNA-binding protein 3 OS=Homo sapiens OX=9606 GN=RBM3 PE=1 SV=1	67.72	47.17	1.44	0.03905
Q9NRX2	39S ribosomal protein L17, mitochondrial OS=Homo sapiens OX=9606 GN=MRPL17 PE=1 SV=1	48.53	39.53	1.23	0.04165
P34981	Thyrotropin-releasing hormone receptor OS=Homo sapiens OX=9606 GN=TRHR PE=1 SV=1	11.39	6.25	1.82	0.04225
O95470	Sphingosine-1-phosphate lyase 1 OS=Homo sapiens OX=9606 GN=SGPL1 PE=1 SV=3	54.38	43.88	1.24	0.04264
A8K727	Pleckstrin homology domain-containing family A member 2 OS=Homo sapiens OX=9606 GN=PLEKHA2 PE=1 SV=1	64.87	53.00	1.22	0.04265
Q14789	Isoform 2 of Golgin subfamily B member 1 OS=Homo sapiens OX=9606 GN=GOLGB1	22.82	15.76	1.45	0.04303
Q9UL42	Paraneoplastic antigen Ma2 OS=Homo sapiens OX=9606 GN=PNMA2 PE=1 SV=2	46.59	33.02	1.41	0.04338
Q9Y6N7	Isoform 2 of Roundabout homolog 1 OS=Homo sapiens OX=9606 GN=ROBO1	248.50	206.04	1.21	0.04568
Q9H3G5	Probable serine carboxypeptidase CPVL OS=Homo sapiens OX=9606 GN=CPVL PE=1 SV=2	120.54	88.45	1.36	0.04755
Q8IXM3	39S ribosomal protein L41, mitochondrial OS=Homo sapiens OX=9606 GN=MRPL41 PE=1 SV=1	45.67	34.79	1.31	0.04776
Q9BWH6	RNA polymerase II-associated protein 1 OS=Homo sapiens OX=9606 GN=RPAP1 PE=1 SV=3	43.05	34.16	1.26	0.04831
Q8N468	Major facilitator superfamily domain-containing protein 4A OS=Homo sapiens OX=9606 GN=MFSD4A PE=2 SV=3	37.21	30.08	1.24	0.04904
Q8IXT5	RNA-binding protein 12B OS=Homo sapiens OX=9606 GN=RBM12B PE=1 SV=2	115.27	86.40	1.33	0.04929
Q9NZR2	Low-density lipoprotein receptor-related protein 1B OS=Homo sapiens OX=9606 GN=LRP1B PE=1 SV=2	69.69	54.32	1.28	0.04967

AD, Alzheimer's disease; FC, fold-change (FC>1.2 included); NC, cognitively normal controls; P<0.05

Supplementary Table 9. Extended mass spectrometry data on significantly down-regulated proteins in the hippocampus of Alzheimer's patients compared to cognitively normal controls.

Accession	Description	AD (Means)	NC (Means)	FC (*-1)	*P*
A0A087WX97	Bcl-2-like protein 13 OS=Homo sapiens OX=9606 GN=BCL2L13 PE=1 SV=1	202.70	267.92	1.32	0.00022
P01009	Alpha-1-antitrypsin OS=Homo sapiens OX=9606 GN=SERPINA1 PE=1 SV=3	305.17	447.14	1.47	0.00145
O15069	NAC-alpha domain-containing protein 1 OS=Homo sapiens OX=9606 GN=NACAD PE=1 SV=3	73.60	99.55	1.35	0.00214
O60216	Double-strand-break repair protein rad21 homolog OS=Homo sapiens OX=9606 GN=RAD21 PE=1 SV=2	26.78	34.90	1.30	0.00803
P04921	Glycophorin-C OS=Homo sapiens OX=9606 GN=GYPC PE=1 SV=1	7.88	13.01	1.65	0.00844
O75508	Claudin-11 OS=Homo sapiens OX=9606 GN=CLDN11 PE=1 SV=2	1483.96	2165.23	1.46	0.01040
P13645	Keratin, type I cytoskeletal 10 OS=Homo sapiens OX=9606 GN=KRT10 PE=1 SV=6	2539.06	4513.88	1.78	0.01402
Q13595	Transformer-2 protein homolog alpha OS=Homo sapiens OX=9606 GN=TRA2A PE=1 SV=1	115.71	147.30	1.27	0.01623
P59768	Guanine nucleotide-binding protein G(I)/G(S)/G(O) subunit gamma-2 OS=Homo sapiens OX=9606 GN=GNG2 PE=1 SV=2	22.99	29.51	1.28	0.01759
P35908	Keratin, type II cytoskeletal 2 epidermal OS=Homo sapiens OX=9606 GN=KRT2 PE=1 SV=2	1529.15	2592.87	1.70	0.02018
A0A0J9YY99	Uncharacterized protein (Fragment) OS=Homo sapiens OX=9606 PE=1 SV=1	65.44	118.69	1.81	0.02178
Q8NHP6	Motile sperm domain-containing protein 2 OS=Homo sapiens OX=9606 GN=MOSPD2 PE=1 SV=1	160.84	202.93	1.26	0.02669
P02533	Keratin, type I cytoskeletal 14 OS=Homo sapiens OX=9606 GN=KRT14 PE=1 SV=4	397.86	710.05	1.78	0.02903
P13647	Keratin, type II cytoskeletal 5 OS=Homo sapiens OX=9606 GN=KRT5 PE=1 SV=3	536.14	855.82	1.60	0.03219
P02671	Fibrinogen alpha chain OS=Homo sapiens OX=9606 GN=FGA PE=1 SV=2	2809.87	3929.11	1.40	0.03320
P04264	Keratin, type II cytoskeletal 1 OS=Homo sapiens OX=9606 GN=KRT1 PE=1 SV=6	7426.96	13057.02	1.76	0.03422
P01780	Immunoglobulin heavy variable 3-7 OS=Homo sapiens OX=9606 GN=IGHV3-7 PE=1 SV=2	34.45	52.87	1.53	0.03453
Q9UF11	Isoform 2 of Pleckstrin homology domain-containing family B member 1 OS=Homo sapiens OX=9606 GN=PLEKHB1	511.09	642.93	1.26	0.03473
J3KQ48	Peptidyl-tRNA hydrolase 2, mitochondrial OS=Homo sapiens OX=9606 GN=PTRH2 PE=1 SV=1	15.74	20.97	1.33	0.03484
Q96DZ9	CKLF-like MARVEL transmembrane domain-containing protein 5 OS=Homo sapiens OX=9606 GN=CMTM5 PE=1 SV=2	244.82	307.59	1.26	0.03510
P14923	Junction plakoglobin OS=Homo sapiens OX=9606 GN=JUP PE=1 SV=3	73.89	98.42	1.33	0.04179
P02675	Fibrinogen beta chain OS=Homo sapiens OX=9606 GN=FGB PE=1 SV=2	1249.34	1699.98	1.36	0.04360
Q96A33	Coiled-coil domain-containing protein 47 OS=Homo sapiens OX=9606 GN=CCDC47 PE=1 SV=1	240.72	305.31	1.27	0.04513
Q8N1N4	Keratin, type II cytoskeletal 78 OS=Homo sapiens OX=9606 GN=KRT78 PE=1 SV=2	66.42	98.91	1.49	0.04869

AD, Alzheimer's disease; FC, fold-change (FC>1.2 included); NC, cognitively normal controls; $P<0.05$

Supplementary Table 10. Extended mass spectrometry data on significantly up-regulated proteins in the cerebellum of Alzheimer's patients compared to cognitively normal controls.

Accession	Description	AD (Means)	NC (Means)	FC	*P*
P02792	Ferritin light chain OS=Homo sapiens OX=9606 GN=FTL PE=1 SV=2	3676.15	2824.18	1.30	0.00036
Q01484	Isoform 2 of Ankyrin-2 OS=Homo sapiens OX=9606 GN=ANK2	90.25	74.93	1.20	0.00063
H3BNK4	Protein NDRG4 (Fragment) OS=Homo sapiens OX=9606 GN=NDRG4 PE=1 SV=8	138.98	111.94	1.24	0.00079
Q8WWI1	LIM domain only protein 7 OS=Homo sapiens OX=9606 GN=LMO7 PE=1 SV=3	117.66	97.99	1.20	0.00090
P15531	Isoform 2 of Nucleoside diphosphate kinase A OS=Homo sapiens OX=9606 GN=NME1	141.43	103.32	1.37	0.00200
Q6ZTA4	Tripartite motif-containing protein 67 OS=Homo sapiens OX=9606 GN=TRIM67 PE=2 SV=3	44.19	32.24	1.37	0.00249
Q9Y2T3	Isoform 3 of Guanine deaminase OS=Homo sapiens OX=9606 GN=GDA	7.74	4.38	1.77	0.00274
P45984	Isoform Alpha-1 of Mitogen-activated protein kinase 9 OS=Homo sapiens OX=9606 GN=MAPK9	94.83	77.16	1.23	0.00302
A8MVW0	Protein FAM171A2 OS=Homo sapiens OX=9606 GN=FAM171A2 PE=1 SV=1	301.93	249.63	1.21	0.00330
Q9BWJ5	Splicing factor 3B subunit 5 OS=Homo sapiens OX=9606 GN=SF3B5 PE=1 SV=1	116.41	95.75	1.22	0.00331
P18084	Integrin beta-5 OS=Homo sapiens OX=9606 GN=ITGB5 PE=1 SV=1	252.02	180.03	1.40	0.00383
Q8NBL3	Transmembrane protein 178A OS=Homo sapiens OX=9606 GN=TMEM178A PE=2 SV=1	43.34	31.51	1.38	0.00438
P17677	Isoform 2 of Neuromodulin OS=Homo sapiens OX=9606 GN=GAP43	6307.28	4933.66	1.28	0.00445
Q53TN4	Cytochrome b reductase 1 OS=Homo sapiens OX=9606 GN=CYBRD1 PE=1 SV=1	90.34	73.18	1.23	0.00480
E9PGV9	ATP-binding cassette sub-family G member 1 OS=Homo sapiens OX=9606 GN=ABCG1 PE=1 SV=1	179.00	135.22	1.32	0.00492
Q96PK6	Isoform 5 of RNA-binding protein 14 OS=Homo sapiens OX=9606 GN=RBM14	81.36	60.28	1.35	0.00529
Q9NY47	Voltage-dependent calcium channel subunit alpha-2/delta-2 OS=Homo sapiens OX=9606 GN=CACNA2D2 PE=1 SV=2	1742.32	1441.09	1.21	0.00552
Q10589	Bone marrow stromal antigen 2 OS=Homo sapiens OX=9606 GN=BST2 PE=1 SV=1	59.92	43.72	1.37	0.00663
Q3ZAQ7	Isoform 2 of Vacuolar ATPase assembly integral membrane protein VMA21 OS=Homo sapiens OX=9606 GN=VMA21	153.03	118.64	1.29	0.00751
Q96PP8	Guanylate-binding protein 5 OS=Homo sapiens OX=9606 GN=GBP5 PE=1 SV=1	214.69	160.06	1.34	0.00793
O00625	Pirin OS=Homo sapiens OX=9606 GN=PIR PE=1 SV=1	76.75	63.22	1.21	0.00805
O75914	Isoform 3 of Serine/threonine-protein kinase PAK 3 OS=Homo sapiens OX=9606 GN=PAK3	97.51	76.28	1.28	0.00940
F6UJY1	Putative hydroxypyruvate isomerase OS=Homo sapiens OX=9606 GN=HYI PE=1 SV=1	312.77	257.82	1.21	0.01084
Q9NWQ8	Phosphoprotein associated with glycosphingolipid-enriched microdomains 1 OS=Homo sapiens OX=9606 GN=PAG1 PE=1 SV=2	378.32	302.24	1.25	0.01190

Q9Y3B9	RRP15-like protein OS=Homo sapiens OX=9606 GN=RRP15 PE=1 SV=2	26.65	18.63	1.43	0.01263
A0A0A0MTC1	E3 ubiquitin-protein ligase RNF213 OS=Homo sapiens OX=9606 GN=RNF213 PE=1 SV=1	52.05	42.57	1.22	0.01334
Q9P121	Isoform 4 of Neurotrimin OS=Homo sapiens OX=9606 GN=NTM	2674.45	2196.95	1.22	0.01343
Q9BY67	Isoform 3 of Cell adhesion molecule 1 OS=Homo sapiens OX=9606 GN=CADM1	1215.62	935.75	1.30	0.01395
Q8WWM9	Cytoglobin OS=Homo sapiens OX=9606 GN=CYGB PE=1 SV=1	30.13	19.61	1.54	0.01637
Q9P0R6	GSK3B-interacting protein OS=Homo sapiens OX=9606 GN=GSKIP PE=1 SV=2	61.61	50.68	1.22	0.01666
F6RH32	Voltage-dependent N-type calcium channel subunit alpha OS=Homo sapiens OX=9606 GN=CACNA1B PE=1 SV=1	267.87	216.75	1.24	0.01781
Q9H1U4	Multiple epidermal growth factor-like domains protein 9 OS=Homo sapiens OX=9606 GN=MEGF9 PE=2 SV=3	153.67	112.63	1.36	0.01814
Q7Z7G2	Complexin-4 OS=Homo sapiens OX=9606 GN=CPLX4 PE=2 SV=1	185.54	149.72	1.24	0.01890
K7EKU3	FXYD domain-containing ion transport regulator OS=Homo sapiens OX=9606 GN=FXYD7 PE=1 SV=1	43.05	34.81	1.24	0.01893
P42658	Isoform DPPX-S of Dipeptidyl aminopeptidase-like protein 6 OS=Homo sapiens OX=9606 GN=DPP6	104.13	82.08	1.27	0.01969
P80723	Brain acid soluble protein 1 OS=Homo sapiens OX=9606 GN=BASP1 PE=1 SV=2	9285.55	7005.42	1.33	0.02061
Q05519	Serine/arginine-rich splicing factor 11 OS=Homo sapiens OX=9606 GN=SRSF11 PE=1 SV=1	338.70	265.47	1.28	0.02113
Q86X02	Cerebellar degeneration-related protein 2-like OS=Homo sapiens OX=9606 GN=CDR2L PE=1 SV=2	258.16	213.60	1.21	0.02225
O14492	Isoform 2 of SH2B adapter protein 2 OS=Homo sapiens OX=9606 GN=SH2B2	29.74	18.78	1.58	0.02301
A0A0A0MTH9	TATA-binding protein-associated factor 172 OS=Homo sapiens OX=9606 GN=BTAF1 PE=1 SV=1	109.90	85.57	1.28	0.02402
Q8NC67	Neuropilin and tolloid-like protein 2 OS=Homo sapiens OX=9606 GN=NETO2 PE=1 SV=1	22.93	16.86	1.36	0.02529
K7EQG4	FXYD domain-containing ion transport regulator (Fragment) OS=Homo sapiens OX=9606 GN=FXYD1 PE=1 SV=2	230.84	189.31	1.22	0.02606
S4R347	Formin-binding protein 1-like OS=Homo sapiens OX=9606 GN=FNBP1L PE=1 SV=1	113.32	93.44	1.21	0.02761
P13521	Secretogranin-2 OS=Homo sapiens OX=9606 GN=SCG2 PE=1 SV=2	985.30	773.16	1.27	0.02806
Q9Y4F5	Isoform 2 of Centrosomal protein of 170 kDa protein B OS=Homo sapiens OX=9606 GN=CEP170B	61.56	48.52	1.27	0.02817
Q9Y333	U6 snRNA-associated Sm-like protein LSm2 OS=Homo sapiens OX=9606 GN=LSM2 PE=1 SV=1	34.10	27.60	1.24	0.02830
Q8WU79	Stromal membrane-associated protein 2 OS=Homo sapiens OX=9606 GN=SMAP2 PE=1 SV=1	155.25	125.60	1.24	0.02945
Q6ZVL6	UPF0606 protein KIAA1549L OS=Homo sapiens OX=9606 GN=KIAA1549L PE=2 SV=2	167.56	126.82	1.32	0.02972
Q96DF8	Splicing factor ESS-2 homolog OS=Homo sapiens OX=9606 GN=ESS2 PE=1 SV=1	94.25	69.56	1.35	0.03000

Q9H7P6	Multivesicular body subunit 12B OS=Homo sapiens OX=9606 GN=MVB12B PE=1 SV=2	58.99	48.09	1.23	0.03048
P56945	Isoform 6 of Breast cancer anti-estrogen resistance protein 1 OS=Homo sapiens OX=9606 GN=BCAR1	429.04	355.86	1.21	0.03057
P07998	Ribonuclease pancreatic OS=Homo sapiens OX=9606 GN=RNASE1 PE=1 SV=4	7.02	4.44	1.58	0.03082
O43597	Protein sprouty homolog 2 OS=Homo sapiens OX=9606 GN=SPRY2 PE=1 SV=1	44.77	36.53	1.23	0.03087
Q6DN14	Multiple C2 and transmembrane domain-containing protein 1 OS=Homo sapiens OX=9606 GN=MCTP1 PE=2 SV=2	338.52	277.37	1.22	0.03239
B1AL88	Transmembrane protein FAM155A OS=Homo sapiens OX=9606 GN=FAM155A PE=2 SV=1	18.77	11.98	1.57	0.03266
O15357	Phosphatidylinositol 3,4,5-trisphosphate 5-phosphatase 2 OS=Homo sapiens OX=9606 GN=INPPL1 PE=1 SV=2	60.38	47.74	1.26	0.03302
Q9H4X1	Regulator of cell cycle RGCC OS=Homo sapiens OX=9606 GN=RGCC PE=1 SV=1	31.37	22.20	1.41	0.03371
Q15560	Transcription elongation factor A protein 2 OS=Homo sapiens OX=9606 GN=TCEA2 PE=1 SV=1	59.24	47.00	1.26	0.03427
Q13595	Transformer-2 protein homolog alpha OS=Homo sapiens OX=9606 GN=TRA2A PE=1 SV=1	506.46	415.74	1.22	0.03498
O15069	NAC-alpha domain-containing protein 1 OS=Homo sapiens OX=9606 GN=NACAD PE=1 SV=3	45.99	36.21	1.27	0.03515
M0QYM7	Tubulin beta-4A chain (Fragment) OS=Homo sapiens OX=9606 GN=TUBB4A PE=1 SV=8	67.22	52.02	1.29	0.03602
Q16849	Receptor-type tyrosine-protein phosphatase-like N OS=Homo sapiens OX=9606 GN=PTPRN PE=1 SV=1	57.29	47.31	1.21	0.03790
P52824	Diacylglycerol kinase theta OS=Homo sapiens OX=9606 GN=DGKQ PE=1 SV=2	19.68	15.54	1.27	0.03881
Q9P266	Junctional protein associated with coronary artery disease OS=Homo sapiens OX=9606 GN=JCAD PE=1 SV=3	26.17	18.62	1.41	0.04153
Q9BZJ0	Crooked neck-like protein 1 OS=Homo sapiens OX=9606 GN=CRNKL1 PE=1 SV=4	54.50	40.14	1.36	0.04167
P02538	Keratin, type II cytoskeletal 6A OS=Homo sapiens OX=9606 GN=KRT6A PE=1 SV=3	74.91	39.21	1.91	0.04254
O75391	Sperm-associated antigen 7 OS=Homo sapiens OX=9606 GN=SPAG7 PE=1 SV=2	81.55	52.93	1.54	0.04353
Q5EBL4	RILP-like protein 1 OS=Homo sapiens OX=9606 GN=RILPL1 PE=1 SV=1	112.30	91.73	1.22	0.04382
P82914	28S ribosomal protein S15, mitochondrial OS=Homo sapiens OX=9606 GN=MRPS15 PE=1 SV=1	278.90	214.57	1.30	0.04509
Q8TB61	Adenosine 3'-phospho 5'-phosphosulfate transporter 1 OS=Homo sapiens OX=9606 GN=SLC35B2 PE=1 SV=1	30.22	24.06	1.26	0.04595
Q8NI22	Multiple coagulation factor deficiency protein 2 OS=Homo sapiens OX=9606 GN=MCFD2 PE=1 SV=1	59.54	34.99	1.70	0.04720
Q5T8D3	Isoform 3 of Acyl-CoA-binding domain-containing protein 5 OS=Homo sapiens OX=9606 GN=ACBD5	194.83	160.83	1.21	0.04759
Q2NKQ1	Small G protein signaling modulator 1 OS=Homo sapiens OX=9606 GN=SGSM1 PE=1 SV=2	169.53	136.66	1.24	0.04935

AD, Alzheimer's disease; FC, fold-change (FC>1.2 included); NC, cognitively normal controls; *P*<0.05

Supplementary Table 11. Extended mass spectrometry data on significantly down-regulated proteins in the cerebellum of Alzheimer's patients compared to cognitively normal controls.

Accession	Description	AD (Means)	NC (Means)	FC (*-1)	*P*
A0A2R8YGD3	Rap guanine nucleotide exchange factor 2 OS=Homo sapiens OX=9606 GN=RAPGEF2 PE=1 SV=1	256.60	371.95	1.45	0.00042
P10643	Complement component C7 OS=Homo sapiens OX=9606 GN=C7 PE=1 SV=2	71.40	101.74	1.42	0.00249
P11908	Isoform 2 of Ribose-phosphate pyrophosphokinase 2 OS=Homo sapiens OX=9606 GN=PRPS2	31.71	40.25	1.27	0.00387
A0A3B3ITJ4	Heterogeneous nuclear ribonucleoprotein L (Fragment) OS=Homo sapiens OX=9606 GN=HNRNPL PE=1 SV=1	50.33	63.23	1.26	0.00534
Q92903	Phosphatidate cytidylyltransferase 1 OS=Homo sapiens OX=9606 GN=CDS1 PE=2 SV=2	55.24	69.13	1.25	0.00728
A0A2R8Y7X9	GLOBIN domain-containing protein OS=Homo sapiens OX=9606 PE=3 SV=1	395.56	742.14	1.88	0.00782
Q9BS92	Protein NipSnap homolog 3B OS=Homo sapiens OX=9606 GN=NIPSNAP3B PE=2 SV=1	184.30	299.11	1.62	0.01036
Q01814	Isoform WA of Plasma membrane calcium-transporting ATPase 2 OS=Homo sapiens OX=9606 GN=ATP2B2	149.15	192.35	1.29	0.01163
A0A0G2JPD3	HLA class I histocompatibility antigen, A-3 alpha chain OS=Homo sapiens OX=9606 GN=HLA-A PE=1 SV=1	15.82	34.98	2.21	0.01184
Q8WVD5	RING finger protein 141 OS=Homo sapiens OX=9606 GN=RNF141 PE=1 SV=1	355.75	444.89	1.25	0.01409
Q86VN1	Vacuolar protein-sorting-associated protein 36 OS=Homo sapiens OX=9606 GN=VPS36 PE=1 SV=1	66.12	87.49	1.32	0.01713
H3BP13	Trafficking protein particle complex subunit 2-like protein OS=Homo sapiens OX=9606 GN=TRAPPC2L PE=1 SV=1	40.44	51.42	1.27	0.02071
K7EQD6	Vesicle-fusing ATPase (Fragment) OS=Homo sapiens OX=9606 GN=NSF PE=1 SV=1	157.55	207.26	1.32	0.02911
P02679	Fibrinogen gamma chain OS=Homo sapiens OX=9606 GN=FGG PE=1 SV=3	1622.19	2447.99	1.51	0.03295
S4R3E9	NEDD8-MDP1 readthrough OS=Homo sapiens OX=9606 GN=NEDD8-MDP1 PE=4 SV=1	126.13	234.11	1.86	0.03462
A0A0A0MRL7	Caspase-7 OS=Homo sapiens OX=9606 GN=CASP7 PE=1 SV=1	23.66	34.42	1.45	0.03540
Q14165	Malectin OS=Homo sapiens OX=9606 GN=MLEC PE=1 SV=1	56.43	75.61	1.34	0.03599
Q9Y4A5	Transformation/transcription domain-associated protein OS=Homo sapiens OX=9606 GN=TRRAP PE=1 SV=3	26.60	34.09	1.28	0.03718
Q7Z7A4	PX domain-containing protein kinase-like protein OS=Homo sapiens OX=9606 GN=PXK PE=1 SV=1	15.09	25.69	1.70	0.04245
A0A1W2PPJ5	Protein cereblon OS=Homo sapiens OX=9606 GN=CRBN PE=1 SV=1	217.62	292.61	1.34	0.04359
P60903	Protein S100-A10 OS=Homo sapiens OX=9606 GN=S100A10 PE=1 SV=2	232.01	318.79	1.37	0.04415
A0A087WZK0	Deoxyhypusine synthase OS=Homo sapiens OX=9606 GN=DHPS PE=1 SV=1	19.34	24.75	1.28	0.04533

AD, Alzheimer's disease; FC, fold-change (FC>1.2 included); NC, cognitively normal controls; $P<0.05$

Supplementary Table 12. Common upregulated DEPs with previous brain datasets.

	Gene Name	Dataset.1	Dataset.2	Dataset.3	Dataset.4	Dataset.5	Dataset.6	Dataset.7
**1**	LSP1	Up	Up	Up	Up	Up	Up	Up
**2**	BLVRB	Up	Up	Up	Up	Up	Up	Up
**3**	HSPB8	Up	Up	Up	Up	Up	Up	Up
**4**	HSPB1	Up	Up	Up	Up	Up	Up	Up
**5**	LZIC	No change	Up	Up	Up	Up	Up	Up
**6**	RSU1	Up	No change	Up	Up	Up	Up	Up
**7**	PAFAH1B3	Up	Up	Up	No change	Up	Up	Up
**8**	GCLM	No change	Up	Up	Up	Up	Up	Up
**9**	FBP1	Up	Up	Up	Up	Up	Up	No change
**10**	FABP7	Up	Up	Up	Up	Up	Up	No change
**11**	BDH2	Up	Up	Up	Up	Up	Up	No change
**12**	BAG3	Up	No change	Up	Up	Up	Up	Up
**13**	S100A1	Up	Up	Up	No change	Up	Up	No change
**14**	ABHD14B	Up	Up	Up	Up	No change	No change	Up
**15**	HEBP2	Up	Up	Up	No change	No change	Up	Up
**16**	FABP5	No change	Up	Up	Up	Up	Up	No change
**17**	MAP2K6	Up	Up	Up	No change	No change	Up	Up
**18**	CMBL	Up	No change	Up	Up	Up	Up	No change
**19**	PSMD9	No change	Up	Up	Up	Up	Up	No change
**20**	PTRHD1	Up	No change	Up	No change	Up	Up	Up
**21**	LAMA1	Up	Up	Up	Up	No change	Up	No change
**22**	ALDH9A1	No change	No change	Up	Up	Up	Up	No change
**23**	ANKRD45	Up	Up	NA	Up	NA	Up	NA
**24**	THTPA	No change	No change	Up	No change	Up	Up	Up
**25**	TNS3	No change	No change	Up	Up	Up	Up	No change
**26**	TAX1BP3	No change	No change	Up	Up	Up	Up	No change

**27**	SWAP70	No change	Up	Up	No change	Up	Up	No change
**28**	SH3BGRL	Up	No change	Up	No change	Up	No change	Up
**29**	S100B	No change	No change	Up	Up	Up	Up	No change
**30**	FNTA	No change	Up	Up	Up	Up	No change	No change
**31**	TOMM34	No change	Up	Up	Up	No change	No change	Up
**32**	GSTM2	Up	No change	Up	No change	Up	Up	No change
**33**	GSN	No change	Up	NA	Up	Up	Up	No change
**34**	EIF4A1	Up	Up	Up	No change	No change	No change	Up
**35**	DFFA	No change	No change	Up	Up	Up	Up	No change
**36**	CARHSP1	No change	No change	Up	Up	Up	No change	Up
**37**	BOLA2	No change	Up	Up	Up	No change	No change	Up
**38**	BLOC1S4	Up	No change	Up	No change	NA	Up	Up
**39**	UBE2H	Up	No change	Up	Up	No change	No change	Up
**40**	RSRC1	No change	No change	Up	Up	NA	Up	Up
**41**	DAG1	Up	No change	Up	No change	Up	Up	No change
**42**	AKR1C1	Up	Up	Up	Up	NA	No change	No change
**43**	NSFL1C	No change	No change	No change	No change	Up	Up	Up
**44**	JMJD6	Up	Up	No change	No change	NA	No change	Up
**45**	GALK1	No change	No change	Up	Up	No change	Up	No change
**46**	PEA15	No change	No change	Up	No change	Up	Up	No change
**47**	TPD52L2	No change	Up	NA	No change	Up	No change	Up
**48**	GALE	Up	Up	Up	No change	No change	No change	No change
**49**	UBA6	No change	Up	Up	No change	No change	Up	No change
**50**	SOD1	No change	Up	Up	No change	Up	No change	No change
**51**	PRKD1	Up	No change	Up	No change	NA	Up	No change
**52**	RLBP1	No change	No change	Up	Up	No change	Up	No change
**53**	UBR7	No change	No change	No change	Up	No change	Up	Up
**54**	PARK7	Up	No change	Up	No change	No change	No change	Up

**55**	SUGT1	No change	Up	NA	No change	No change	Up	Up
**56**	SLC7A6OS	No change	No change	NA	Up	NA	Up	Up
**57**	PPCS	No change	Up	Up	No change	No change	Up	No change
**58**	NUDCD2	No change	No change	Up	No change	No change	Up	Up
**59**	ITGB2	No change	Up	NA	NA	Up	Up	No change
**60**	HBD	Up	Up	No change	No change	No change	Up	No change
**61**	LAGE3	Up	No change	No change	No change	No change	Up	Up
**62**	CRABP1	Up	No change	Up	Up	No change	No change	No change
**63**	ASS1	No change	No change	Up	Up	Up	No change	No change
**64**	AKR1C3	No change	Up	Up	Up	No change	No change	No change
**65**	AKR7A3	No change	No change	Up	No change	Up	Up	No change
**66**	PSMD10	No change	No change	Up	No change	Up	Up	No change
**67**	PHPT1	No change	No change	Up	No change	Up	Up	No change
**68**	C1orf198	No change	No change	Up	No change	Up	Up	No change
**69**	TERF2IP	No change	No change	Up	Up	No change	No change	Up
**70**	CD58	Up	NA	Up	No change	No change	Up	No change
**71**	FSTL1	Up	No change	Up	Up	NA	No change	NA
**72**	FBXO2	Up	No change	Up	No change	No change	Up	No change
**73**	DUSP23	No change	No change	Up	No change	Up	No change	Up
**74**	COLEC12	No change	Up	Up	Up	No change	No change	No change
**75**	CTSB	No change	Up	Up	Up	No change	No change	No change
**76**	AIF1	No change	Up	Up	Up	No change	No change	No change
**77**	SELENBP1	No change	No change	NA	No change	Up	Up	No change
**78**	VAV3	No change	No change	Up	Up	NA	No change	No change
**79**	SHTN1	No change	Up	Up	No change	NA	NA	No change
**80**	TYMP	No change	No change	Up	Up	No change	No change	No change
**81**	NAP1L4	No change	No change	Up	No change	No change	Up	No change
**82**	TPM1	No change	No change	NA	NA	No change	Up	Up

**83**	TPM3	No change	No change	NA	No change	Up	Up	No change
**84**	ITGAM	No change	No change	Up	No change	Up	No change	No change
**85**	ATG3	No change	No change	Up	No change	No change	Up	No change
**86**	SPR	No change	No change	No change	No change	No change	Up	Up
**87**	ARHGDIB	No change	No change	Up	Up	No change	No change	No change
**88**	PSME1	No change	No change	Up	No change	Up	No change	No change
**89**	PCNA	No change	No change	Up	No change	No change	Up	No change
**90**	PMVK	No change	No change	Up	No change	No change	Up	No change
**91**	IFT27	No change	No change	Up	No change	No change	Up	No change
**92**	HBB	Up	Up	No change	No change	No change	No change	NA
**93**	HBA1	Up	Up	No change	No change	No change	No change	NA
**94**	HEBP1	No change	Up	Up	No change	No change	No change	No change
**95**	HCLS1	No change	No change	Up	No change	Up	No change	No change
**96**	GCHFR	Up	No change	NA	Up	No change	No change	NA
**97**	GSTM3	No change	No change	Up	No change	No change	Up	No change
**98**	FRMD8	No change	No change	Up	No change	No change	No change	Up
**99**	FADD	Up	No change	Up	No change	No change	No change	No change
**100**	GSPT2	Up	No change	Up	No change	No change	No change	No change
**101**	DUSP12	Up	No change	Up	No change	NA	No change	No change
**102**	CCS	No change	No change	Up	No change	No change	No change	Up
**103**	CHKB	Up	No change	Up	No change	No change	No change	No change
**104**	CLIP1	No change	No change	Up	No change	Up	No change	NA
**105**	BROX	No change	No change	Up	Up	No change	No change	No change
**106**	AIF1L	No change	Up	Up	No change	No change	No change	No change
**107**	NUDT1	No change	No change	Up	Up	NA	No change	No change
**108**	TPM3	No change	No change	NA	No change	Up	Up	No change
**109**	SLC16A2	No change	No change	Up	No change	No change	No change	Up
**110**	MOB3B	No change	No change	Up	No change	No change	Up	No change

**111**	EFNB1	No change	No change	Up	No change	No change	Up	No change
**112**	CYP2U1	No change	No change	Up	No change	NA	Up	No change
**113**	COMMD8	No change	No change	Up	No change	No change	Up	No change
**114**	PCYT1B	Up	Up	No change	No change	No change	No change	No change
**115**	CDH4	No change	No change	Up	No change	No change	Up	No change
**116**	C4BPA	Up	Up	No change	No change	No change	No change	No change
**117**	AIFM2	Up	No change	Up	No change	NA	No change	No change
**118**	CAST	No change	No change	No change	No change	Up	No change	No change
**119**	TRAPPC2	Up	No change	No change	No change	No change	No change	No change
**120**	EIF4A2	No change	Up	No change	No change	No change	No change	No change
**121**	ERAP1	No change	No change	Up	No change	No change	No change	No change
**122**	ACSS2	No change	No change	No change	No change	No change	No change	Up
**123**	MEAF6	No change	No change	NA	Up	NA	No change	No change
**124**	TCEAL4	No change	No change	No change	No change	No change	No change	Up
**125**	UBE2L6	No change	No change	Up	No change	No change	No change	No change
**126**	USP5	No change	Up	No change	No change	No change	No change	No change
**127**	LSM8	No change	No change	No change	No change	No change	Up	No change
**128**	LSM7	No change	No change	No change	No change	No change	Up	No change
**129**	TXNL1	Up	No change	No change	No change	No change	No change	No change
**130**	SAE1	Up	No change	No change	No change	No change	No change	No change
**131**	SULT1A1	No change	No change	Up	No change	No change	No change	No change
**132**	SKP1	No change	No change	Up	No change	No change	No change	No change
**133**	SNX12	No change	No change	No change	No change	No change	No change	Up
**134**	SH3BGRL3	No change	No change	Up	No change	No change	No change	No change
**135**	STK4	No change	No change	Up	No change	No change	No change	No change
**136**	AKT2	No change	No change	Up	No change	No change	No change	NA
**137**	PDXK	No change	No change	No change	No change	No change	Up	No change
**138**	IMPACT	No change	No change	Up	No change	No change	No change	No change

**139**	PDCL3	No change	No change	No change	No change	No change	No change	Up
**140**	PPIL3	No change	No change	No change	No change	No change	Up	No change
**141**	MIEN1	No change	No change	No change	No change	No change	No change	Up
**142**	MIF4GD	No change	No change	Up	No change	No change	No change	No change
**143**	MDP1	No change	No change	Up	No change	No change	No change	No change
**144**	INPP1	No change	No change	No change	No change	No change	Up	No change
**145**	GLRX3	No change	No change	No change	No change	No change	No change	Up
**146**	GLRX	No change	Up	No change	No change	No change	No change	No change
**147**	GSDMD	No change	No change	Up	No change	No change	No change	No change
**148**	EIF3G	No change	No change	Up	No change	No change	No change	No change
**149**	EIF1AX	No change	No change	Up	No change	No change	No change	No change
**150**	CRADD	No change	No change	Up	No change	NA	No change	No change
**151**	CTPS1	No change	No change	No change	No change	No change	No change	Up
**152**	CA1	No change	Up	No change	No change	No change	No change	No change
**153**	BPGM	No change	Up	No change	No change	No change	No change	No change
**154**	ATIC	No change	No change	Up	No change	No change	No change	No change
**155**	EPHX2	No change	No change	Up	No change	No change	No change	No change
**156**	ATXN7L3B	No change	No change	No change	No change	No change	No change	Up
**157**	AHSA1	No change	No change	No change	No change	No change	No change	Up
**158**	TBCEL	No change	No change	Up	No change	No change	No change	No change
**159**	TPM4	No change	No change	Up	No change	No change	No change	No change
**160**	CTDP1	No change	No change	Up	No change	No change	No change	No change
**161**	HEXIM2	Up	No change	NA	No change	NA	No change	No change
**162**	CTSH	No change	No change	Up	No change	No change	No change	No change
**163**	PLLP	No change	No change	No change	No change	No change	No change	Up
**164**	PPL	No change	Up	No change	No change	No change	No change	No change
**165**	GCSH	Up	No change	No change	No change	No change	No change	No change
**166**	NCCRP1	Up	NA	NA	NA	NA	NA	NA

**167**	POLR2D	No change	No change	Up	No change	No change	No change	No change
**168**	DERL2	Up	NA	NA	NA	NA	NA	NA
**169**	CSTA	Up	No change	NA	No change	NA	No change	NA
**170**	CRTC1	No change	No change	No change	No change	No change	No change	Up
**171**	ARF6	Up	No change	No change	No change	No change	No change	No change

Ref*:* Bai, B., Vanderwall, D., Li, Y. *et al.* Proteomic landscape of Alzheimer's Disease
Mol Neurodegeneration 16, 55 (2021). https://doi.org/10.1186/s13024-021-00474-z
Dataset 1-3; Bai et al. Neuron 2020, 105:975-991:
Dataset 2: individual case TMT analysis of Banner Sun cohort
Dataset 3: individual case TMT analysis of Mount Sinai cohort
Banner Sun cohort: Human postmortem brain tissue samples
Mount Sinai cohort: frontal pole (https://www.nature.com/articles/sdata2018185)
Dataset 4: Wang et al. Anal. Chem. 2020, 92:7162-7170 (16-plex TMT analysis of Banner Sun cohort)
Datasets 5-6: Higginbotham et al. Sci. Adv. 2020, 6:eaaz9360 (dataset5: Table S2C; dataset6: Table S2F)
Dataset 7: Sathe et al. J. Neurochem. 2021, 156:988-1002
For the comparison of our retinal data against the literature brain cortex proteome data, proteins in each of the seven studies were regarded as DEP if the t-test p-value was less than 0.05, up or down regulated based on the log fold change AD/control (>0 up-regulated, <0 down-regulated).
Proteins p-values less than 0.05 were regarded as unchanged.

Supplementary Table 13. Common downregulated DEPs with previous brain datasets.

	Gene Name	*Dataset.1*	*Dataset.2*	*Dataset.3*	*Dataset.4*	*Dataset.5*	*Dataset.6*	*Dataset.7*
**1**	RTN4	Down	Down	Down	Down	Down	Down	Down
**2**	NDUFB3	Down	Down	Down	Down	Down	Down	Down
**3**	NDUFS1	No change	Down	Down	Down	Down	Down	Down
**4**	STXBP1	No change	Down	Down	Down	Down	Down	Down
**5**	AP2M1	No change	Down	Down	Down	Down	Down	Down
**6**	ADCY1	No change	Down	Down	Down	Down	Down	Down
**7**	SDHB	No change	Down	Down	Down	Down	Down	Down
**8**	SLC2A3	No change	Down	Down	Down	Down	Down	Down
**9**	FAM210A	No change	Down	Down	Down	Down	Down	Down
**10**	NDUFS7	No change	Down	Down	Down	Down	Down	Down
**11**	NDUFS2	No change	Down	Down	Down	Down	Down	Down
**12**	NDUFV1	No change	Down	Down	Down	Down	Down	Down
**13**	NDUFB7	No change	Down	Down	Down	Down	Down	Down
**14**	NDUFB4	No change	Down	Down	Down	Down	Down	Down
**15**	NDUFA7	No change	Down	Down	Down	Down	Down	Down
**16**	NDUFA10	No change	Down	Down	Down	Down	Down	Down
**17**	SLC25A22	No change	Down	Down	Down	Down	Down	Down
**18**	MDH2	No change	Down	Down	Down	Down	Down	Down
**19**	DBT	Down	Down	Down	No change	Down	Down	Down
**20**	AGK	No change	Down	Down	Down	Down	Down	Down
**21**	HK1	No change	Down	Down	Down	Down	Down	No change
**22**	UNC13A	No change	Down	Down	Down	Down	No change	Down
**23**	GNB5	No change	Down	NA	Down	Down	Down	Down
**24**	GLS	No change	No change	Down	Down	Down	Down	Down
**25**	NIPSNAP1	No change	No change	Down	Down	Down	Down	Down
**26**	FAM162A	No change	No change	Down	Down	Down	Down	Down
**27**	NDUFS5	No change	No change	Down	Down	Down	Down	Down

**28**	NDUFS3	No change	Down	Down	Down	Down	Down	No change
**29**	NDUFB9	No change	Down	Down	Down	Down	Down	No change
**30**	NDUFB6	No change	Down	Down	Down	Down	Down	No change
**31**	NDUFB5	No change	No change	Down	Down	Down	Down	Down
**32**	NDUFB10	No change	No change	Down	Down	Down	Down	Down
**33**	NDUFA9	No change	No change	Down	Down	Down	Down	Down
**34**	NDUFA8	No change	No change	Down	Down	Down	Down	Down
**35**	NDUFA6	No change	Down	Down	Down	Down	Down	No change
**36**	NDUFA5	No change	No change	Down	Down	Down	Down	Down
**37**	NDUFA12	No change	No change	Down	Down	Down	Down	Down
**38**	MFN2	No change	No change	Down	Down	Down	Down	Down
**39**	DNAJC19	No change	No change	Down	Down	Down	Down	Down
**40**	IDH3G	No change	Down	Down	Down	Down	Down	No change
**41**	FBXO41	No change	No change	Down	Down	Down	Down	Down
**42**	COX4I1	No change	No change	Down	Down	Down	Down	Down
**43**	CKMT1A	No change	Down	Down	Down	Down	Down	NA
**44**	SLC25A5	No change	No change	Down	Down	Down	Down	Down
**45**	SLC25A4	No change	Down	Down	Down	Down	Down	No change
**46**	ACO2	No change	No change	Down	Down	Down	Down	Down
**47**	KCNMA1	No change	Down	NA	NA	Down	Down	Down
**48**	SCAMP5	No change	No change	Down	Down	Down	No change	Down
**49**	VDAC2	No change	No change	Down	Down	Down	Down	No change
**50**	KCNB1	No change	Down	Down	Down	No change	Down	No change
**51**	RPS8	No change	No change	Down	No change	Down	Down	Down
**52**	PDK1	No change	Down	Down	Down	No change	No change	Down
**53**	OXCT1	No change	No change	Down	Down	Down	No change	Down
**54**	SLC25A36	Down	Down	No change	Down	NA	Down	No change
**55**	LMTK2	No change	Down	Down	Down	No change	Down	No change
**56**	RAB18	No change	No change	Down	No change	Down	Down	Down
**57**	OPA3	No change	Down	No change	Down	No change	Down	Down

**58**	CHCHD3	No change	No change	Down	Down	Down	Down	No change
**59**	IDH3B	No change	Down	Down	No change	Down	Down	No change
**60**	IDH3A	No change	No change	Down	Down	Down	Down	No change
**61**	ABHD16A	No change	No change	Down	No change	Down	Down	Down
**62**	DNAJC11	No change	No change	No change	Down	Down	Down	Down
**63**	CYC1	No change	No change	Down	Down	Down	Down	No change
**64**	MRPL2	No change	No change	Down	No change	Down	Down	Down
**65**	RAC1	No change	No change	No change	Down	Down	No change	Down
**66**	PPP3CC	No change	No change	Down	Down	No change	No change	Down
**67**	CPNE4	No change	No change	Down	No change	Down	No change	Down
**68**	SLC25A10	No change	Down	No change	No change	Down	Down	No change
**69**	AP2A2	No change	No change	Down	Down	Down	No change	No change
**70**	TRAPPC6B	No change	No change	Down	No change	Down	Down	No change
**71**	TARS2	Down	No change	No change	No change	No change	Down	Down
**72**	PCM1	Down	Down	No change	No change	No change	Down	No change
**73**	LONP1	No change	No change	No change	Down	Down	No change	Down
**74**	KATNB1	No change	No change	Down	No change	Down	Down	No change
**75**	TUFM	Down	No change	No change	No change	Down	Down	No change
**76**	NT5C3A	No change	No change	No change	No change	Down	Down	Down
**77**	COA1	Down	No change	Down	No change	No change	Down	No change
**78**	CELF2	No change	No change	NA	NA	Down	Down	Down
**79**	NECAP1	No change	Down	Down	Down	No change	No change	No change
**80**	RPS9	No change	No change	No change	No change	Down	Down	Down
**81**	RPS3A	No change	No change	Down	No change	Down	Down	No change
**82**	TMEM143	No change	No change	No change	Down	Down	Down	No change
**83**	SLC25A44	No change	No change	No change	Down	NA	Down	Down
**84**	SLC25A40	No change	No change	No change	Down	No change	Down	Down
**85**	ATP1B2	No change	No change	No change	No change	Down	Down	Down
**86**	SCCPDH	No change	No change	No change	No change	Down	Down	Down
**87**	RDH14	No change	No change	Down	Down	No change	Down	No change

**88**	SCO1	No change	No change	Down	Down	No change	Down	No change
**89**	MPV17	No change	No change	No change	No change	Down	Down	Down
**90**	PISD	No change	No change	No change	Down	No change	Down	Down
**91**	PTCD3	No change	No change	Down	No change	No change	Down	Down
**92**	OAT	No change	No change	No change	No change	Down	Down	Down
**93**	OCIAD2	No change	No change	No change	Down	No change	Down	Down
**94**	TIMM44	No change	No change	Down	No change	Down	Down	No change
**95**	MCAT	No change	No change	No change	No change	Down	Down	Down
**96**	GDPD1	No change	No change	Down	No change	Down	No change	Down
**97**	IARS2	No change	No change	No change	No change	Down	Down	Down
**98**	TSFM	No change	No change	Down	No change	Down	No change	Down
**99**	COX6C	No change	No change	Down	Down	Down	No change	No change
**100**	TMEM63B	Down	No change	Down	No change	No change	Down	No change
**101**	CCDC51	No change	Down	Down	Down	No change	No change	No change
**102**	AIFM1	No change	No change	No change	Down	Down	Down	No change
**103**	RPL13	Down	No change	No change	No change	Down	Down	No change
**104**	MRPL16	Down	No change	No change	No change	NA	Down	Down
**105**	MRPS7	Down	No change	No change	No change	No change	Down	Down
**106**	MRPS35	No change	No change	Down	No change	No change	Down	Down
**107**	MRPS15	Down	No change	No change	No change	Down	Down	No change
**108**	SLC4A7	No change	Down	NA	Down	No change	No change	No change
**109**	SLC25A25	No change	No change	NA	Down	No change	Down	No change
**110**	SSR3	No change	NA	No change	No change	No change	Down	Down
**111**	ALDH5A1	No change	No change	No change	No change	Down	No change	Down
**112**	COX20	No change	No change	Down	Down	No change	No change	No change
**113**	IBA57	No change	Down	No change	No change	No change	No change	Down
**114**	FAM126B	No change	No change	Down	Down	No change	No change	No change
**115**	PRPF19	No change	No change	No change	No change	No change	Down	Down
**116**	ACOX1	Down	No change	NA	No change	No change	No change	Down
**117**	MTHFD1L	No change	No change	NA	NA	Down	Down	No change

**118**	MAP1LC3B2	No change	No change	Down	No change	No change	Down	No change
**119**	IQSEC1	Down	No change	NA	NA	Down	No change	No change
**120**	ELOVL4	No change	No change	NA	No change	Down	Down	NA
**121**	RNF170	No change	No change	No change	No change	No change	Down	Down
**122**	CAMK2A	No change	No change	NA	Down	Down	No change	No change
**123**	AGAP2	No change	No change	NA	NA	Down	Down	No change
**124**	AARS2	No change	Down	No change	No change	Down	No change	No change
**125**	SSX2IP	No change	No change	No change	No change	Down	Down	No change
**126**	RPL32	No change	No change	No change	No change	Down	Down	No change
**127**	RPL26	No change	No change	Down	No change	No change	Down	No change
**128**	RPL24	No change	No change	No change	No change	Down	Down	No change
**129**	RPL23	No change	No change	Down	No change	No change	Down	No change
**130**	RPL18A	No change	No change	No change	No change	Down	Down	No change
**131**	RPL10	No change	No change	No change	No change	Down	Down	No change
**132**	RPS4X	No change	No change	No change	No change	Down	Down	No change
**133**	SLC24A2	No change	No change	NA	No change	Down	Down	No change
**134**	SLC35A4	No change	No change	NA	Down	No change	Down	NA
**135**	SPCS1	No change	NA	No change	No change	No change	Down	Down
**136**	RDH13	No change	No change	Down	No change	No change	No change	Down
**137**	RAI1	Down	Down	No change	No change	NA	No change	No change
**138**	FAM3C	No change	No change	Down	No change	No change	Down	No change
**139**	FAM210B	No change	Down	No change	No change	Down	No change	NA
**140**	NDUFAF7	No change	No change	No change	No change	Down	Down	No change
**141**	OCIAD1	Down	No change	No change	No change	No change	Down	No change
**142**	CYB5R1	No change	No change	No change	No change	No change	Down	Down
**143**	MPC2	No change	No change	No change	Down	No change	Down	No change
**144**	TOMM70	No change	No change	Down	Down	NA	NA	NA
**145**	TIMM21	No change	No change	No change	No change	Down	Down	No change
**146**	ACSL3	No change	No change	No change	No change	Down	Down	No change
**147**	LRRC59	No change	No change	No change	No change	Down	Down	No change

**148**	L2HGDH	No change	No change	No change	No change	Down	Down	No change
**149**	NUBPL	No change	No change	No change	No change	No change	Down	Down
**150**	EI24	Down	No change	Down	No change	NA	No change	NA
**151**	DYNLL2	No change	No change	No change	No change	Down	No change	Down
**152**	COX7A1	No change	NA	Down	No change	No change	Down	No change
**153**	ALG1	Down	No change	No change	No change	No change	No change	Down
**154**	ZW10	No change	No change	No change	No change	No change	Down	Down
**155**	CA14	No change	No change	Down	No change	No change	Down	No change
**156**	ATAD1	No change	No change	No change	Down	Down	No change	No change
**157**	MT-ATP8	No change	Down	Down	No change	No change	No change	NA
**158**	GOT2	No change	No change	Down	Down	No change	No change	No change
**159**	RPL8	No change	No change	No change	No change	Down	Down	No change
**160**	RPL34	No change	No change	No change	No change	Down	Down	No change
**161**	RPL3	No change	No change	No change	No change	Down	Down	No change
**162**	MRPS30	No change	No change	No change	No change	No change	Down	Down
**163**	FAM161A	No change	Down	NA	NA	NA	NA	NA
**164**	SFSWAP	No change	Down	No change	No change	NA	No change	NA
**165**	MYT1	No change	NA	NA	Down	NA	NA	NA
**166**	DHX30	No change	No change	Down	No change	No change	No change	No change
**167**	EPHB2	No change	No change	NA	No change	No change	No change	Down
**168**	FKBP8	No change	No change	NA	No change	Down	No change	No change
**169**	YTHDF2	No change	No change	No change	No change	No change	Down	No change
**170**	VWA8	No change	No change	No change	No change	No change	No change	Down
**171**	HSD17B12	No change	No change	No change	No change	No change	No change	Down
**172**	TEX10	No change	No change	No change	Down	No change	No change	No change
**173**	RDH11	No change	No change	No change	No change	No change	No change	Down
**174**	DDX5	No change	No change	No change	No change	No change	No change	Down
**175**	ME2	No change	No change	No change	No change	No change	No change	Down
**176**	ACSL6	No change	No change	No change	No change	Down	No change	No change
**177**	LRIF1	No change	NA	NA	NA	NA	Down	NA

**178**	SETD1A	Down	No change	No change	No change	NA	No change	NA
**179**	FMN1	No change	No change	Down	No change	No change	No change	No change
**180**	FAM3A	Down	No change	NA	No change	NA	NA	NA
**181**	SUPT16H	No change	Down	No change	No change	No change	No change	No change
**182**	EEF1E1	No change	No change	No change	No change	No change	Down	No change
**183**	CCDC90B	No change	No change	No change	Down	No change	No change	No change
**184**	DDX3Y	No change	No change	No change	No change	No change	No change	Down
**185**	RPL14	No change	No change	NA	NA	No change	No change	Down
**186**	RPL13A	No change	No change	No change	No change	No change	Down	No change
**187**	RPS27	No change	No change	No change	No change	No change	Down	No change
**188**	RPS2	No change	No change	No change	No change	No change	Down	No change
**189**	RPS15A	No change	No change	No change	No change	No change	Down	No change
**190**	MRPS14	No change	NA	NA	No change	NA	No change	Down
**191**	SEC22A	No change	No change	NA	No change	NA	Down	No change
**192**	TMED4	No change	No change	No change	No change	No change	No change	Down
**193**	SCFD1	No change	No change	No change	No change	No change	No change	Down
**194**	RTN4IP1	No change	No change	Down	No change	No change	No change	No change
**195**	RAB10	No change	No change	No change	No change	No change	Down	No change
**196**	POLDIP2	No change	No change	No change	No change	No change	Down	No change
**197**	WWP1	No change	No change	No change	No change	NA	Down	No change
**198**	OXA1L	No change	No change	NA	NA	No change	Down	No change
**199**	SSRP1	Down	No change	No change	No change	No change	No change	No change
**200**	STT3B	No change	No change	No change	No change	No change	Down	No change
**201**	DNAJA3	No change	No change	No change	No change	No change	Down	No change
**202**	DNAJB12	Down	No change	No change	No change	No change	No change	No change
**203**	TMEM126B	No change	Down	NA	No change	NA	No change	No change
**204**	CCDC127	No change	No change	No change	No change	No change	Down	No change
**205**	CPT2	No change	No change	No change	No change	No change	No change	Down
**206**	CPT1C	No change	No change	No change	Down	No change	No change	No change
**207**	SLC25A13	No change	No change	No change	No change	No change	Down	No change

**208**	TMCO1	No change	No change	No change	No change	No change	No change	Down
**209**	ACOT13	No change	No change	No change	No change	No change	Down	No change
**210**	RPL4	No change	No change	No change	No change	No change	Down	No change
**211**	RPL35	No change	No change	No change	No change	No change	Down	No change
**212**	RPL30	No change	No change	No change	No change	Down	No change	No change
**213**	RPL18	No change	No change	No change	No change	No change	Down	No change
**214**	RPL10A	No change	No change	No change	No change	Down	No change	No change
**215**	MRPS31	No change	No change	Down	No change	No change	No change	No change
**216**	MRPS12	No change	No change	Down	No change	No change	No change	No change

Ref*:* Bai, B., Vanderwall, D., Li, Y. *et al.* Proteomic landscape of Alzheimer's Disease
Mol Neurodegeneration 16, 55 (2021). https://doi.org/10.1186/s13024-021-00474-z
Dataset 1-3; Bai et al. Neuron 2020, 105:975-991:
Dataset 1: pooled TMT analysis of Banner Sun cohort without cell type normalization
Dataset 2: individual case TMT analysis of Banner Sun cohort
Dataset 3: individual case TMT analysis of Mount Sinai cohort
Banner Sun cohort: Human postmortem brain tissue samples
Mount Sinai cohort: frontal pole (https://www.nature.com/articles/sdata2018185)
Dataset 4: Wang et al. Anal. Chem. 2020, 92:7162-7170 (16-plex TMT analysis of Banner Sun cohort)
Datasets 5-6: Higginbotham et al. Sci. Adv. 2020, 6:eaaz9360 (dataset5: Table S2C; dataset6: Table S2F)
Dataset 7: Sathe et al. J. Neurochem. 2021, 156:988-1002
For the comparision of our retinal data against the literature brain cortex proteome data, proteins in each of the seven studies were regarded as DEP if the t-test p-value was less than 0.05, up or down regulated based on the log fold change AD/control (>0 up-regulated, <0 down-regulated).
Proteins p-values equal or higher than 0.05 were regarded as unchanged.
